# Functional Evolution of Mammalian Odorant Receptors

**DOI:** 10.1371/journal.pgen.1002821

**Published:** 2012-07-12

**Authors:** Kaylin A. Adipietro, Joel D. Mainland, Hiroaki Matsunami

**Affiliations:** 1 Department of Molecular Genetics and Microbiology, Duke University Medical Center, Durham, North Carolina, United States of America; 2 Department of Neurobiology and Duke Institute for Brain Sciences, Duke University Medical Center, Durham, North Carolina, United States of America; University of Michigan, United States of America

## Abstract

The mammalian odorant receptor (OR) repertoire is an attractive model to study evolution, because ORs have been subjected to rapid evolution between species, presumably caused by changes of the olfactory system to adapt to the environment. However, functional assessment of ORs in related species remains largely untested. Here we investigated the functional properties of primate and rodent ORs to determine how well evolutionary distance predicts functional characteristics. Using human and mouse ORs with previously identified ligands, we cloned 18 OR orthologs from chimpanzee and rhesus macaque and 17 mouse-rat orthologous pairs that are broadly representative of the OR repertoire. We functionally characterized the *in vitro* responses of ORs to a wide panel of odors and found similar ligand selectivity but dramatic differences in response magnitude. 87% of human-primate orthologs and 94% of mouse-rat orthologs showed differences in receptor potency (EC50) and/or efficacy (dynamic range) to an individual ligand. Notably dN/dS ratio, an indication of selective pressure during evolution, does not predict functional similarities between orthologs. Additionally, we found that orthologs responded to a common ligand 82% of the time, while human OR paralogs of the same subfamily responded to the common ligand only 33% of the time. Our results suggest that, while OR orthologs tend to show conserved ligand selectivity, their potency and/or efficacy dynamically change during evolution, even in closely related species. These functional changes in orthologs provide a platform for examining how the evolution of ORs can meet species-specific demands.

## Author Summary

The mammalian odorant receptor repertoire has been subjected to significant gene duplication and gene loss between species, presumably to adapt to the environment of an organism. However, even in distantly related species, a clear orthologous relationship exists for many genes. While ligands have been identified for several ORs, many of these receptors remain uncharacterized, especially in species other than human and mouse. Due to this paucity of functional data, it is assumed that ORs with similar sequence share functional characteristics. Here we investigate the functional evolution of OR orthologs—genes related via speciation—and OR paralogs—genes related via a duplication event—to provide insight as to how this large gene family has evolved. We show that OR orthologs have similar ligand selectivity to a panel of odors but differ in response magnitude. Additionally, orthologs respond to a common ligand more often than human OR paralogs, but there are vast differences in the potency and efficacy of individual receptors. This result stresses the broad importance of combining evolutionary genomics and molecular biology approaches to study gene function.

## Introduction

Odorant receptors (ORs) expressed at the cell-surface of olfactory sensory neurons (OSNs) in the main olfactory epithelium detect chemical cues in the proximate environment. The ability to detect these cues is crucial for survival of an individual and a species; odorants can signify favorable or toxic food sources, mating preferences, predators and habitat [1], [2]. The ecological niche that an animal inhabits is directly associated with the OR repertoire in each species [3], but we do not know how the functional OR repertoire evolves to maximize an animals' fitness. As a first step to understand the functional evolution of ORs, it is essential to compare OR function in related species, paying attention to the evolutionary relationship of each tested receptor.

Gene orthology is a key concept in evolutionary and functional genomics. Here we define orthologs as genes derived from a single ancestral gene that diverged since a speciation event; this is in contrast to paralogs, which are genes related via gene duplication [4], [5] (Figure S1). Orthologous genes typically perform equivalent–if not identical—functions, especially when comparing closely related species, while paralogs are thought to be more divergent in function [4]–[7]. Indeed, this is a key assumption in a wide variety of biological research, as it allows research from model organisms to translate into health interventions in humans [7]. Comparisons of sequenced genomes show that many orthologous genes can be identified between divergent species, but the functional equivalency of the vast majority have not been experimentally tested [4].

The OR repertoire suffered extensive gains and losses of genes between species, resulting in a significant decline in the number of putatively functional ORs in primate species when compared to the OR repertoires in other mammals [8]–[12]. Comparisons of high-coverage primate genomes revealed that the size of the functional OR repertoire and percentage of pseudogenized ORs is quite similar between human, chimpanzee (Great Ape) and rhesus macaque (Old World Monkey) [12], [13]. However, between humans and chimpanzees, approximately 25% of the OR repertoire exists in only one species [13], suggesting adaptation to differing environments to meet species-specific demands [3], [14], [15]. Importantly, many ORs have clear orthologs in closely related species [13]. Sequence similarity of ORs is often used as a proxy for functional variability [9], [16]–[19], but this assumption remains largely untested [9], [20] due to the paucity of functional data matching ORs with ligands. While full-length sequence comparison provides insight into the evolutionary relationship of ORs, it is thought to have less predictive value about the binding sites of these receptors [9]. Man et al. (2004) proposed a set of 22 amino acids important for ligand binding under the assumption that orthologs will have more similar odor specificities than paralogs, showing a greater conservation in amino acids residues at odor-binding sites than across the entire coding region [9], [17].

Several studies have examined changes in ligand selectivity and sensitivity of OR orthologs, but these studies were limited to a single receptor set [17], [21]–[23]. It is still unclear whether or not this change in sensitivity of orthologs is restricted to a specific family of ORs or is a more general phenomenon across all OR orthologs. To understand how the olfactory system has evolved and how the human OR repertoire was shaped, we must identify the functional changes of orthologous ORs between species. With the development of a high-throughput *in vitro* assay for OR function, we are now able to directly test how well OR sequence similarity predicts function [24]–[26]. Here we conduct the first multi-receptor comparison of ligand selectivity and sensitivity of OR orthologs in primates and rodents and, further, ask if orthologs respond to a common ligand more often than OR paralogs from the same subfamily.

## Results

### Putatively functional OR orthologs are broadly distributed

Starting with a set of human ORs previously matched to at least one ligand (deorphaned ORs) [27], we searched the chimpanzee and rhesus macaque genomes for putatively functional orthologous gene sets where there was no evidence of gene duplication in either species (one-to-one ortholog) [13]. We identified 18 ORs that have putatively functional orthologs: 12 orthologous trios (in all three species), five human-chimp duos that lack orthologs in macaque and one human-macaque duo that lacks a chimp ortholog. Additionally, we identified 17 mouse-rat ortholog sets where there is at least one known ligand for the mouse OR (for sequences of ORs used in functional experiments, see Table S1) [27], [28].

**Figure 1:**
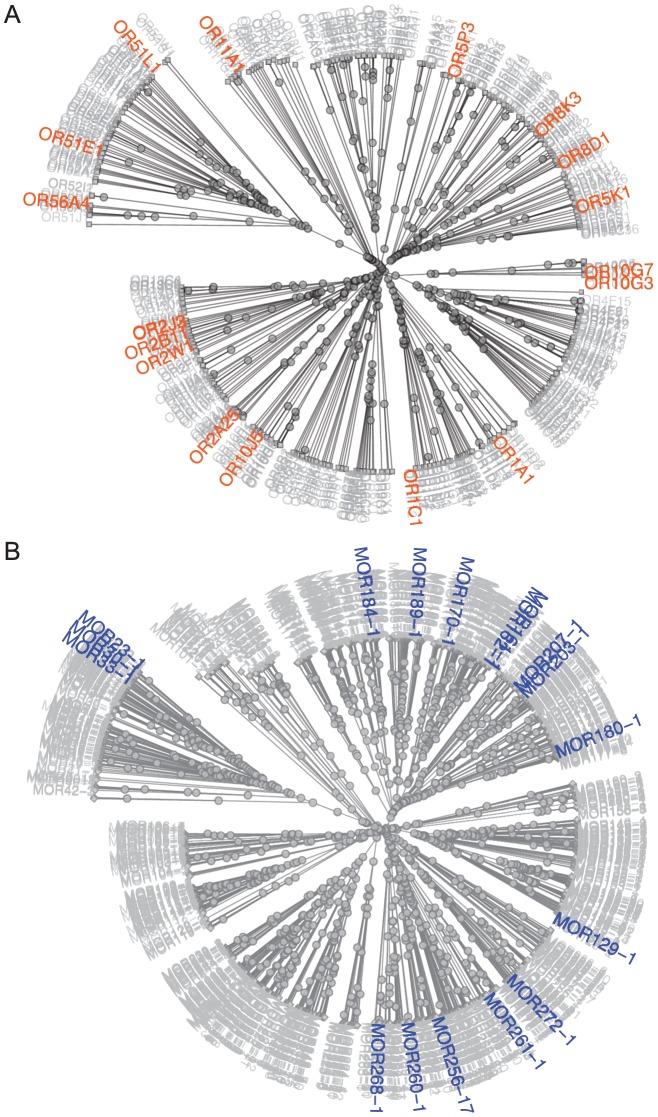
Distribution of primate and rodent OR orthologs with known ligands among OR classes and families. (A) Unrooted tree of human ORs based on similarity of amino acid properties. 12 human-chimpanzee-rhesus macaque orthologs, 5 human-chimp orthologs and 1 human-macaque ortholog are highlighted in orange. (B) Unrooted tree of mouse ORs based on similarity of amino acid properties. 17 mouse-rat orthologs are highlighted in blue. Receptor similarity was quantified using Grantham's amino acid property scale [29].

Using the similarity of amino acid properties [29], we constructed a tree of all 390 putatively functional human ORs; the 18 orthologous primate sets used for analysis are highlighted on the tree (Figure 1A). These ORs represent Class I and II receptors, seven of the 13 families described by Hayden et al. (2010) [3] and contain human ORs shown to be both broadly and narrowly tuned to odors [27]. Our mouse-rat orthologs also cover both Class I and II ORs and represent 17 of the 228 families described by Zhang and Firestein (2002) [18] (Figure 1B). This suggests that our set of ORs is not significantly biased towards any particular family of ORs.

Using the Jukes-Cantor model of nucleotide substitution rates [30], comparison between orthologs are consistent with the expected phylogeny between species. Human-chimp orthologs are the most similar, followed by human-macaque and chimp-macaque orthologs, with mouse-rat orthologs being the most divergent (Figure S2A, Table S2). We used Grantham's distance to compare amino acid similarity of the entire open reading frame (ORF) among ortholog sets [29]. Our results show our OR sets are not biased to ORs with highly similar amino acid substitutions (Figure S2B, Table S2). Additionally, we compared the Grantham's distance of the 22 amino acids predicted to be involved in ligand binding by Man et al. (2004) [17]. We found that all human-chimp orthologs are identical at these 22 positions, while six human-macaque orthologs and two mouse-rat orthologs differ, but the amino acid substitutions are fairly conservative (Table S2, Figure S3A).

### Selective pressures within OR coding regions

Previous literature suggests there is evidence for both positive and purifying selection in the OR repertoire, so to determine if our OR sets broadly represent genes evolving under different selective pressures, we calculated the ratio of nonsynonymous to synonymous substitutions (*ω* or dN/dS) for our 18 orthologous primate sets and for each of the putatively functional 259 human-chimp or 152 human-macaque 1∶1 orthologs from Go and Niimura (2008) [13] (Figure S4). In the absence of selective pressure, the rates of synonymous substitutions per synonymous site (dS) are equal to that of nonsynonymous substitutions (change the resulting amino acid) per nonsynonymous site (dN), thus, *ω* = dN/dS = 1; *ω*<1 suggests evidence of purifying selection and *ω*>1 indicates evidence of positive selection acting on a gene [31]. The distributions of *ω* are significantly different between human-chimp and human-macaque (median human-chimp *ω* = 0.608; median human-macaque *ω* = 0.319; z = −9.61, p<0.001, Wilcoxon Rank Sum) (Figure S4). All human-macaque gene pairs have *ω*<1, while the human-chimp gene pairs show a wide distribution of *ω* values (for branch-test and branch-site tests, see [13]). The median *ω* value of mouse-rat orthologs was 0.124, consistent with previous literature [32].

### Similar ligand selectivity, differences in magnitude within an orthologous set

**Figure 2:**
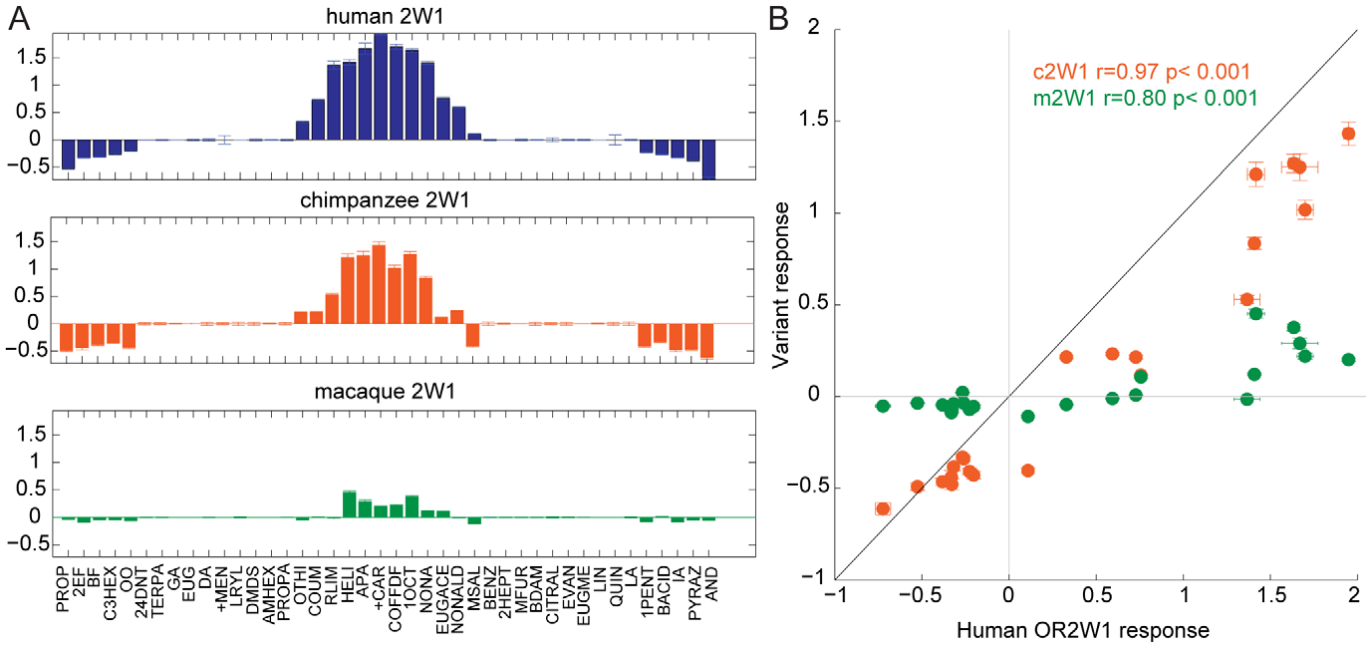
Response of OR orthologs to 42 chemically diverse odors. (A) Tuning curves of human, chimp and macaque OR2W1 orthologs tested against 42 odors using a cAMP-mediated luciferase assay [26]. Odorants are ordered along the x-axis according to the response elicited from the human OR2W1, with the best ligands closer to the center. Y-axis represents the luciferase response to an odor at 100 µM (n = 3, ± S.E.). Negative values on the y-axis indicate the odor elicited an inhibitory response on OR signaling. See Figure S6 for additional tuning curves. (B) Response of chimp and macaque OR2W1 orthologs (variant responses) plotted against the human OR2W1 response using the data from (A). X-axis and y-axis are response in luciferase assay at 100 µM (n = 3, ± S.E.). The black line represents the unit-slope line. Odor abbreviations in Table S3.

To determine if gene orthology accurately predicts the functional properties of orthologs, we expressed each OR in a heterologous cell system, using a cyclic adenosine monophosphate (cAMP)-mediated luciferase reporter gene to assay the function [26]. We tested each orthologous OR set against a panel of chemically diverse odors to compare their ligand selectivity and responses. We chose a panel of 42 chemically diverse odors to represent most of “odor space” using a method described previously [27], [33], and tested these chemicals in triplicate at 100 µM (Figure S5, Table S3). Within an OR set, the response of orthologs across the panel of odors was consistent, but with differences in the overall magnitude of the response of an OR (negative values on the y-axis indicate an odor elicited an inhibitory response) (Figure 2, Figure S6). For example, human OR2W1 responded to 12 ligands, while chimp OR2W1 responded to the same 12 odors but with a diminished magnitude. In some instances the response of the human and mouse ORs could not be used to predict the OR function in other species, as the ligands tested did not activate the orthologous receptors. To address concerns that there is a species-specific interaction between ORs and variants of the Receptor Transporting Protein-1 short form (RTP1S, the accessory protein necessary for functional expression of ORs at the cell surface) we tested the functional consequence of swapping human and mouse versions of RTP1S with four human and four mouse ORs [25], [26], [34]. With the tested ORs, our data did not support the idea that mouse RTP1S was the most efficient for trafficking only mouse ORs and human RTP1S was the most efficient at trafficking the human ORs (F(7,80) = 1.03, p = 0.416, 2-way ANOVA) (Figure S7).

### Sequence similarity and the functional properties of OR orthologs

**Figure 3:**
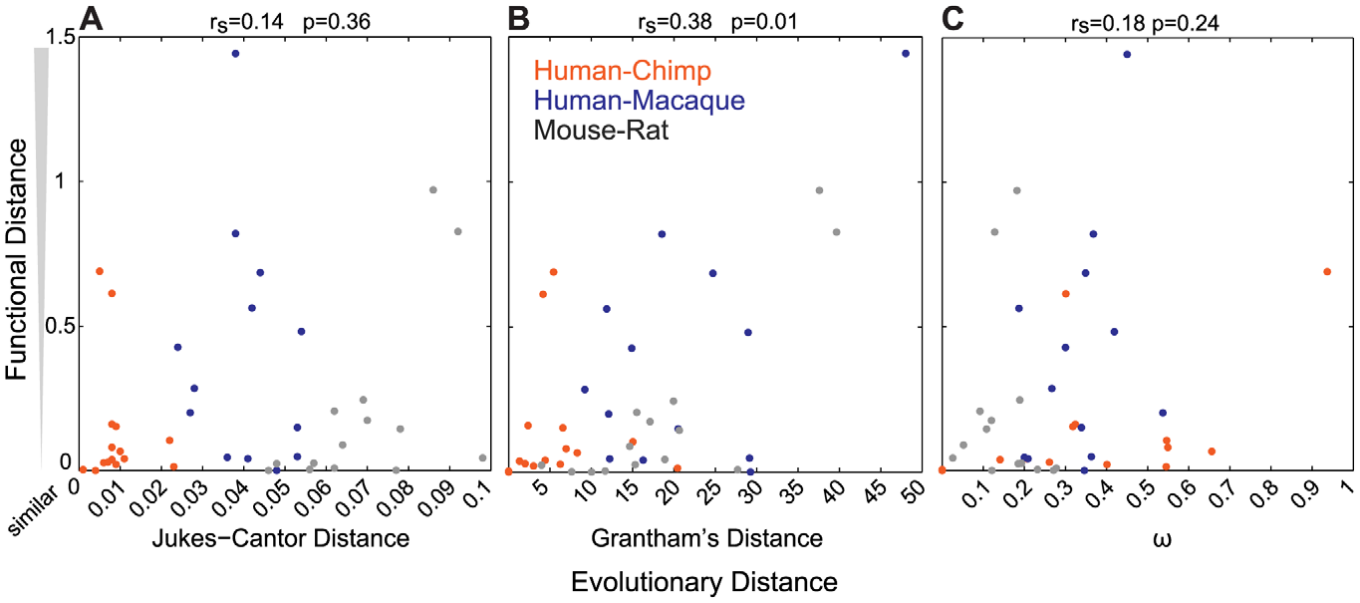
Sequence similarity does not accurately predict the functional properties of ORs. For each OR pair, the (A) Jukes-Cantor (nucleotide) distance (B) Grantham (amino acid) distance and (C) *ω* (dN/dS) is plotted against the functional distance, as defined by the correlation (1-R, Table S2) of the response across the 42-odor panel for ORs responding to more than three odors. *ω* values for human-chimp OR2W1 were included in the calculation but eliminated from the plot for better visual representation. Values closer to zero are more similar in sequence and function. Jukes-Cantor and *ω* do not correlate with functional distance (J-C r_s_ = 0.14, p = 0.36; *ω*, r_s_ = 0.18, p = 0.24) while Grantham's distance has a correlation to functional distance (r_s_ = 0.38, p = 0.01).

To determine how well sequence similarity among orthologs can predict the function of ORs, we plotted the relationship of Jukes-Cantor distance (J-C, nucleotide), Grantham's distance (amino acid similarity) using the entire ORF or 22 amino acid positions predicted to be involved in ligand binding, and *ω* (dN/dS) versus the functional distance of the orthologous sets. Here we define the functional distance as the correlation between OR responses across the 42 odor panel, where a receptor responded to three or more odors. Jukes-Cantor and pairwise *ω* values do not correlate with functional distance (J-C, r_s_ = 0.14, p = 0.36; *ω*, r_s_ = 0.18, p = 0.24, Spearman's correlation) (Figure 3A, 3C). Amino acid similarity using the ORF has a correlation to functional distance (r_s_ = 0.38, p = 0.01, Spearman's correlation) and amino acid similarity using predicted binding residues is similar but slightly less significant (r_s_ = 0.32, p = 0.04) (Figure 3B, Figure S3B). Additionally, we did not find a correlation between sequence similarity and the number of ligands that activate each OR (*ω*, r_s_ = 0.02, p = 0.92; J-C, r_s_ = 0.10, p = 0.49; Grantham ORF, r_s_ = 0.03, p = 0.83; Grantham 22AA, r_s_ = 0.02, p = 0.87, Spearman's correlation, data not shown).

Finally, the removal of primate OR sets that lack orthologs in one of the three primate species (five human-chimp duos and one human-macaque duo) from our analysis did not change the overall conclusions when comparing Jukes-Cantor, Grantham and *ω* values to the functional distance, suggesting that inclusion of these data does not bias our results.

### Changes in sensitivity to individual ligands within an orthologous set

**Figure 4:**
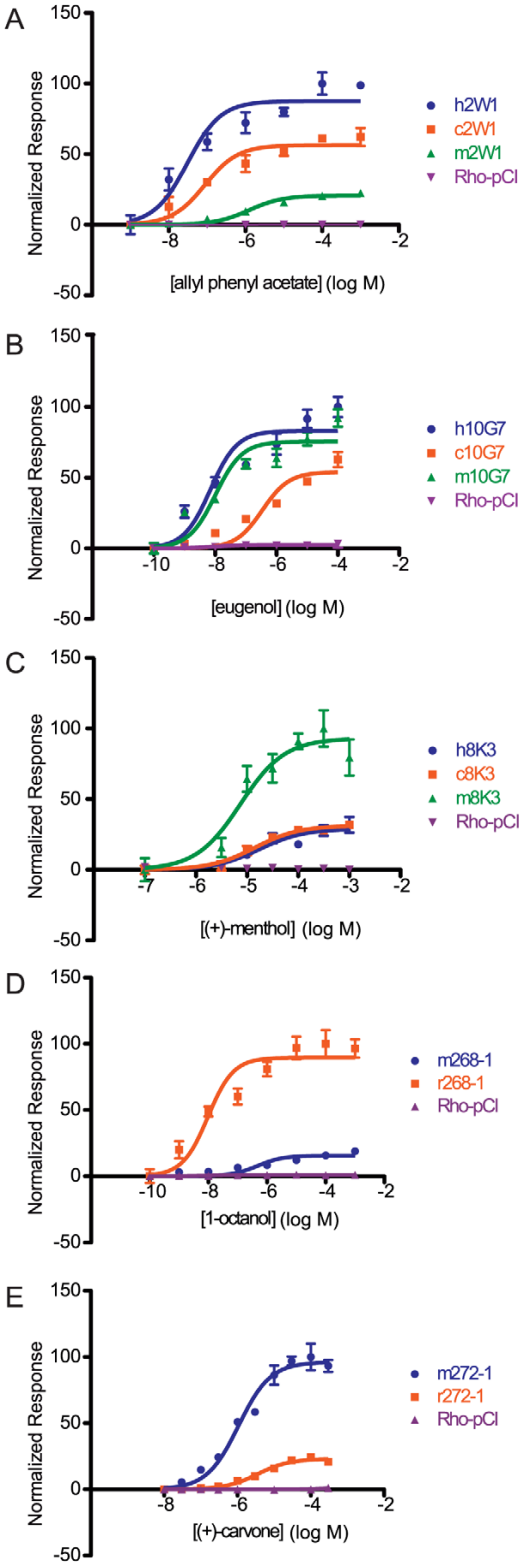
Dose-response curves of an OR ortholog set to a given ligand. (A) Primate OR2W1 orthologs to allyl phenyl acetate. (B) Primate OR10G7 orthologs to eugenol. (C) Primate OR8K3 orthologs to (+)-menthol. (D) Rodent 268-1 orthologs to 1-octanol. *(E)* Rodent 272-1 orthologs to (+)-carvone. X-axis is the concentration of a given odor in Log Molar. Y-axis is normalized response (n = 3, ± S.E.M.). Human (h), chimpanzee (c) and rhesus macaque (m) in primate ortholog sets; mouse (m) and rat (r) for rodent ortholog sets. Vector control is Rho-pCI. See Figure S9 for additional dose-response data.

We next wanted to examine the functional changes in sensitivity of orthologs to individual ligands by testing each ortholog across a range of concentrations. We selected a single odor for a given OR to construct a representative dose-response curve, fit the data to a sigmoid curve, and then compared the response of the human allele against the primate orthologs and the mouse allele with the rat ortholog using an extra sum of squares test. Looking at both the potency (EC50) and efficacy (dynamic range) of each OR to a particular ligand, an orthologous pair was classified as either indistinguishable, hyper/hypo functional (one OR had both a lower potency and efficacy), or undefined (orthologs were different but potency and efficacy did not change concordantly) (Figure S8). Within each OR set, we saw dramatic differences in the overall potency and efficacy to a particular ligand (Figure 4, Figure S9). For example, human and chimp OR8K3 orthologs are indistinguishable in their response to (+)-menthol (Extra sum of squares test, F(3,36) = 0.13, p = 0.944), but human and chimp OR8K3 are hypofunctional in comparison to macaque OR8K3 when tested with the same ligand (Extra sum of squares test, human to macaque F(3,36) = 15.16, p<0.001, chimp to macaque F(3,36) = 17.40, p<0.001) (Figure 4C, Table S4). Macaque OR10G7 and human OR10G7 are indistinguishable in response to eugenol (Extra sum of squares test, F(3,36) = 0.97, p = 0.418), but chimp OR10G7 is hypofunctional to human (Extra sum of squares test, F(3,36) = 54.54, p<0.001) and macaque (Extra sum of squares test, F(3,36) = 84.82, p<0.001) (Figure 4B, Table S4). Additionally, mouse and rat ORs showed differential responses to a given ligand (Figure 4D, 4E).

**Figure 5:**
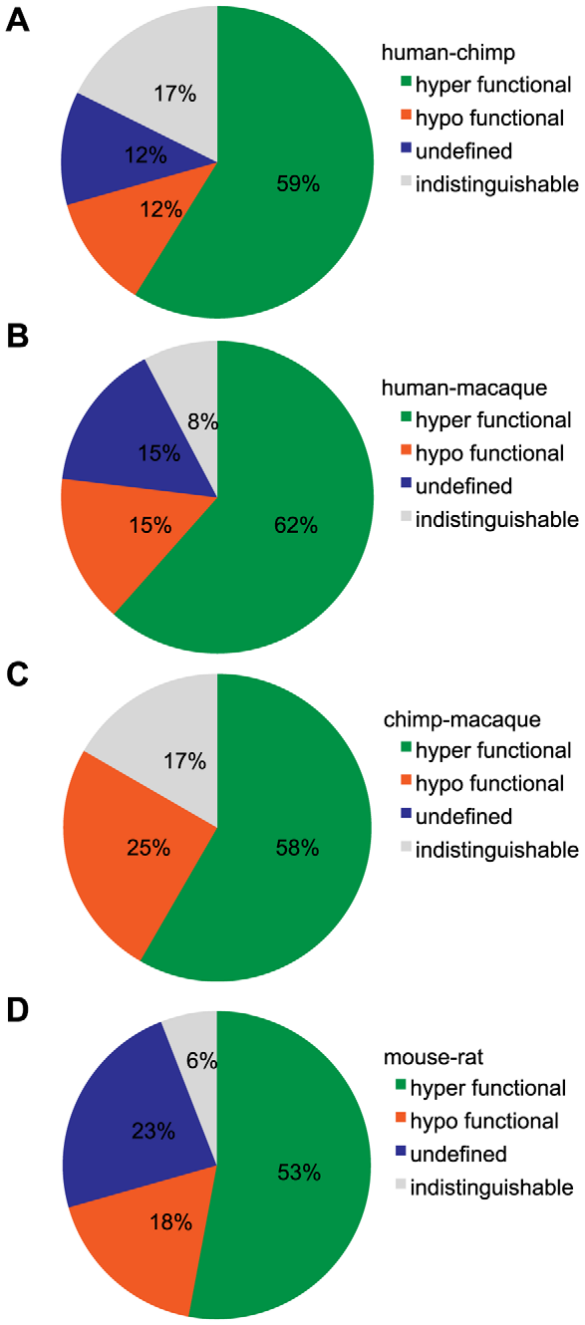
Classification of functional changes in dose-response between orthologous OR pairs. (A) human-chimp, (B) human-macaque, (C) chimp-macaque and (D) mouse-rat. Using both the potency (EC50) and efficacy (dynamic range) to a particular ligand, an orthologous pair is classified as either indistinguishable, hyper/hypo functional (one OR had both a lower potency and efficacy) or undefined (orthologs were significantly different but potency and efficacy did not change concordantly). Each pie chart refers to the first species in the comparison (*e.g.* (A) human is ___ to chimp). For visual explanation, see Figure S8.

If we define functional differences as changes in potency and efficacy of a common ligand, comparison of our set of human ORs to primate orthologs revealed functional differences 87% of the time, while mouse-rat orthologs differed 94% of the time (Figure 5A–5D). If our human ORs are randomly compared to other human alleles of the same OR, their dose-response curves are functionally different 25% of the time (Mainland et al., unpublished). In other words, sequence variation within the human population does not alter receptor function as much as sequence variation between orthologs in closely related species.

### Responses of OR orthologs and paralogs from the same subfamily to a common ligand

**Figure 6:**
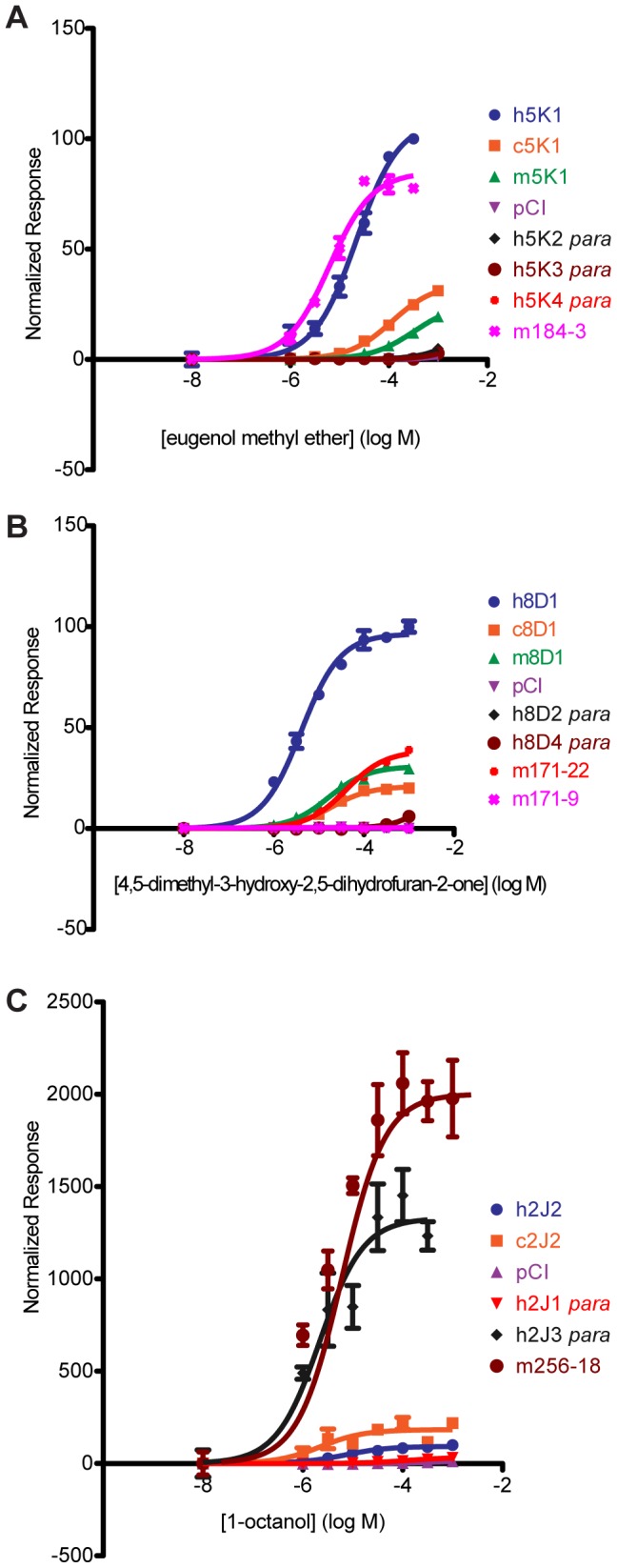
Dose-response curves of OR orthologs and paralogs to a given ligand. (A) OR5K1 orthologs and 5 K subfamily to eugenol methyl ether. (B) OR8D1 orthologs and 8D subfamily members to 4,5-dimethyl-3-hydroxy-2,5-dihydrofuran-2-one. (C) OR2J2 orthologs and 2J subfamily members to 1-octanol. X-axis is the concentration of a given odor in Log Molar. Y-axis is normalized response (n = 3, ± S.E.M.). Human (h), chimpanzee (c) and rhesus macaque (m), mouse receptors (m+number); *para* indicates a receptor that is a paralog to the human reference OR. Vector control is Rho-pCI. See Figure S11 for additional dose-response data from orthologs and subfamily members.

To further address the question of how well orthology predicts function, we compared the response of orthologous ORs from closely related species to that of orthologs from more distant species and to ORs that are classified in the same subfamily based upon sequence similarity. For our sets of primate orthologs, we identified the putative human-mouse ortholog, as defined by the reciprocal ‘best-hit’ with >80% amino acid identity, and human OR paralogs—members of the same subfamily [11], [35]—and tested these receptors against a common ligand. Comparison of sequences using Neighbor-Joining phylogenetic analyses showed that our primate orthologs are most similar to the human reference OR, while the mouse best-hit ORs are more distantly related. Human paralogs have a unique relationship for each OR group (Figure S10), but are generally less related than the primate orthologs. In sum, our ortholog and paralog assignment is congruent with speciation and gene duplication events. Overall, we find that orthologs respond to a common ligand 82% of the time while human OR subfamily members respond to a common ligand 33% of the time. Species-specific comparison of orthologs showed human-chimp orthologs respond to a common ligand 93% (14/15) of the time, human-macaque 67% (8/12), and human-mouse (10/12) 83%. Using the above criteria to define changes in function to a given ligand, we again find significant differences in the potency and efficacy of each OR within a group (Figure 6, Figure S11, Table S5).

For example, human OR5K1 and mouse ortholog mOR184-3 respond to eugenol methyl ether (human to mouse F(3,39) = 21.59, p<0.001, undefined) but human OR5K1 is hyperfunctional to both chimp and macaque OR5K1 orthologs. None of the three human 5K family paralogs respond to this ligand (Figure 6A, Table S5). Human OR8D1 is hyperfunctional to chimp and macaque OR8D1 orthologs, while human paralogs 8D2 and 8D4 do not respond (Figure 6B, Table S5). Mouse ortholog mOR171-22 is hypofunctional to OR8D1 (F(3,42) =  873.69, p<0.001) while mOR171-9 does not respond.

From our analysis, orthologs respond to a common ligand more often than OR paralogs of the same subfamily, albeit with differences in sensitivity, suggesting that OR paralogs in the same subfamily may show distinct ligand selectivity.

**Figure 7:**
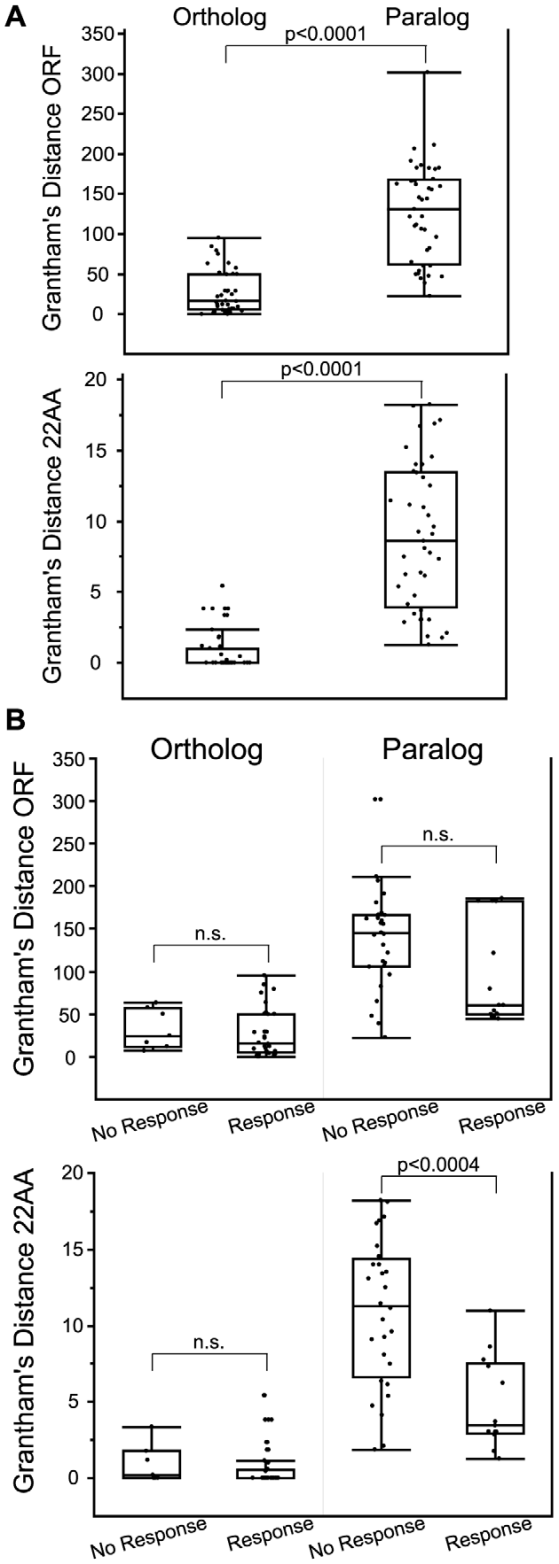
Comparison of amino acid similarity among orthologs and paralogs. Grantham's distance was calculated for OR open reading frame (ORF) and for 22 predicted binding residues (22AA) used in Man et al. (2004) [17]. (A) Orthologs are paralogs are significantly different using both ORF and 22AA (z = −6.61, p<0.0001 ORF; z = −7.35, p<0.0001 22AA, Wilcoxon Rank Sum). (B) Grantham's distance ORF and 22AA for orthologs and paralogs segregated by response to a common odor. Amino acid similarity of the 22 predicted binding residues (22AA) was significantly different for paralogs, with responding paralogs being more similar in sequence (z = −3.54, p<0.0004, Wilcoxon Rank Sum). n.s. is not significant. Box plots show minimum values,10% and 25% quantiles, median, 75% and 90% quantiles, and maximum values for each data set. Each ortholog and paralog is compared to the reference human OR in that group (listed first in Table S6).

Orthologs were more similar than paralogs when measuring Grantham's amino acid similarity using both the entire ORF and the 22 predicted binding residues (z = −6.61, p<0.0001 ORF; z = −7.35, p<0.0001 22AA, Wilcoxon Rank Sum) (Figure 7A, Table S6, Figure S12). Orthologs that responded to the same odor as the human reference OR were not significantly different from orthologs that did not respond when comparing amino acid similarity of both the ORF and the 22 predicted binding residues (z = 0.89, p = 0.37 ORF; z = 1.22, p = 0.22, 22AA, Wilcoxon Rank Sum) (Figure 7B). This suggests that amino acid similarity did not accurately predict OR function among orthologs. While amino acid similarity of the ORF did not predict the response of paralogs (z = −1.47, p = 0.14, Wilcoxon Rank Sum), the amino acid similarity of the 22 predicted binding residues was significantly different, with responding paralogs being more similar in sequence (z = −3.54, p<0.0004, Wilcoxon Rank Sum) (Figure 7B lower panel, Table S6). Our data suggest that comparing the 22 residues involved in ligand binding is better than the entire ORF when predicting the response of OR paralogs.

### OR surface expression does not predict receptor response

**Figure 8:**
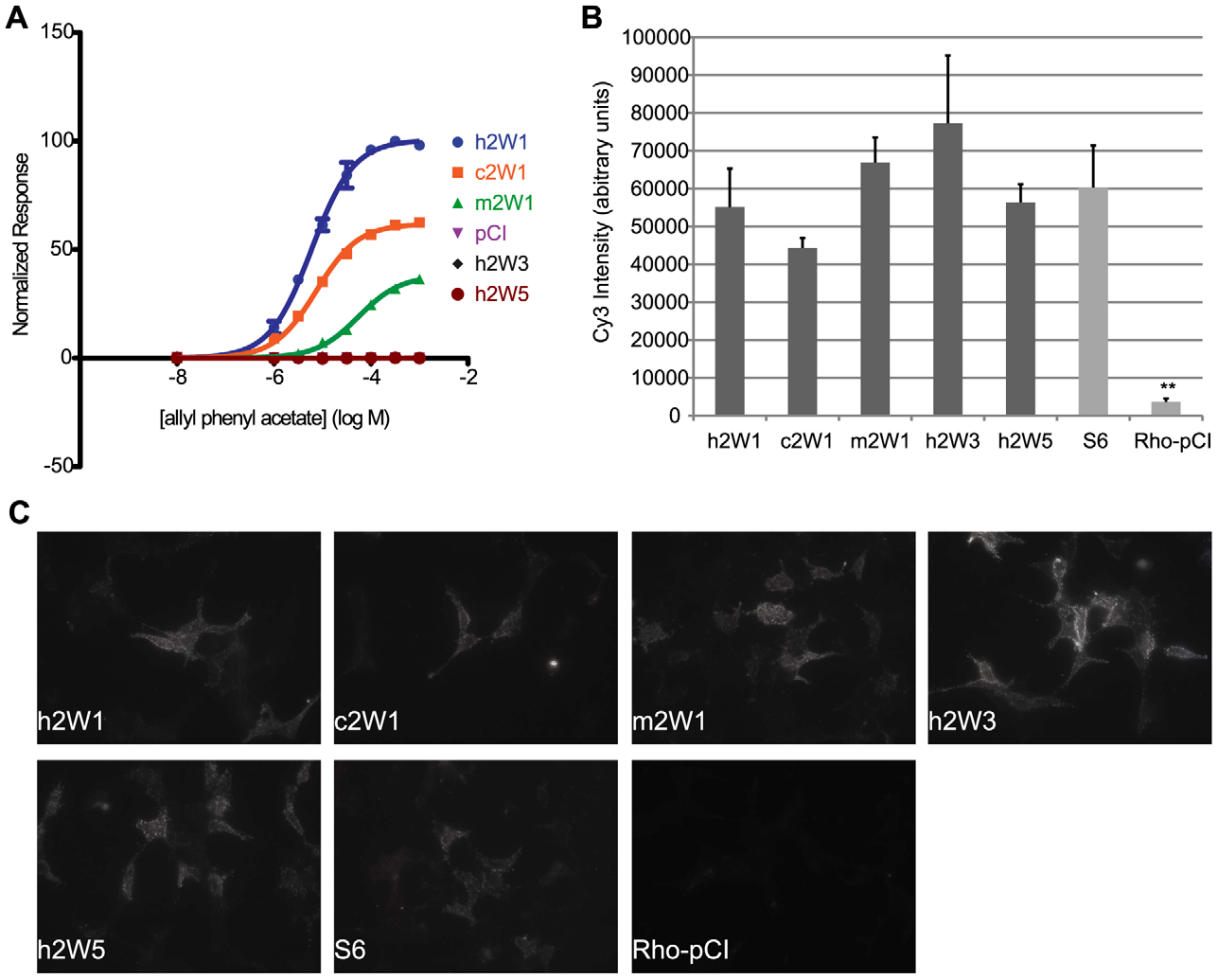
Cell surface expression does not predict function of ORs. (A) Primate OR2W1 orthologs and 2W subfamily members to allyl phenyl acetate. (B) Quantification of live cell-surface expression of each receptor. ** p<0.01 when compared to hOR2W1. Y-axis denotes the average Cy3 intensity in arbitrary units (a.u.) (n = 3, ± S.E.M.). S6 is positive control and Rho-pCI is negative control. (C) Representative image of live cell-surface staining for each receptor. For additional live-cell surface staining, see Figure S13, Table S7.

To determine if individual differences in receptor activity are influenced by the amount of receptor at the cell surface, we assessed cell surface expression of each OR [36], [37]. Using fluorescent immunocytochemistry in live cells, we measured the Cy3 signal intensity of each ortholog and paralog in our receptor set and compared them against the human counterpart. Within each set of receptors, we found cell-surface signal levels did not predict the potency of the OR to a single ligand (Figure 8, Figure S13). For example, human, chimp and macaque OR2W1 are similar in their receptor tuning with differences in response magnitude (Figure 2A, 2B) and have differences in EC50 values to a common ligand, allyl phenyl acetate, while human paralogs OR2W3 and OR2W5 do not respond to the common ligand (Figure 4A, Figure 8A). Surface labeling of human OR2W1 was not significantly different from either orthologs or paralogs (Figure 8B, 8C, Table S7), consistent with the idea that surface expression levels of ORs do not predict sensitivity of ORs. No OR surface expression results in no response to known ligands [34]. However, ORs with very intense surface staining are not necessarily responsive to a common ligand, nor are they the most sensitive to that ligand if they do respond. ORs with very few detectable receptors at the surface still showed functional activity, suggesting receptor amount does not dictate response (Figure S13).

## Discussion

Here we showed that OR orthologs are similarly tuned within an OR set, but that dramatic differences in efficacy and potency to a common odor are frequent. These functional changes are not specific to the primate lineage where significant gene loss has impacted the size of the OR repertoire and a decline in the relative importance of the olfactory system is commonly assumed [8], [10], [13], [14], [38], as we see similar changes in rodent orthologs. Comparison of primate orthologs, more distantly related orthologs (human-mouse) and human OR paralogs of the same subfamily suggest that orthologs respond to a common ligand more often than other subfamily members. This idea is consistent with the approach used by Man et al. (2004, 2007) comparing residues conserved in orthologs and differing in paralogs to predict 22 amino acid residues involved in ligand binding [9], [17]. In our example of human OR8D1 and mouse orthologs mOR171-22 and mOR171-9, we see that mOR171-22 responds to a common ligand while mOR171-9 does not (Figure 6B). Comparison of the predicted binding residues shows that mOR171-22 is identical to human OR8D1 at all 22 sites, while mOR171-9 differs at one position (Figure S12, Table S6); each receptor has an overall amino acid identity of 85% to the human ortholog. While the amino acid similarity using the ORF or the 22 predicted binding residues did not predict OR ortholog response, the amino acid similarity of the 22 predicted binding residues was significantly different for paralogs that responded to a common ligand, (z = −3.54, p<0.0004, Wilcoxon Rank Sum) (Figure 7B lower panel, Table S6). Thus, the identification of a true ‘functional ortholog’ must be supported by both bioinformatics and experimental approaches.

A significant portion of mammalian ORs are orphan receptors, although progress has been made in matching individual OR-ligand interactions [9], [27]. Not all ORs with an intact ORF are necessarily functional. Similarities between coding sequences are used to predict functionality in lieu of real experimental data, thus, ORs grouped into the same subfamily are thought to share similar functional properties based upon sequence homology [10], [38]–[40]. This has also led to the idea that OR orthologs from different species will maintain the same olfactory capabilities [9], [16]. We have taken the approach of using an evolutionary analysis to examine the relationship of OR-ligand interactions.

Several studies have looked at changes in ligand selectivity and sensitivity of OR orthologs using a single receptor. One functional study identified 18 ligands that activate human paralogs OR1A1 and OR1A2. Human OR1A1 and mouse ortholog Olfr43 shared 9 common odors; twelve amino acids thought to influence ligand binding properties overlap with the prediction from Man et al. (2004) [17], [21]. Krautwurst et al. (1998) showed that the orthologous mouse I7 and rat I7 receptors show changes in the fine-tuning of ligand selectivity, with mouse I7 preferring heptanal to octanal, and the reverse was shown for the rat ortholog [22].

Zhuang et al. (2009) showed that OR7D4 orthologs from many primate species differed in potency and efficacy to a common ligand [23], but the question remained whether these functional differences extended to many ORs or if OR7D4 was a special case. Androstenone and androstadienone, the steroid ligands for OR7D4, are found in human male sweat, urine and semen, and have extreme perceptual differences in the human population [41], [42]. Additionally, these odorous steroids have been linked to changes in physiological response in both males and females, making them a unique case [43], [44]. Our data suggest that many ORs show dynamic functional changes during evolution, thus OR7D4 is not the special case.

In the case of many mammalian OR orthologs, we show that amino acid changes have dramatic functional consequences on the OR. While the ligand selectivity across OR orthologs is similar, there are changes in the magnitude of response; there are also frequent functional changes in the potency and efficacy of response to a common odor. We find that orthologs respond to a common odor more often than paralogs (82% versus 33%, respectively). Species-specific comparison of orthologs shows human-chimp orthologs responding to a common odor 93% (14/15) of the time, human-macaque orthologs 67% (8/12) of the time and human-mouse orthologs 83% (10/12) of the time, again with differences in potency and efficacy. While sequence comparison predicts human-macaque orthologs to be more similar than human-mouse best-hit pairs, it is interesting that human-mouse orthologs respond to a common ligand more often. While our results raises a possibility of accelerated functional changes in the macaque lineage, further investigation with more ORs and additional macaque species will be necessary. However, this result must be interpreted with caution, as our sample size may not extrapolate to a larger data set and our assignment of human-mouse orthologs is based upon the mutual best-hit assignment in BLAST. The use of best-hit to define our human-mouse orthologs may result in an OR pair representing a one-to-many relationship, in contrast to the more closely characterized one-to-one relationship among primate and rodent orthologs. However, investigating the phylogenetic relationship of our orthologs does reflect overall predicted speciation events, supporting the idea that human-macaque comparisons should be more functionally conserved (Figure S10).

One caveat in our study is that we do not examine within-species variation; we are using only one allele from one animal in a particular species and using these data to represent the complexity of OR response. Though we do not know functional variation within non-human species, a study comparing human alleles of the same OR shows much lower frequency of functional differences (25%) when comparing response to a common ligand (Mainland et al., unpublished), suggesting within species variation would account for minor fraction of functional differences in our analysis. In the future, it would be very interesting to address the within-species allelic variation from primates and rodents to examine how sequence variation within species impacts OR function in comparison with what is seen in the human population.

Aside from ORs, there are several examples where the functions of orthologous genes are not equivalent [5]–[7]. First, the FOXP2 transcription factor that plays a role in speech and language in humans, is highly conserved from mice to humans, differing at only 3 amino acid positions; despite the similarity, the genetic substitution of mouse Foxp2 with the human ortholog results in differences in ultrasonic vocalizations and affects dopamine levels, dendrite morphology, gene expression and synaptic plasticity of medium spiny neurons in the basal ganglia [45]–[47]. Second, a functional comparison of rhodopsin genes from 35 vertebrate species and 11 reconstructed ancestral genes revealed that each rhodopsin receptor has a specific wavelength of maximal absorption that can be related to the environmental changes of an organism's habitat [48]. There are also studies elucidating changes in ligand selectivity of nuclear hormone receptors using ancestral reconstruction [49]–[51], showing changes in ligand preference, sensitivity and general function over time. Human and chimpanzee bitter taste receptors at the TAS2R38 locus show changes in potency to the known ligand PTC (phenylthiocarbamide) when tested in a heterologous system [52], raising the possibility that bitter receptors might also show dynamic functional evolution in closely related species.

One concern is that our *in vitro* system does not mimic the *in vivo* olfactory sensory system and that the expression of primate receptors is systematically misrepresented. Our data suggest that human RTP1S is capable of trafficking ORs from different species and that these receptors can couple to the canonical signaling pathway, sometimes outperforming the human version of the OR. We can also interchange human and mouse versions of RTP1s and do not see a pattern of species-specific RTP-OR interactions (Figure S7). While our set of human ORs tends to be hyperfunctional in comparison to primate orthologs, it does not mean that human ORs as a whole are necessarily more functional. Our selection process for OR orthologs began with previously deorphaned human ORs, thus, we would expect all human receptors to show a response while the chimp and macaque orthologs are variable.

An additional concern is that codon bias between species may alter the expression levels, leading to variability that would not exist in the *in vivo* system. Our data include two human-chimp orthologs (OR5P3 and OR8K3) that differ at the nucleotide level but have identical amino acid sequence. For these pairs of OR orthologs, the response is indistinguishable across 42 ligands and within a single odor (Figure 4C, Figure S6, Figure S9). This suggests that sequence variation at the nucleotide level does not impact the results in our heterologous expression system.

Cell surface expression levels of individual ORs do not appear to dictate the changes in potency of a given receptor in our assay, suggesting the functional changes are an inherent property of the receptor itself (Figure 8, Figure S13). While this assay may not provide the resolution for low-levels of OR expression, our data is consistent with the idea that cell-surface expression level does not correlate with OR function, though a minimum level of cell-surface expression, facilitated by the accessory proteins, is required [25], [26], [34], [42]. It is important to note that for ORs that do not have known ligands, a lack of response to a given odor does not mean these ORs are nonfunctional. Rather, it is possible that these ORs have acquired a new functional activation by different odors not used in our assay. In addition to ligand binding to the ORs, it is plausible that differences in G-protein coupling or receptor recycling do exist between these receptors and should be investigated in the future.

There are several studies that have shown the reliability of this *in vitro* system to predict *in vivo* function and odor perception. Comparison of patch-clamp recordings of olfactory sensory neurons expressing the mouse receptor SR1 to heterologous cells transiently expressing SR1 showed similar patterns of activation to a panel of odors; however, the response from the heterologous system did appear less sensitive than the intact olfactory sensory neurons [53]. In another study, variants of human OR7D4 were shown to respond differently to the ligands androstenone and androstadienone in a heterologous system, and these differences translated to perceptual differences to the odors in the human population [42]. However, the *in vitro* system is not perfect. Our *in vitro* assay lacks many components of an *in vivo* olfactory system, including odorant binding proteins, a mucosal layer, intracellular molecules, and sniffing behaviors. The failure of a specific OR to respond to any of the tested odorants must be interpreted with caution, since it may reflect a failure of the OR to function in our assay rather than a lack of sensitivity to the tested odorant. Taken together, probing the functional differences of OR genes across species using an *in vitro* system is likely to provide useful information in understanding the evolution of the OR family.

Comparisons of high-coverage sequenced genomes show that orthologous relationships of genes between divergent species can be identified for a majority of genes [4], [7]. The idea that the identified function of a gene is upheld for orthologs of that gene across species is a widely accepted assumption for the progress of bioinformatics, as most sequenced genes may never be subjected to functional experimentation; for many examples in closely related species, this idea of equivalent function is upheld [4], [54]–[61]. In the multi-gene family of ORs, it appears that even with a clear 1∶1 evolutionary relationship of orthologs between closely related species, that functional equivalency, in terms of efficacy and potency, is limited. To further understand functional changes of ORs, it will be necessary to test the functional properties of individual mammalian ORs from many species to determine if any ORs orthologs have undergone changes in ligand selectivity.

Hayden et al. (2010) showed that the olfactory subgenome in different species is directly associated with the habitat in which the animal exists [3]. Additionally, a comparison of fruit fly *Drosophila melanogaster* with mosquito *Anopheles gambiae* OR repertoires suggests ecology has shaped the repertoires and that odorants are differentially encoded in a way consistent with ecological niches of each organisms; the two species show different coverage of a chemically defined odor space, tuned to their food-seeking preferences [62]. In the case of rhodopsin orthologs from vertebrate species, the change in functional wavelength adapted to the environment of the organism [48]. While we do not know if these OR functional changes have an impact on the behavior of an individual or species, we can speculate that the OR repertoire in each species has adapted to meet niche- and species-specific demands.

## Methods

### Ortholog identification, cloning, and sequence analysis

Starting with a list of human and mouse ORs with previously identified ligands [27], we identified orthologous genes between human, chimpanzee and rhesus macaque [13] and between mouse and rat [28]. Putative human-mouse OR orthologs were defined as the reciprocal best-hit to the human reference OR with a >80% amino acid identity. Selection of additional human OR subfamily members (paralogs) were based upon receptors already cloned and available in our library. ORs were amplified from genomic DNA (Coriell Cell Repositories) using Phusion polymerase (New England Biolabs) and subcloned into a mammalian expression vector, pCI (Promega), containing the first 20 amino acids of human rhodopsin (Rho-tag). Each receptor was sequenced using a 3100 or 3730 Genetic Analyzer (ABI Biosystems). Analysis of sequence variation was conducted in MATLAB. Evolutionary distance of the nucleotide sequences for each ortholog pair was calculated using the Jukes-Cantor model [30] and the amino acid comparisons were made using Grantham's scale [29]. The 22 amino acid alignment was conducted in Seaview using the ClustalW2 alignment method. The pairwise dN/dS (ω) was determined using the Nei-Gojobori 1986 method, based on the Jukes-Cantor model [63] The additional sequence data for ORs used in Figure S4 originated in Go and Niimura [13], who originally conducted this pairwise analysis using the modified Nei-Gojobori method and additionally assessed selection pressure using branch-test and branch-site test for ORs. Neighbor-Joining trees were built in Seaview.

### Luciferase assay

Dual-Glo Luciferase Assay System (Promega) was used for the luciferase assay as previously described [26]. Rho-tagged ORs (5 ng/well) were transfected into the Hana3A cell line in 95-well plate format (Thermo Scientific) along with the human receptor trafficking protein, RTP1S [25] (5 ng/well), pRL-SV40 (5 ng/well; Promega), CRE-luciferase (10 ng/well; Stratagene) and muscarinic acetylcholine receptor (M3) [24] (2.5 ng/well). Luminescence was measured using a Polarstar Optima plate reader (BMG). First, all luminescence values were divided by the Renilla Luciferase activity to control for transfection efficiency and cell viability in a given well. Normalized luciferase activity was calculated by the formula (L_N_-L_min_)/(L_max_-L_min_), where L_N_ is the luminescence of firefly luciferase in response to the odorant, L_min_ is the minimum luciferase value on a plate or set of plates, and L_max_ is the maximum luciferase value on a plate or set of plates. Data was analyzed using GraphPad Prism 5.0 and MATLAB.

### Screening 42 odors and dose–response curves

42 odorants that quantitatively span chemical space were chosen using a method previously described [27], [33]. Briefly, we generated 20 physicochemical descriptors that predict 62% of the variance in mammalian OR responses [27] for 2683 commonly used odorants. We then divided the 2683 odorants into 42 clusters using k-means clustering. For each cluster, we selected the odorant closest to the centroid of the cluster among odorants that are previously shown to activate at least one OR. If no such ligand was present in the cluster, we selected the odorant closest to the centroid of the cluster to maximize structural diversity. Each orthologous set and a vector control (Rho-pCI) were tested against each odorant at 100 µM (except androstenone, which was applied at 10 µM) and compared to a no odor control; each comparison was performed in triplicate and statistical significance was assessed by a t-test with a correction for multiple comparisons (2-tailed t-test, α = 0.05/42). The human and mouse ORs were used as the reference OR and chimpanzee, rhesus macaque and rat orthologs were the variant ORs. The order of odors is the same within a set of OR orthologs, but is different across ORs. Odors are listed in Table S3.

### Dose–response curves

Dose-response curves were constructed using a single odor at concentrations ranging from 10 nM to 10 mM for the OR-odor pairs for each orthologous set. Each concentration was tested in triplicate and a vector-only control (Rho-pCI) was included for each odorant. The odors for dose responses were chosen before we determined the responses to the comprehensive set of 42 odors, thus, we did not always choose the best ligand for dose response curves, although the chosen ligands always robustly activated at least one of the tested orthologs. We tested all the orthologs against this panel of 42 odors and since we did not find changes in ligand selectivity among orthologs, we did not go back to test the best ligands for dose-responses.

The dose-response data were fit to a sigmoid curve and the resulting data were fit with a 3-parameter logistic model. An odorant was considered an agonist if the 95% confidence intervals of the top and bottom parameters did not overlap, the standard deviation of the fitted log EC50 was less than 1 log unit, and the extra sums-of-squares test confirmed that the odorant activated the receptor significantly more than the vector-only transfected control. For each pair of ORs, we determined if one model fit the data from both ORs better than two separate models using the extra sums-of-squares test. A pair of ORs is classified as hyper/hypofunctional if one OR in the pair had both a higher EC50 (lower efficacy) and a lower potency (dynamic range, or top-bottom). A pair of ORs was undefined if the potency and efficacy showed discordant changes. Dose-response curve images were graphed in GraphPad Prism 5.0 and further analyzed in MATLAB. For dose-response curves, data was baslined and normalized to the maximum response across a set of ORS (Figure 4, Figure 6). In Figure S9, the left column was normalized to the human or mouse OR variant response to show the differences in receptor basline activity and the identical data in the right column was baselined and normalized to the maximum response across the set of receptors. Classification of ORs using the above criteria was coducted in MATLAB.

### Flourescent immunocytochemistry

Hana3A cells were maintained in minimal essetial medium (Sigma) containing 10% fetal bovine serum (Sigma) (M10), 500 µg/ml peniciilin-streptomycin (Invitrogen) and 6 µg/ml amphotericin B (Sigma) [34]. Live-cell surface staining was done as previously described [25], [36], [37]. Briefly, Hana3A cells were seeded on poly-d-lysine coared glass coverslips in 35 mm dishes and transfected with 1000 ng OR, 250 ng RTP1S, and 50 ng of EGFP to control for transfection efficiency. 24-hours post-transfection, primary incubation was carried out at 4°C using mouse monoclonal antibody anti-rhodopsin 4D2 (provided by R. Molday) diluted 1∶100 in M10 containing 15 mM NaN_3_ and 10 mM HEPES (Invitrogen) for 45 min. Cells were washed in Hanks' balanced salt solution containing containing 15 mM NaN_3_ and 10 mM HEPES (Invitrogen), followed by secondary incubation with Cy3-conjugated donkey anti-mouse IgG (Jackson Immunologicals) for 30 min at 4°C, fixed in 1% paraformaldehyde and later mounted in Mowiol. Slides were analzyed on a Zeiss Axioskop2 microscope at 40x oil lens and images were captured using QImaging Retiga 2000R camera and QCapture Pro 6.0 software. For ORs being compared, staining was performed in parallel and pictures were taken with the same exposure time, brightness and contrast. Images were anaylzed in Adobe Photoshop. Cy3 intensity was measured as integrated density (grey value mean X area) and quantifed for background levels and cell-surface expression. Background was subtracted from the cell-surface values and the average and S.E.M. were calculated for each receptor. Cy3 intensity was then compared to the human OR in each OR set using a student's t-test (p<0.05).

## Supporting Information

Figure S1 Relationship of hypothetical OR orthologs and paralogs. Orthologs are defined as genes related via a speciation event (compare OR A from human, chimp and macaque or compare OR B from human, chimp and macaque), while paralogs are genes related via a gene duplication event (compare human OR A to human OR B). hOR is human, cOR is chimpanzee and mOR is rhesus macaque.
(PDF)

Figure S2 Species-specific sequence comparison of OR ortholog sets. *(A)* Nucleotide sequences of ortholog pairs were compared by the Jukes-Cantor method [30]. Identical sequences will have a Jukes-Cantor distance of zero. *(B)* Protein sequences of ortholog pairs were compared using Grantham's amino acid property scale [29]. Sequences with highly similar amino acid substitutions will have a Grantham distance closer to zero.
(PDF)

Figure S3 Analysis of 22 amino acid positions in orthologs predicted to be involved in ligand binding. *(A)* Alignment of corresponding 22 amino acids predicted to be involved in ligand binding [17] from our primate and rodent OR orthologs. Amino acid color categories: KR, red; AFILMVW, blue; NQST, green; HY, teal; C, salmon; DE, purple; P, yellow; G, orange. *(B)* Amino acid similarity of the 22 amino acids using Grantham's distance plotted against functional distance, as defined by the correlation (1-R, Table S2) of the response across the 42-odor panel for ORs responding to more than three odors (r_s_ = 0.32, p = .04, Spearman's correlation; compare to Grantham's amino acid similarity for full length OR sequences (Figure 3B)).
(PDF)

Figure S4 Distribution of ω (dN/dS) values for putatively functional orthologous pairs of ORs. 259 1∶1 putatively functional orthologs from human-chimp (orange) and 152 1∶1 human-macaque functional orthologs (blue) from Go and Niimura (GN2008) (26) are plotted with our OR ortholog sets (darker shades). Dotted line ω = 1.0.
(PDF)

Figure S5 Defining odor space. We calculated 20 chemical descriptors previously shown to explain more than 62% of the variance in functional responses in a heterologous system for 2683 odorants [27]. For display purposes, the odorants are projected onto a 2D space made of the first and second principal components. Black crosses represent all 2683 odorants, orange circles represent the 42 odorants chosen to span olfactory space and used in our tuning curve data (Figure 2, Figure S6). Odors are listed in Table S3.
(PDF)

Figure S6 Sensitivity-ordered tuning curves for all OR ortholog sets. 42 odors are displayed along the x-axis according to the response elicited from the human OR for primate sets and mouse OR for rodent pairs, with the best response in the center of the distribution. The order of the odors is the same between orthologs in a set, but different across receptors. The y-axis represents the luciferase response to an odor at 100 µM (n = 3, ± S.E.). Negative values on the y-axis indicate the odor elicited an inhibitory response on OR signaling. If a given odorant did not signifcantly activate any of the ORs above the no-odor control (2-tailed t-test, α = 0.05/42), the response was set to zero. Odors are listed in Table S3.
(PDF)

Figure S7 Receptor transport protein, RTP1S, does not show a species-specific interaction. Four mouse and four human ORs were tested against a known ligand at 100 µM in a luciferase assay using either human RTP1S (grey) or mouse RTP1S (black). Y-axis denotes response (n = 6, ± S.E). Mouse RTP1S outperformed human RTP1S in most cases (F(1,80) = 11.44, p = 0.0011, 2-way ANOVA), but this response was not species-specific (F(7,80) = 1.03, p = 0.416, 2-way ANOVA). OR/ligand pairs were as follows: MOR33-1 to octanoic acid, MOR170-1 to coumarin, MOR204-6 to coumarin, MOR277-1 to (+)-camphor; OR2W1 to allyl phenyl acetate, OR10G3 to ethyl vanillin, OR5P3 to coumarin, OR8K3 to (+)-menthol.
(PDF)

Figure S8Hypothetical dose-response curve explaining the classification of functional changes. Using both the potency (EC50, a) and efficacy (dynamic range, b) to a particular ligand, an OR pair is classified as either indistinguishable (compare OR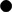 to OR ▴), hyper/hypo functional (one OR had both a lower potency *and* efficacy, compare OR ▪ to OR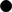/▴) or undefined (orthologs were significantly different *but* potency and efficacy did not change concordantly, compare OR ▪ to OR ▾).(PDF)

Figure S9 Dose-response curves for all OR ortholog sets. X-axis is the concentration of a given odor in Log Molar. Y-axis is normalized response (n = 3, ± S.E.M.). Human (h), chimpanzee (c) and rhesus macaque (m) in primate ortholog sets; mouse (m) and rat (r) for rodent ortholog sets. Vector control is Rho-pCI. Data in the left column are normalized to the human OR response. Identical data in the right column are baselined and normalized to the maximum response across a set of receptors for easier visual comparison.
(PDF)

Figure S10 Phylogenetic relationship of OR orthologs and paralogs. A phylogenetic tree for each OR set was constructed using the Neighbor-Joining method in Seaview. Bootstrap values of 100 replicates are included for each branch. For all OR sets, human-chimpanzee-rhesus macaque orthologs are most closely related to each other, while mouse best-hit ORs (presumably human-mouse orthologs) and human paralogs have unique relationships among each OR set. Human reference OR is in black text, ORs that responded to a common a ligand are in green and ORs that did not respond to a common ligand are in red.
(PDF)

Figure S11 Dose-response curves for all OR orthologs and paralogs. X-axis is the concentration of a given odor in Log Molar. Y-axis is normalized response (n = 3, ± S.E.M.). Human (h), chimpanzee (c) and rhesus macaque (m), mouse receptors (m+number); *para* indicates a receptor that is a paralog to the human reference OR. Vector control is Rho-pCI. Data are normalized to the human OR response.
(PDF)

Figure S12 Alignment of 22 amino acid positions in orthologs and paralogs predicted to be involved in ligand binding. Alignment of corresponding 22 amino acids [17] from our orthologs and paralogs. Amino acid color categories: KR, red; AFILMVW, blue; NQST, green; HY, teal; C, salmon; DE, purple; P, yellow; G, orange. See Table S6 for corresponding Grantham's distance.
(PDF)

Figure S13 Live cell-surface staining for orthologs and subfamily members. Each panel contains a quantification of live cell-surface expression of each receptor. * p<0.05,** p<0.01, ***p<0.001 (student's t-test) when compared to primary human OR in each set. Y-axis denotes the average Cy3 intensity in arbitrary units (a.u.) (n = 3, ± S.E.M.) with S6 as positive control and Rho-pCI as negative control. Representative images of live cell-surface staining for each receptor. Cy3 intensity is plotted against the EC50 of each receptor to a common ligand (and analyzed using Spearman's correlation; see Table S5, Table S7). If receptor did not respond, EC50 was plotted at -3 Log Molar.
(PDF)

Table S1 Receptor Sequences. FASTA file of the amino acid sequences of the ORs used in all functional experiments.
(PDF)

Table S2 Comparison of OR orthologs. For each pair of orthologs, the Jukes-Cantor distance (38), Grantham distance (37) of open reading frame (ORF) and 22 amino acids from Man et al. (2004) [17] (22AA), ω (dN/dS) and correlation values (R and p) for tuning curve responses are listed.
(PDF)

Table S3 Odors used in the study. Odors are listed by their common name, Chemical Abstract Service registry number (CAS#), and corresponding abbreviation used in the tuning curve data (Figure 2, Figure S6).
(PDF)

Table S4 Comparison of dose-response curves from orthologous sets. LogEC50 (M), Span (dynamic-range) for each OR is given. DNR, does not respond. F-ratio and p-values from extra sum-of-squares test.
(PDF)

Table S5 Comparison of dose-response curves from orthologs and paralogs. LogEC50 (M), Span (dynamic-range) for each OR is given. DNR, does not respond. F-ratio and p-values from extra sum-of-squares test.
(PDF)

Table S6 Grantham's distance for orthologs and paralogs. The Grantham's distance was calculated for OR open reading frame (ORF) and for 22 amino acids (22AA) used in Man et al. (2004) [17]. Each ortholog and paralog is compared to the reference human OR in that group (listed first). The distribution of orthologs to paralogs using ORF and 22A was significantly different (z = −6.61, p<0.0001 ORF; z = −7.35, p<0.0001 22AA, Wilcoxon Rank Sum), as was the distribution of paralogs that did or did not respond using 22AA (z = −3.54, p<0.0004, Wilcoxon Rank Sum) (Figure 7).
(PDF)

Table S7 Live cell-surface expression of individual receptors. For each receptor in a group, the average Cy3 intensity in arbitrary units (a.u.) (n = 3, ± S.E.M.) and p-value to the main human OR in the group is given. S6 is positive control and Rho-pCI is negative control.
(PDF)
