# Supplementary figures and images for: Functional Evolution of Mammalian Odorant Receptors
Source: PLoS Genet. 2012 Jul 12;8(7):e1002821. doi: 10.1371/journal.pgen.1002821 (PMC3395614; doi:10.1371/journal.pgen.1002821)

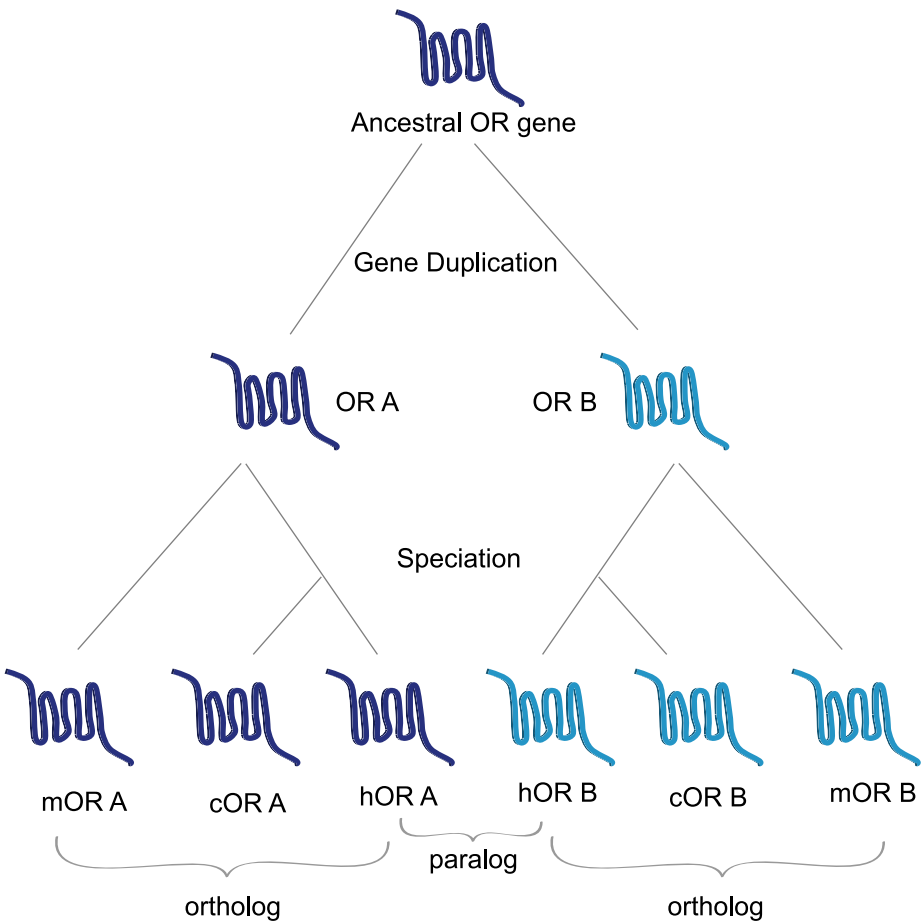

Supplement: Figure S1 — Relationship of hypothetical OR orthologs and paralogs. Orthologs are defined as genes related via a speciation event (compare OR A from human, chimp and macaque or compare OR B from human, chimp and macaque), while paralogs are genes related via a gene duplication event (compare human OR A to human OR B). hOR is human, cOR is chimpanzee and mOR is rhesus macaque. (PDF) [file pgen.1002821.s001.pdf]

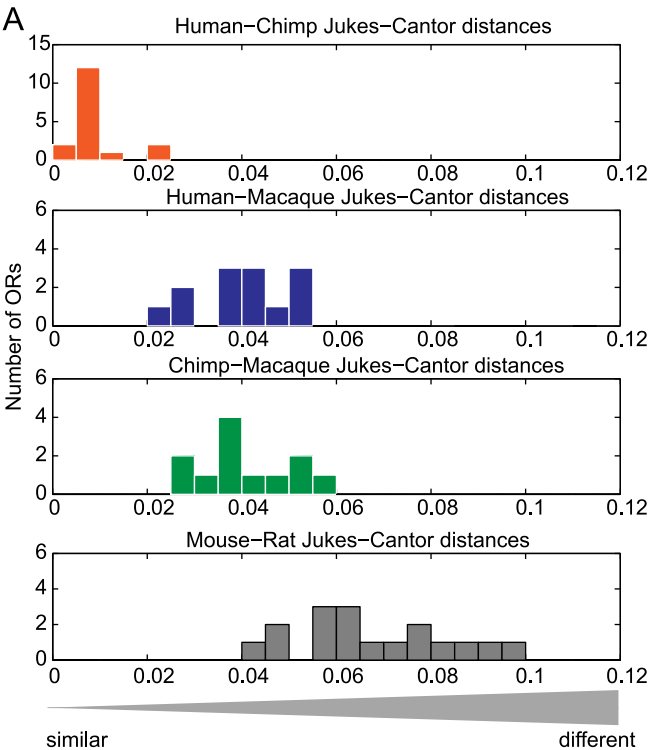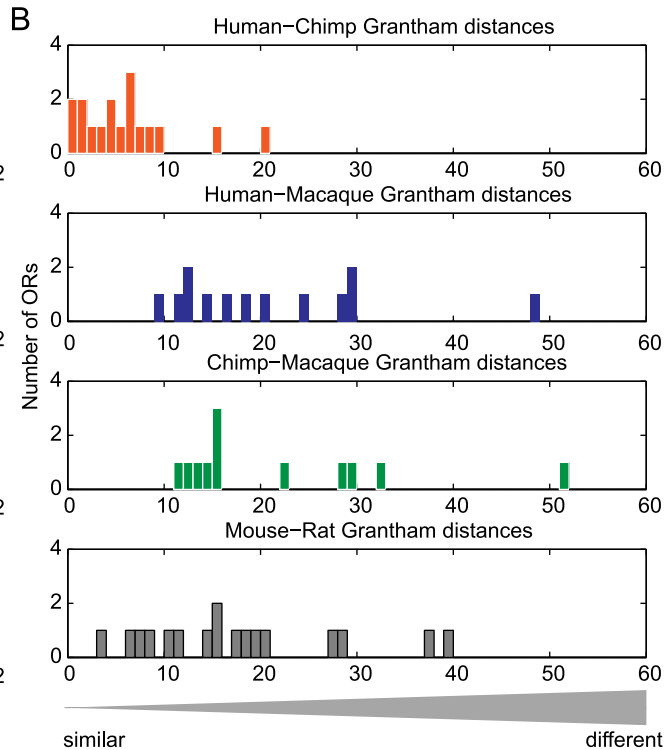

Supplement: Figure S2 — Species-specific sequence comparison of OR ortholog sets. (A) Nucleotide sequences of ortholog pairs were compared by the Jukes-Cantor method [30]. Identical sequences will have a Jukes-Cantor distance of zero. (B) Protein sequences of ortholog pairs were compared using Grantham's amino acid property scale [29]. Sequences with highly similar amino acid substitutions will have a Grantham distance closer to zero. (PDF) [file pgen.1002821.s002.pdf]

Frequency (Number of ORs)

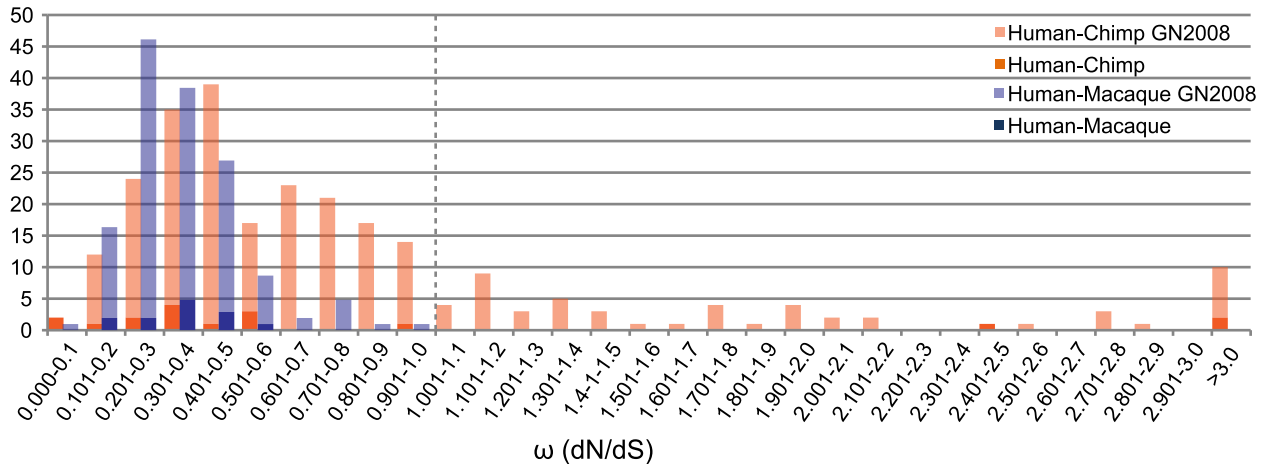

Supplement: Figure S4 — Distribution of ω (dN/dS) values for putatively functional orthologous pairs of ORs. 259 1∶1 putatively functional orthologs from human-chimp (orange) and 152 1∶1 human-macaque functional orthologs (blue) from Go and Niimura (GN2008) (26) are plotted with our OR ortholog sets (darker shades). Dotted line ω = 1.0. (PDF) [file pgen.1002821.s004.pdf]

# Odor Space

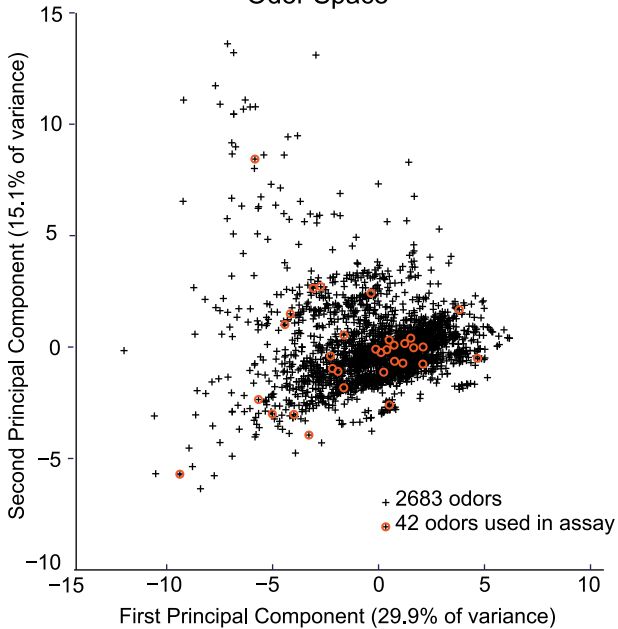

Supplement: Figure S5 — Defining odor space. We calculated 20 chemical descriptors previously shown to explain more than 62% of the variance in functional responses in a heterologous system for 2683 odorants [27]. For display purposes, the odorants are projected onto a 2D space made of the first and second principal components. Black crosses represent all 2683 odorants, orange circles represent the 42 odorants chosen to span olfactory space and used in our tuning curve data (Figure 2, Figure S6). Odors are listed in Table S3. (PDF) [file pgen.1002821.s005.pdf]

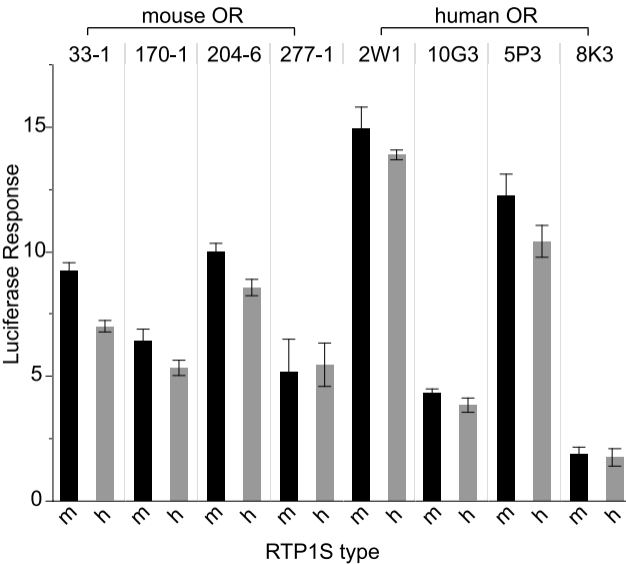

Supplement: Figure S7 — Receptor transport protein, RTP1S, does not show a species-specific interaction. Four mouse and four human ORs were tested against a known ligand at 100 µM in a luciferase assay using either human RTP1S (grey) or mouse RTP1S (black). Y-axis denotes response (n = 6, ± S.E). Mouse RTP1S outperformed human RTP1S in most cases (F(1,80) = 11.44, p = 0.0011, 2-way ANOVA), but this response was not species-specific (F(7,80) = 1.03, p = 0.416, 2-way ANOVA). OR/ligand pairs were as follows: MOR33-1 to octanoic acid, MOR170-1 to coumarin, MOR204-6 to coumarin, MOR277-1 to (+)-camphor; OR2W1 to allyl phenyl acetate, OR10G3 to ethyl vanillin, OR5P3 to coumarin, OR8K3 to (+)-menthol. (PDF) [file pgen.1002821.s007.pdf]

# Hypothetical Dose-Response

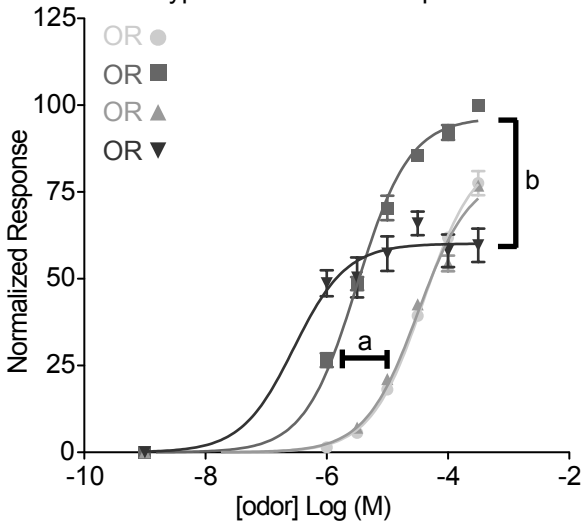

Supplement: Figure S8 — Hypothetical dose-response curve explaining the classification of functional changes. Using both the potency (EC50, a) and efficacy (dynamic range, b) to a particular ligand, an OR pair is classified as either indistinguishable (compare OR to OR ▴), hyper/hypo functional (one OR had both a lower potency and efficacy, compare OR ▪ to OR/▴) or undefined (orthologs were significantly different but potency and efficacy did not change concordantly, compare OR ▪ to OR ▾). (PDF) [file pgen.1002821.s008.pdf]

Human-Chimpanzee-Macaque (H,C,M) OR orthologs

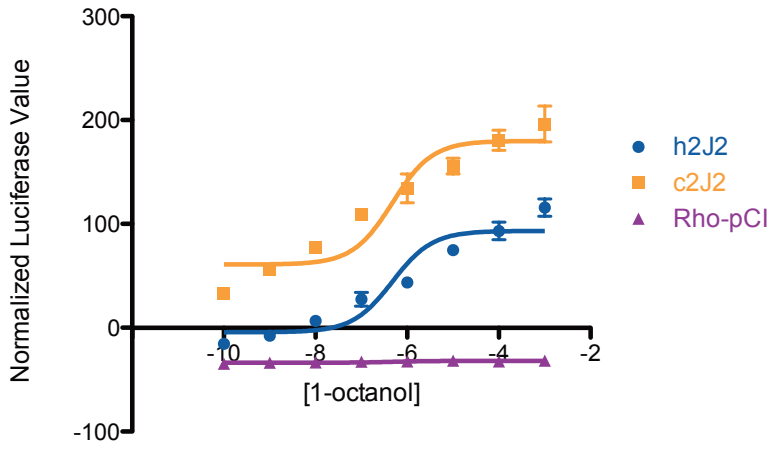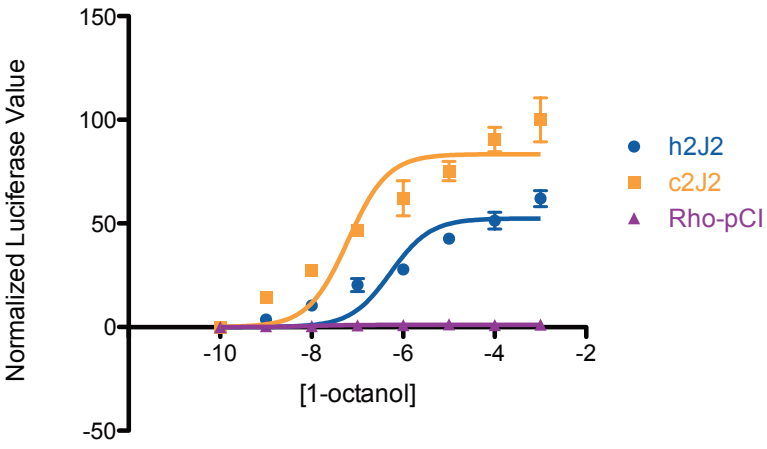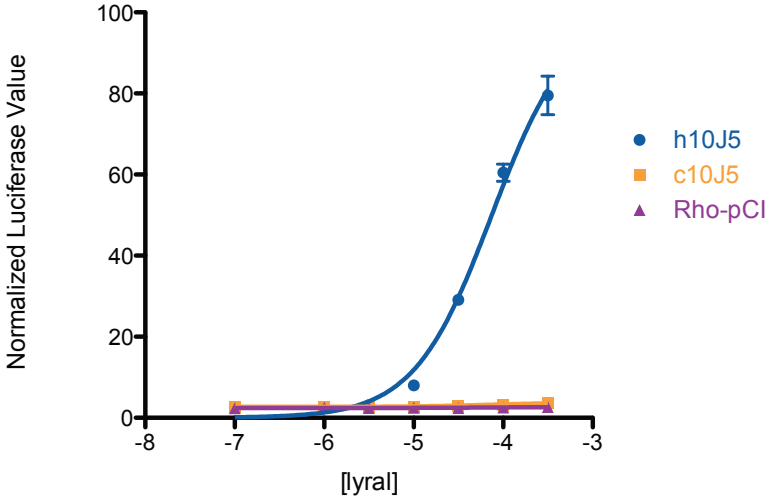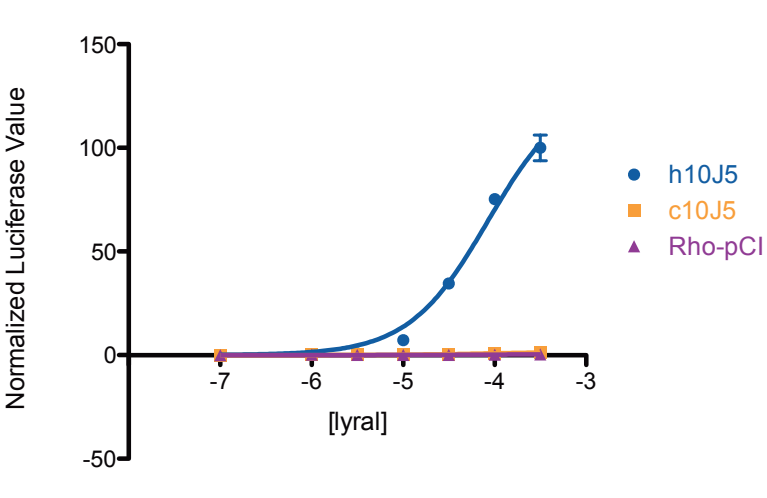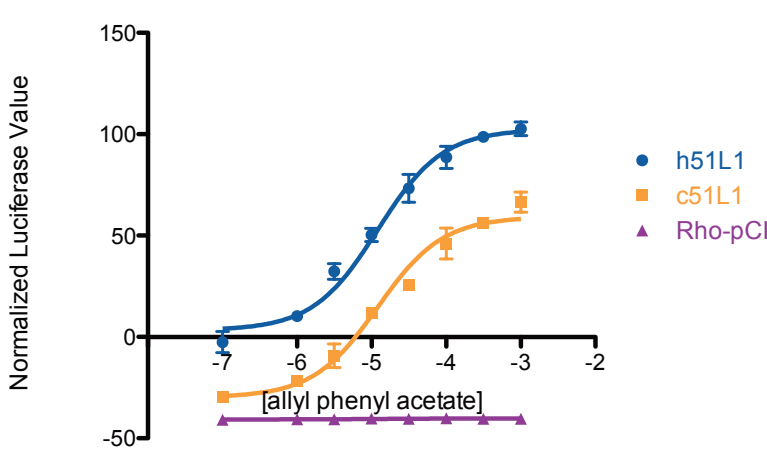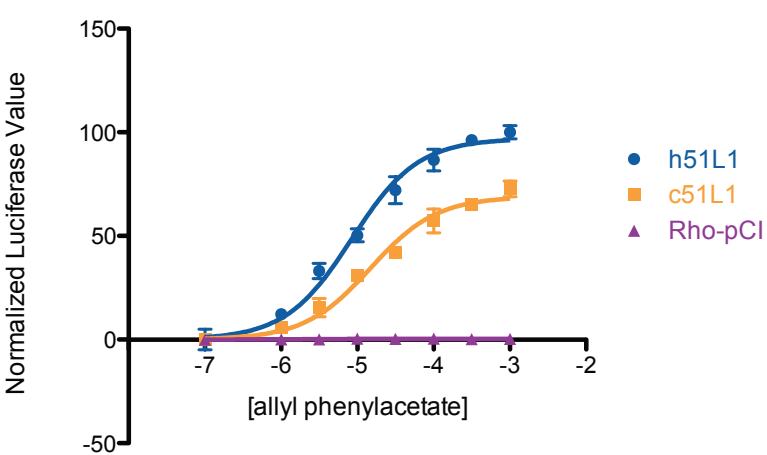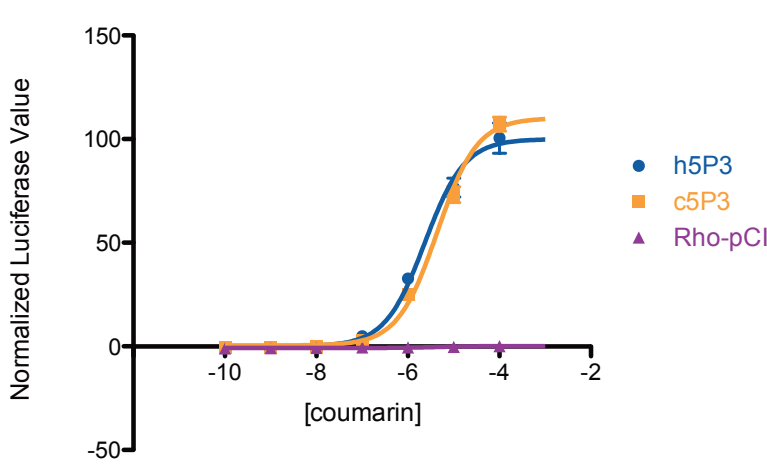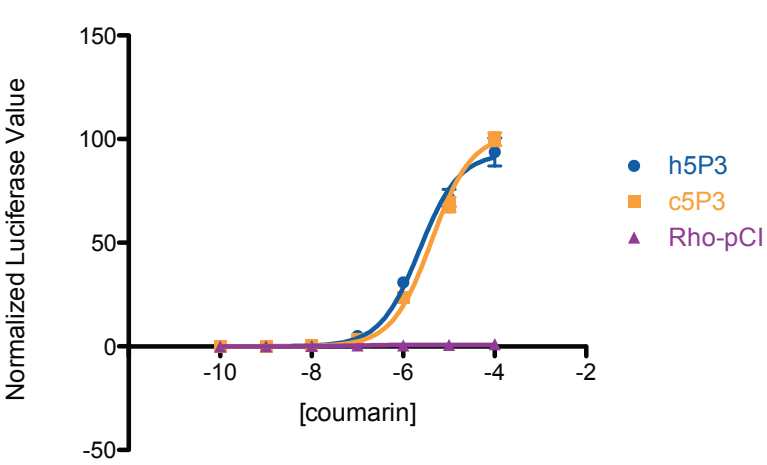

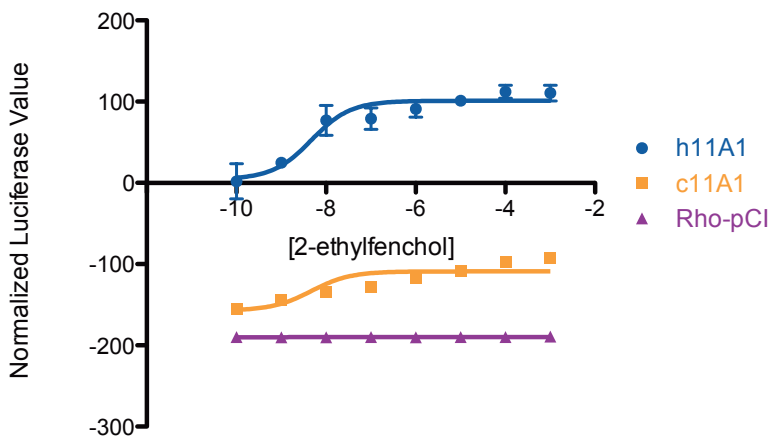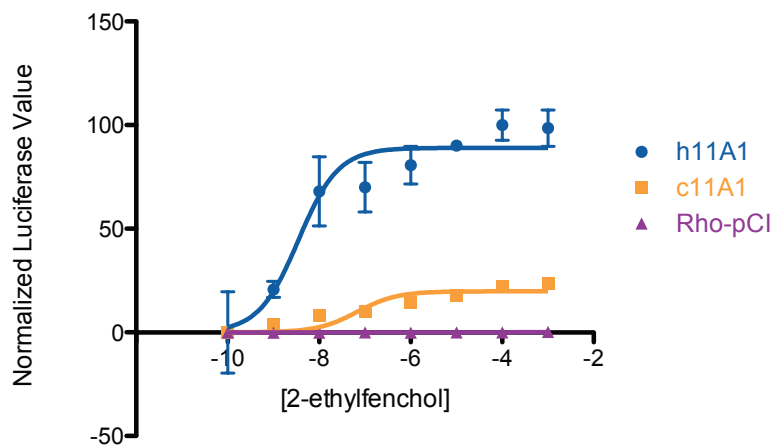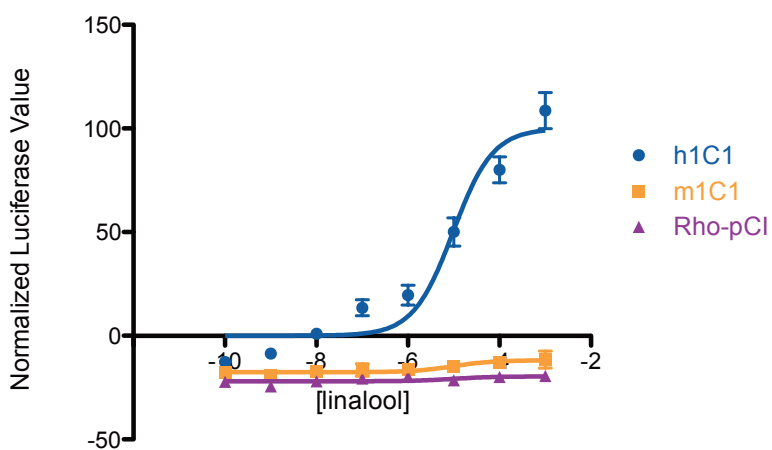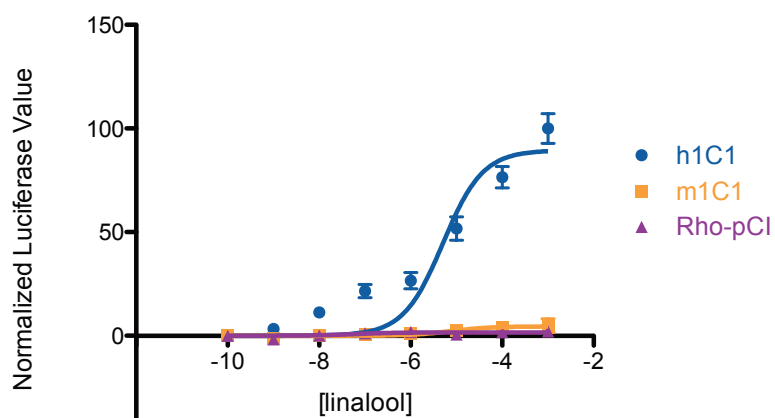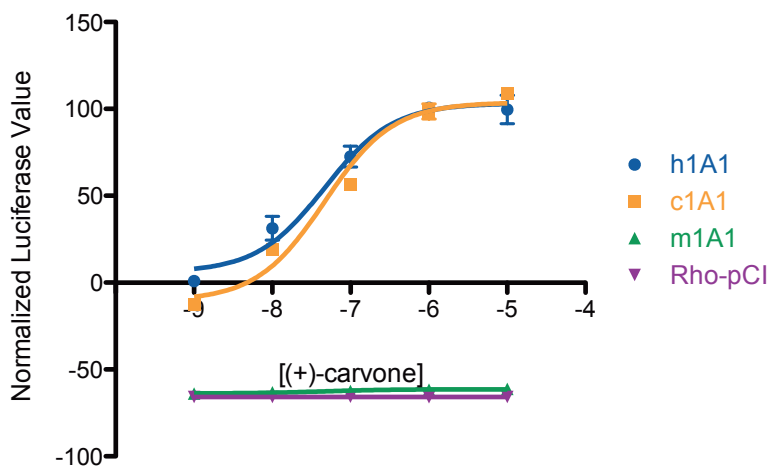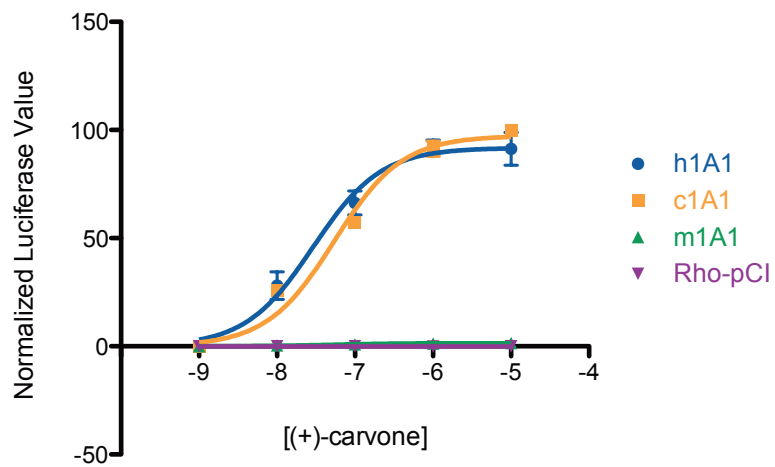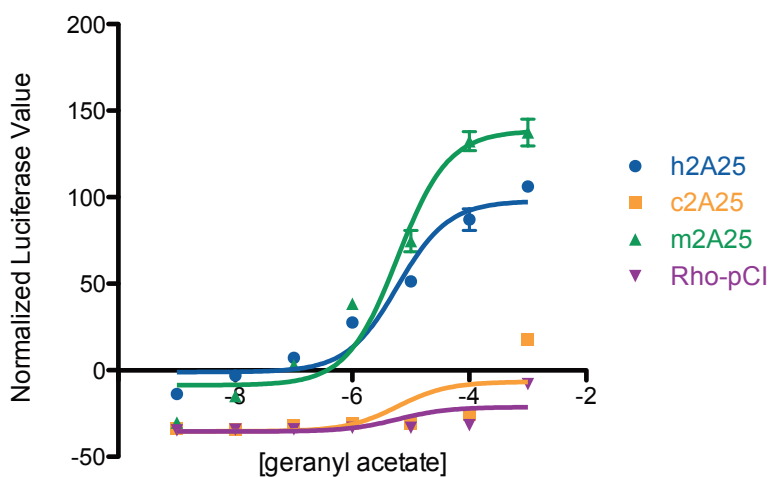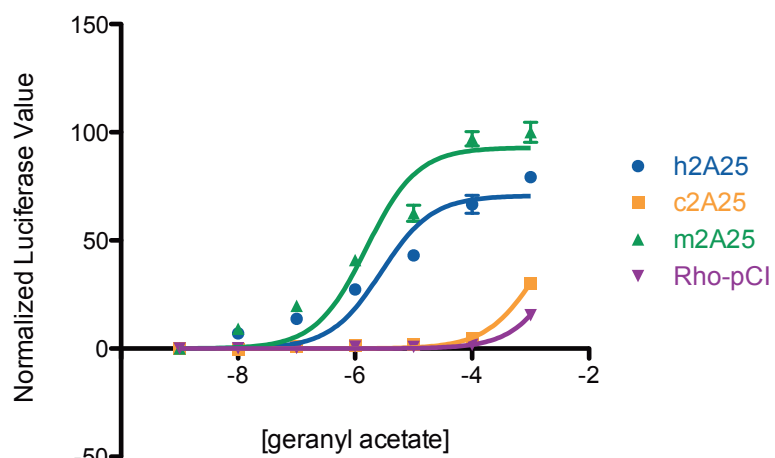

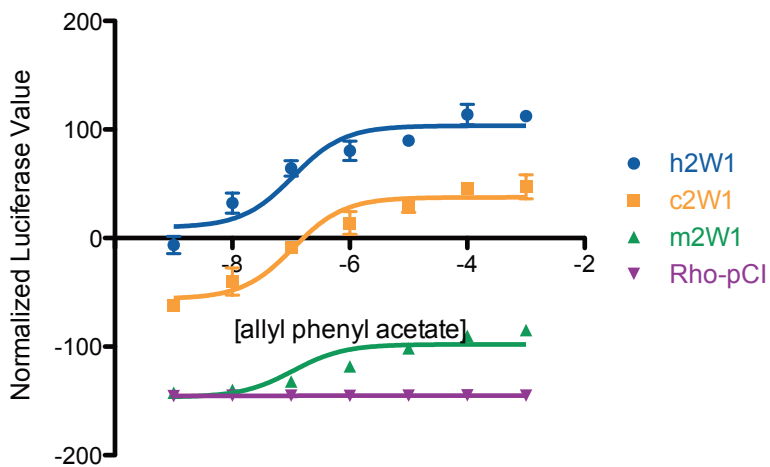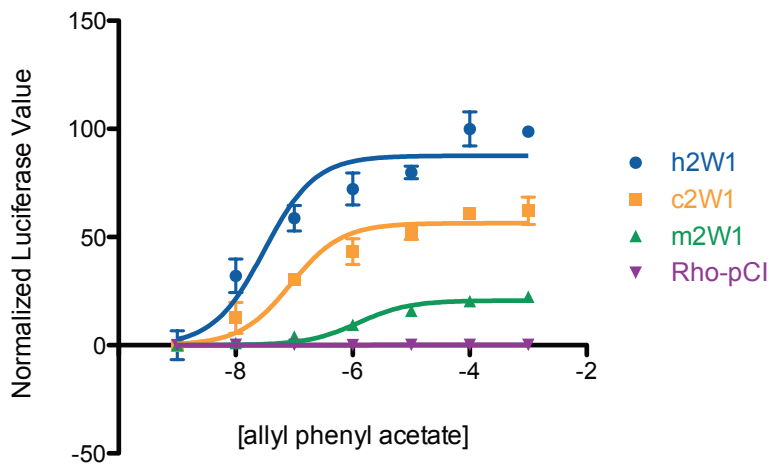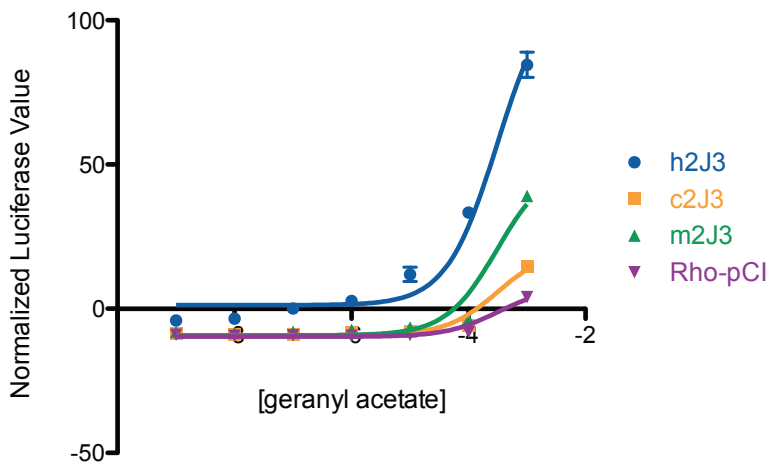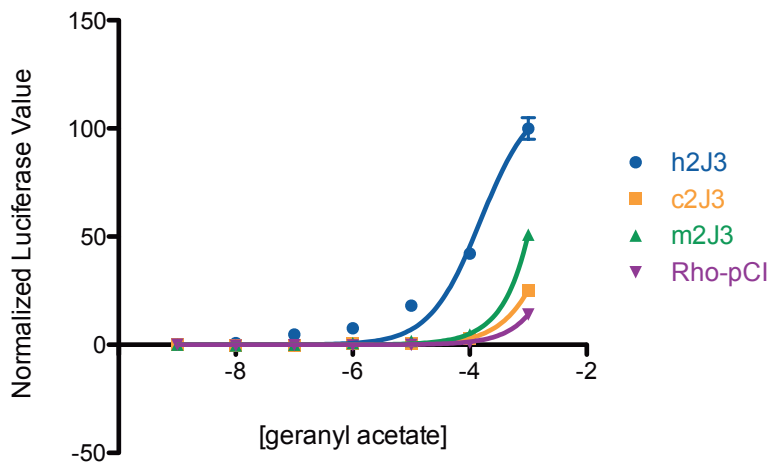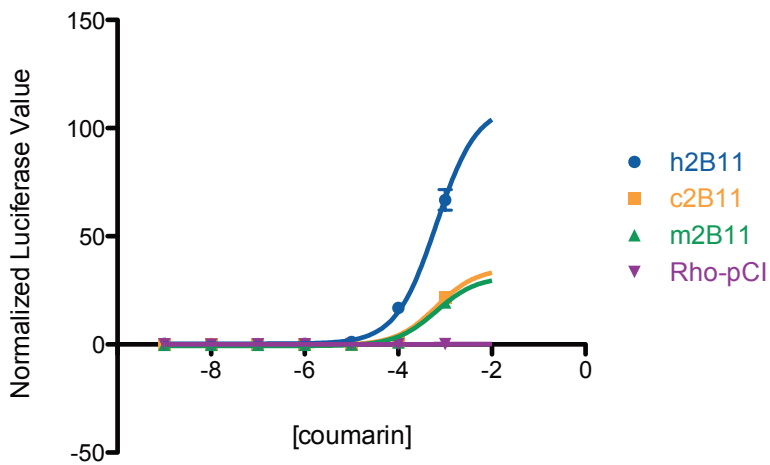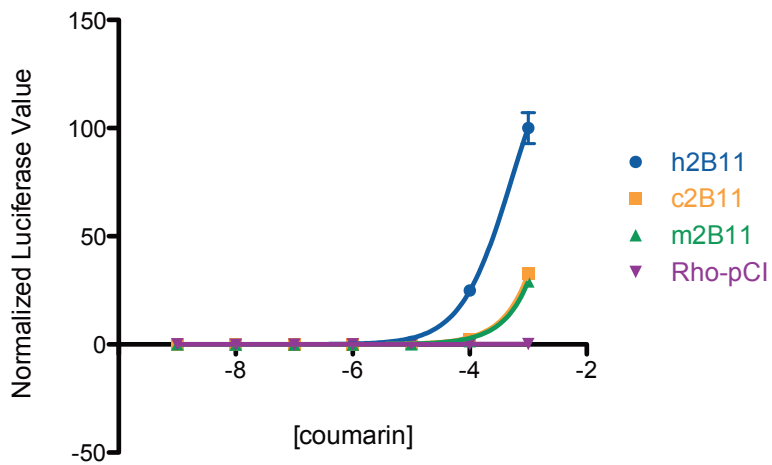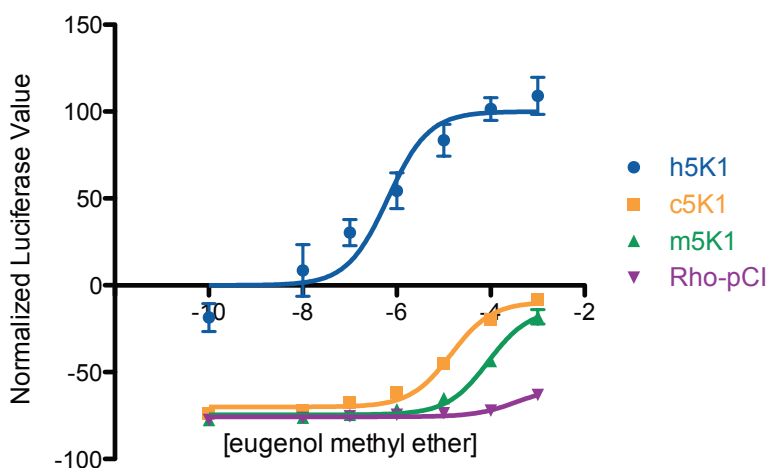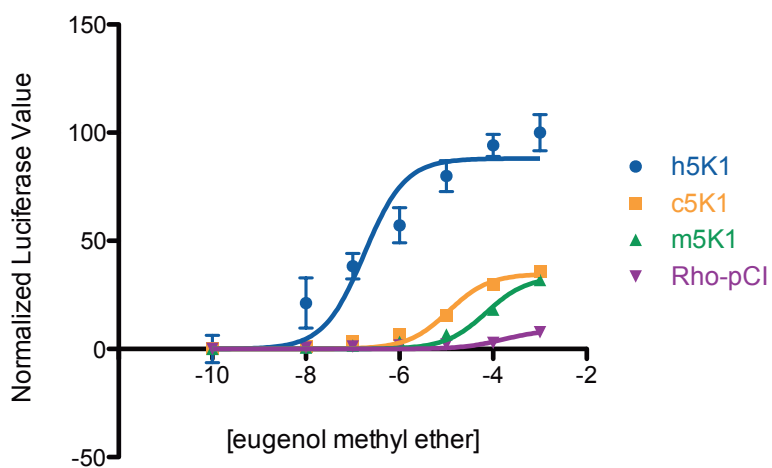

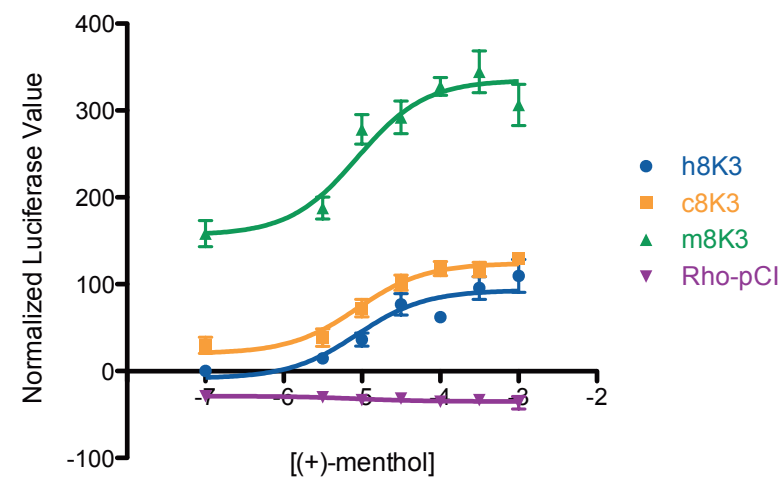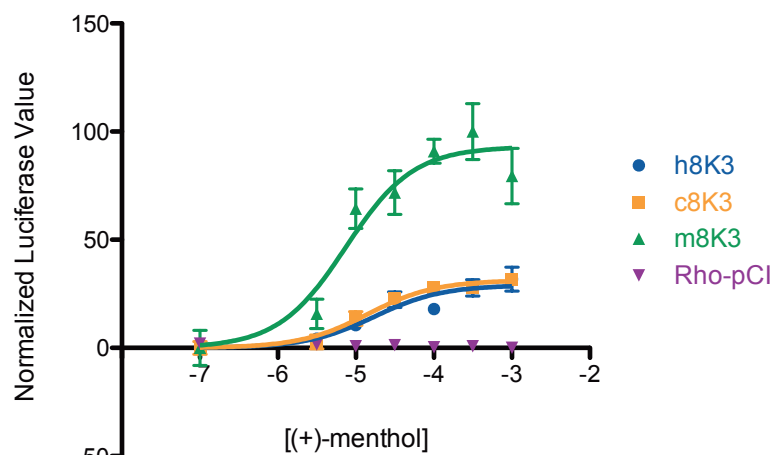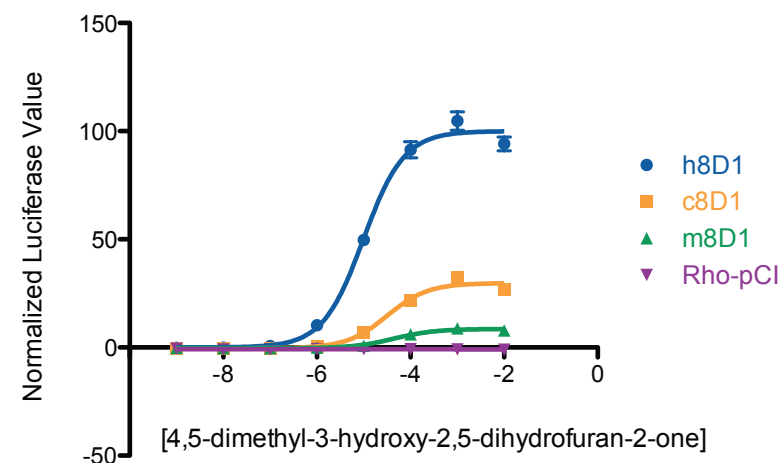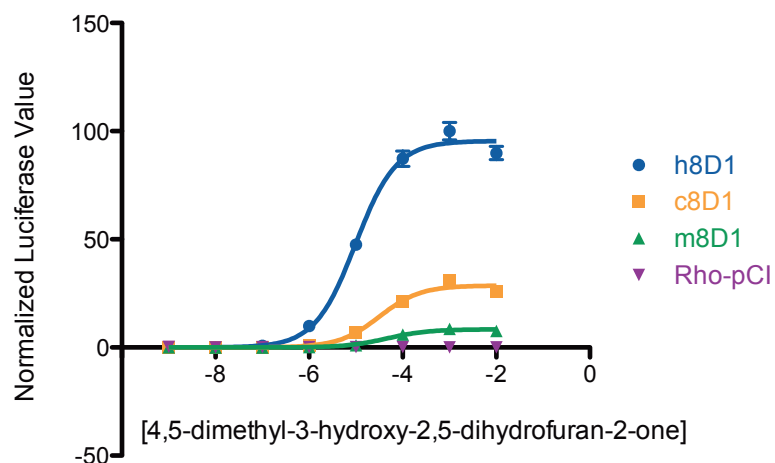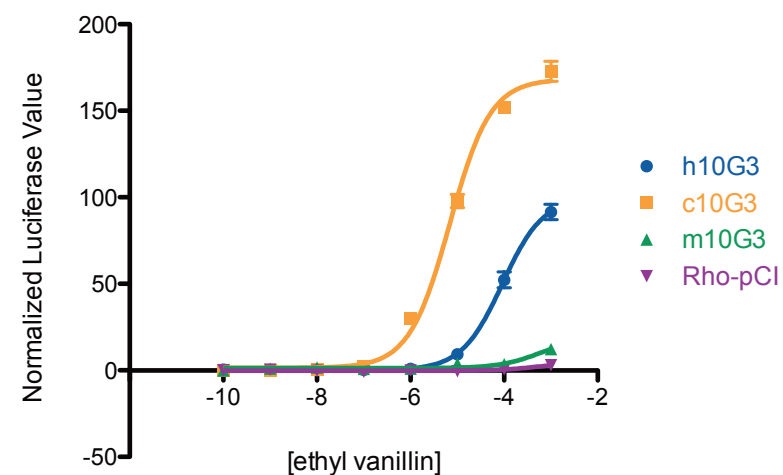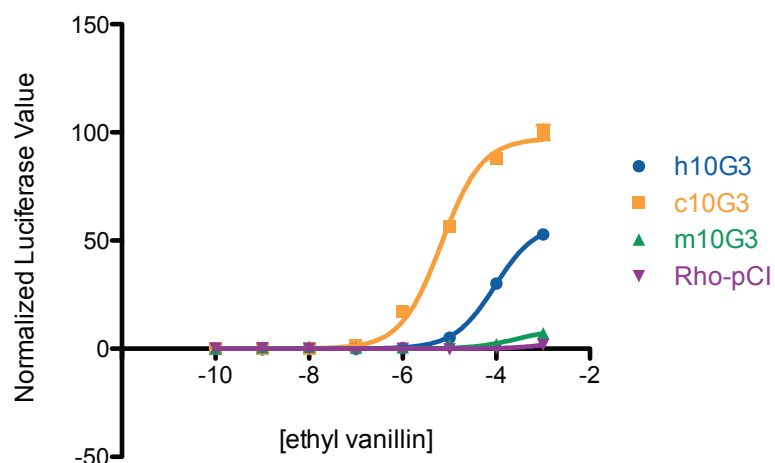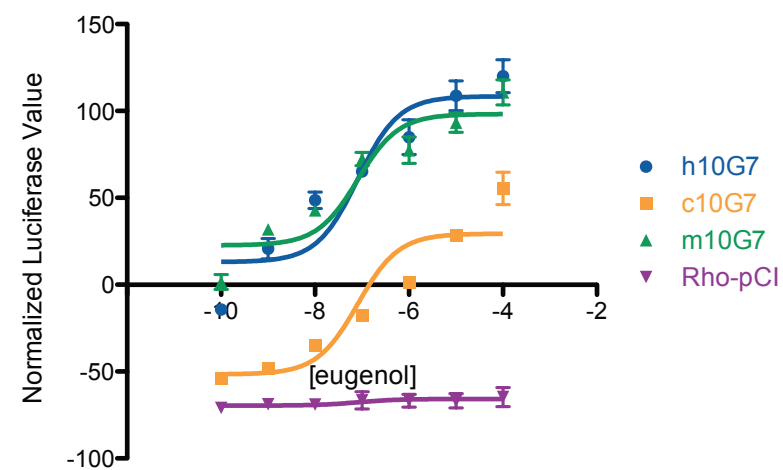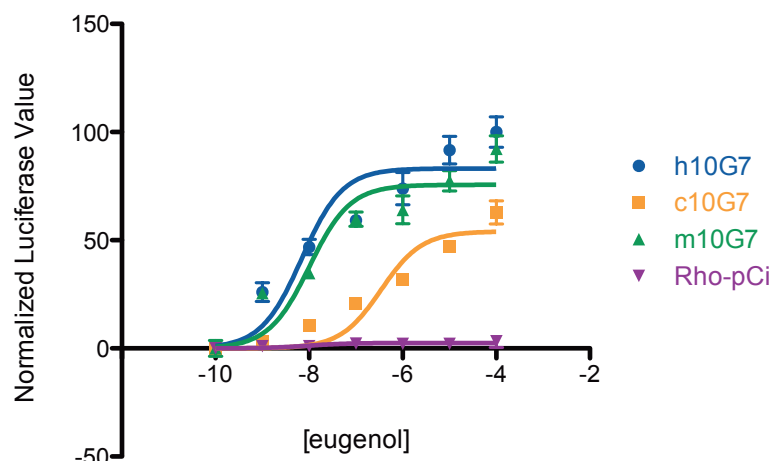

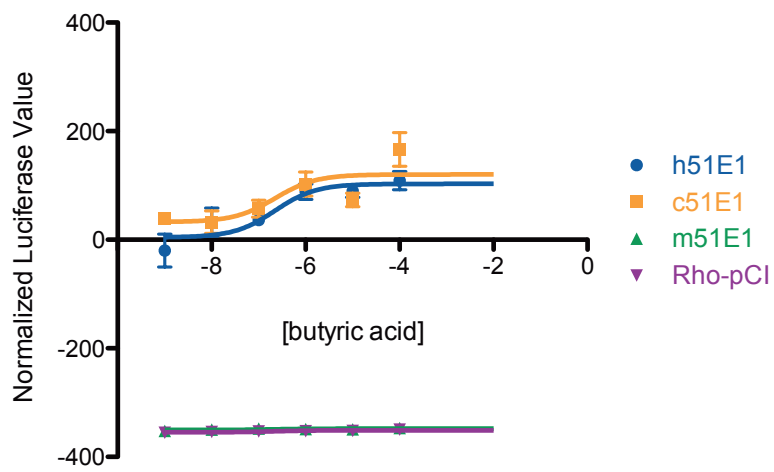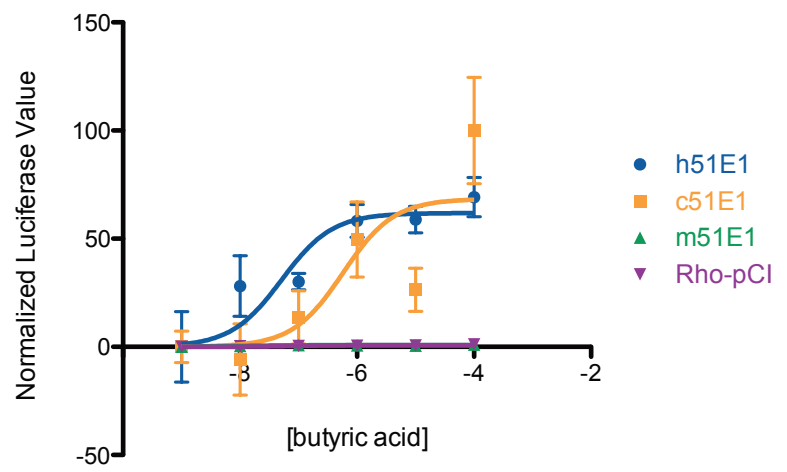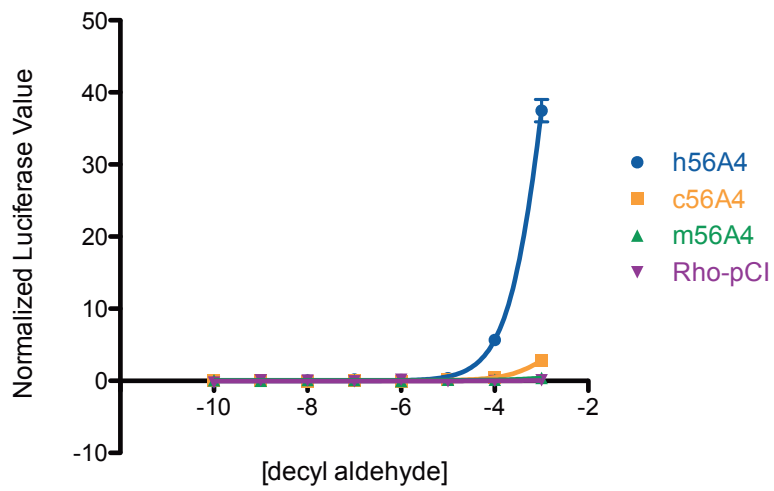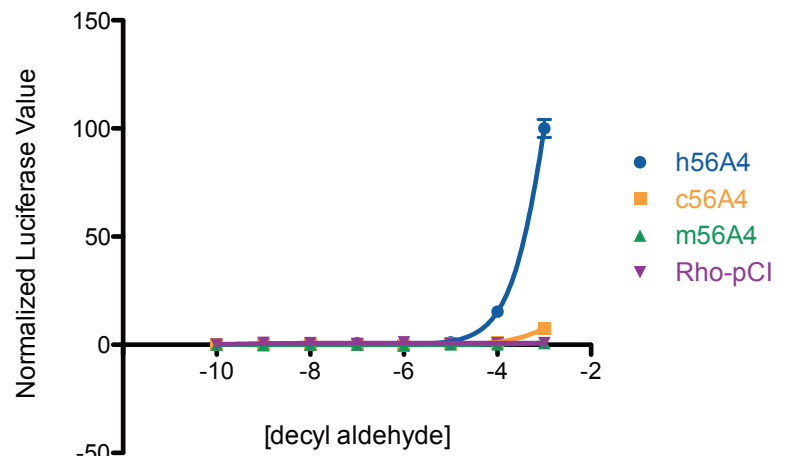

## Mouse-Rat (M,R) OR orthologs

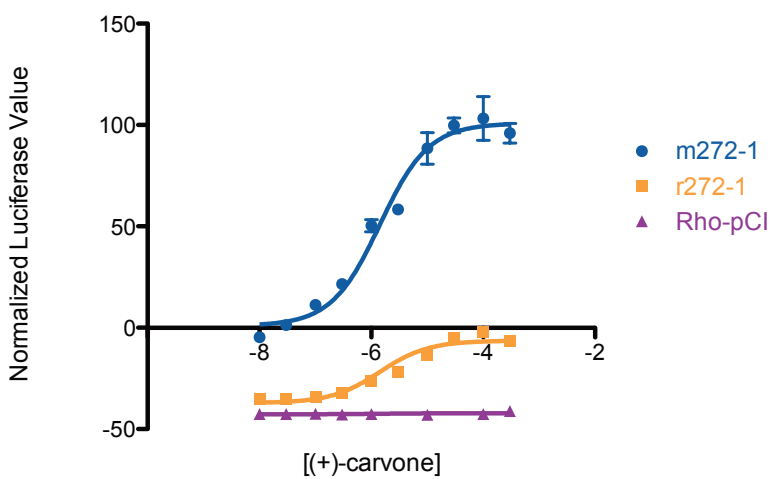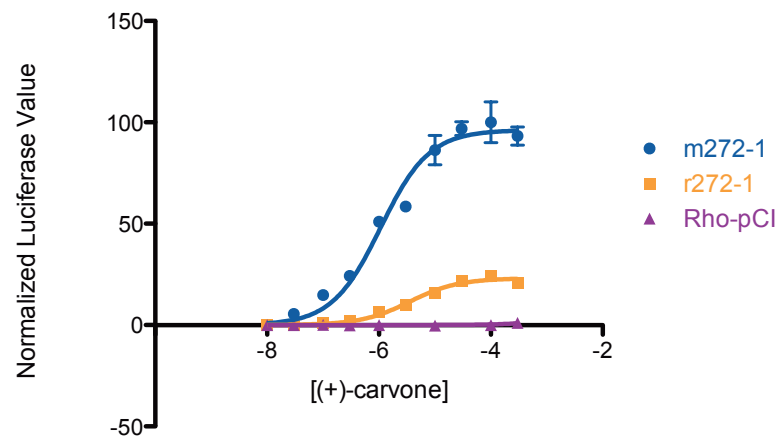

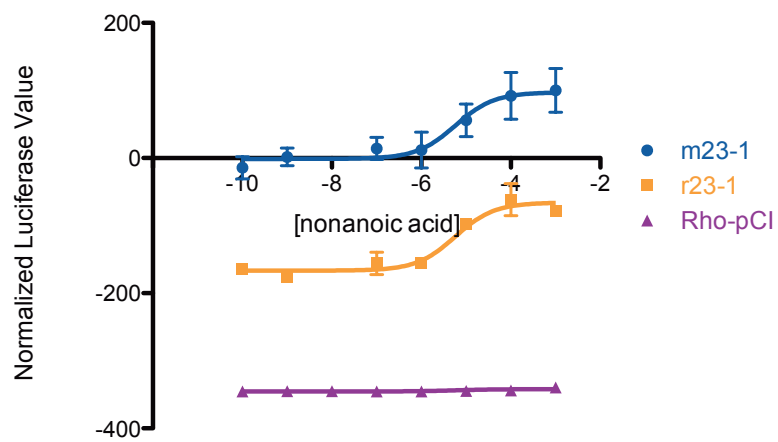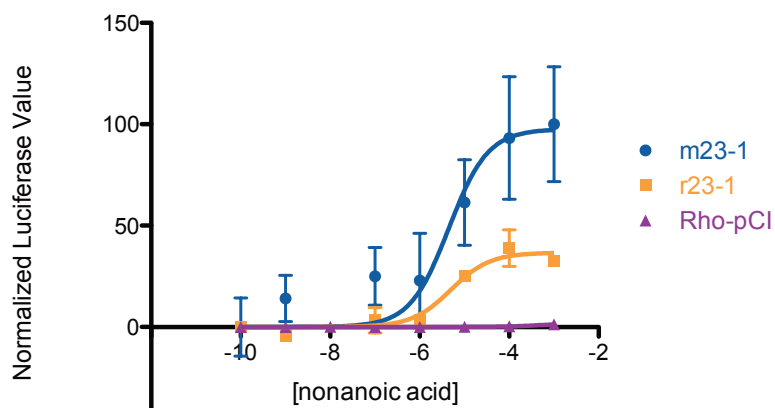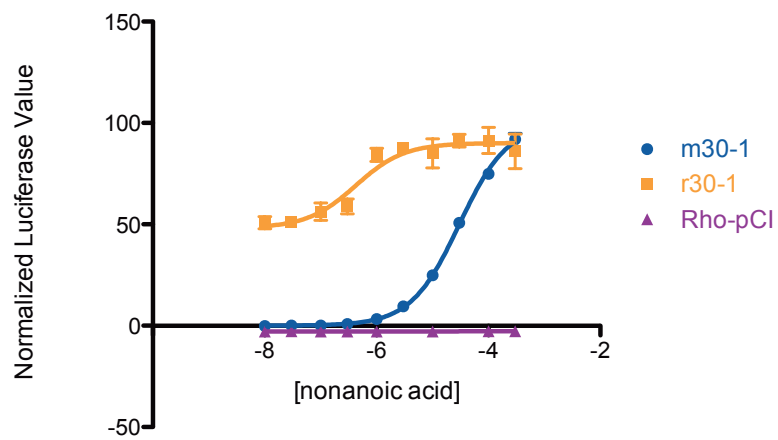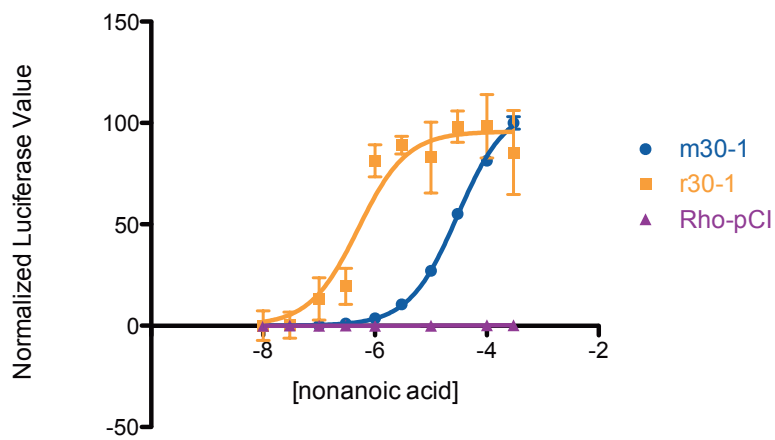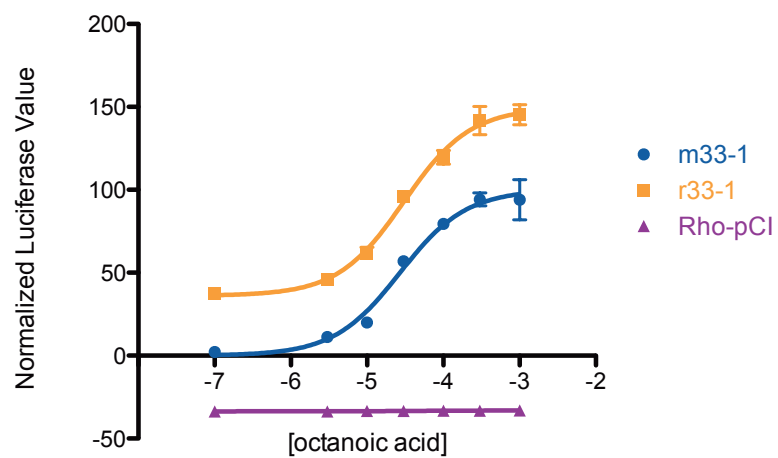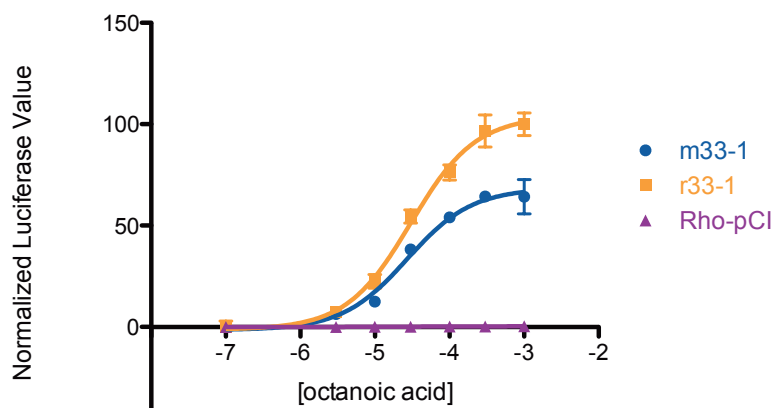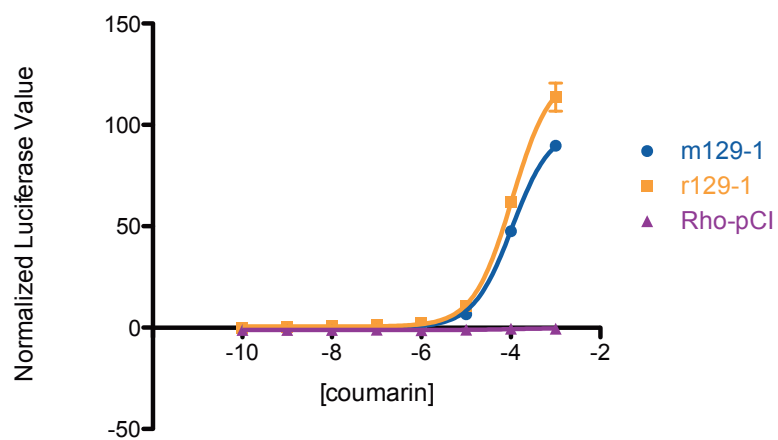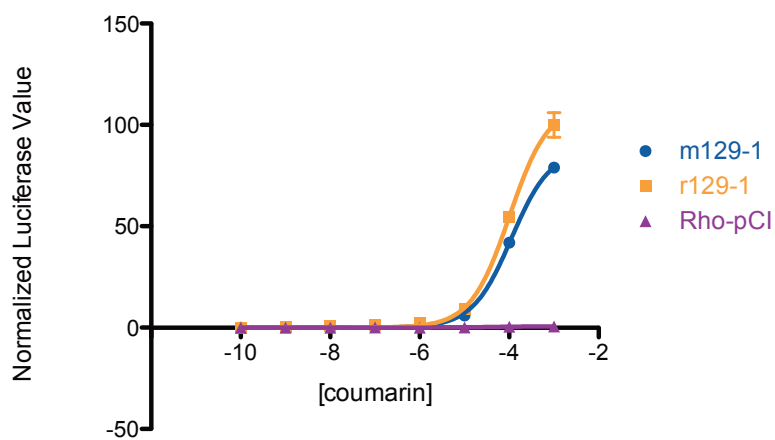

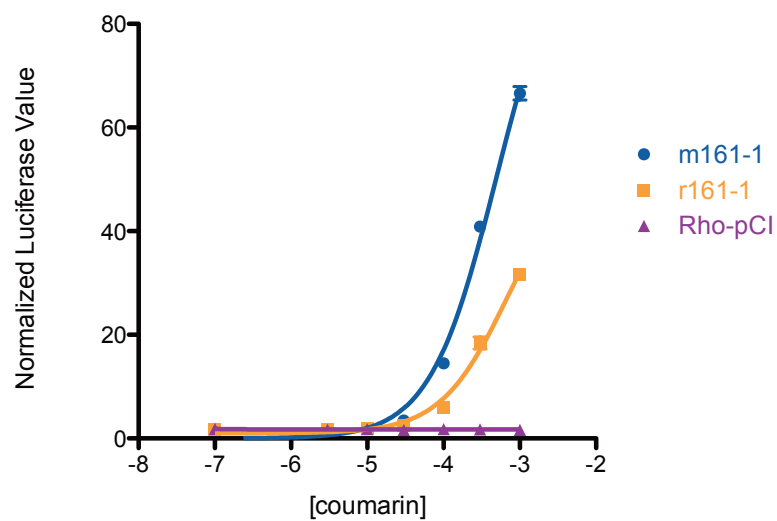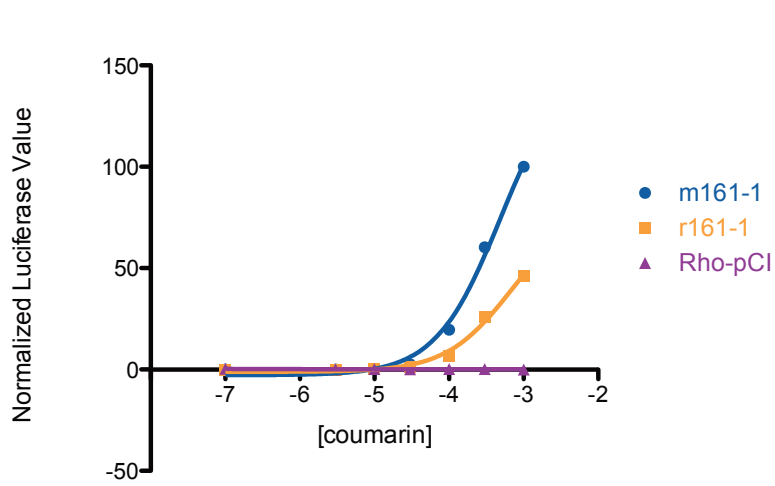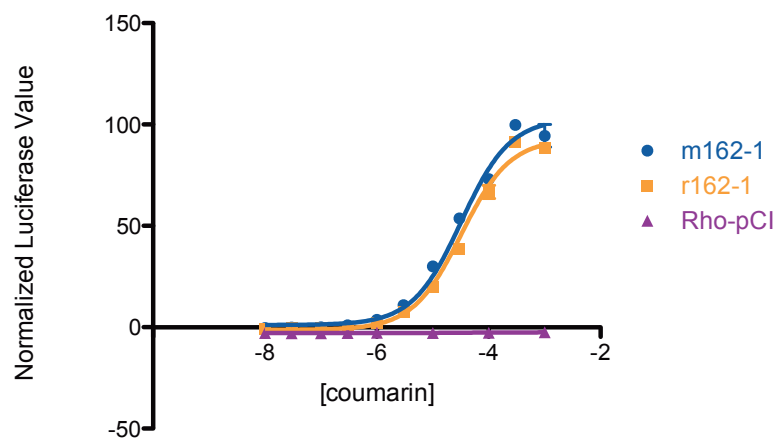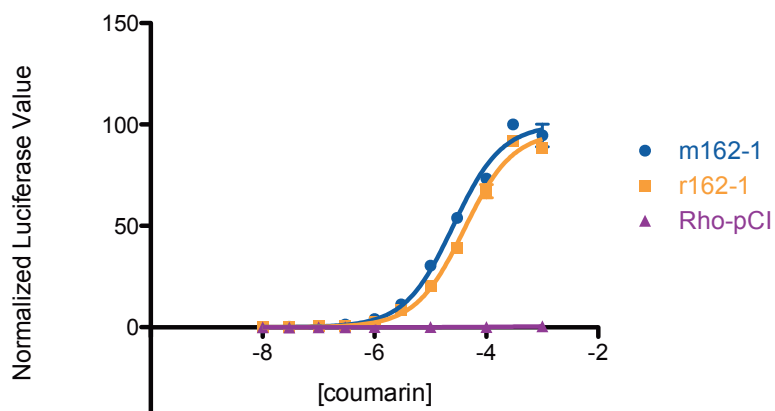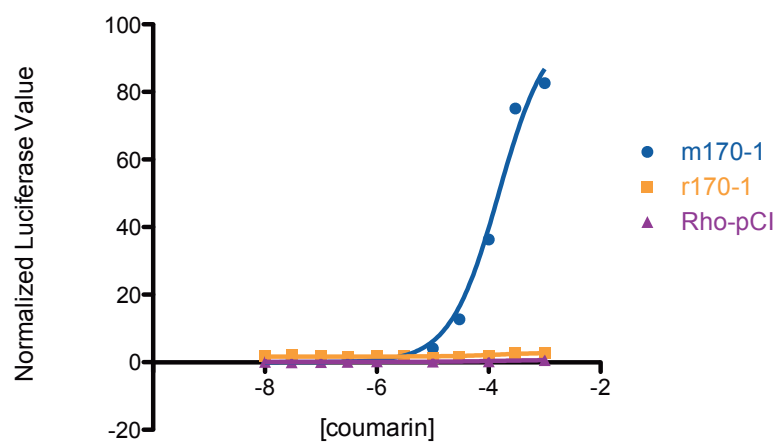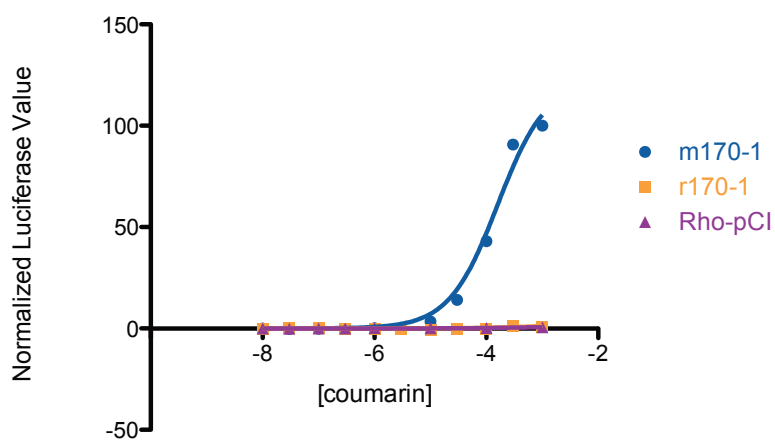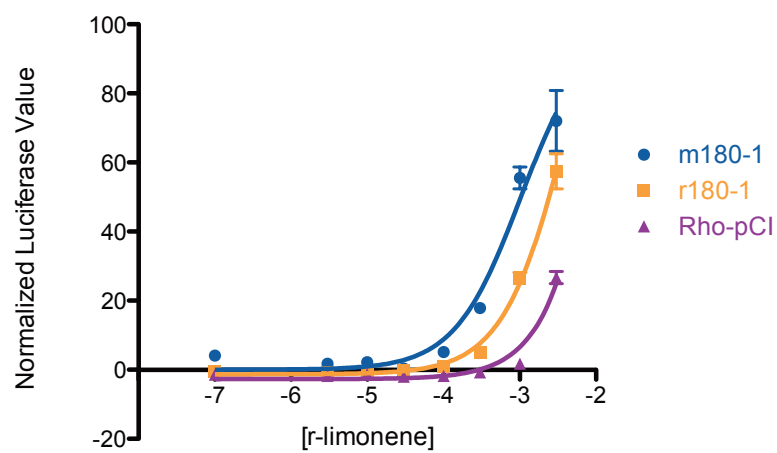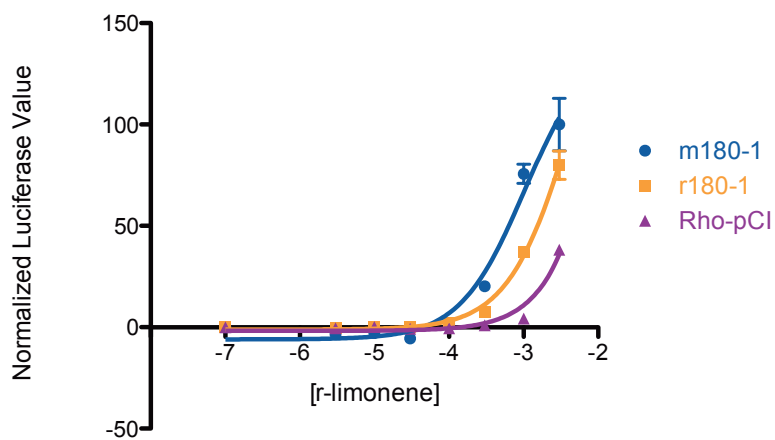

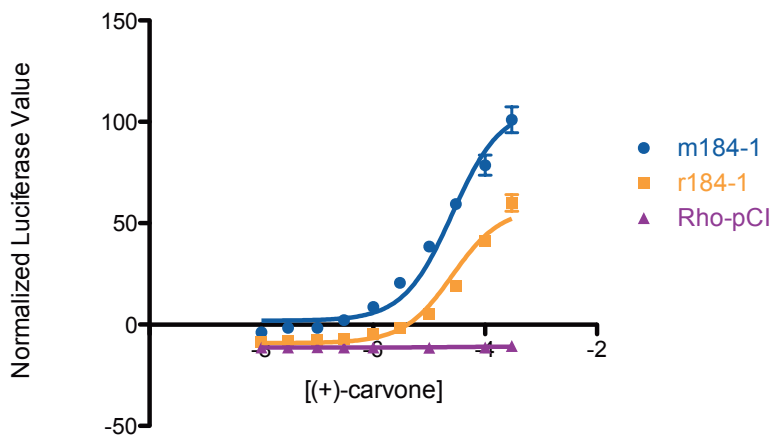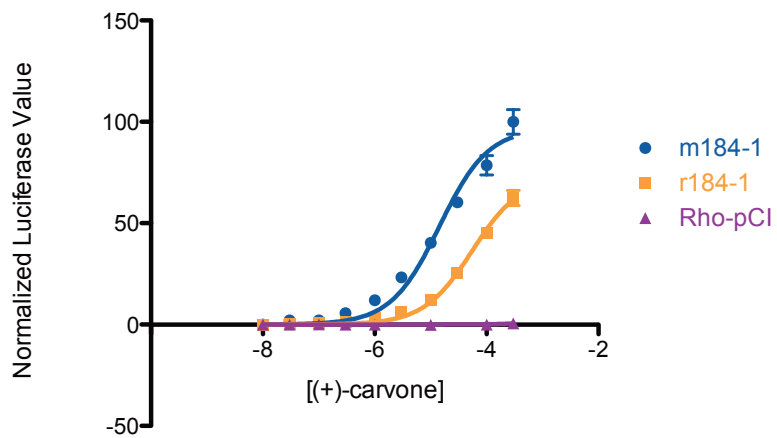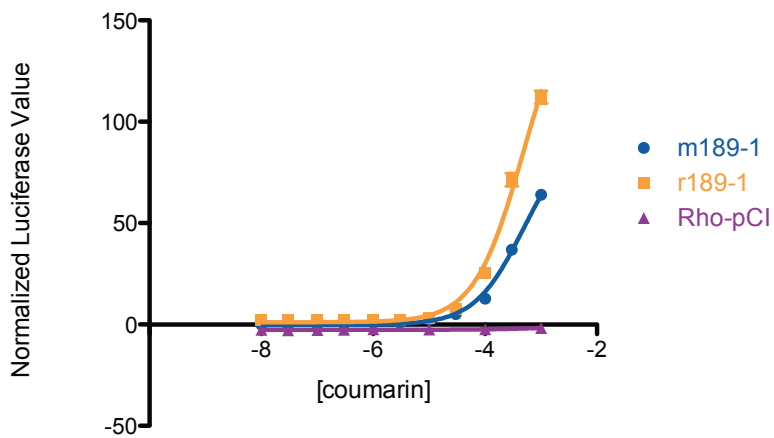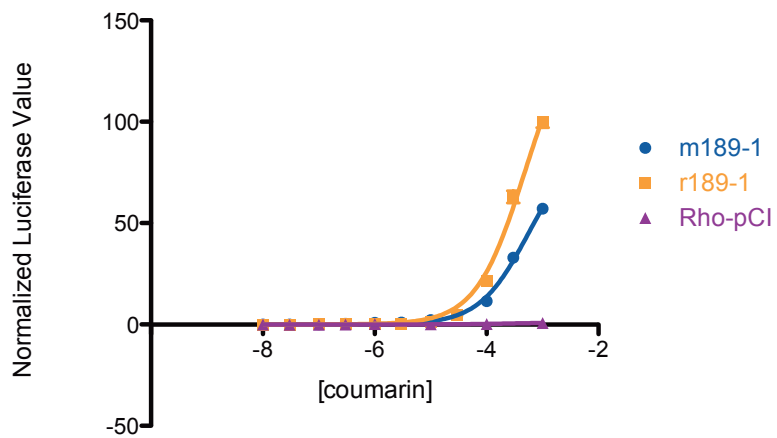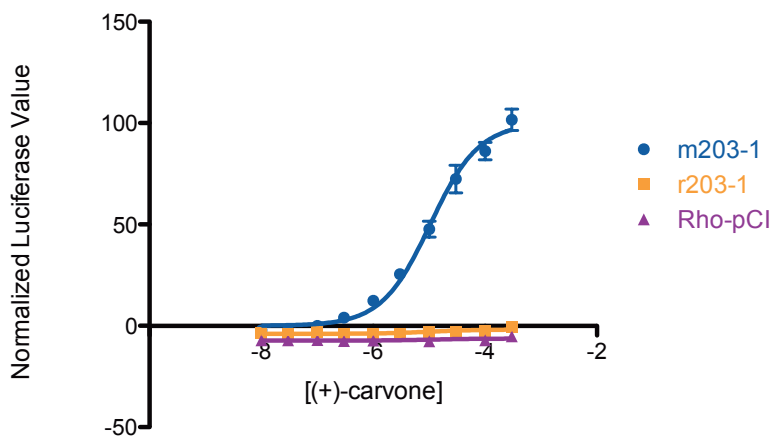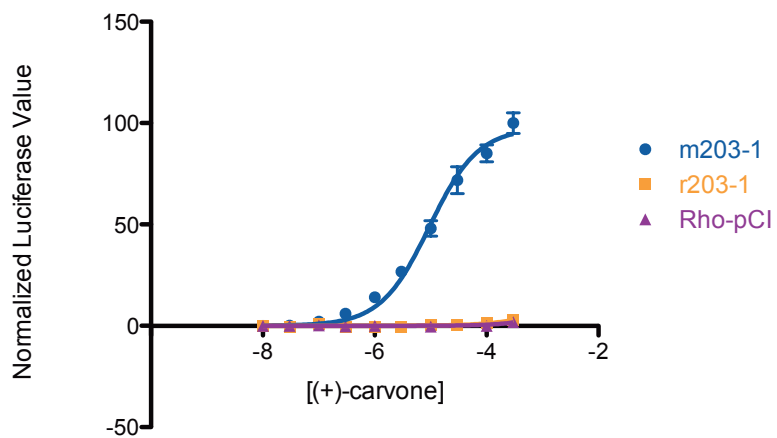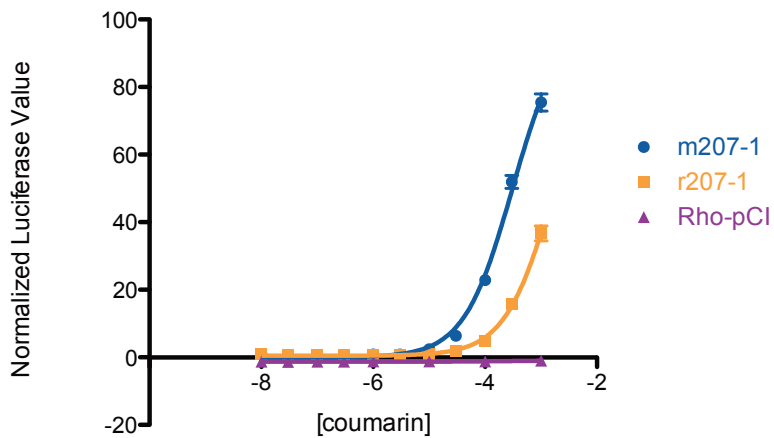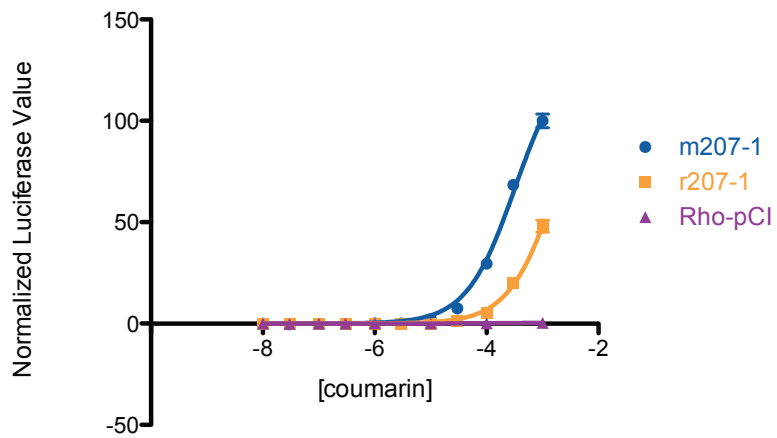

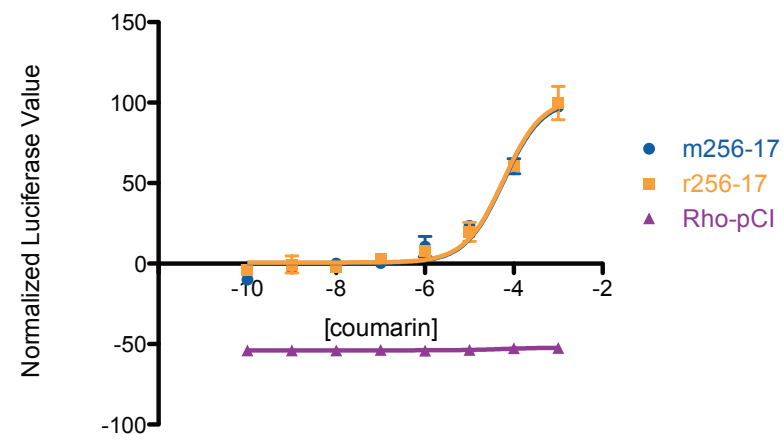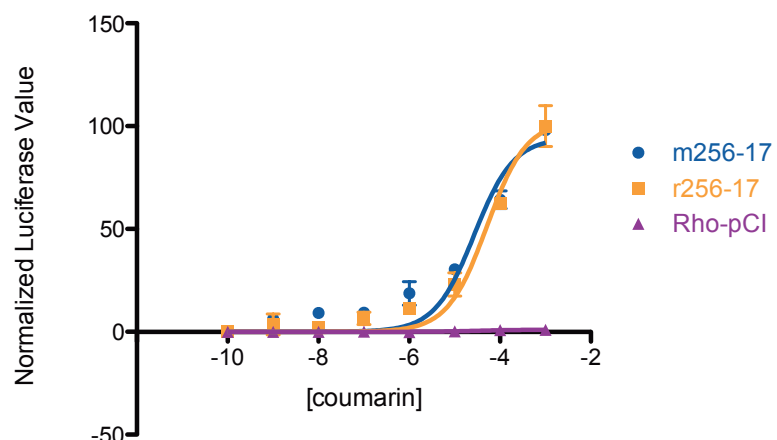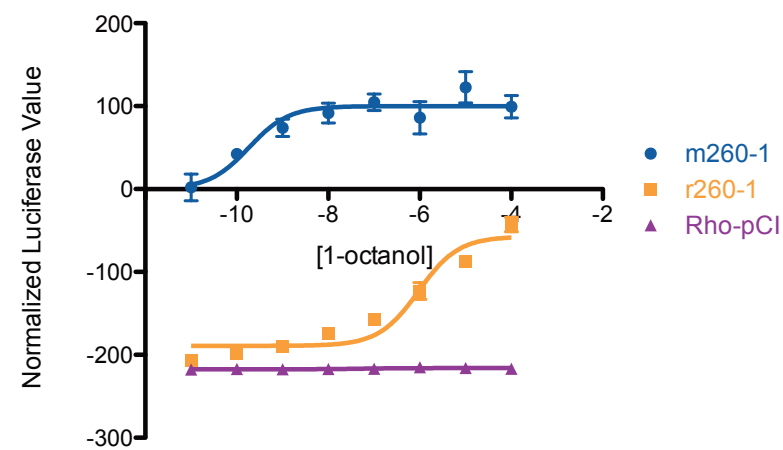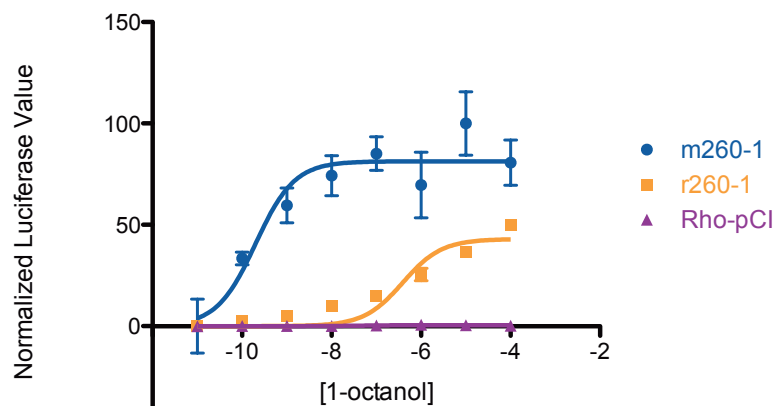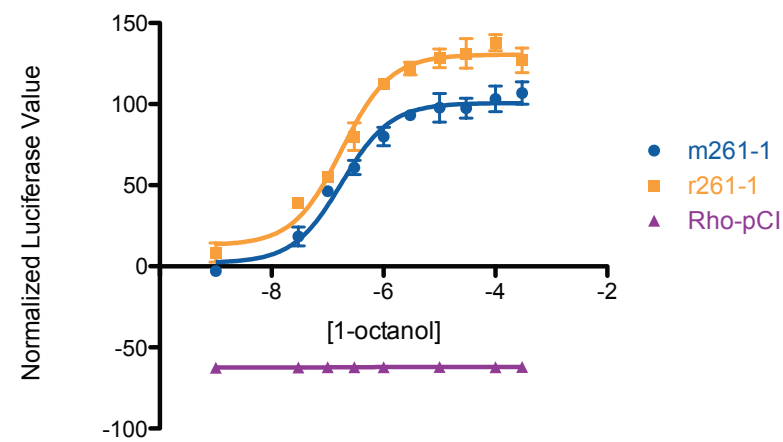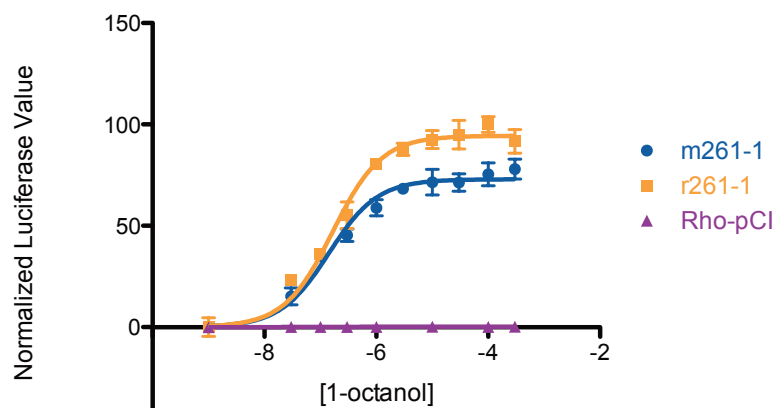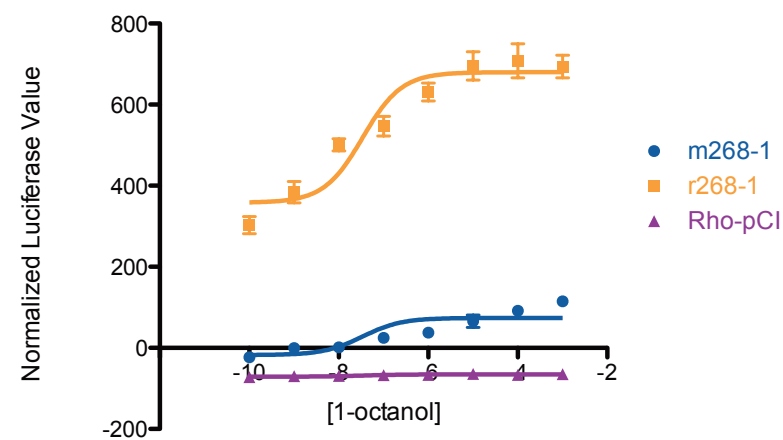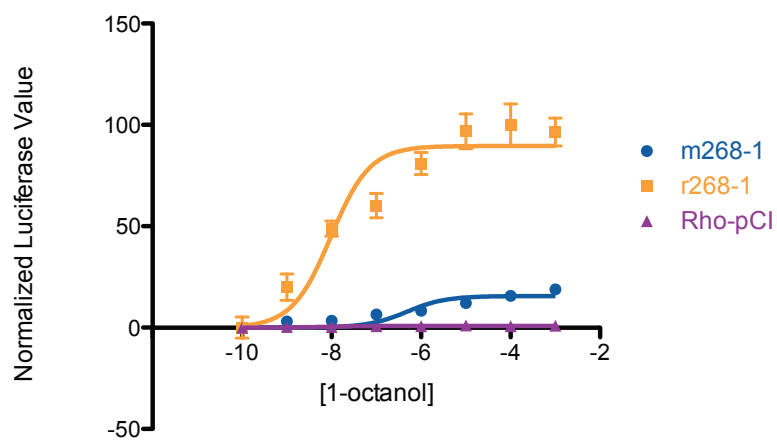

Supplement: Figure S9 — Dose-response curves for all OR ortholog sets. X-axis is the concentration of a given odor in Log Molar. Y-axis is normalized response (n = 3, ± S.E.M.). Human (h), chimpanzee (c) and rhesus macaque (m) in primate ortholog sets; mouse (m) and rat (r) for rodent ortholog sets. Vector control is Rho-pCI. Data in the left column are normalized to the human OR response. Identical data in the right column are baselined and normalized to the maximum response across a set of receptors for easier visual comparison. (PDF) [file pgen.1002821.s009.pdf]

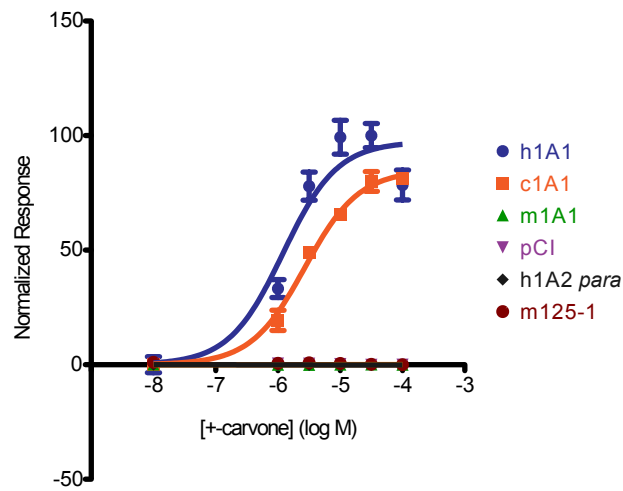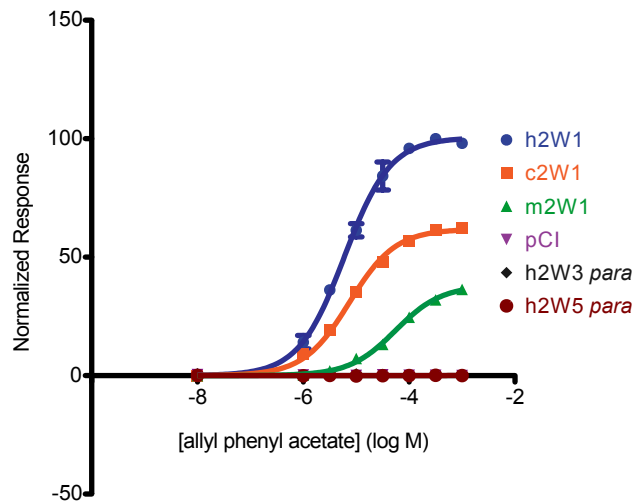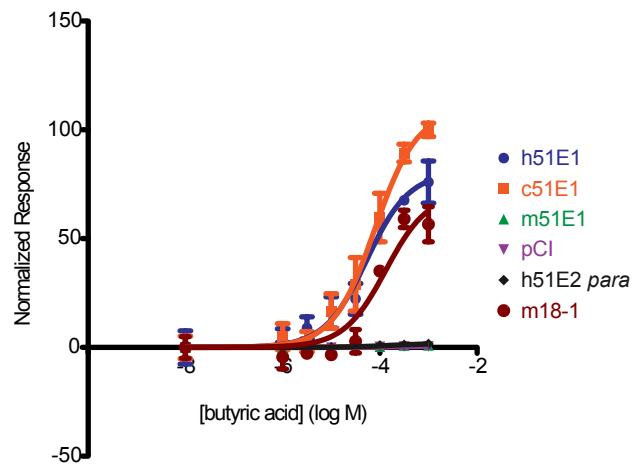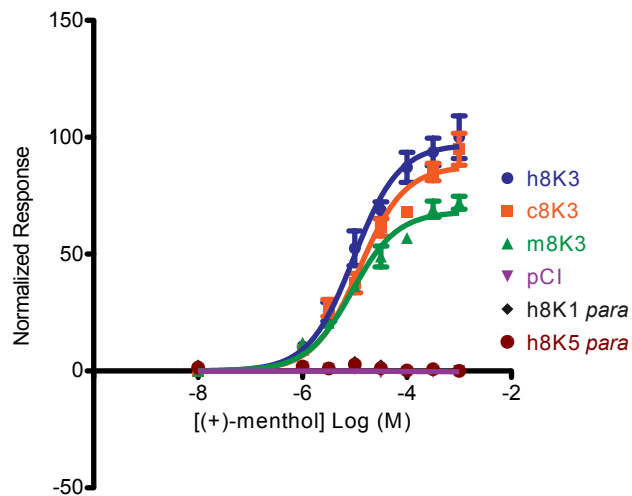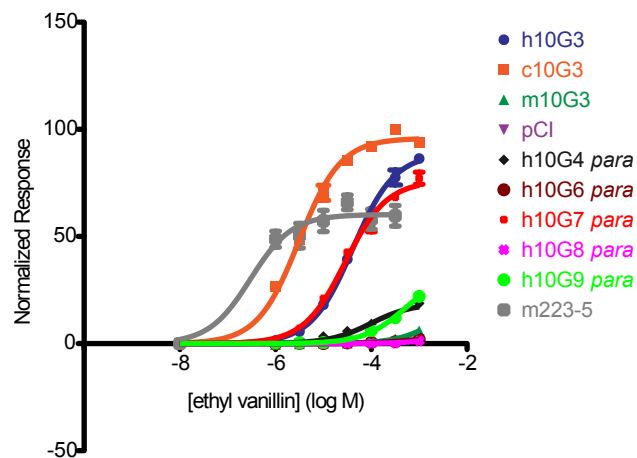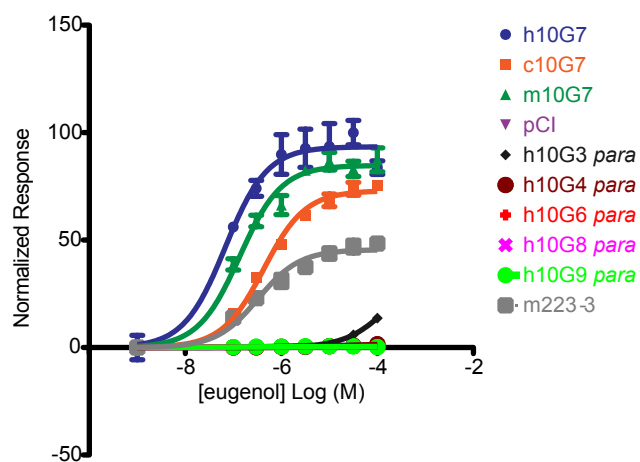

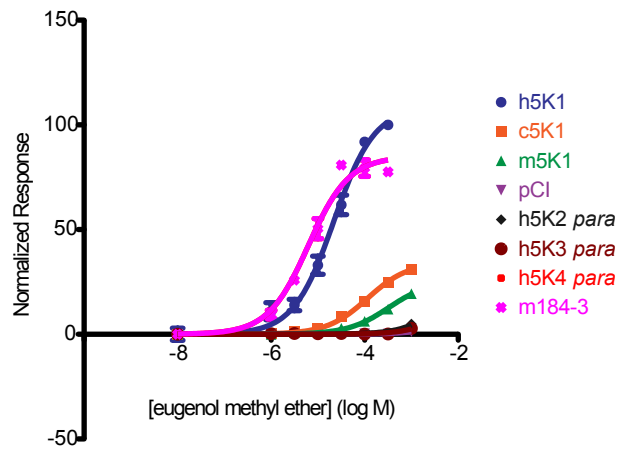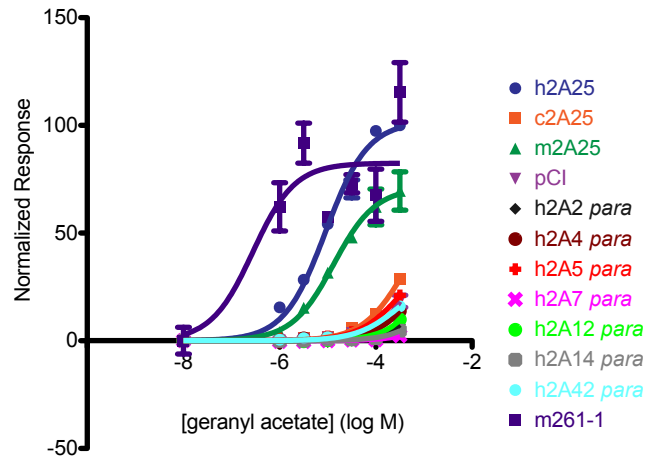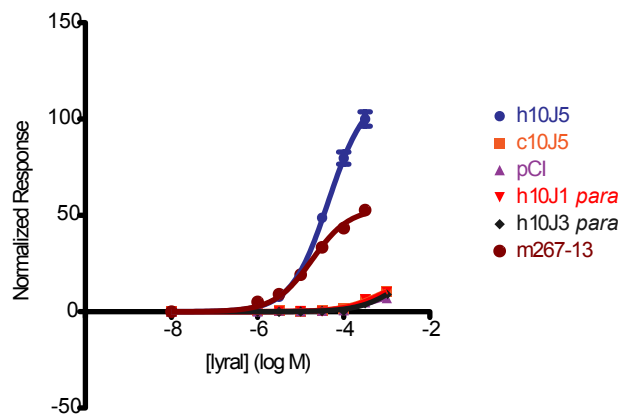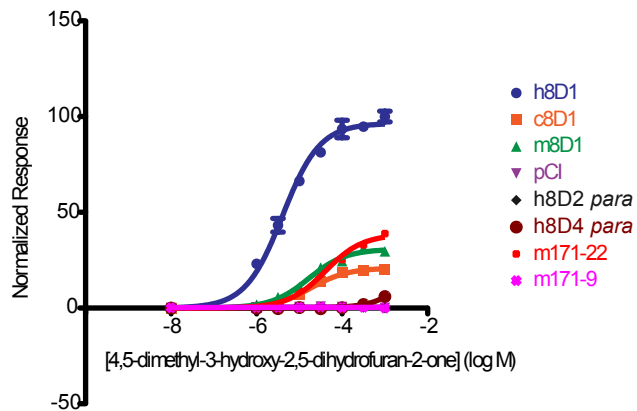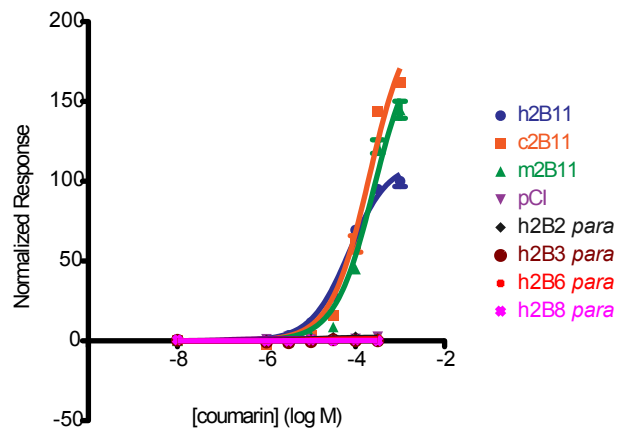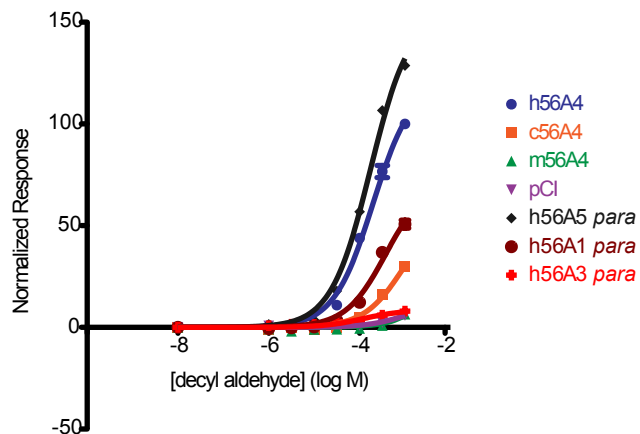

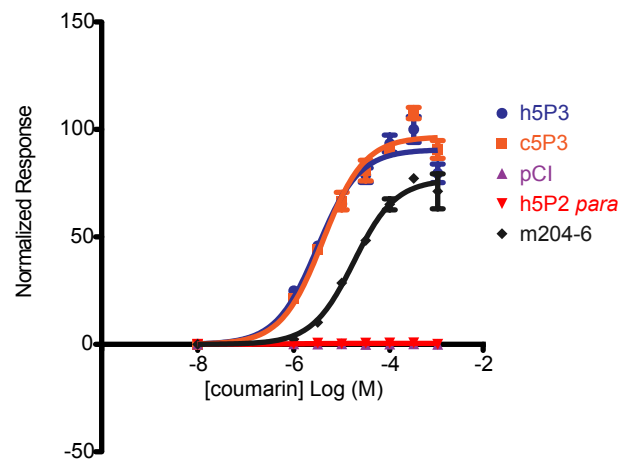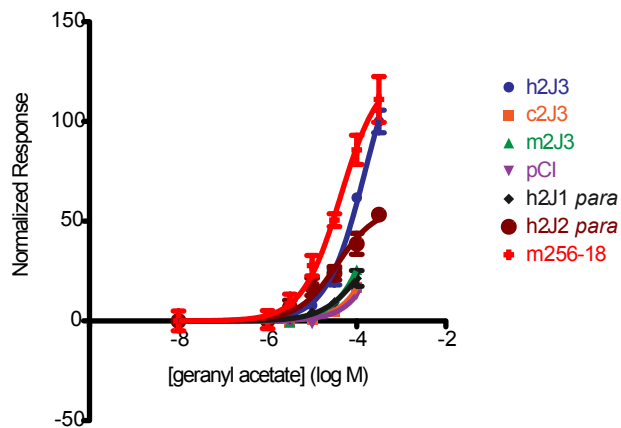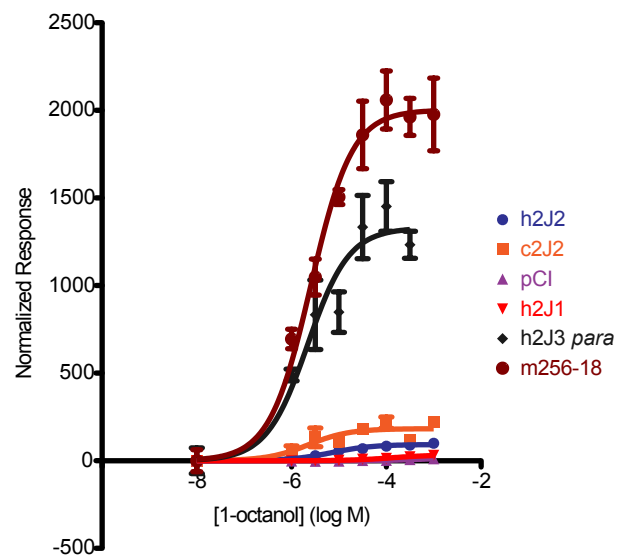

Supplement: Figure S11 — Dose-response curves for all OR orthologs and paralogs. X-axis is the concentration of a given odor in Log Molar. Y-axis is normalized response (n = 3, ± S.E.M.). Human (h), chimpanzee (c) and rhesus macaque (m), mouse receptors (m+number); para indicates a receptor that is a paralog to the human reference OR. Vector control is Rho-pCI. Data are normalized to the human OR response. (PDF) [file pgen.1002821.s011.pdf]

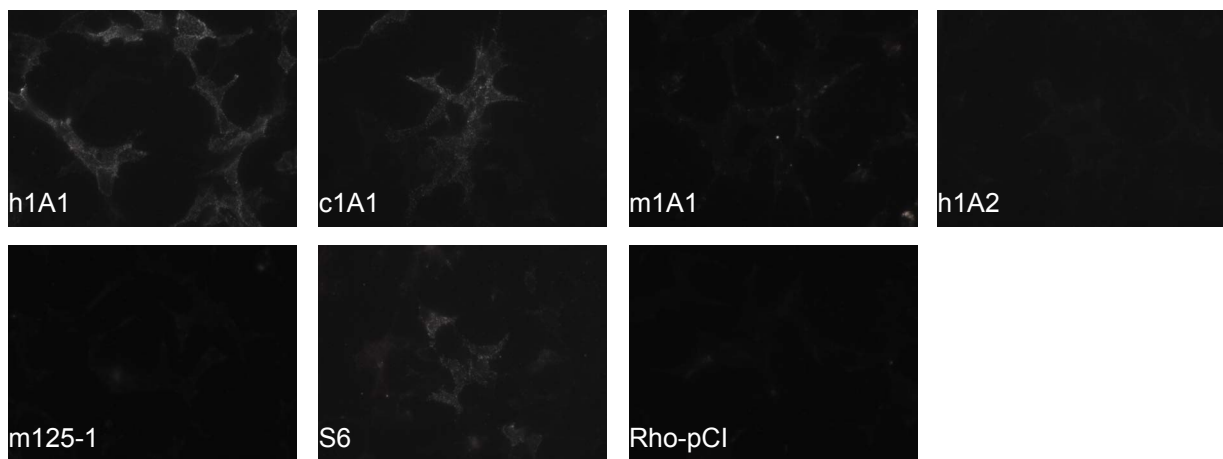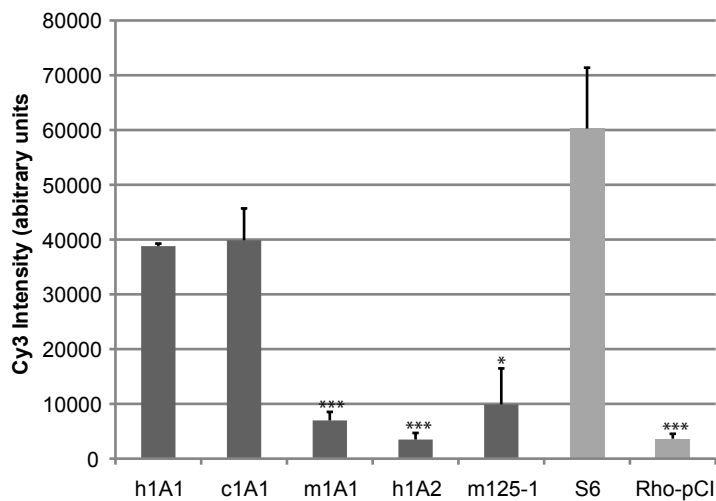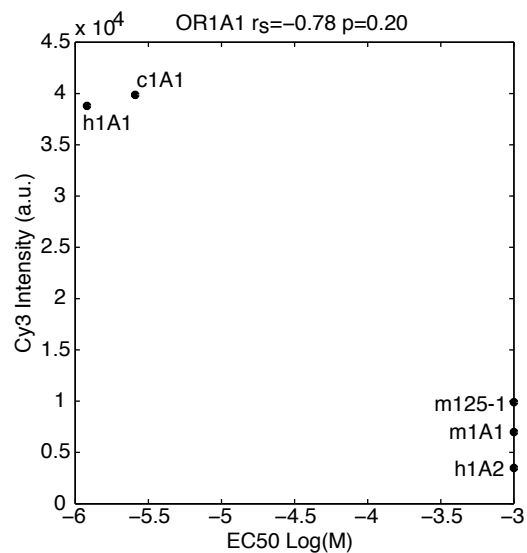

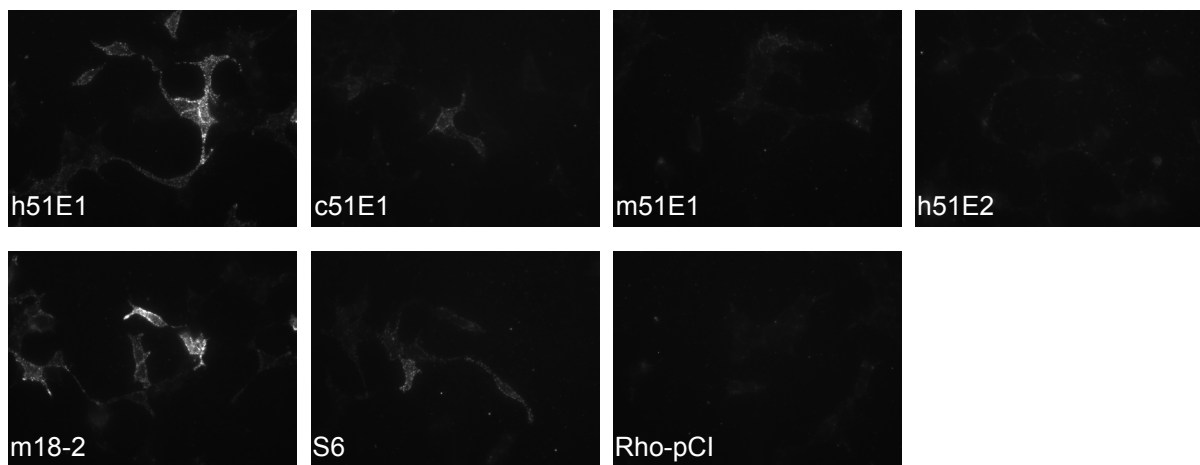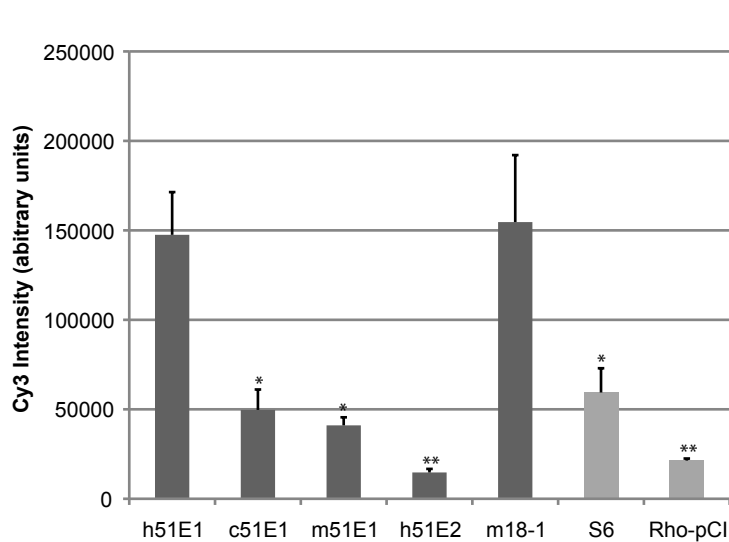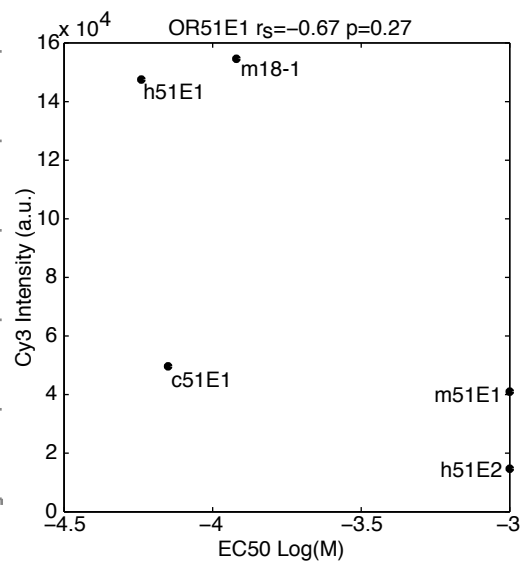

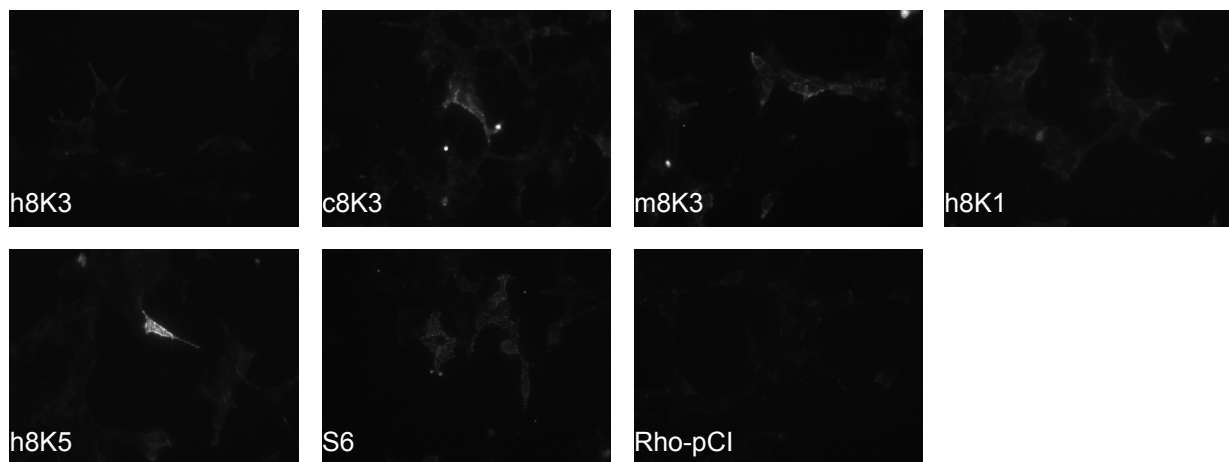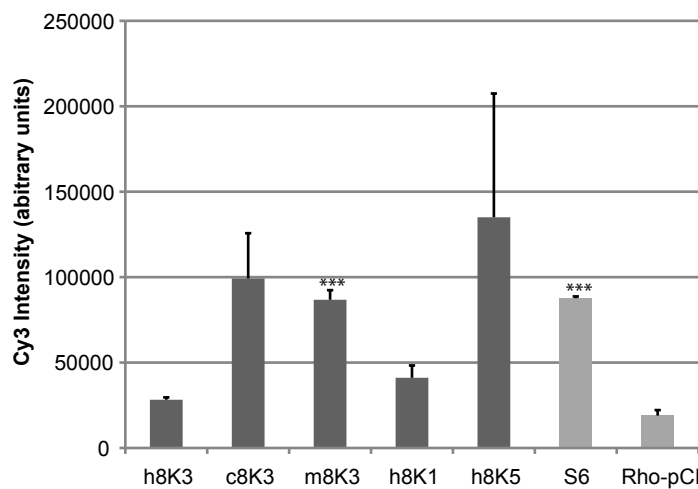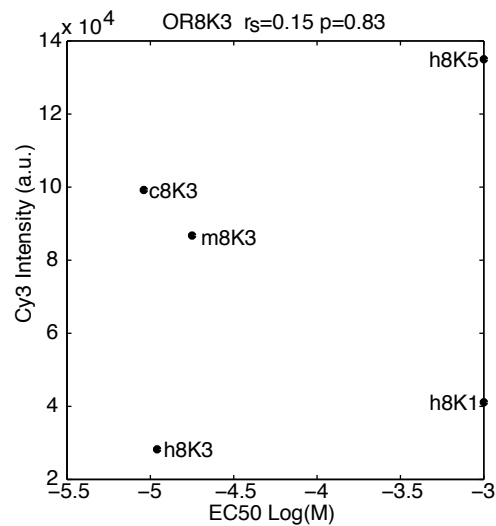

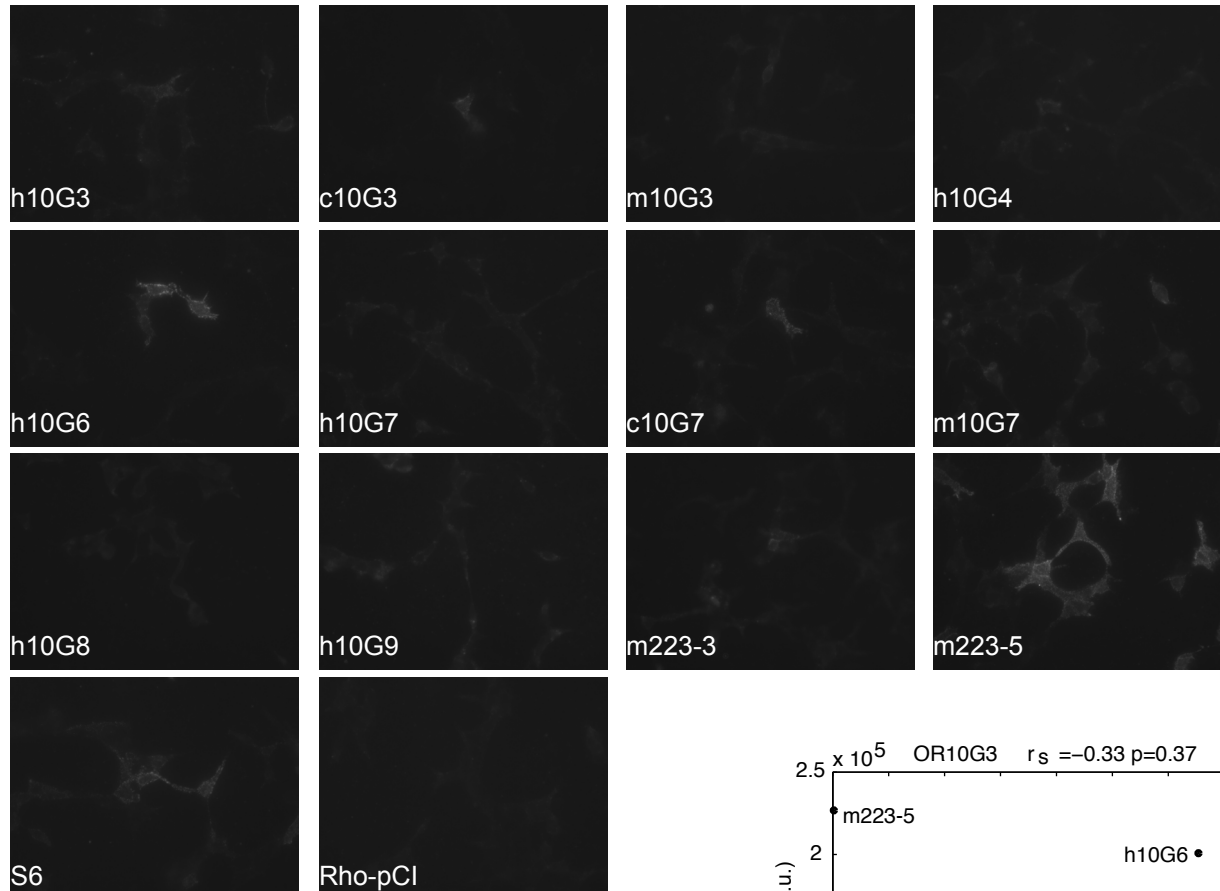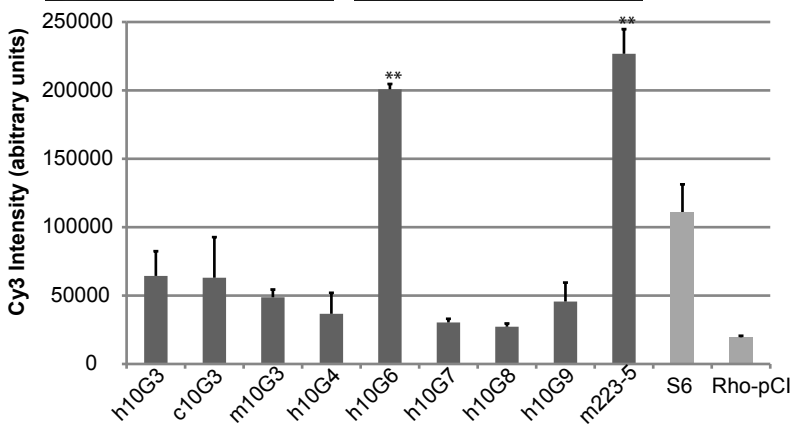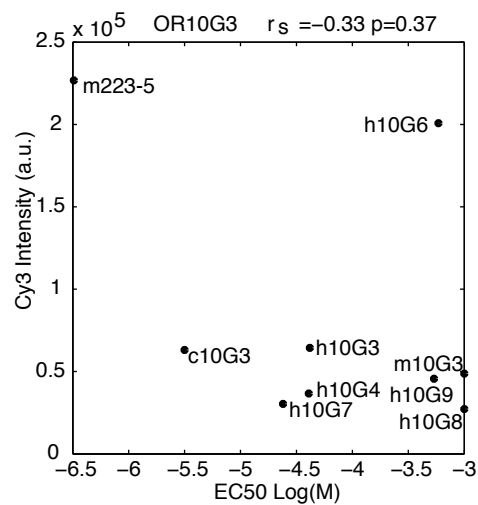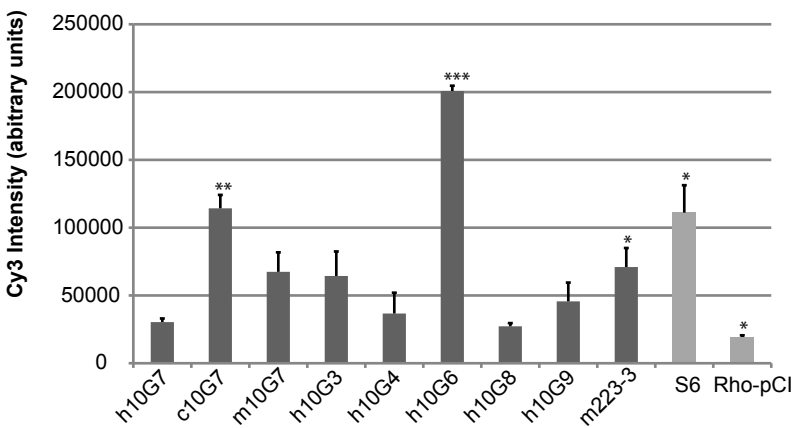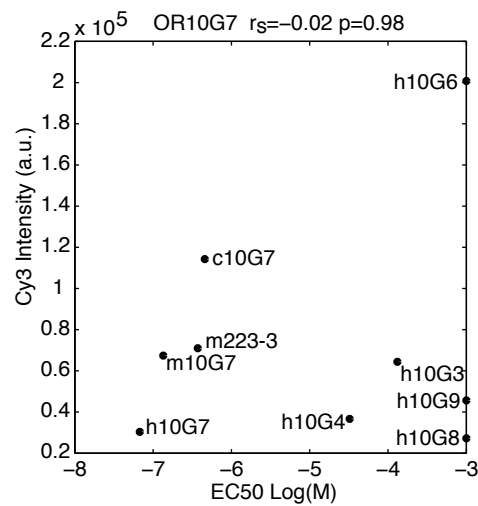

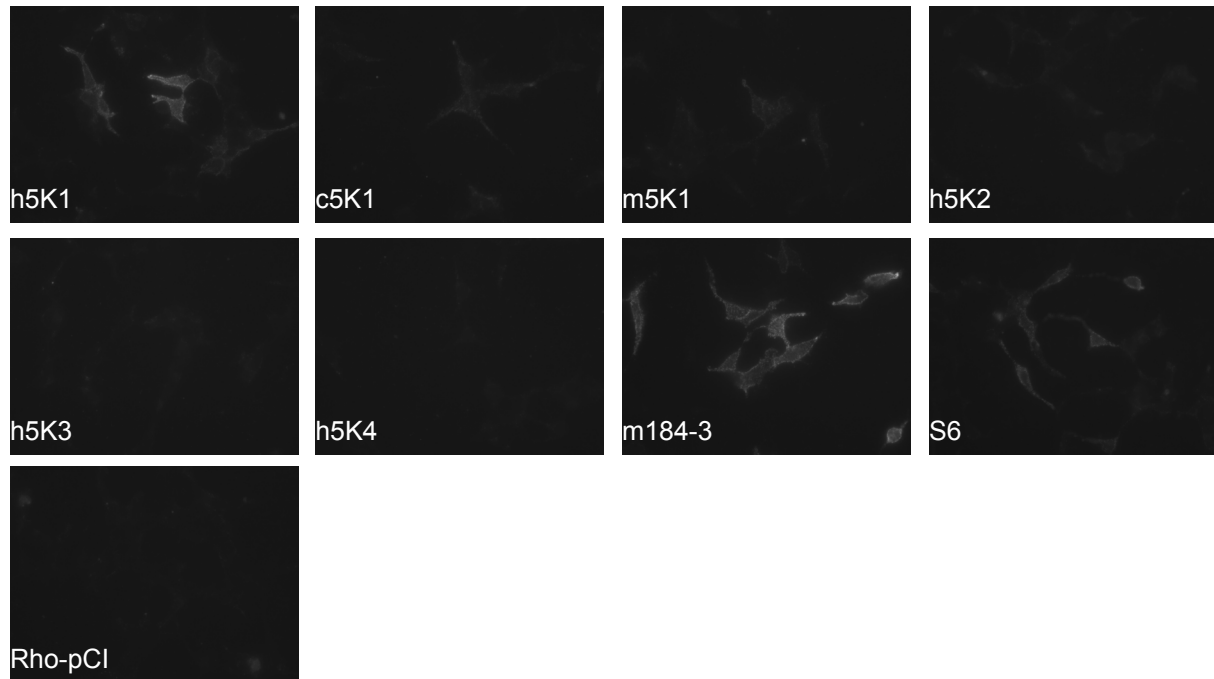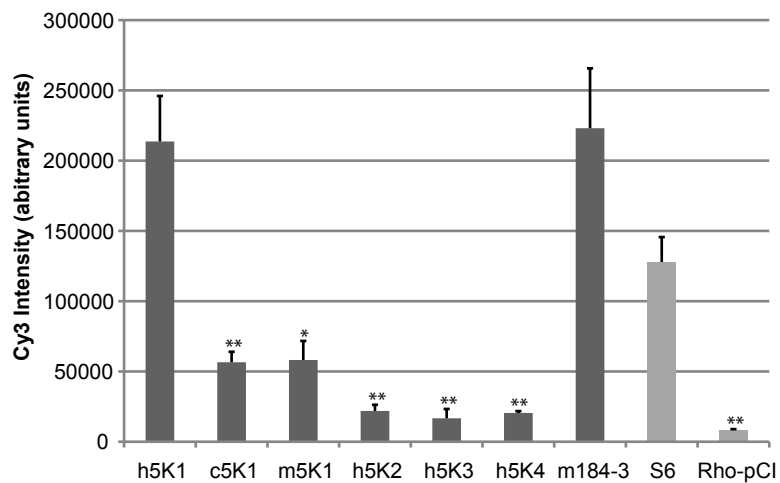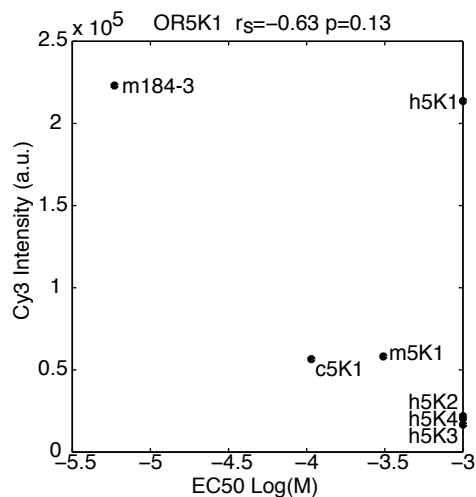

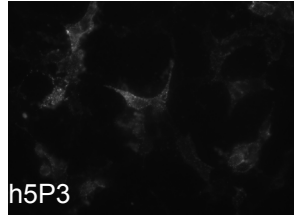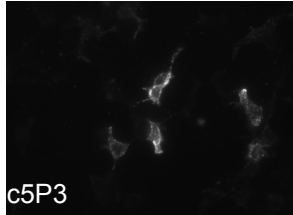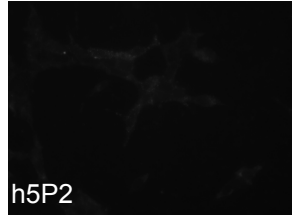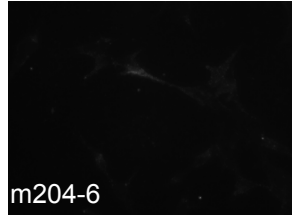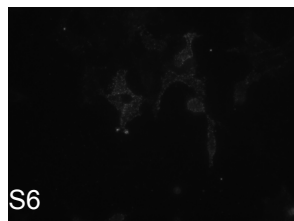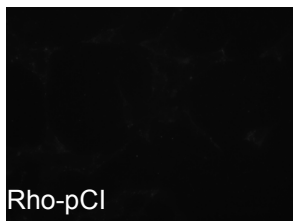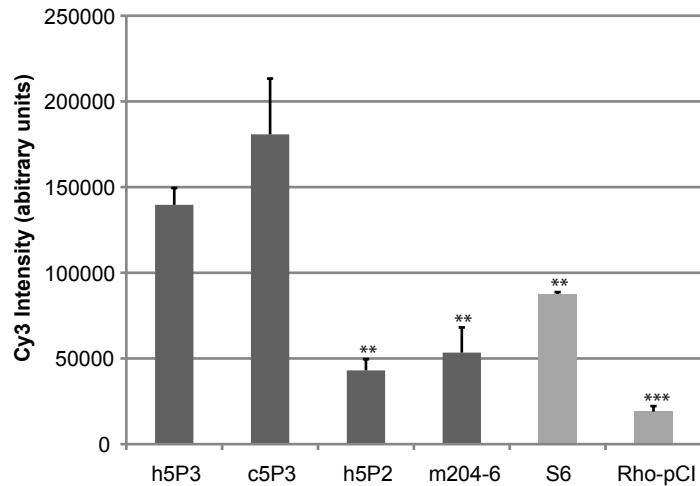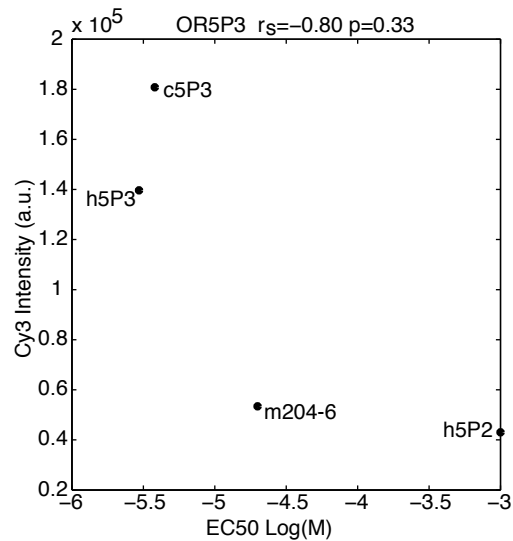

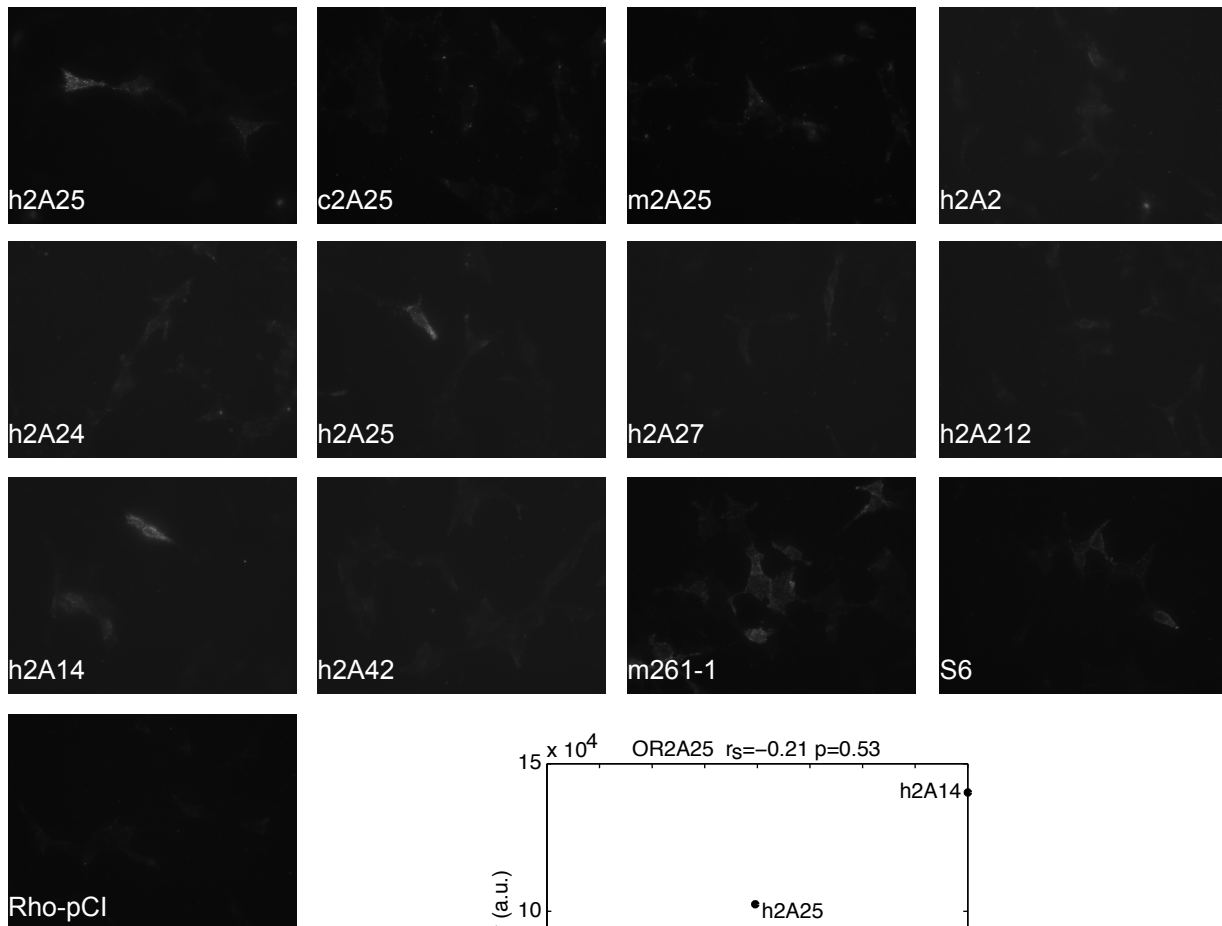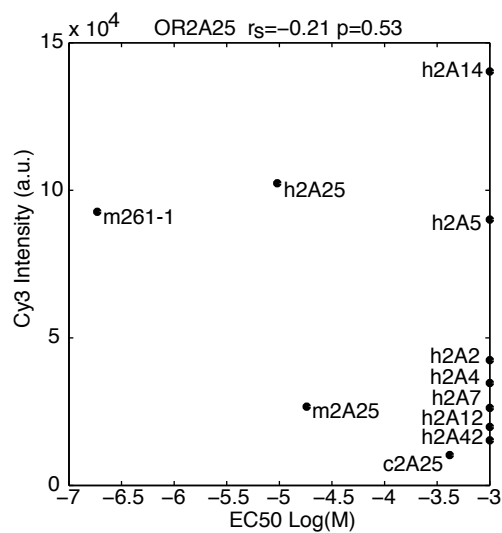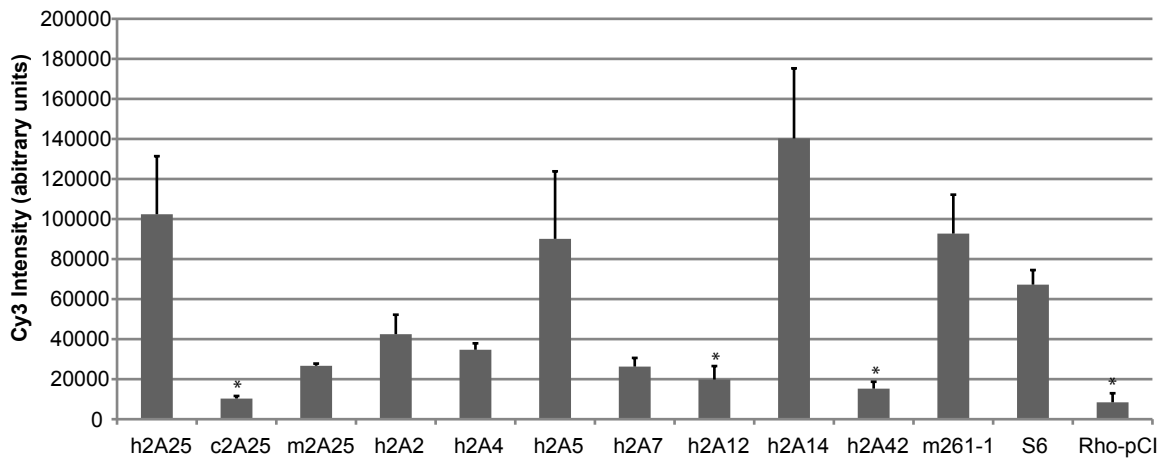

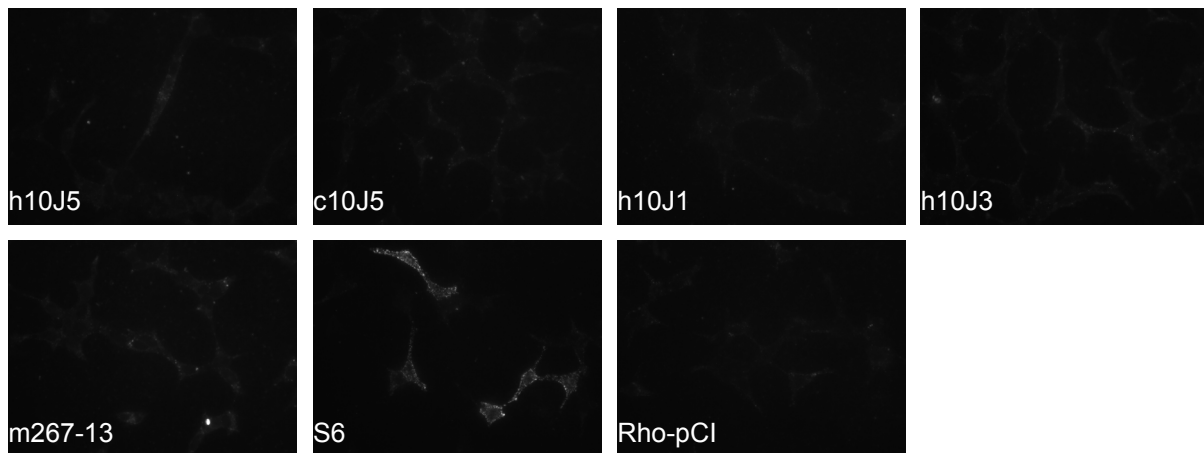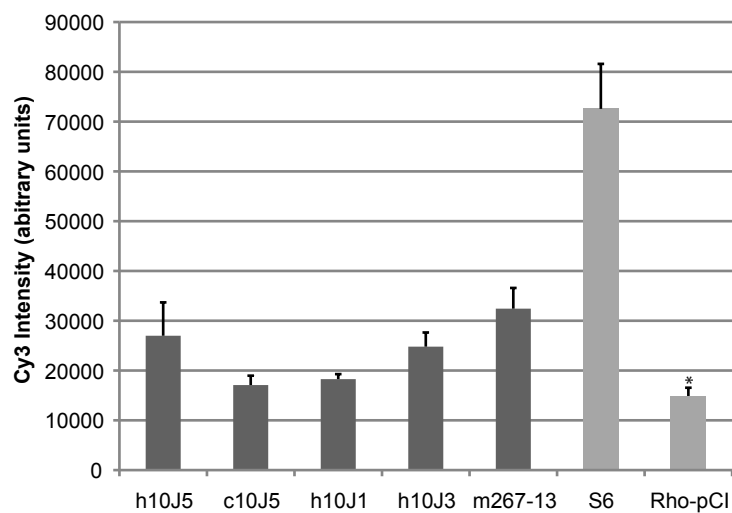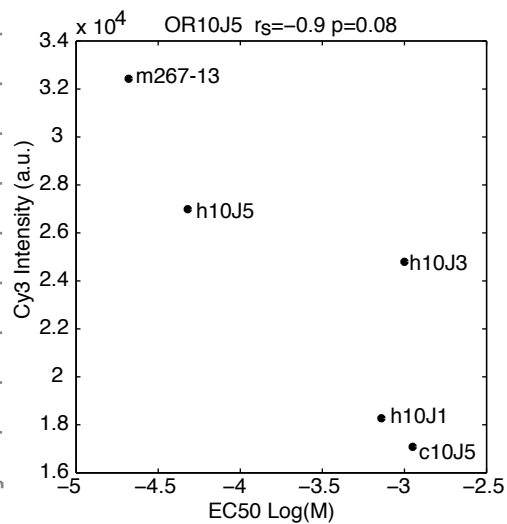

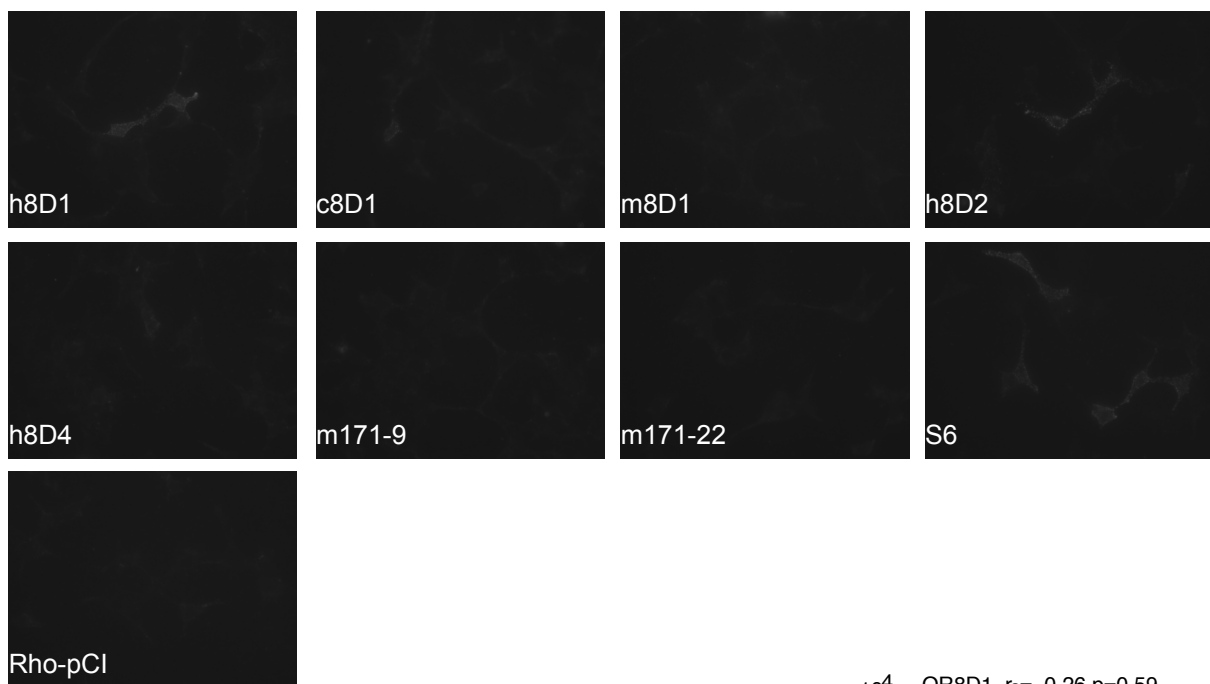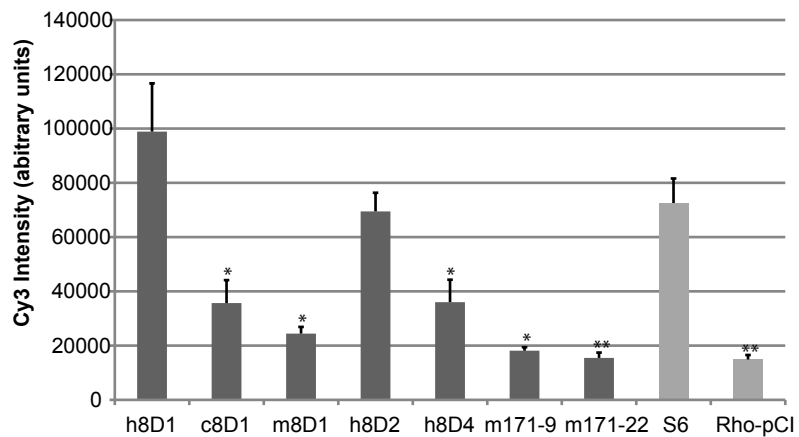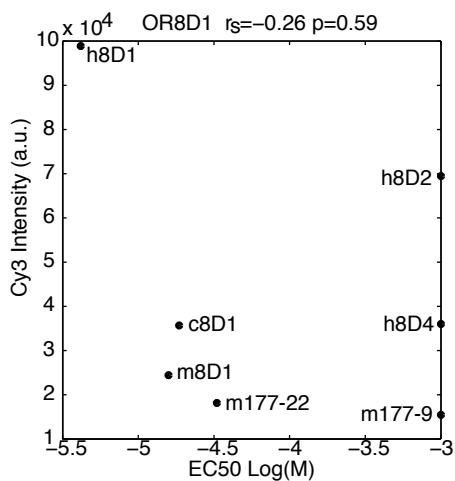

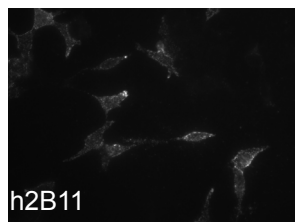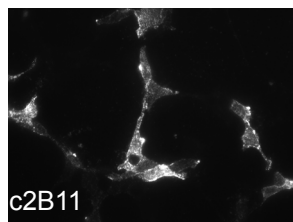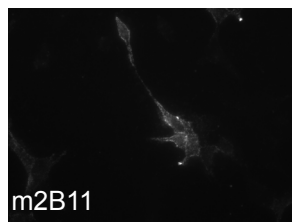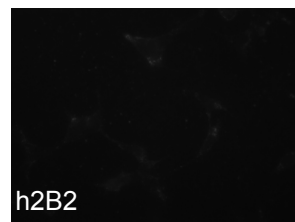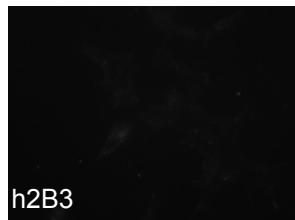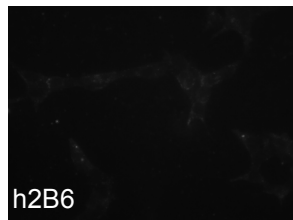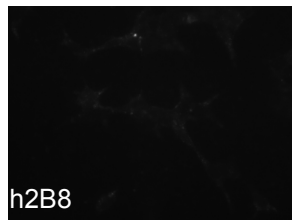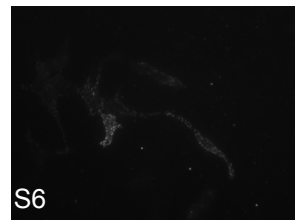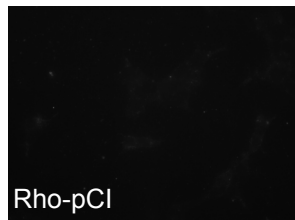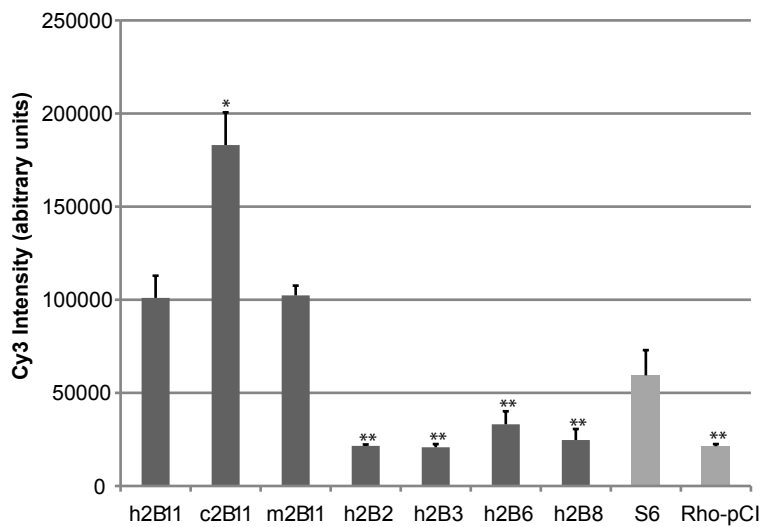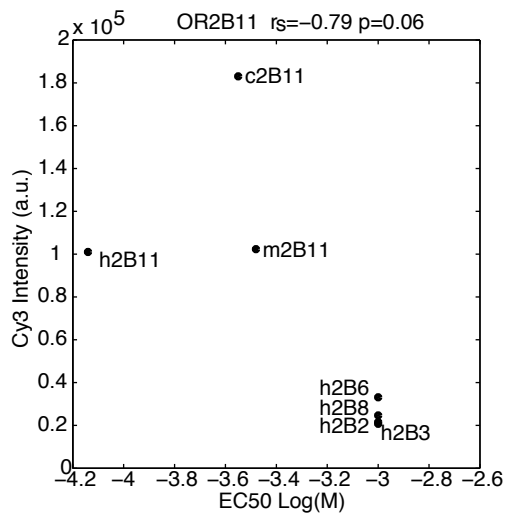

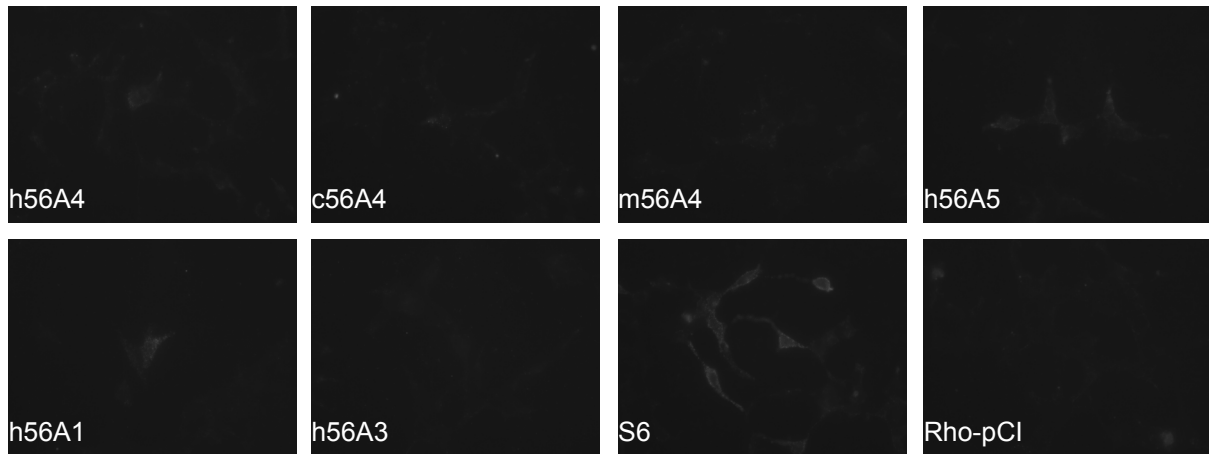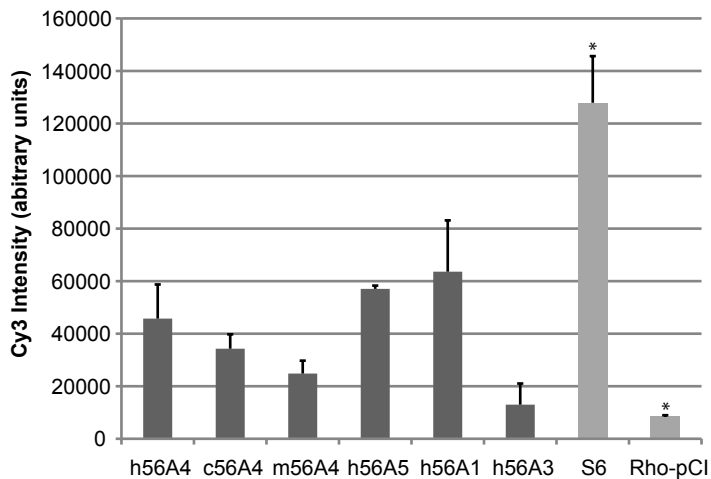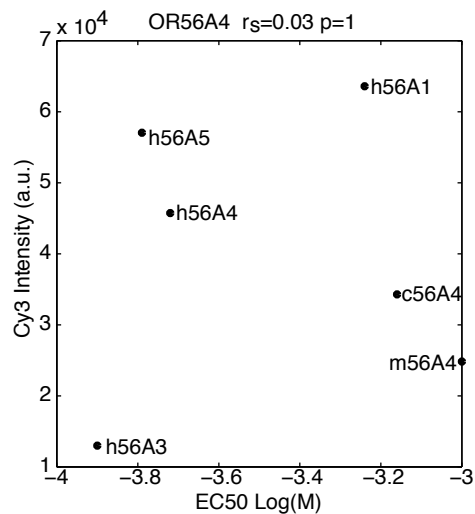

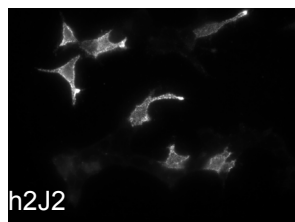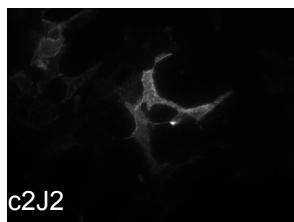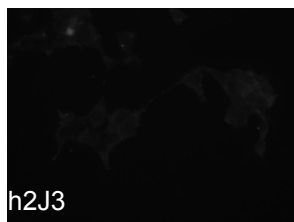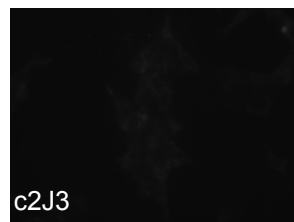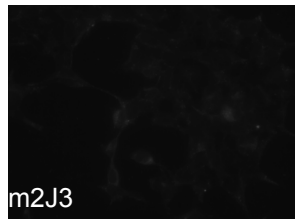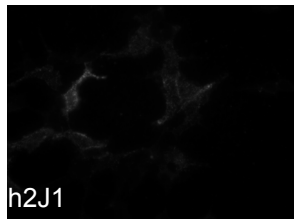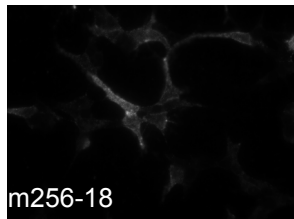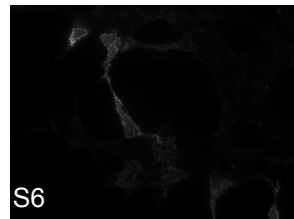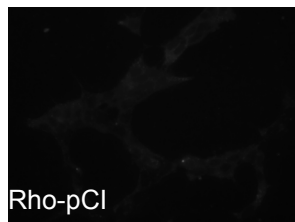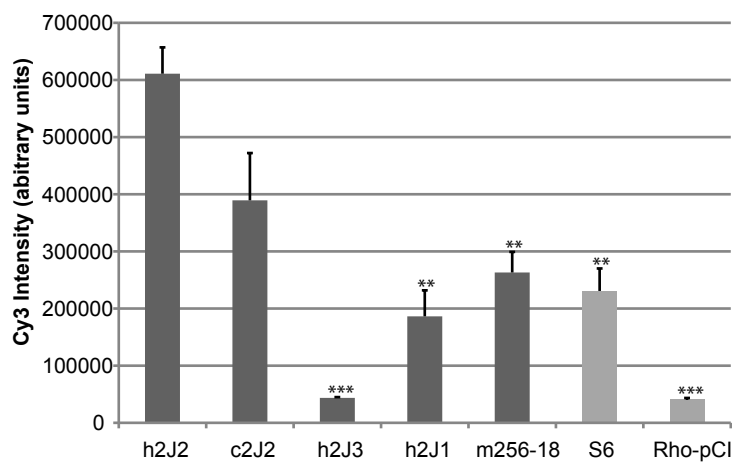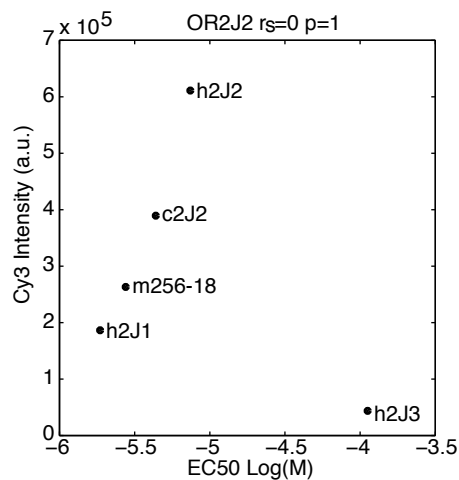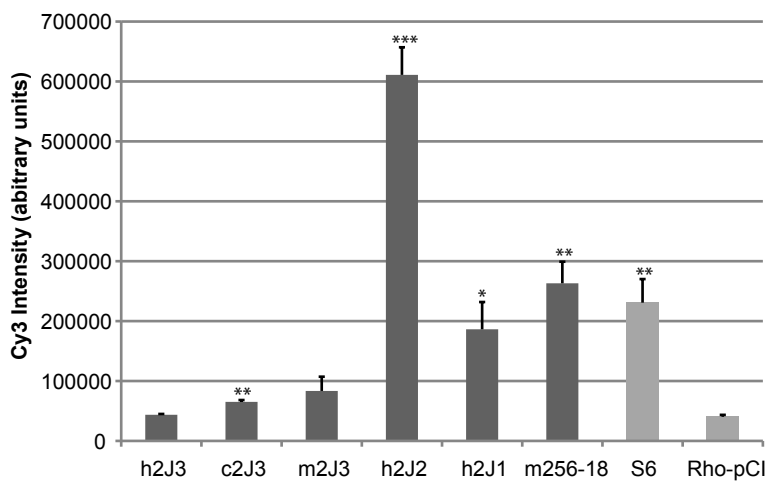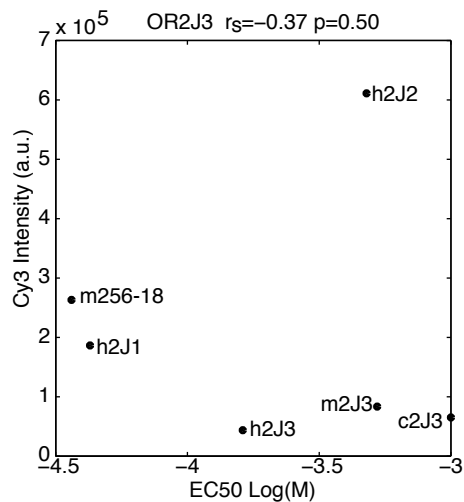

Supplement: Figure S13 — Live cell-surface staining for orthologs and subfamily members. Each panel contains a quantification of live cell-surface expression of each receptor. * p<0.05,** p<0.01, ***p<0.001 (student's t-test) when compared to primary human OR in each set. Y-axis denotes the average Cy3 intensity in arbitrary units (a.u.) (n = 3, ± S.E.M.) with S6 as positive control and Rho-pCI as negative control. Representative images of live cell-surface staining for each receptor. Cy3 intensity is plotted against the EC50 of each receptor to a common ligand (and analyzed using Spearman's correlation; see Table S5, Table S7). If receptor did not respond, EC50 was plotted at -3 Log Molar. (PDF) [file pgen.1002821.s013.pdf]
