# Supplementary material for: Functional Evolution of Mammalian Odorant Receptors
Source: PLoS Genet. 2012 Jul 12;8(7):e1002821. doi: 10.1371/journal.pgen.1002821 (PMC3395614; doi:10.1371/journal.pgen.1002821)
Supplement: Figure S3 — Analysis of 22 amino acid positions in orthologs predicted to be involved in ligand binding. (A) Alignment of corresponding 22 amino acids predicted to be involved in ligand binding [17] from our primate and rodent OR orthologs. Amino acid color categories: KR, red; AFILMVW, blue; NQST, green; HY, teal; C, salmon; DE, purple; P, yellow; G, orange. (B) Amino acid similarity of the 22 amino acids using Grantham's distance plotted against functional distance, as defined by the correlation (1-R, Table S2) of the response across the 42-odor panel for ORs responding to more than three odors (rs = 0.32, p = .04, Spearman's correlation; compare to Grantham's amino acid similarity for full length OR sequences (Figure 3B)). (PDF) [file pgen.1002821.s003.pdf]

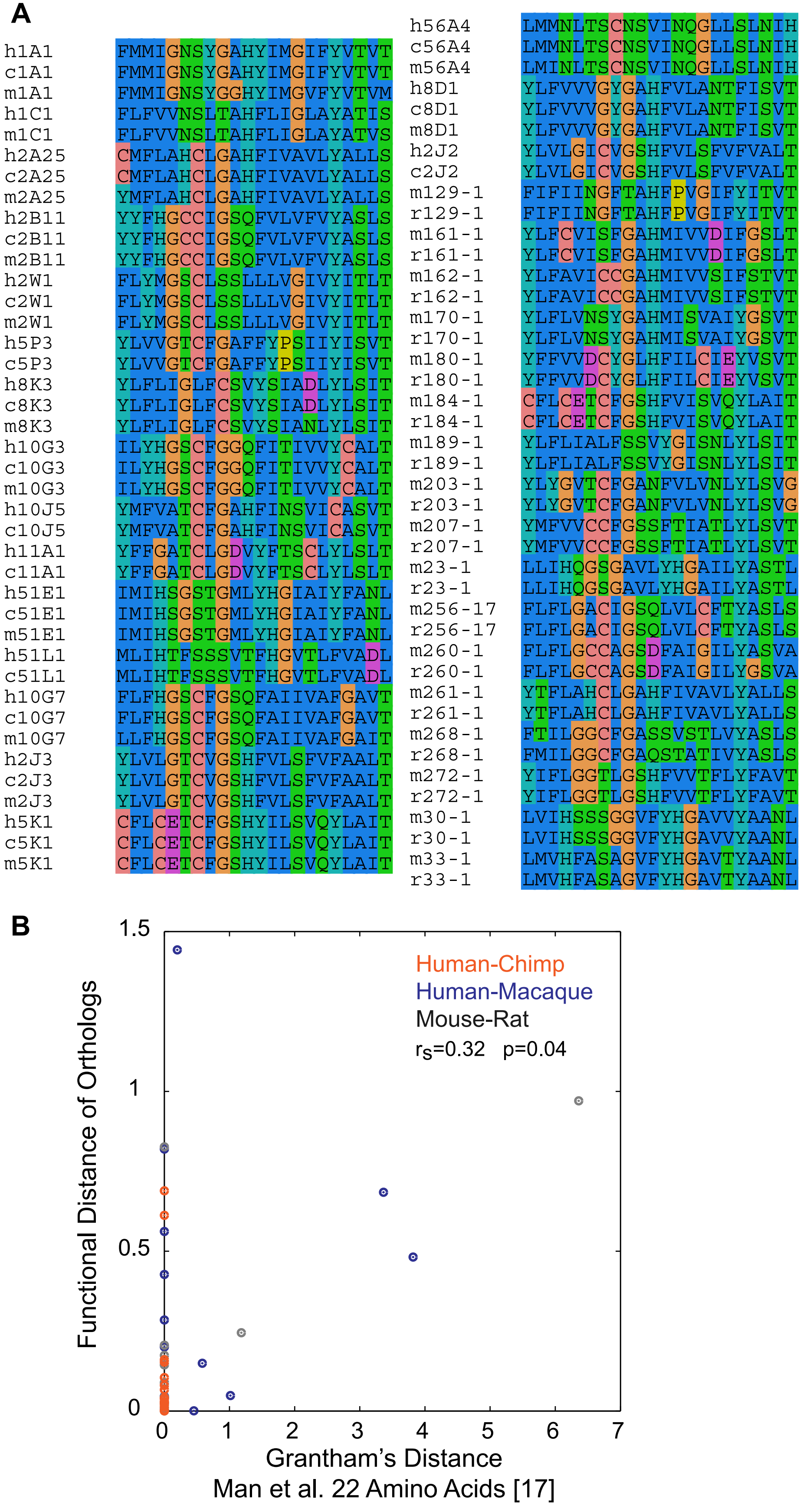
A
h1A1 FMMIGNSYGAHYIMGIFYVTVT
c1A1 FMMIGNSYGAHYIMGIFYVTVT
m1A1 FMMIGNSYGGHYIMGVFYVTVM
h1C1 FLFVVNSLTAHFLIGLAYATIS
m1C1 FLFVVNSLTAHFLIGLAYATVS
h2A25 CMFLAHCLGAHFIVAVLYALLS
c2A25 CMFLAHCLGAHFIVAVLYALLS
m2A25 YMFLAHCLGAHFIVAVLYALLS
h2B11 YYFHGCCIGSQFVLVFVYASLS
c2B11 YYFHGCCIGSQFVLVFVYASLS
m2B11 YYFHGCCIGSQFVLVFVYASLS
h2W1 FLYMGSCLSSLLLVGIVYITLT
c2W1 FLYMGSCLSSLLLVGIVYITLT
m2W1 FLYMGSCLSSLLLVGIVYITLT
h5P3 YLVVGTCFGAFFYPSIIYISVT
c5P3 YLVVGTCFGAFFYPSIIYISVT
h8K3 YLFLIGLFCSVYSIADLYLSIT
c8K3 YLFLIGLFCSVYSIADLYLSIT
m8K3 YLFLIGLFCSVYSIANLYLSIT
h10G3 ILYHGSCFGGQFITIVVYCALT
c10G3 ILYHGSCFGGQFITIVVYCALT
m10G3 ILYHGSCFGGQFITIVVYCALT
h10J5 YMFVATCFGAHFINSVICASVT
c10J5 YMFVATCFGAHFINSVICASVT
h11A1 YFFGATCLGDVYFTSCLYLSLT
c11A1 YFFGATCLGDVYFTSCLYLSLT
h51E1 IMIHSGSTGMLYHGIAIYFANL
c51E1 IMIHSGSTGMLYHGIAIYFANL
m51E1 IMIHSGSTGMLYHGIAIYFANL
h51L1 MLIHTFSSSVTFHGVTLFVADL
c51L1 MLIHTFSSSVTFHGVTLFVADL
h10G7 FLFHGSCFGSQFAIIVAFGAVT
c10G7 FLFHGSCFGSQFAIIVAFGAVT
m10G7 LLFHGSCFGSQFAIIVAFGAIT
h2J3 YLVLGTCVGSHFVLSFVFAALT
c2J3 YLVLGTCVGSHFVLSFVFAALT
m2J3 YLVLGTCVGSHFVLSFVFAALT
h5K1 CFLCETCFGSHYILSVQYLAIT
c5K1 CFLCETCFGSHYILSVQYLAIT
m5K1 CFLCETCFGSHYILSVQYLAIT
h56A4 LMMNLTSCNSVINQGLLSLNIH
c56A4 LMMNLTSCNSVINQGLLSLNIH
m56A4 LMINLTSCNSVINQGLLSLNIH
h8D1 YLFVVVGYGAHFVLANTFISVT
c8D1 YLFVVVGYGAHFVLANTFISVT
m8D1 YLFVVVGYGAHFVLANTFISVT
h2J2 YLVLGICVGSHFVLSFVFVALT
c2J2 YLVLGICVGSHFVLSFVFVALT
m129-1 FIFIINGFTAHFPVGIFYITVT
r129-1 FIFIINGFTAHFPVGIFYITVT
m161-1 YLFCVISFGAHMIVVDIFGSLT
r161-1 YLFCVISFGAHMIVVDIFGSLT
m162-1 YLFAVICCGAHMIVVSIFSTVT
r162-1 YLFAVICCGAHMIVVSIFSTVT
m170-1 YLFLVNSYGAHMISVAIYGSVT
r170-1 YLFLVNSYGAHMISVAIYGSVT
m180-1 YFFVVDCYGLHFILCIEYVSVT
r180-1 YFFVVDCYGLHFILCIEYVSVT
m184-1 CFLCETCFGSHFVISVQYLAIT
r184-1 CFLCETCFGSHFVISVQYLAIT
m189-1 YLFLIALFSSVYGISNLYLSIT
r189-1 YLFLIALFSSVYGISNLYLSIT
m203-1 YLYGVTCFGANFVLVNLYLSVG
r203-1 YLYGVTCFGANFVLVNLYLSVG
m207-1 YMFVVCCFGSSFTIATLYLSVT
r207-1 YMFVVCCFGSSFTIATLYLSVT
m23-1 LLIHQGSGAVLYHGAILYASTL
r23-1 LLIHQGSGAVLYHGAILYASTL
m256-17 FLFLGACIGSQLVLCFTYASLS
r256-17 FLFLGACIGSQLVLCFTYASLS
m260-1 FLFLGCCAGSDFAIGILYASVA
r260-1 FLFLGCCAGSDFAIGILYGSVA
m261-1 YTFLAHCLGAHFIVAVLYALLS
r261-1 YTFLAHCLGAHFIVAVLYALLS
m268-1 FTILGGCFGASSVSTLVYASLS
r268-1 FMILGGCFGAQSTATIVYASLS
m272-1 YIFLGGTLGSHFVVTFLYFAVT
r272-1 YIFLGGTLGSHFVVTFLYFAVT
m30-1 LVIHSSSGGVFYHGAVVYAANL
r30-1 LVIHSSSGGVFYHGAVVYAANL
m33-1 LMVHFASAGVFYHGAVTYAANL
r33-1 LMVHFASAGVFYHGAVTYAANL
B
Human-Chimp
Human-Macaque
Mouse-Rat
r_S=0.32 p=0.04
Functional Distance of Orthologs
1.5
1
0.5
0
0 1 2 3 4 5 6 7
Grantham's Distance
Man et al. 22 Amino Acids [17]
