# Supplementary material for: Functional Evolution of Mammalian Odorant Receptors
Source: PLoS Genet. 2012 Jul 12;8(7):e1002821. doi: 10.1371/journal.pgen.1002821 (PMC3395614; doi:10.1371/journal.pgen.1002821)
Supplement: Figure S6 — Sensitivity-ordered tuning curves for all OR ortholog sets. 42 odors are displayed along the x-axis according to the response elicited from the human OR for primate sets and mouse OR for rodent pairs, with the best response in the center of the distribution. The order of the odors is the same between orthologs in a set, but different across receptors. The y-axis represents the luciferase response to an odor at 100 µM (n = 3, ± S.E.). Negative values on the y-axis indicate the odor elicited an inhibitory response on OR signaling. If a given odorant did not signifcantly activate any of the ORs above the no-odor control (2-tailed t-test, α = 0.05/42), the response was set to zero. Odors are listed in Table S3. (PDF) [file pgen.1002821.s006.pdf]

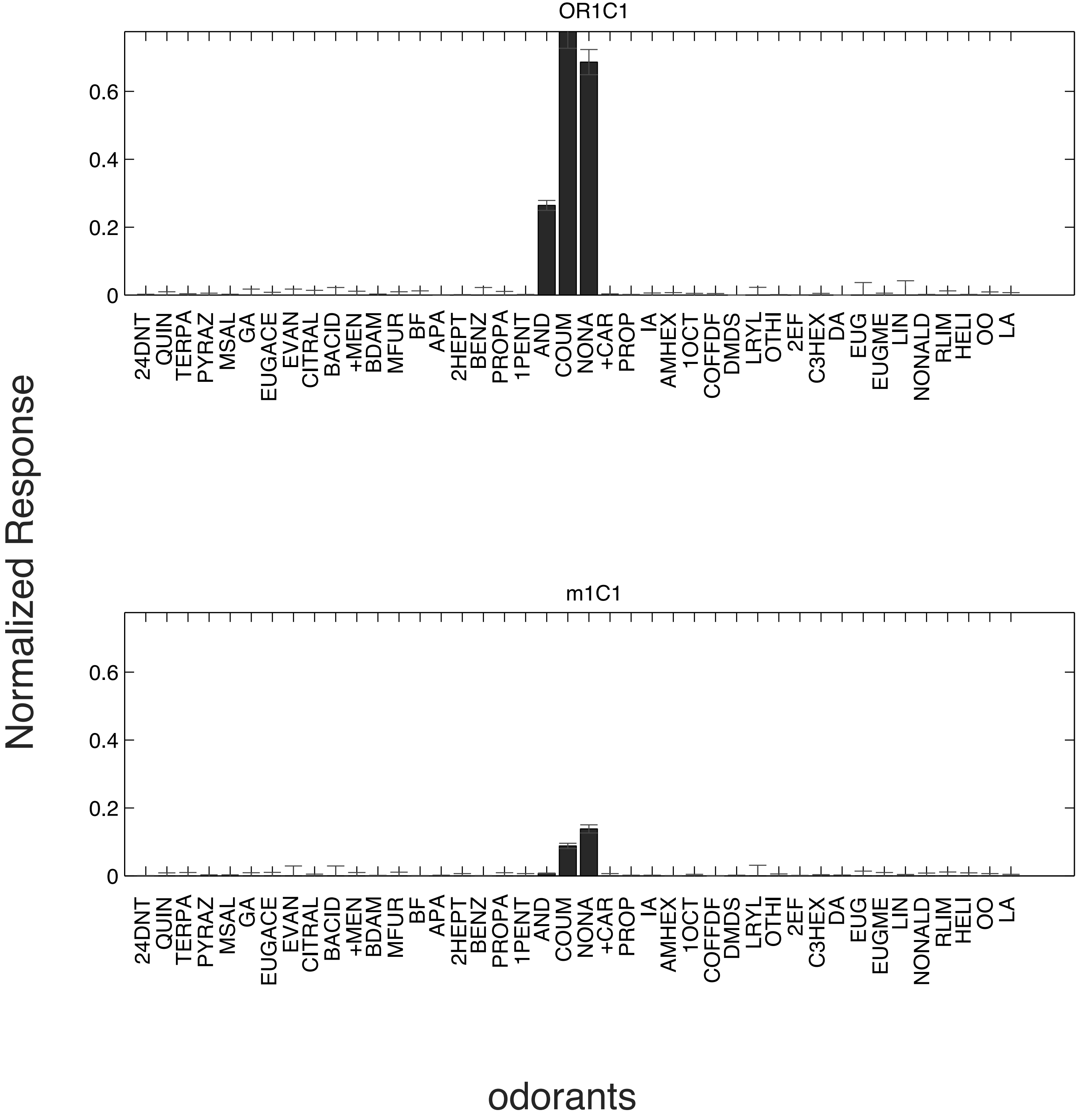
OR1C1
m1C1
Normalized Response
odorants
0
0.2
0.4
0.6
24DNT
QUIN
TERPA
PYRAZ
MSAL
GA
EUGACE
EVAN
CITRAL
BACID
+MEN
BDAM
MFUR
BF
APA
2HEPT
BENZ
PROPA
1PENT
AND
COUM
NONA
+CAR
PROP
IA
AMHEX
1OCT
COFFDF
DMDS
LRYL
OTHI
2EF
C3HEX
DA
EUG
EUGME
LIN
NONALD
RLIM
HELI
OO
LA

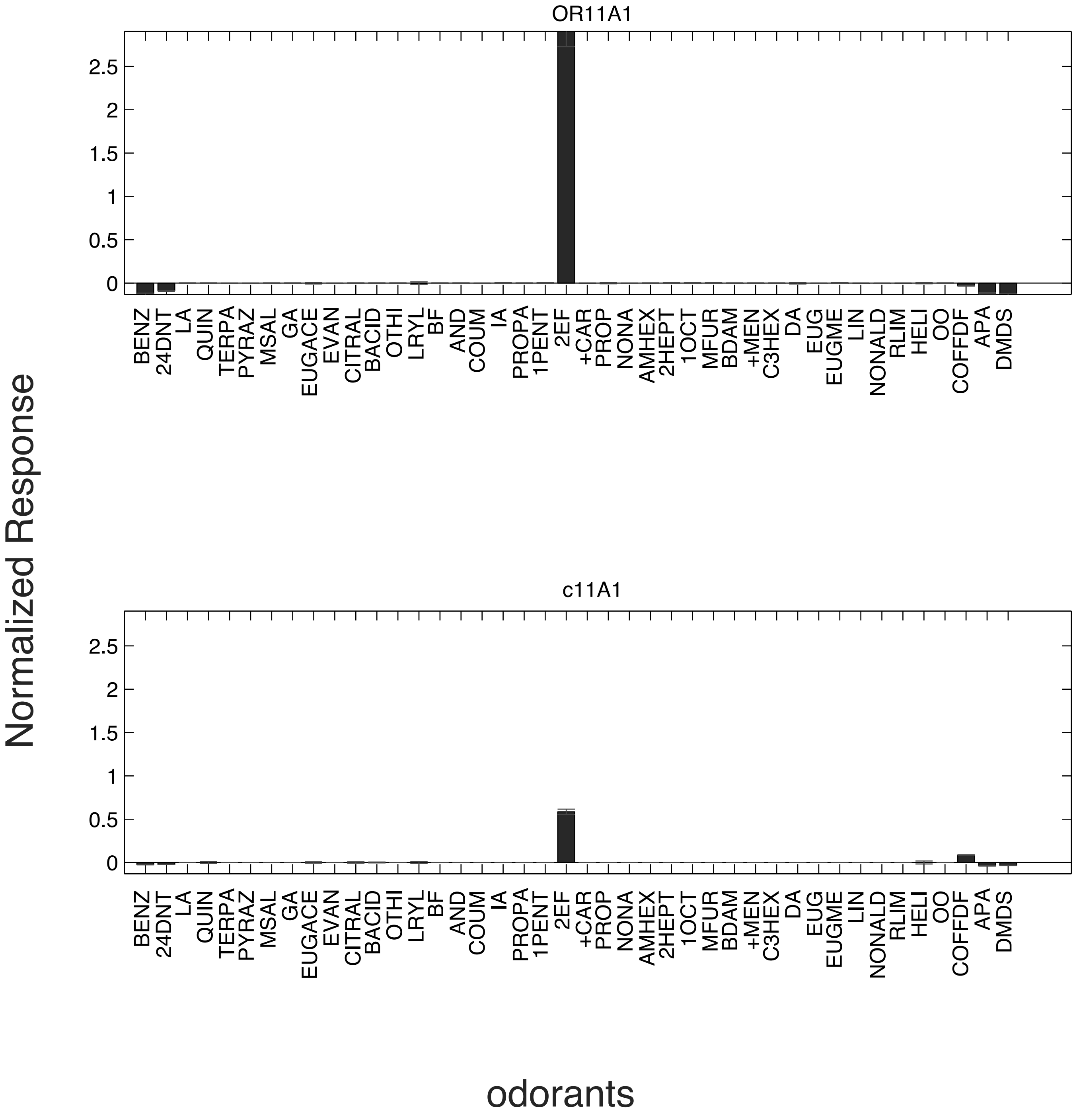
OR11A1
c11A1
Normalized Response
odorants
0
0.5
1
1.5
2
2.5
BENZ
24DNT
LA
QUIN
TERPA
PYRAZ
MSAL
GA
EUGACE
EVAN
CITRAL
BACID
OTHI
LRYL
BF
AND
COUM
IA
PROPA
1PENT
2EF
+CAR
PROP
NONA
AMHEX
2HEPT
1OCT
MFUR
BDAM
+MEN
C3HEX
DA
EUG
EUGME
LIN
NONALD
RLIM
HELI
OO
COFFDF
APA
DMDS

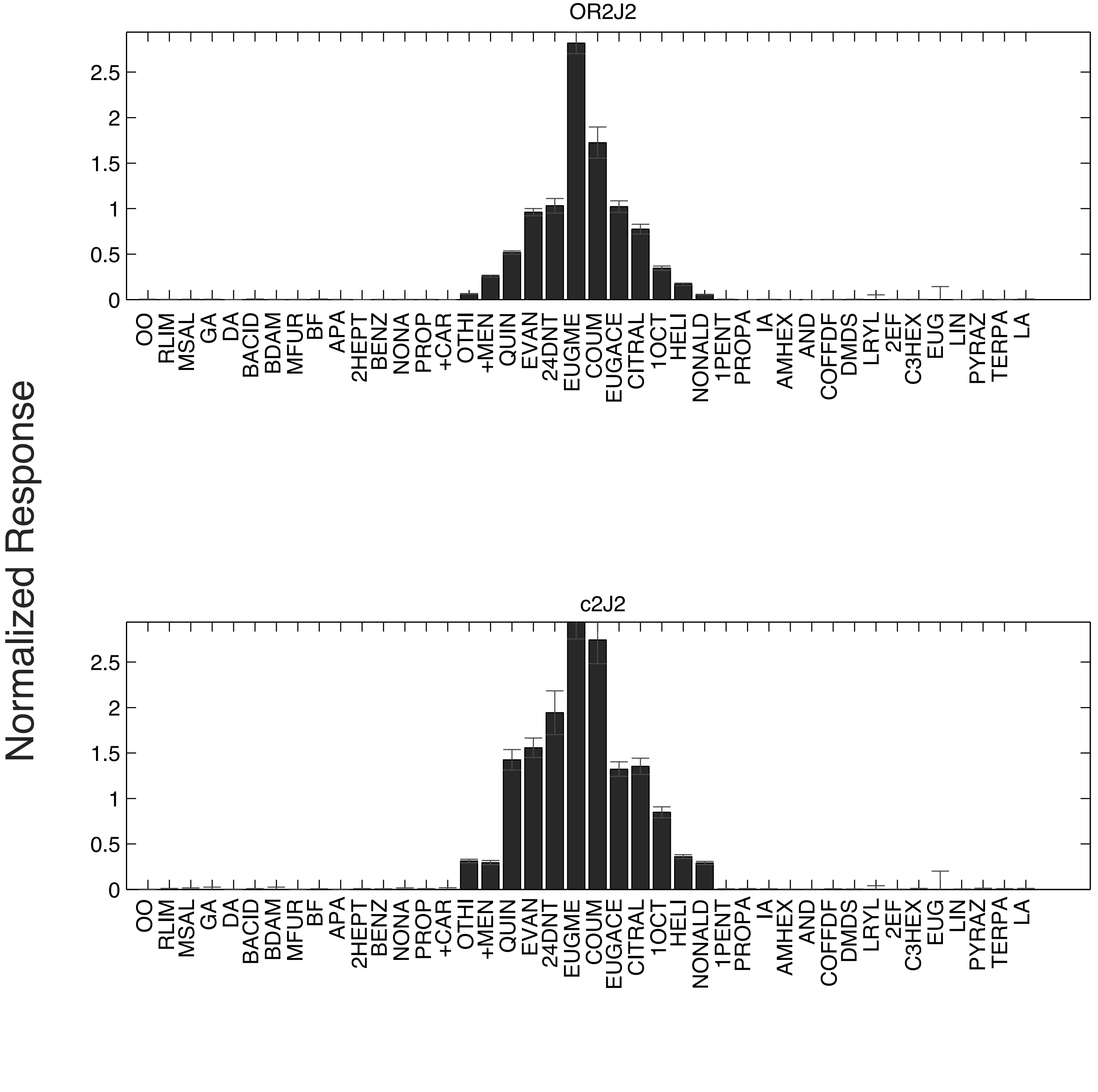
OR2J2
c2J2
Normalized Response
odorants
0
0.5
1
1.5
2
2.5
OO
RLIM
MSAL
GA
DA
BACID
BDAM
MFUR
BF
APA
2HEPT
BENZ
NONA
PROP
+CAR
OTHI
+MEN
QUIN
EVAN
24DNT
EUGME
COUM
EUGACE
CITRAL
1OCT
HELI
NONALD
1PENT
PROPA
IA
AMHEX
AND
COFFDF
DMDS
LRYL
2EF
C3HEX
EUG
LIN
PYRAZ
TERPA
LA

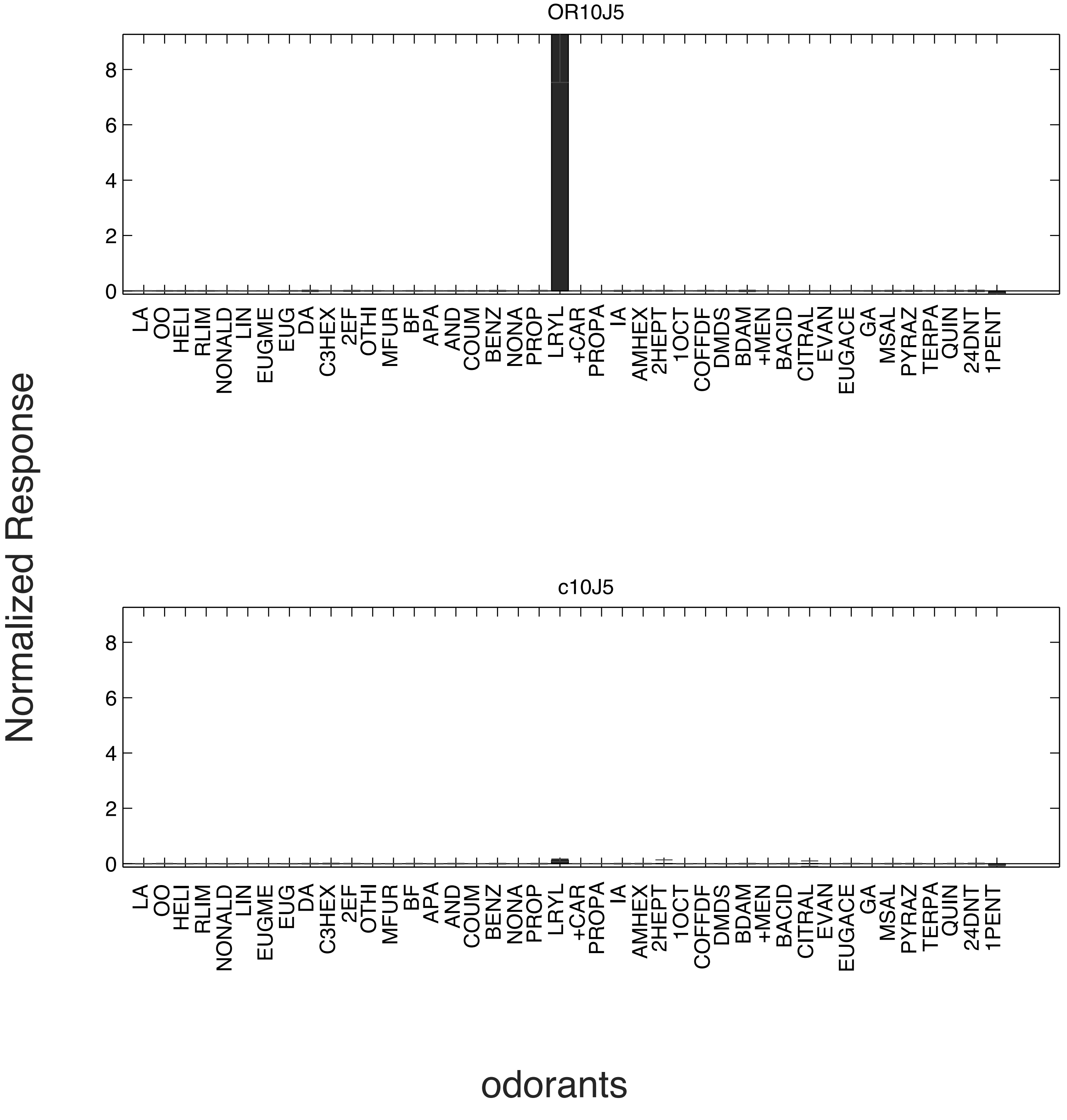
OR10J5
c10J5
Normalized Response
odorants
0
2
4
6
8
LA
OO
HELI
RLIM
NONALD
LIN
EUGME
EUG
DA
C3HEX
2EF
OTHI
MFUR
BF
APA
AND
COUM
BENZ
NONA
PROP
LRYL
+CAR
PROPA
IA
AMHEX
2HEPT
1OCT
COFFDF
DMDS
BDAM
+MEN
BACID
CITRAL
EVAN
EUGACE
GA
MSAL
PYRAZ
TERPA
QUIN
24DNT
1PENT

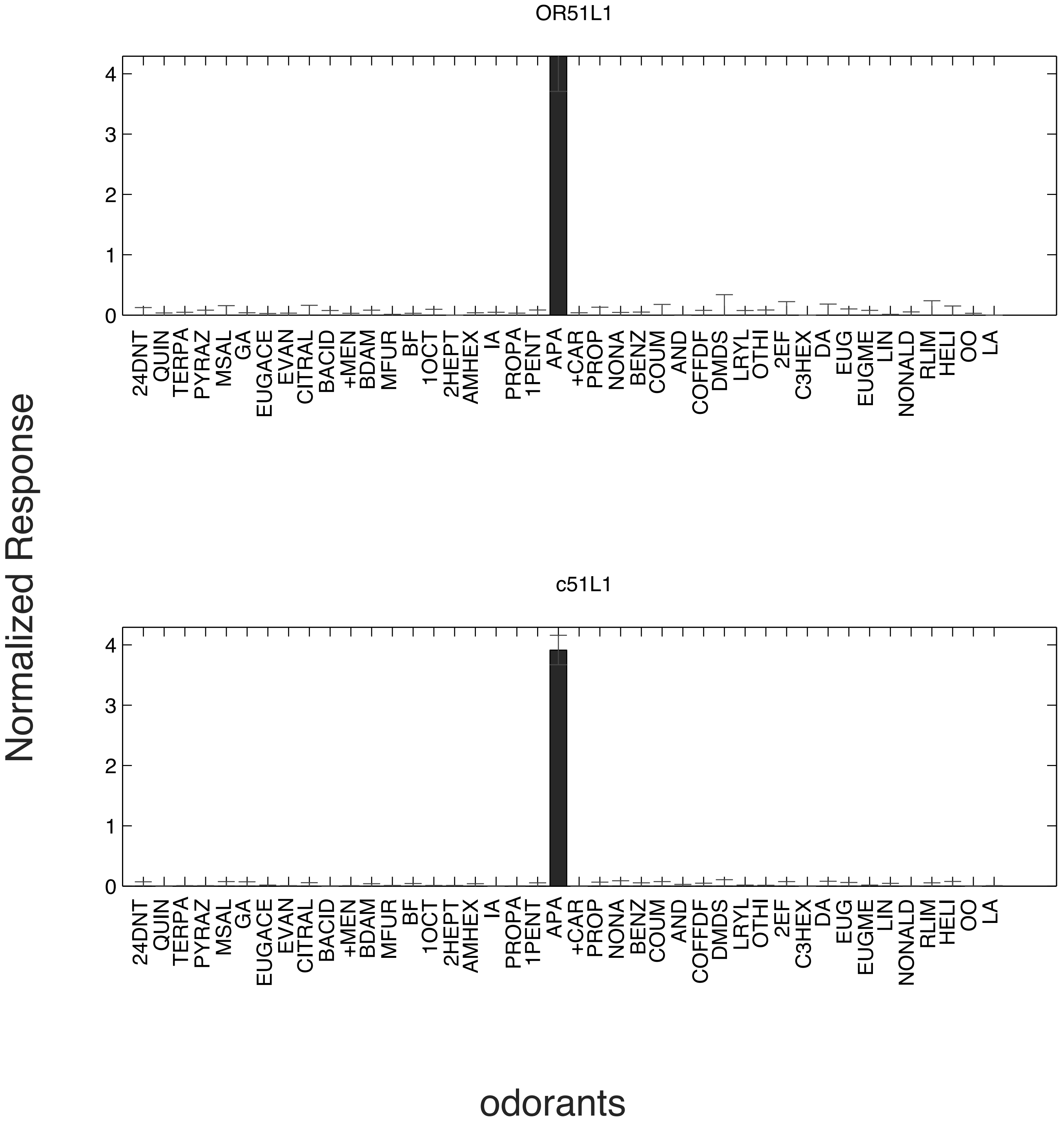

OR51L1
c51L1
Normalized Response
odorants
0
1
2
3
4
24DNT QUIN TERPA PYRAZ MSAL GA EUGACE EVAN CITRAL BACID +MEN BDAM MFUR BF 1OCT 2HEPT AMHEX IA PROPA 1PENT APA +CAR PROP NONA BENZ COUM AND COFFDF DMDS LRYL OTHI 2EF C3HEX DA EUG EUGME LIN NONALD RLIM HELI OO LA

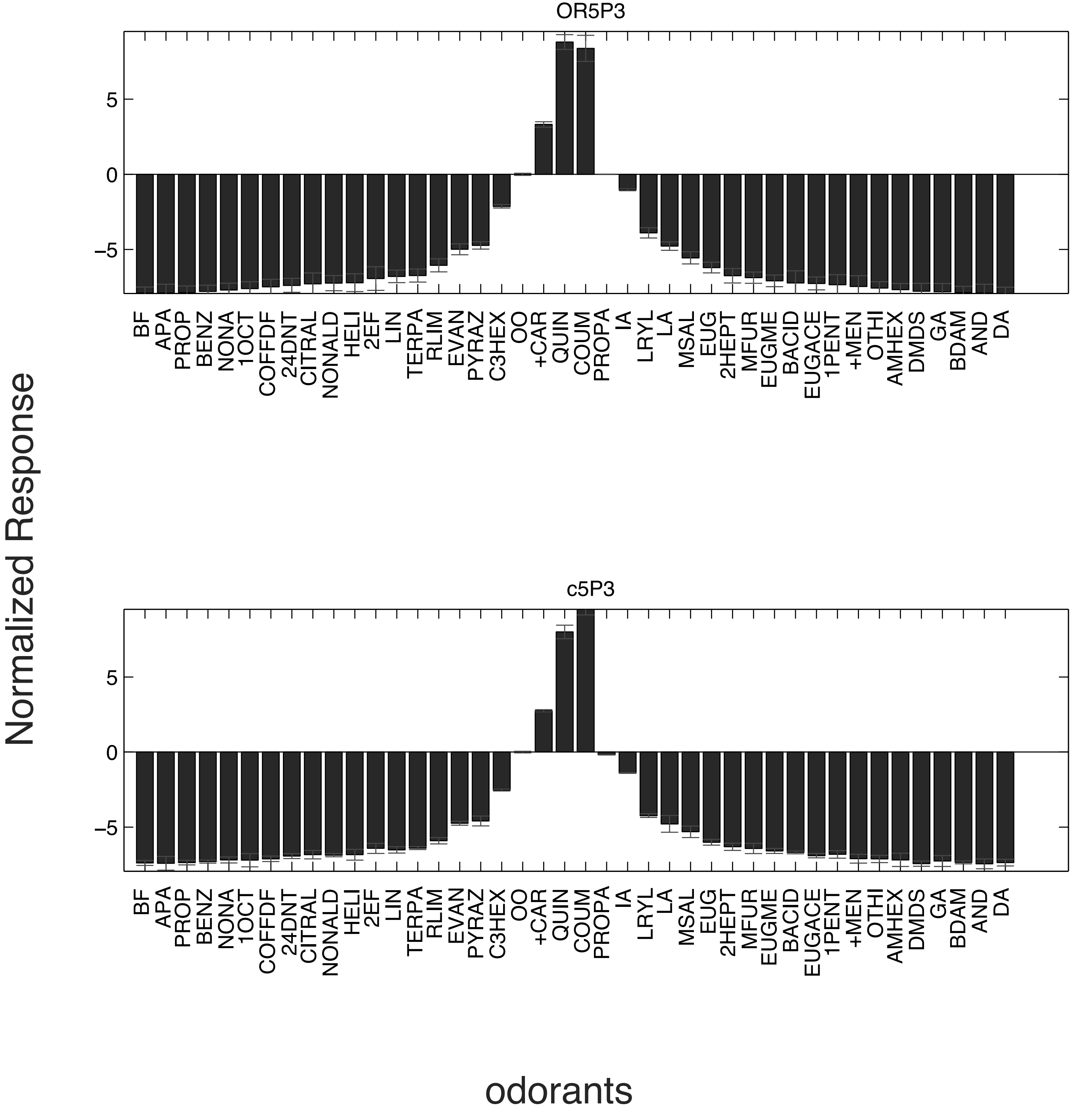
OR5P3
c5P3
Normalized Response
odorants
5
0
−5
BF
APA
PROP
BENZ
NONA
1OCT
COFFDF
24DNT
CITRAL
NONALD
HELI
2EF
LIN
TERPA
RLIM
EVAN
PYRAZ
C3HEX
OO
+CAR
QUIN
COUM
PROPA
IA
LRYL
LA
MSAL
EUG
2HEPT
MFUR
EUGME
BACID
EUGACE
1PENT
+MEN
OTHI
AMHEX
DMDS
GA
BDAM
AND
DA

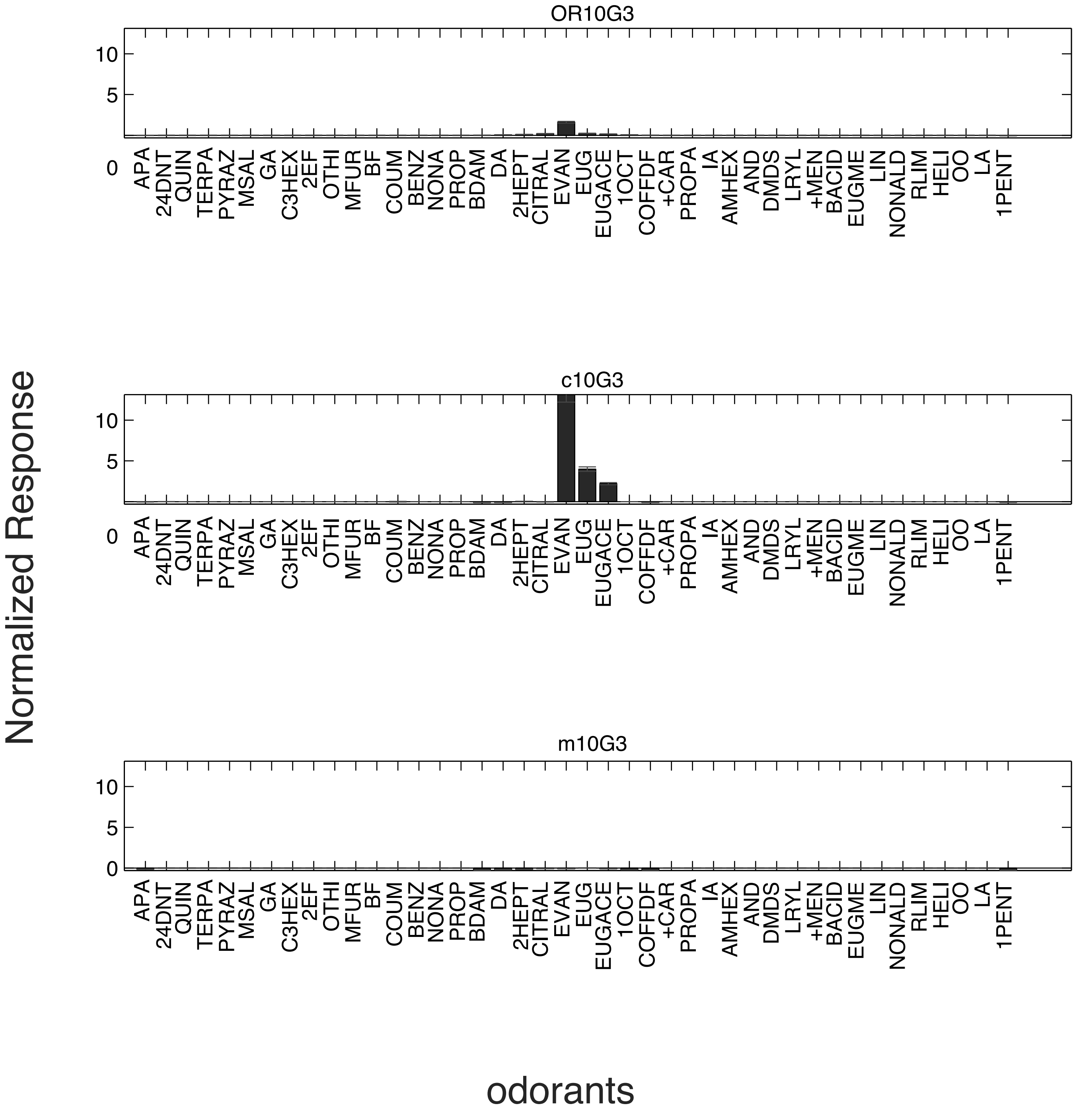
OR10G3
c10G3
m10G3
Normalized Response
odorants
0
5
10
APA
24DNT
QUIN
TERPA
PYRAZ
MSAL
GA
C3HEX
2EF
OTHI
MFUR
BF
COUM
BENZ
NONA
PROP
BDAM
DA
2HEPT
CITRAL
EVAN
EUG
EUGACE
1OCT
COFFDF
+CAR
PROPA
IA
AMHEX
AND
DMDS
LRYL
+MEN
BACID
EUGME
LIN
NONALD
RLIM
HELI
OO
LA
1PENT

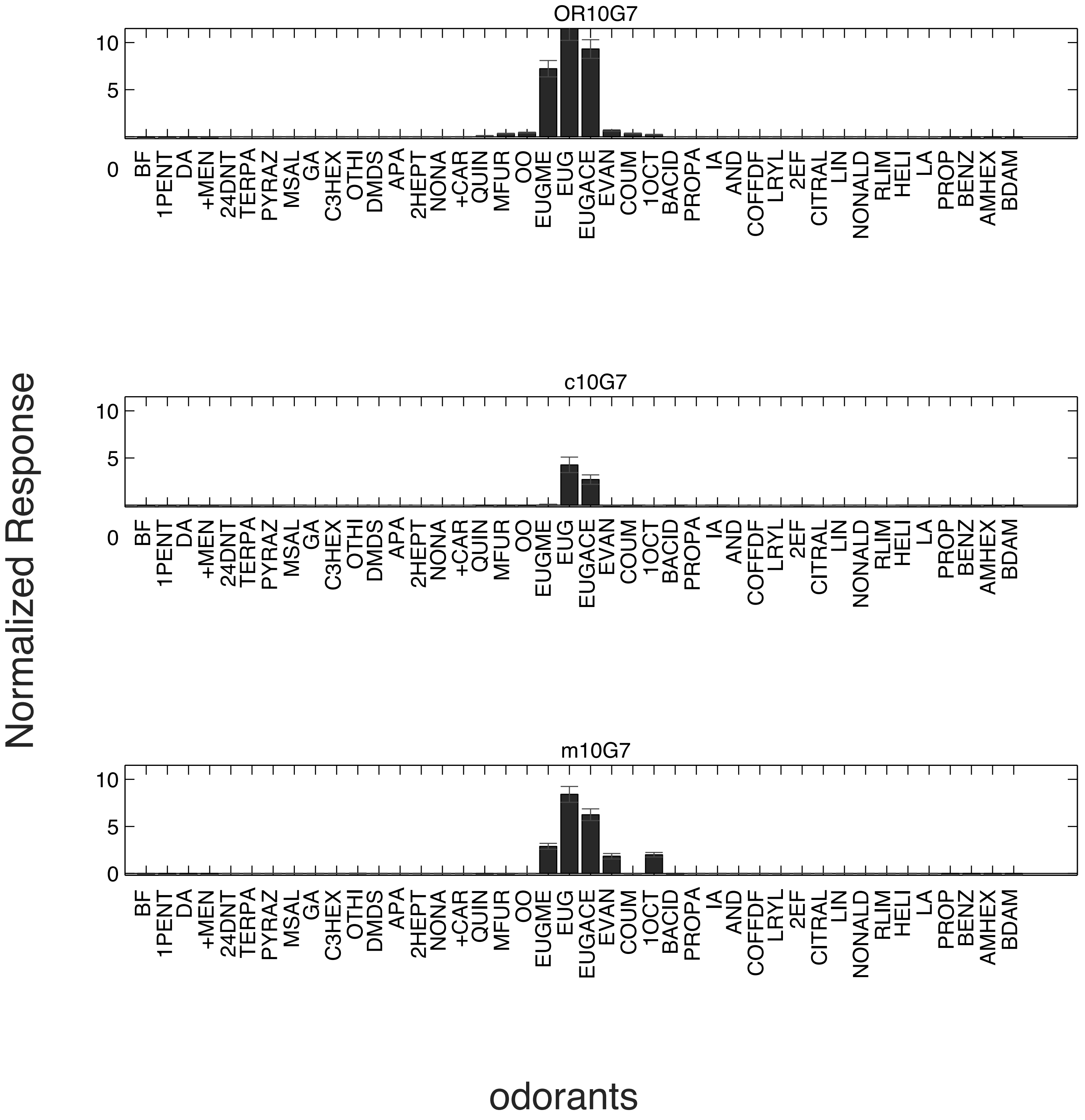

OR10G7
10
5
0
BF 1PENT DA +MEN 24DNT TERPA PYRAZ MSAL GA C3HEX OTHI DMDS APA 2HEPT NONA +CAR QUIN MFUR OO EUGME EUG EUGACE EVAN COUM 1OCT BACID PROPA IA AND COFFDF LRYL 2EF CITRAL LIN NONALD RLIM HELI LA PROP BENZ AMHEX BDAM
c10G7
10
5
0
BF 1PENT DA +MEN 24DNT TERPA PYRAZ MSAL GA C3HEX OTHI DMDS APA 2HEPT NONA +CAR QUIN MFUR OO EUGME EUG EUGACE EVAN COUM 1OCT BACID PROPA IA AND COFFDF LRYL 2EF CITRAL LIN NONALD RLIM HELI LA PROP BENZ AMHEX BDAM
m10G7
10
5
0
BF 1PENT DA +MEN 24DNT TERPA PYRAZ MSAL GA C3HEX OTHI DMDS APA 2HEPT NONA +CAR QUIN MFUR OO EUGME EUG EUGACE EVAN COUM 1OCT BACID PROPA IA AND COFFDF LRYL 2EF CITRAL LIN NONALD RLIM HELI LA PROP BENZ AMHEX BDAM
Normalized Response
odorants

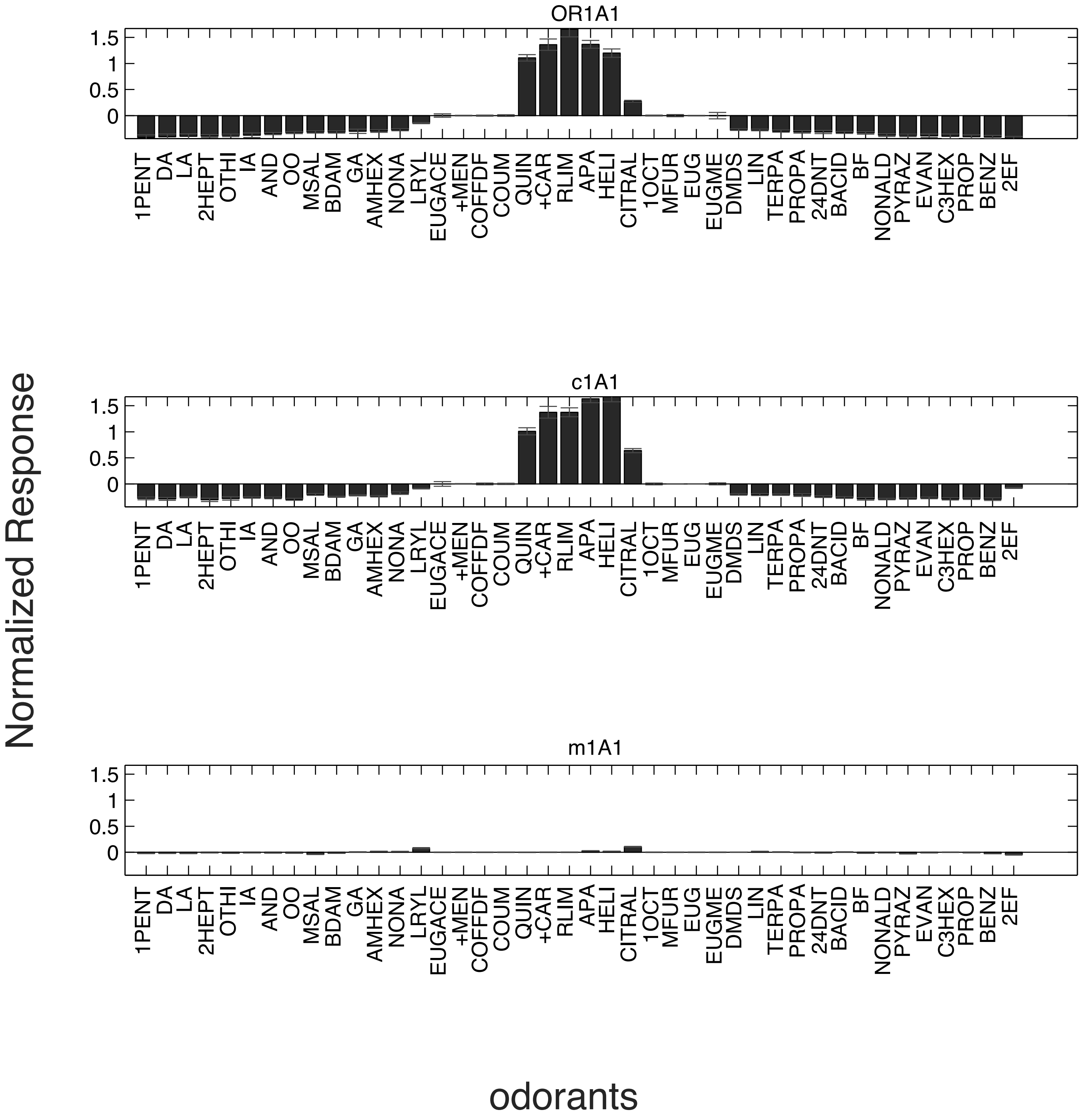
OR1A1
c1A1
m1A1
1.5
1
0.5
0
1PENT DA LA 2HEPT OTHI IA AND OO MSAL BDAM GA AMHEX NONA LRYL EUGACE +MEN COFFDF COUM QUIN +CAR RLIM APA HELI CITRAL 1OCT MFUR EUG EUGME DMDS LIN TERPA PROPA 24DNT BACID BF NONALD PYRAZ EVAN C3HEX PROP BENZ 2EF
Normalized Response
odorants

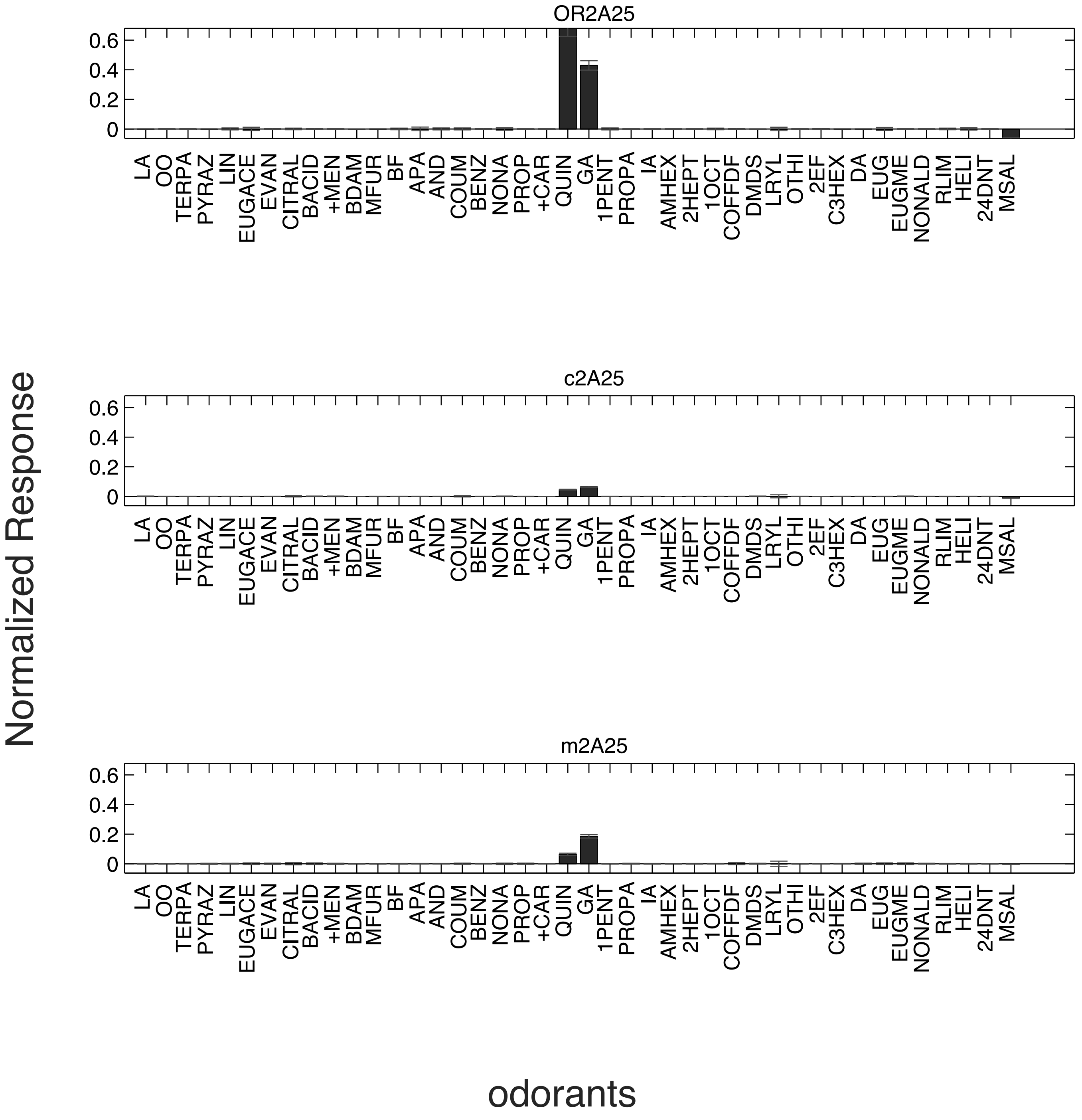
OR2A25
c2A25
m2A25
0.6
0.4
0.2
0
LA OO TERPA PYRAZ LIN EUGACE EVAN CITRAL BACID +MEN BDAM MFUR BF APA AND COUM BENZ NONA PROP +CAR QUIN GA 1PENT PROPA IA AMHEX 2HEPT 1OCT COFFDF DMDS LRYL OTHI 2EF C3HEX DA EUG EUGME NONALD RLIM HELI 24DNT MSAL
Normalized Response
odorants

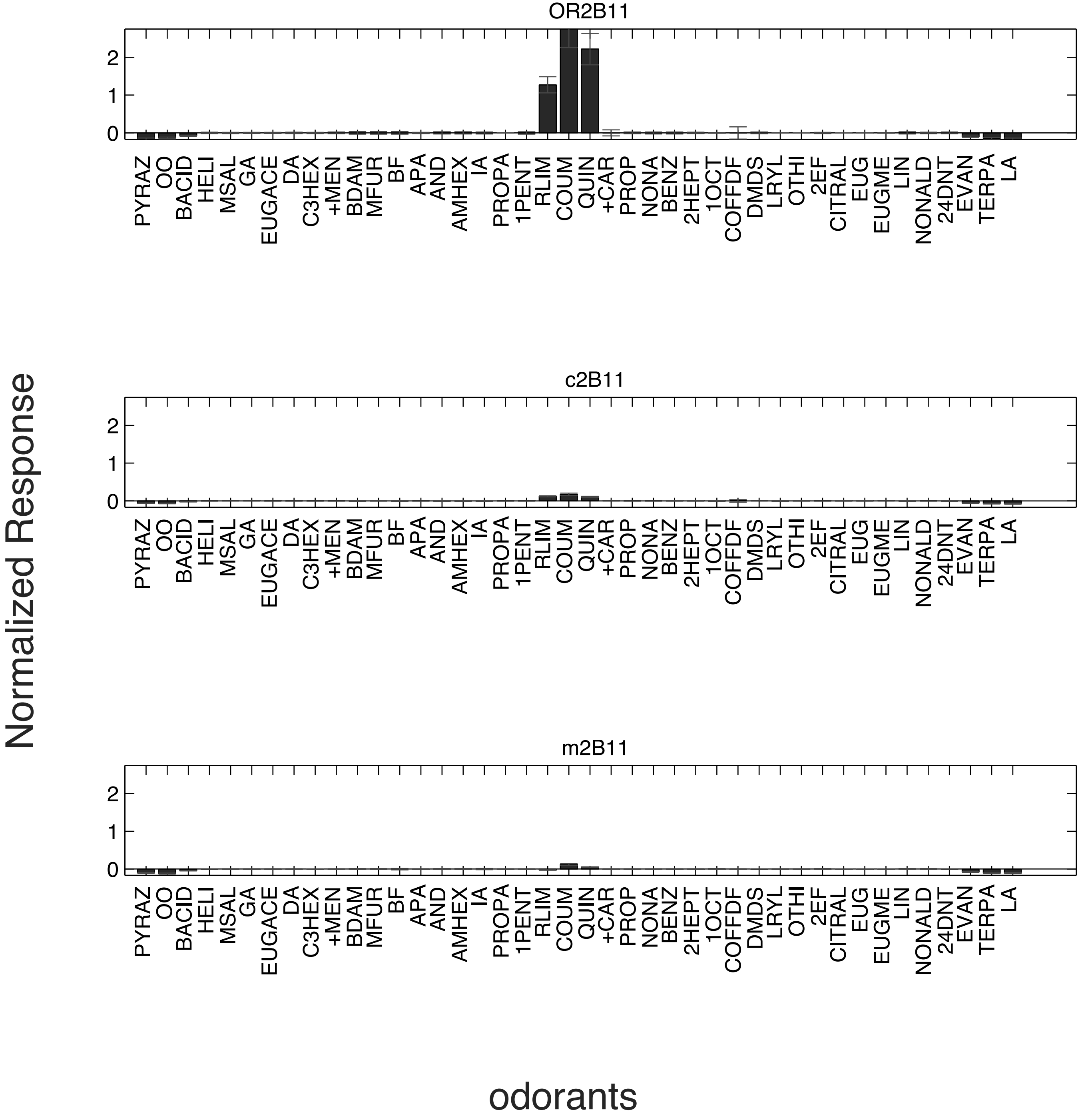
OR2B11
c2B11
m2B11
Normalized Response
odorants
0
1
2
PYRAZ
OO
BACID
HELI
MSAL
GA
EUGACE
DA
C3HEX
+MEN
BDAM
MFUR
BF
APA
AND
AMHEX
IA
PROPA
1PENT
RLIM
COUM
QUIN
+CAR
PROP
NONA
BENZ
2HEPT
1OCT
COFFDF
DMDS
LRYL
OTHI
2EF
CITRAL
EUG
EUGME
LIN
NONALD
24DNT
EVAN
TERPA
LA

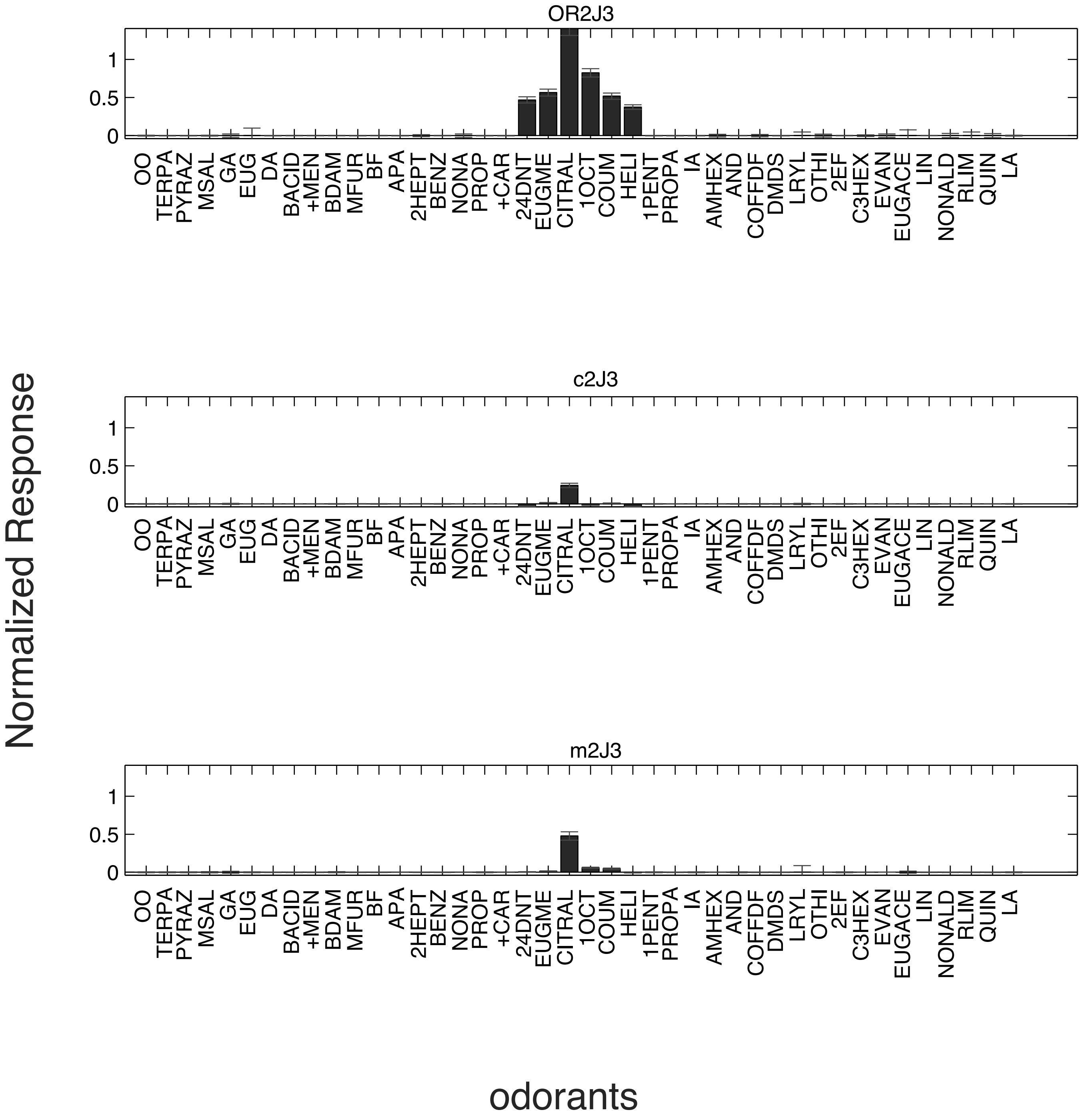
OR2J3
c2J3
m2J3
Normalized Response
odorants
0
0.5
1
OO
TERPA
PYRAZ
MSAL
GA
EUG
DA
BACID
+MEN
BDAM
MFUR
BF
APA
2HEPT
BENZ
NONA
PROP
+CAR
24DNT
EUGME
CITRAL
1OCT
COUM
HELI
1PENT
PROPA
IA
AMHEX
AND
COFFDF
DMDS
LRYL
OTHI
2EF
C3HEX
EVAN
EUGACE
LIN
NONALD
RLIM
QUIN
LA

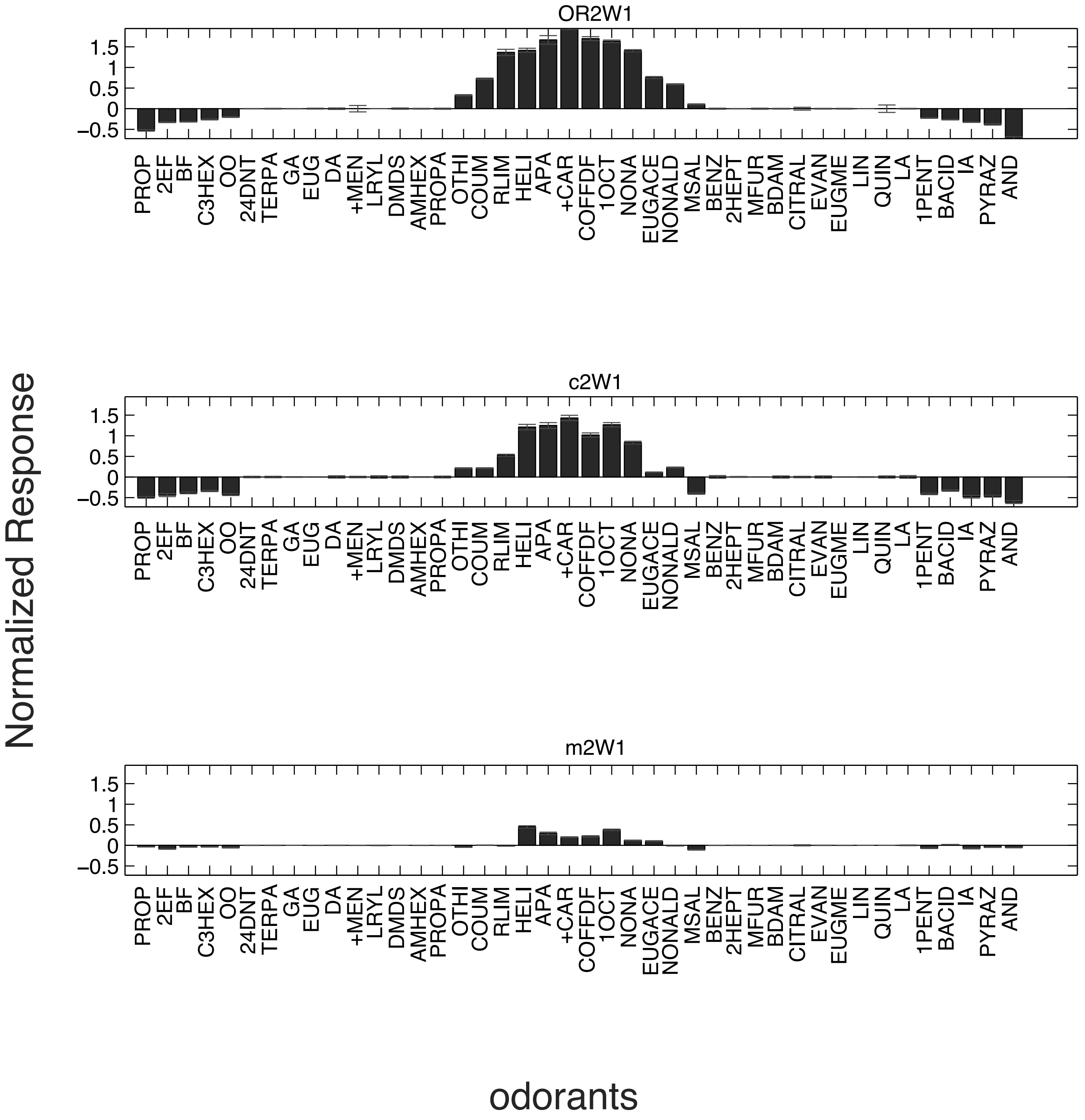

OR2W1
c2W1
m2W1
1.5
1
0.5
0
−0.5
PROP 2EF BF C3HEX OO 24DNT TERPA GA EUG DA +MEN LRYL DMDS AMHEX PROPA OTHI COUM RLIM HELI APA +CAR COFFDF 1OCT NONA EUGACE NONALD MSAL BENZ 2HEPT MFUR BDAM CITRAL EVAN EUGME LIN QUIN LA 1PENT BACID IA PYRAZ AND
Normalized Response
odorants

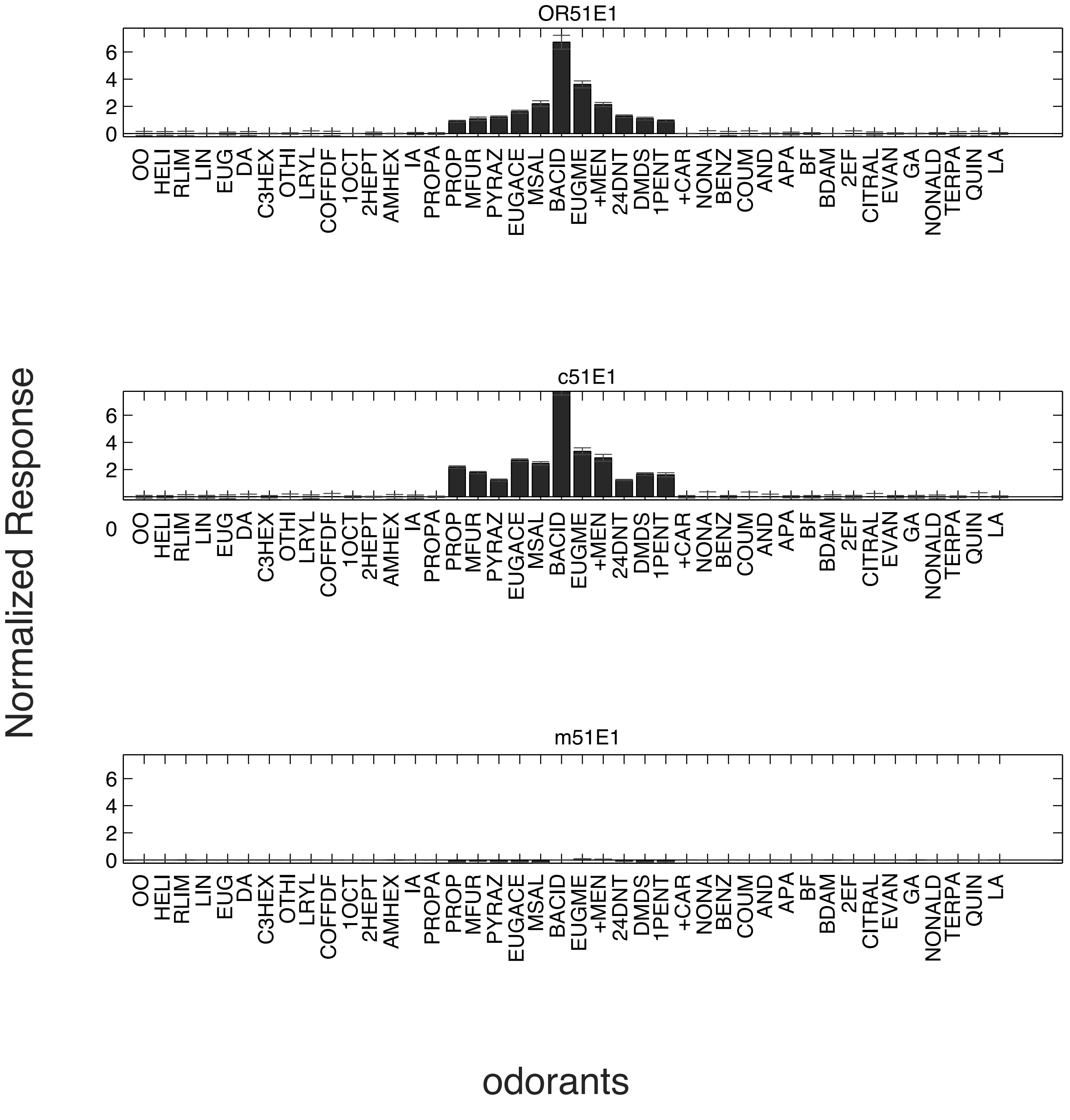
OR51E1
c51E1
m51E1
Normalized Response
odorants
0
2
4
6
OO HELI RLIM LIN EUG DA C3HEX OTHI LRYL COFFDF 1OCT 2HEPT AMHEX IA PROPA PROP MFUR PYRAZ EUGACE MSAL BACID EUGME +MEN 24DNT DMDS 1PENT +CAR NONA BENZ COUM AND APA BF BDAM 2EF CITRAL EVAN GA NONALD TERPA QUIN LA

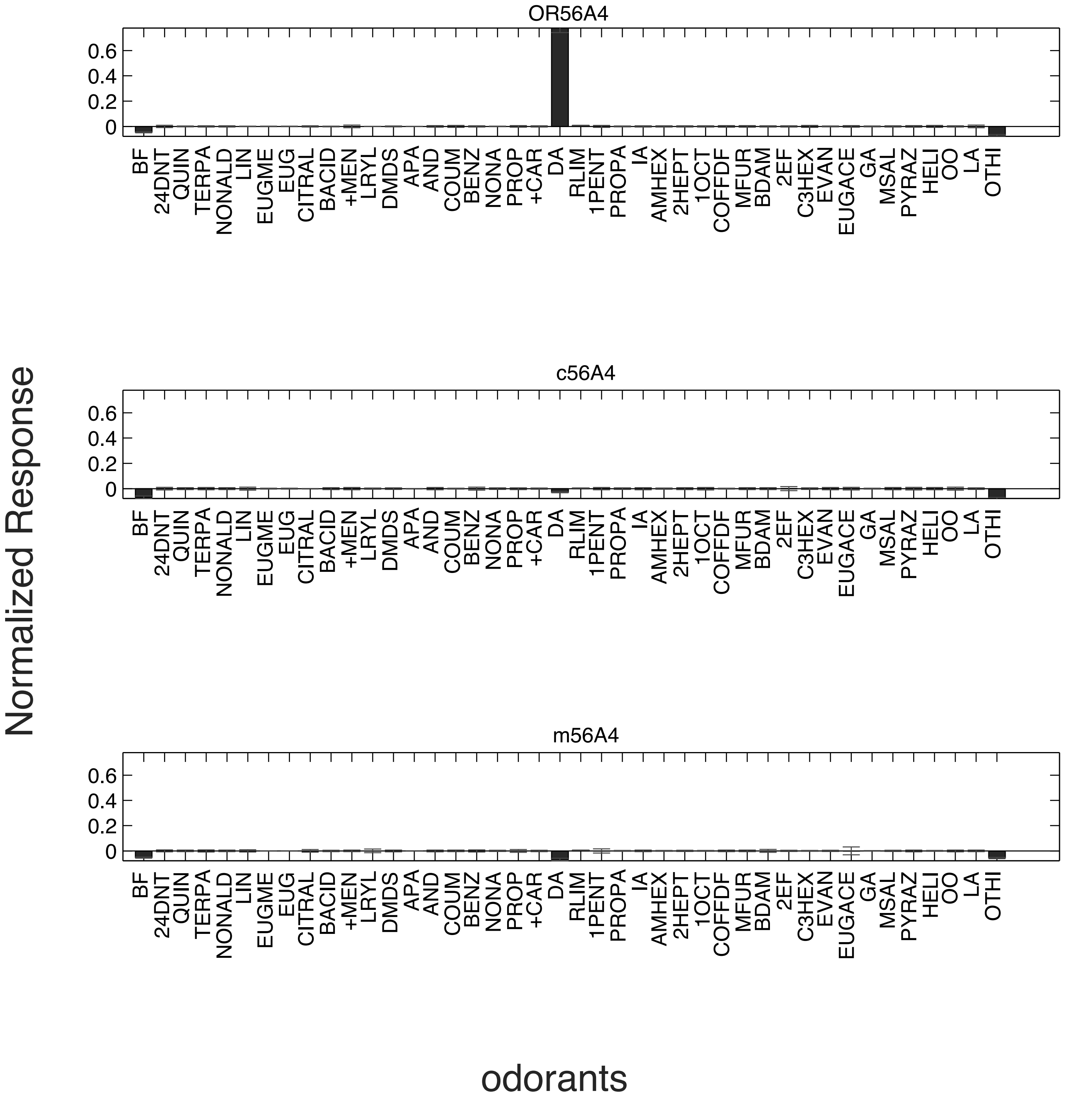
OR56A4
0.6
0.4
0.2
0
BF 24DNT QUIN TERPA NONALD LIN EUGME EUG CITRAL BACID +MEN LRYL DMDS APA AND COUM BENZ NONA PROP +CAR DA RLIM 1PENT PROPA IA AMHEX 2HEPT 1OCT COFFDF MFUR BDAM 2EF C3HEX EVAN EUGACE GA MSAL PYRAZ HELI OO LA OTHI
c56A4
0.6
0.4
0.2
0
BF 24DNT QUIN TERPA NONALD LIN EUGME EUG CITRAL BACID +MEN LRYL DMDS APA AND COUM BENZ NONA PROP +CAR DA RLIM 1PENT PROPA IA AMHEX 2HEPT 1OCT COFFDF MFUR BDAM 2EF C3HEX EVAN EUGACE GA MSAL PYRAZ HELI OO LA OTHI
m56A4
0.6
0.4
0.2
0
BF 24DNT QUIN TERPA NONALD LIN EUGME EUG CITRAL BACID +MEN LRYL DMDS APA AND COUM BENZ NONA PROP +CAR DA RLIM 1PENT PROPA IA AMHEX 2HEPT 1OCT COFFDF MFUR BDAM 2EF C3HEX EVAN EUGACE GA MSAL PYRAZ HELI OO LA OTHI
Normalized Response
odorants

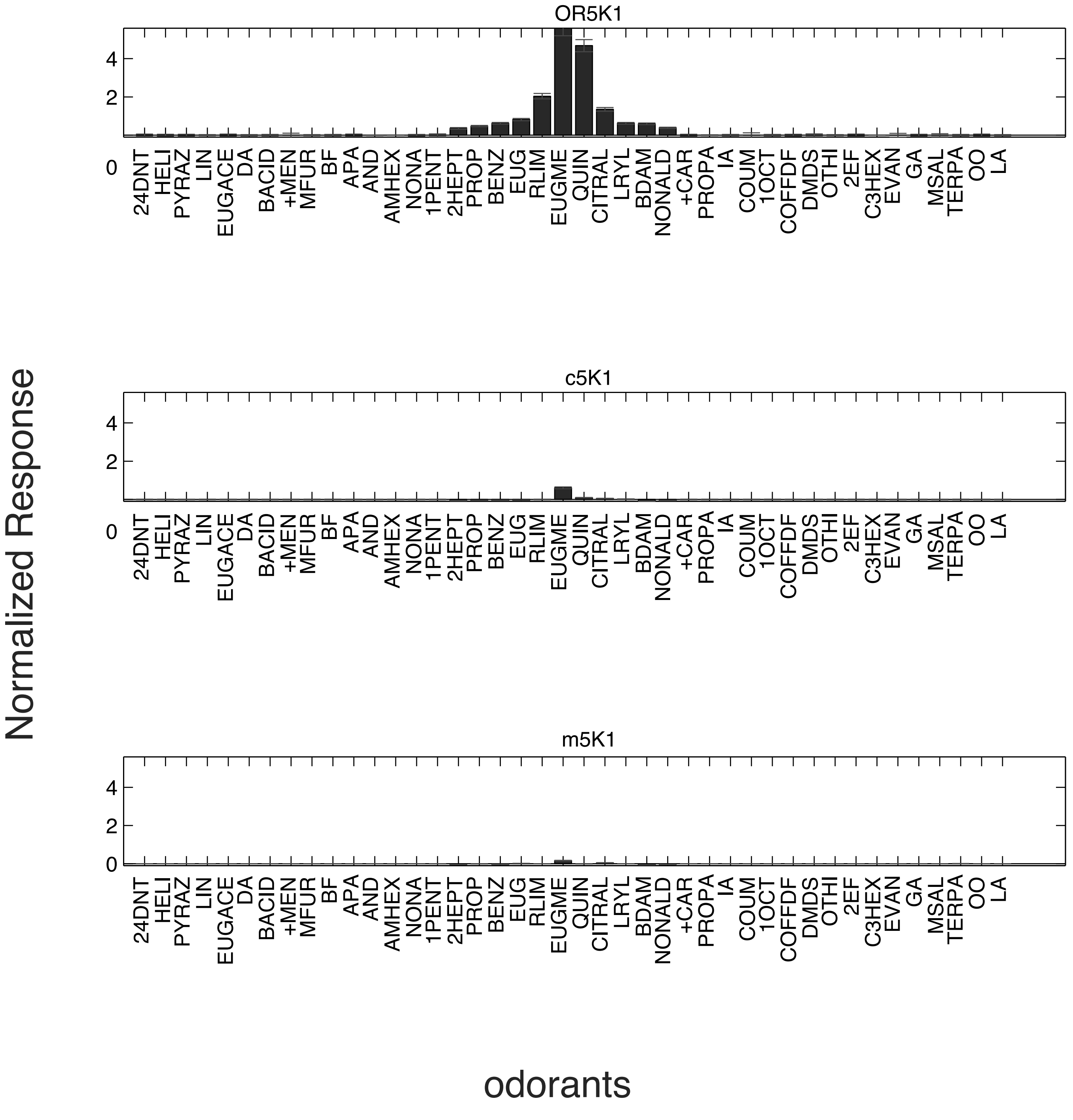
OR5K1
c5K1
m5K1
0
2
4
24DNT HELI PYRAZ LIN EUGACE DA BACID +MEN MFUR BF APA AND AMHEX NONA 1PENT 2HEPT PROP BENZ EUG RLIM EUGME QUIN CITRAL LRYL BDAM NONALD +CAR PROPA IA COUM 1OCT COFFDF DMDS OTHI 2EF C3HEX EVAN GA MSAL TERPA OO LA
Normalized Response
odorants

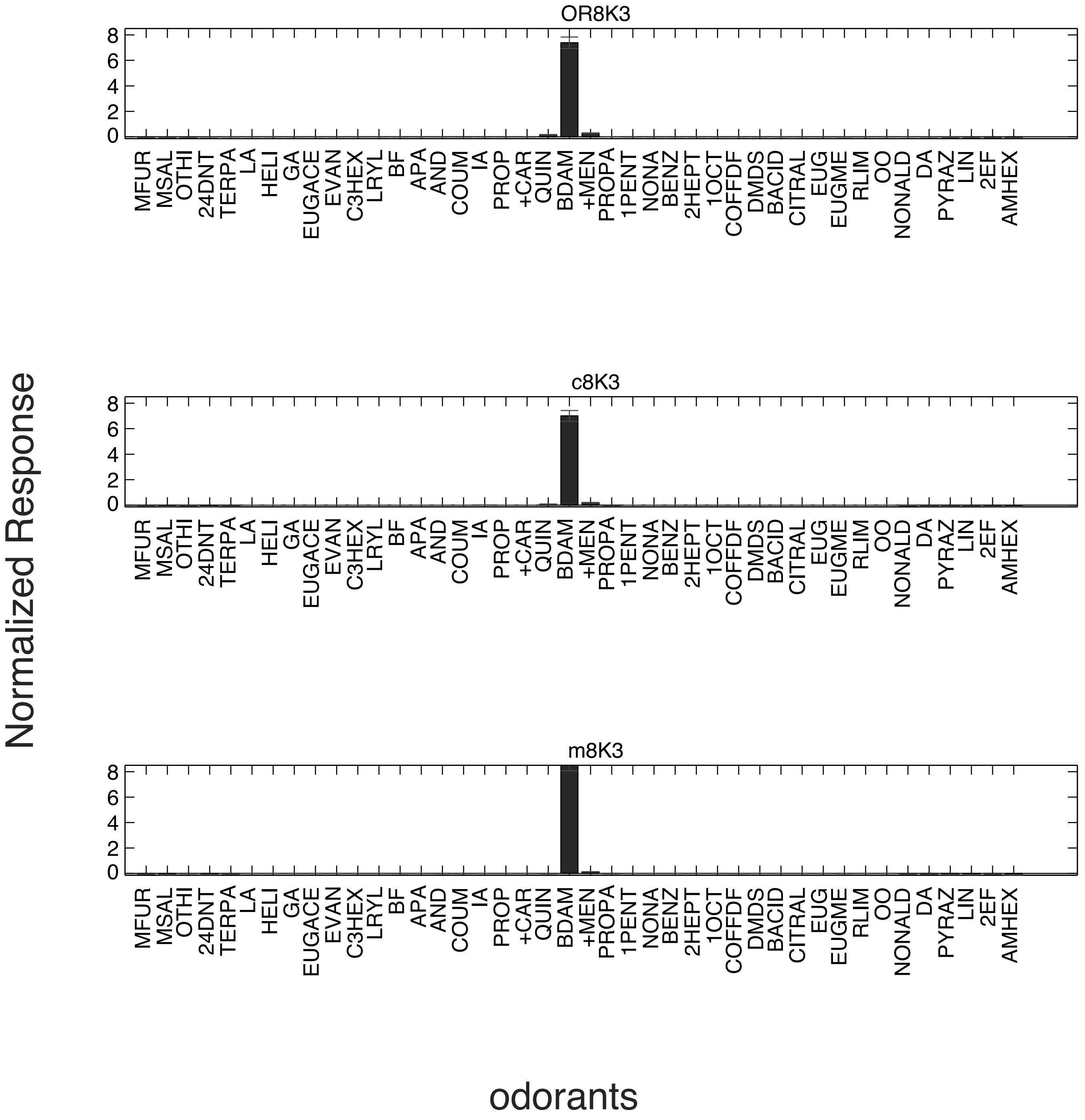
OR8K3
c8K3
m8K3
Normalized Response
odorants
MFUR MSAL OTHI 24DNT TERPA LA HELI GA EUGACE EVAN C3HEX LRYL BF APA AND COUM IA PROP +CAR QUIN BDAM +MEN PROPA 1PENT NONA BENZ 2HEPT 1OCT COFFDF DMDS BACID CITRAL EUG EUGME RLIM OO NONALD DA PYRAZ LIN 2EF AMHEX
0
2
4
6
8

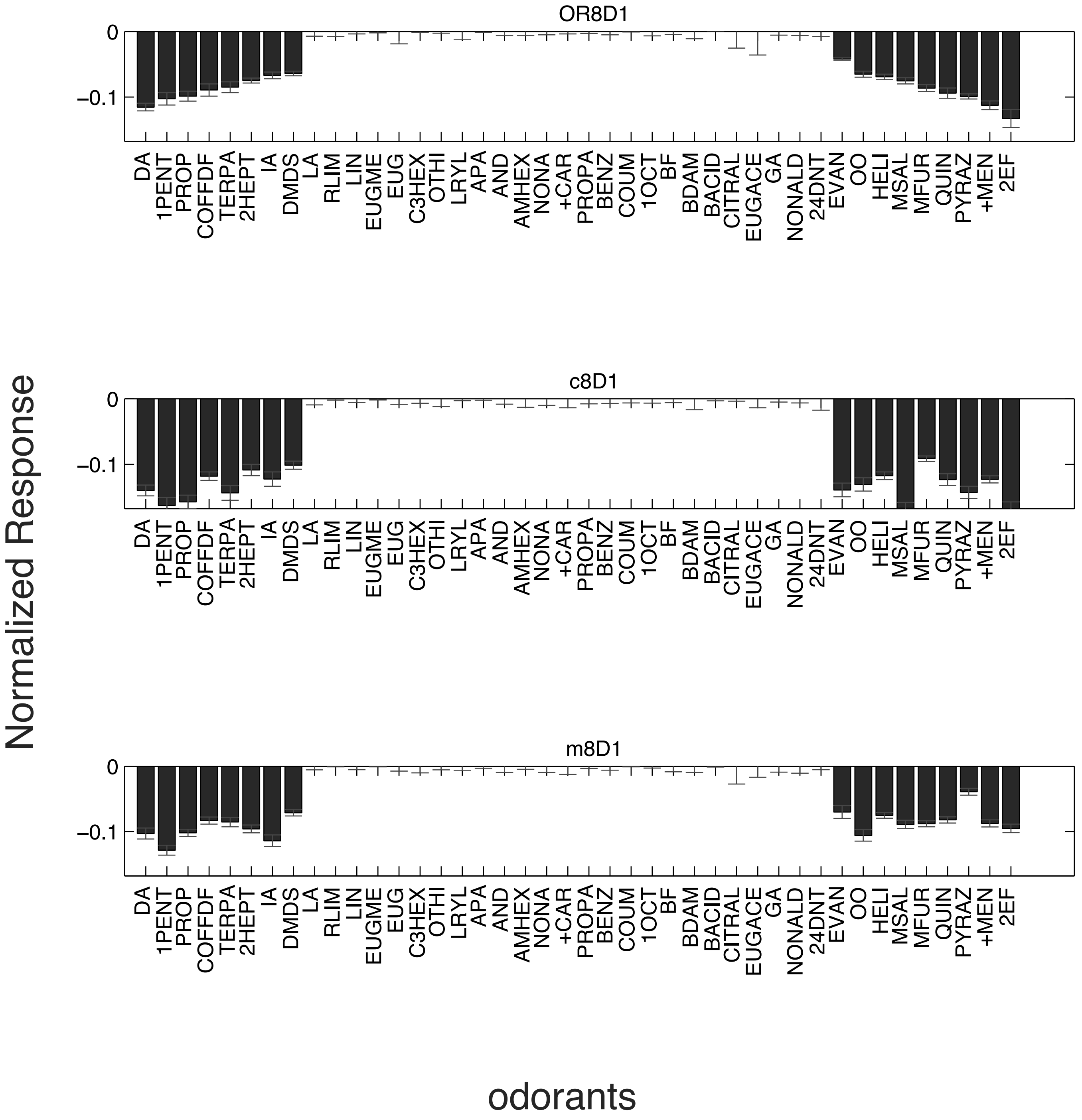

OR8D1
0
−0.1
DA 1PENT PROP COFFDF TERPA 2HEPT IA DMDS LA RLIM LIN EUGME EUG C3HEX OTHI LRYL APA AND AMHEX NONA +CAR PROPA BENZ COUM 1OCT BF BDAM BACID CITRAL EUGACE GA NONALD 24DNT EVAN OO HELI MSAL MFUR QUIN PYRAZ +MEN 2EF
c8D1
0
−0.1
DA 1PENT PROP COFFDF TERPA 2HEPT IA DMDS LA RLIM LIN EUGME EUG C3HEX OTHI LRYL APA AND AMHEX NONA +CAR PROPA BENZ COUM 1OCT BF BDAM BACID CITRAL EUGACE GA NONALD 24DNT EVAN OO HELI MSAL MFUR QUIN PYRAZ +MEN 2EF
m8D1
0
−0.1
DA 1PENT PROP COFFDF TERPA 2HEPT IA DMDS LA RLIM LIN EUGME EUG C3HEX OTHI LRYL APA AND AMHEX NONA +CAR PROPA BENZ COUM 1OCT BF BDAM BACID CITRAL EUGACE GA NONALD 24DNT EVAN OO HELI MSAL MFUR QUIN PYRAZ +MEN 2EF
Normalized Response
odorants

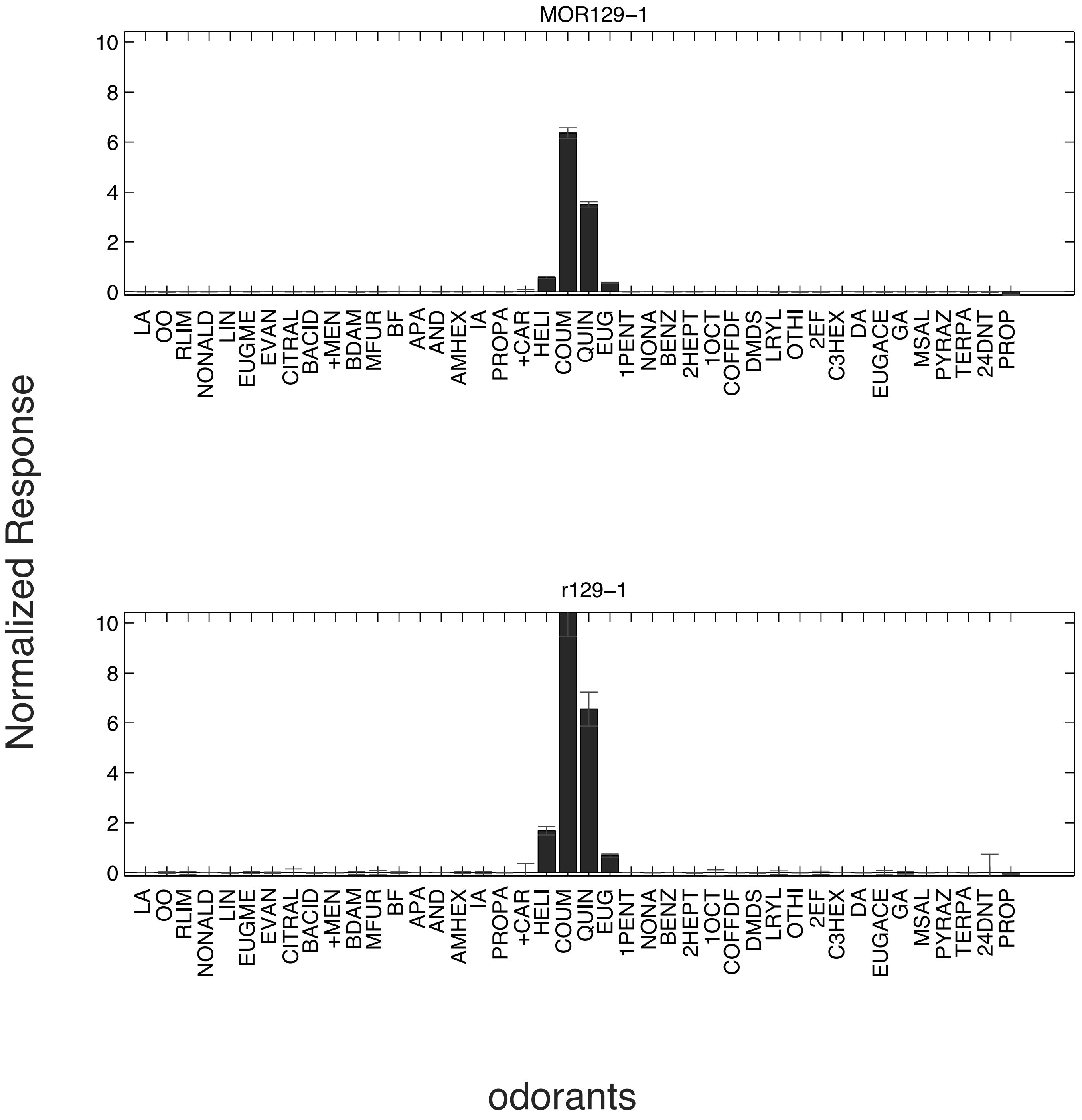
MOR129-1
r129-1
Normalized Response
odorants
LA
OO
RLIM
NONALD
LIN
EUGME
EVAN
CITRAL
BACID
+MEN
BDAM
MFUR
BF
APA
AND
AMHEX
IA
PROPA
+CAR
HELI
COUM
QUIN
EUG
1PENT
NONA
BENZ
2HEPT
1OCT
COFFDF
DMDS
LRYL
OTHI
2EF
C3HEX
DA
EUGACE
GA
MSAL
PYRAZ
TERPA
24DNT
PROP

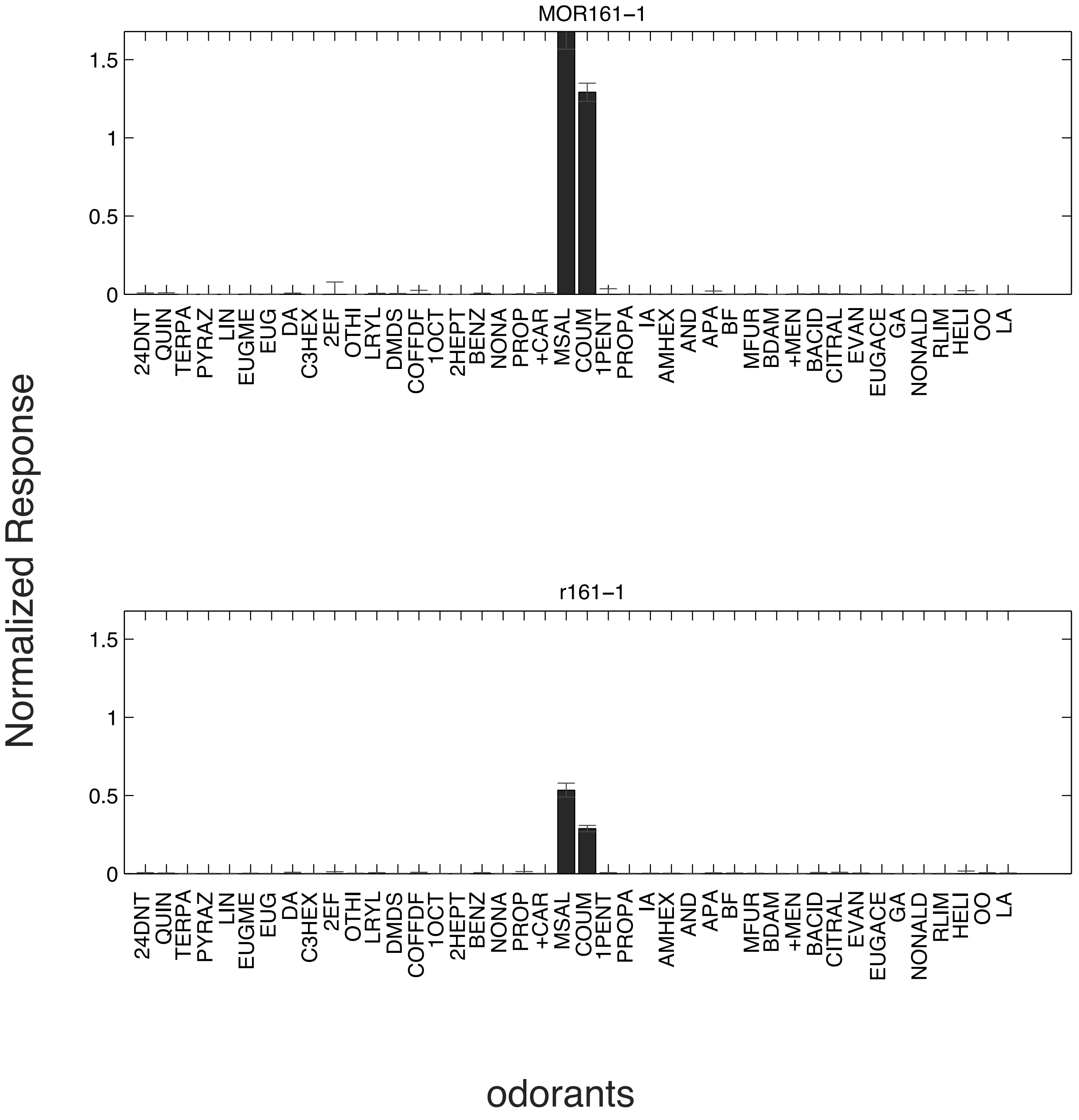
MOR161-1
r161-1
Normalized Response
odorants
0
0.5
1
1.5
24DNT
QUIN
TERPA
PYRAZ
LIN
EUGME
EUG
DA
C3HEX
2EF
OTHI
LRYL
DMDS
COFFDF
1OCT
2HEPT
BENZ
NONA
PROP
+CAR
MSAL
COUM
1PENT
PROPA
IA
AMHEX
AND
APA
BF
MFUR
BDAM
+MEN
BACID
CITRAL
EVAN
EUGACE
GA
NONALD
RLIM
HELI
OO
LA

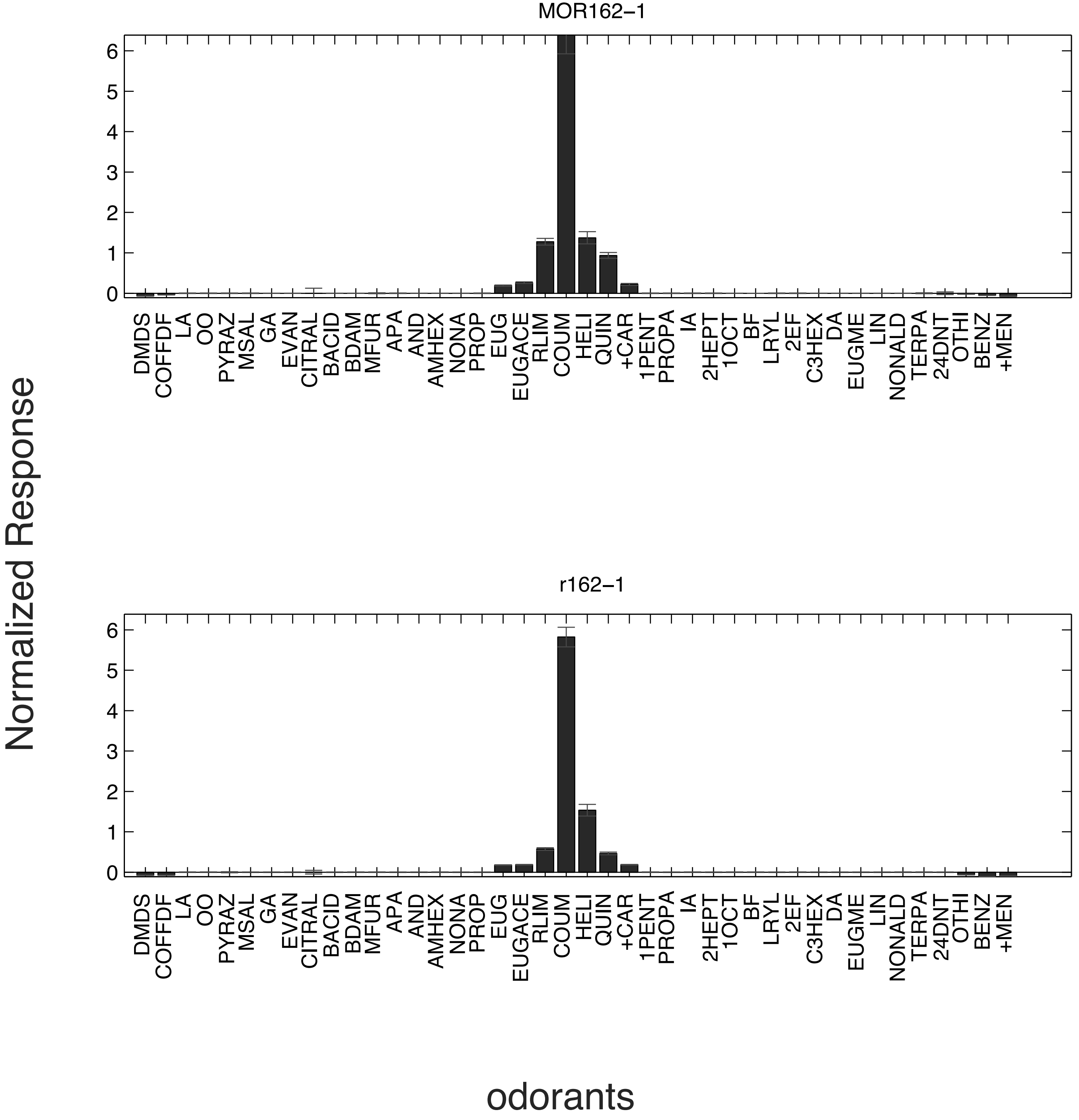

MOR162-1
r162-1
Normalized Response
odorants
0
1
2
3
4
5
6
DMDS
COFFDF
LA
OO
PYRAZ
MSAL
GA
EVAN
CITRAL
BACID
BDAM
MFUR
APA
AND
AMHEX
NONA
PROP
EUG
EUGACE
RLIM
COUM
HELI
QUIN
+CAR
1PENT
PROPA
IA
2HEPT
1OCT
BF
LRYL
2EF
C3HEX
DA
EUGME
LIN
NONALD
TERPA
24DNT
OTHI
BENZ
+MEN

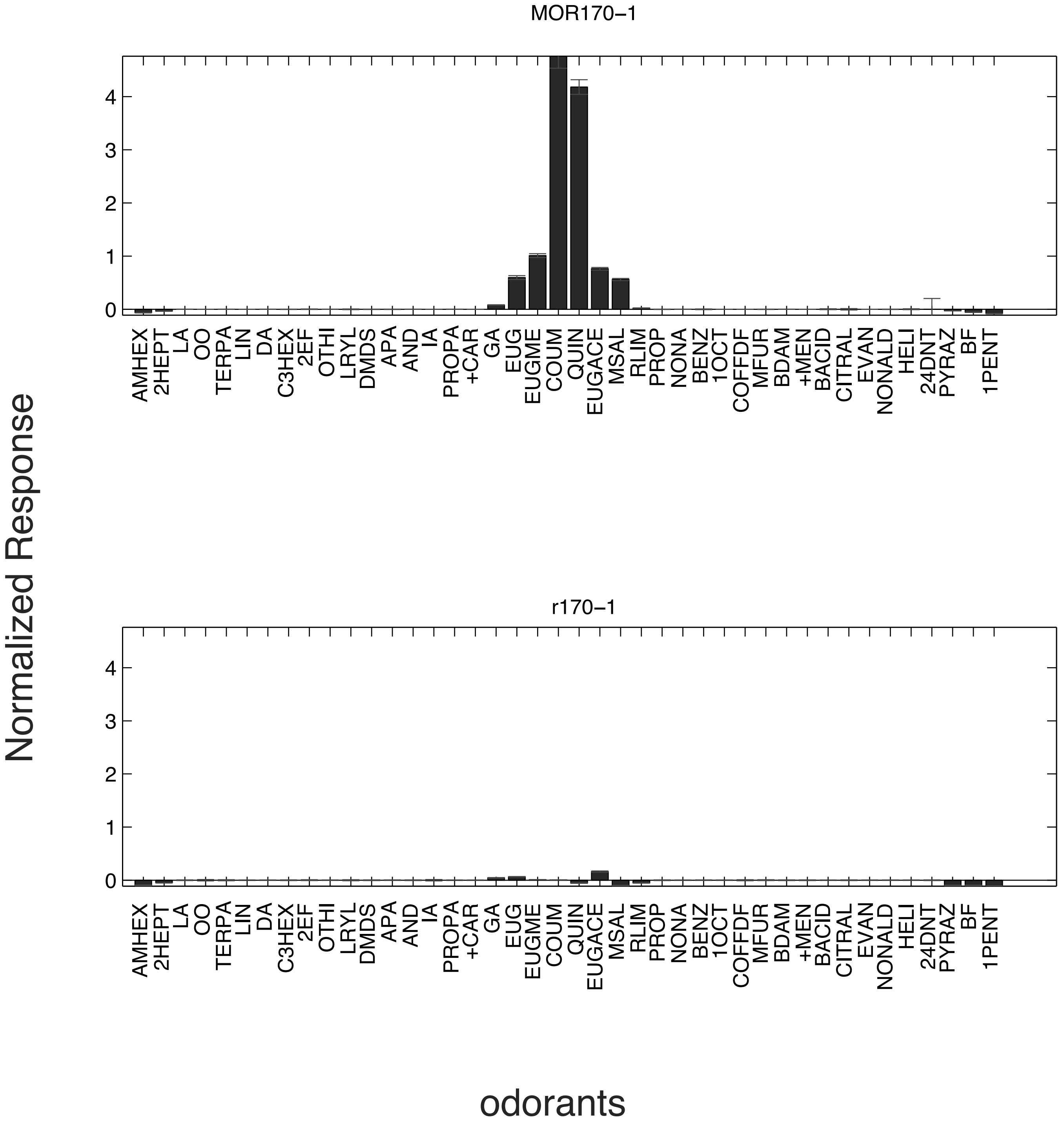
MOR170-1
r170-1
Normalized Response
odorants
0
1
2
3
4
AMHEX
2HEPT
LA
OO
TERPA
LIN
DA
C3HEX
2EF
OTHI
LRYL
DMDS
APA
AND
IA
PROPA
+CAR
GA
EUG
EUGME
COUM
QUIN
EUGACE
MSAL
RLIM
PROP
NONA
BENZ
1OCT
COFFDF
MFUR
BDAM
+MEN
BACID
CITRAL
EVAN
NONALD
HELI
24DNT
PYRAZ
BF
1PENT

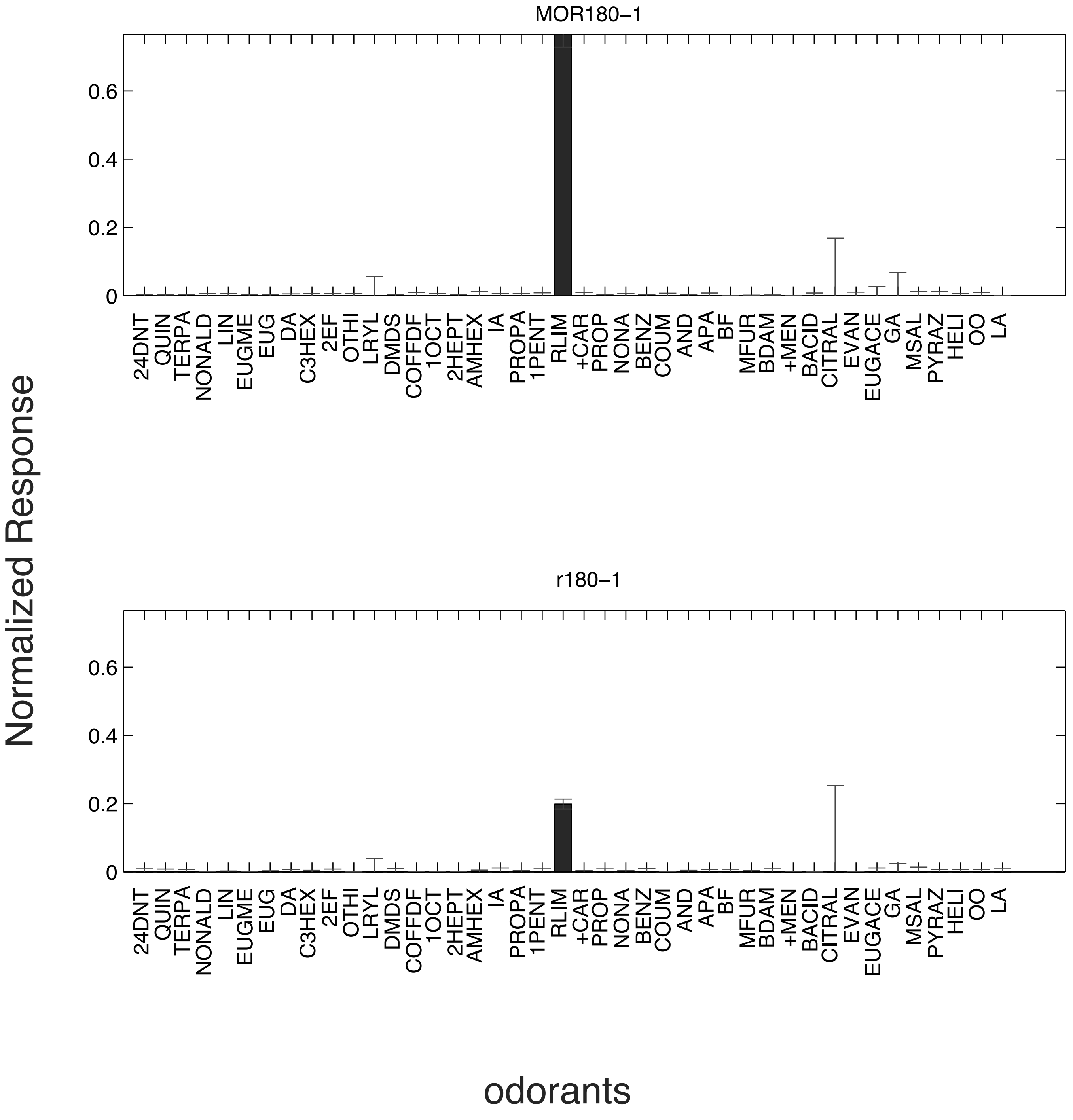
MOR180-1
r180-1
Normalized Response
odorants
0
0.2
0.4
0.6
24DNT
QUIN
TERPA
NONALD
LIN
EUGME
EUG
DA
C3HEX
2EF
OTHI
LRYL
DMDS
COFFDF
1OCT
2HEPT
AMHEX
IA
PROPA
1PENT
RLIM
+CAR
PROP
NONA
BENZ
COUM
AND
APA
BF
MFUR
BDAM
+MEN
BACID
CITRAL
EVAN
EUGACE
GA
MSAL
PYRAZ
HELI
OO
LA

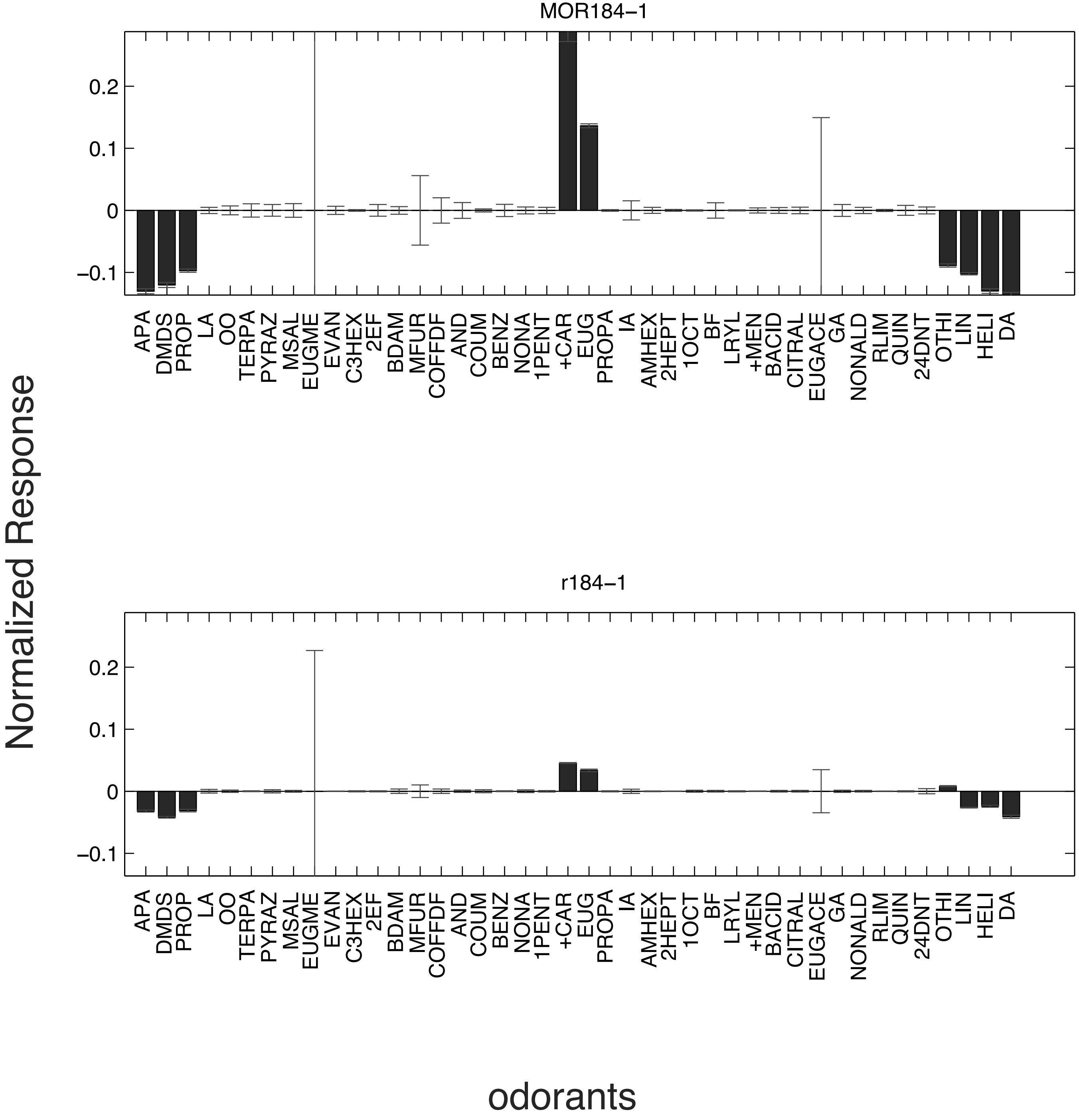

MOR184-1
r184-1
Normalized Response
odorants
0.2
0.1
0
−0.1
APA
DMDS
PROP
LA
OO
TERPA
PYRAZ
MSAL
EUGME
EVAN
C3HEX
2EF
BDAM
MFUR
COFFDF
AND
COUM
BENZ
NONA
1PENT
+CAR
EUG
PROPA
IA
AMHEX
2HEPT
1OCT
BF
LRYL
+MEN
BACID
CITRAL
EUGACE
GA
NONALD
RLIM
QUIN
24DNT
OTHI
LIN
HELI
DA

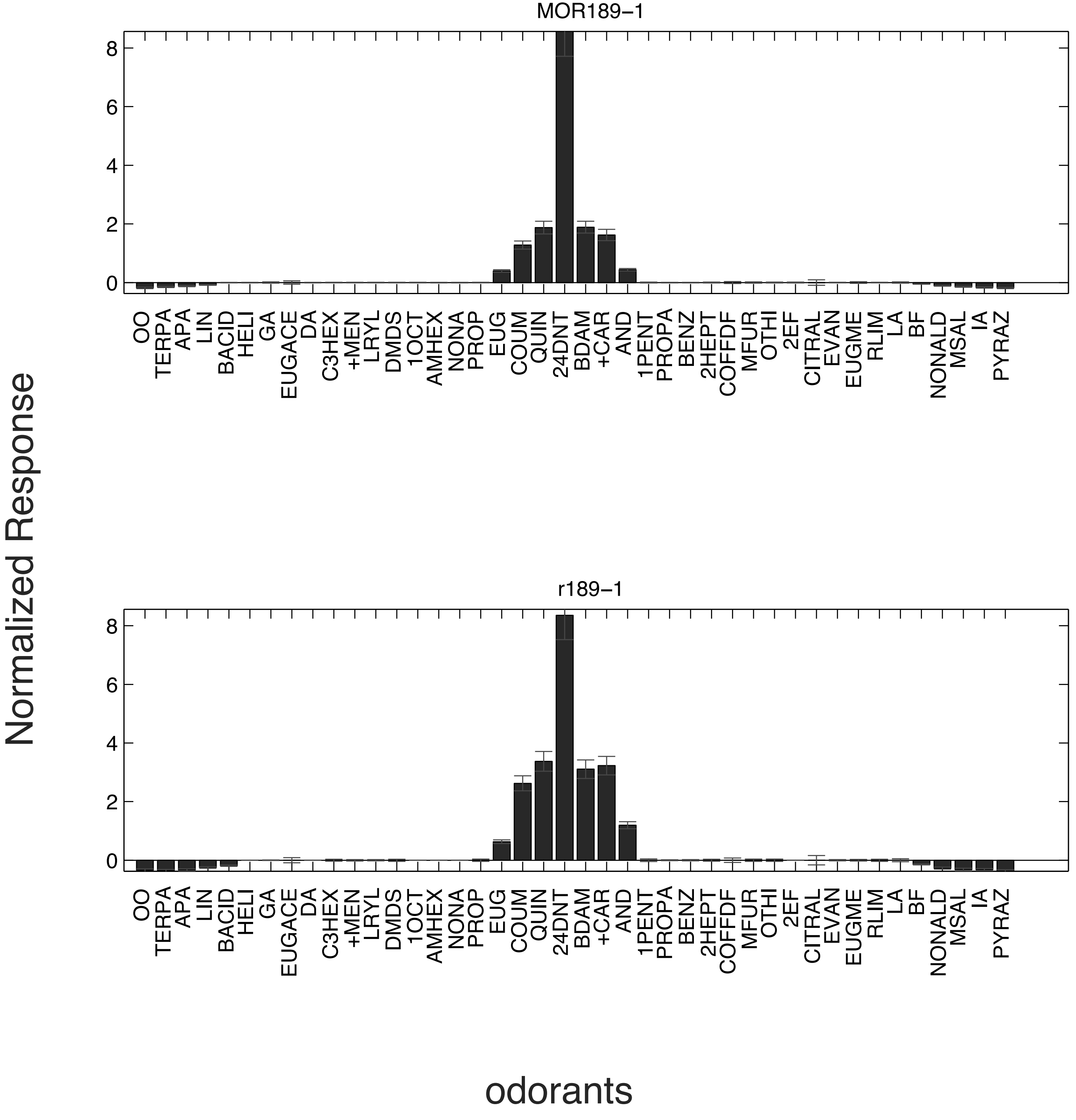
MOR189-1
r189-1
Normalized Response
odorants
0
2
4
6
8
OO
TERPA
APA
LIN
BACID
HELI
GA
EUGACE
DA
C3HEX
+MEN
LRYL
DMDS
1OCT
AMHEX
NONA
PROP
EUG
COUM
QUIN
24DNT
BDAM
+CAR
AND
1PENT
PROPA
BENZ
2HEPT
COFFDF
MFUR
OTHI
2EF
CITRAL
EVAN
EUGME
RLIM
LA
BF
NONALD
MSAL
IA
PYRAZ

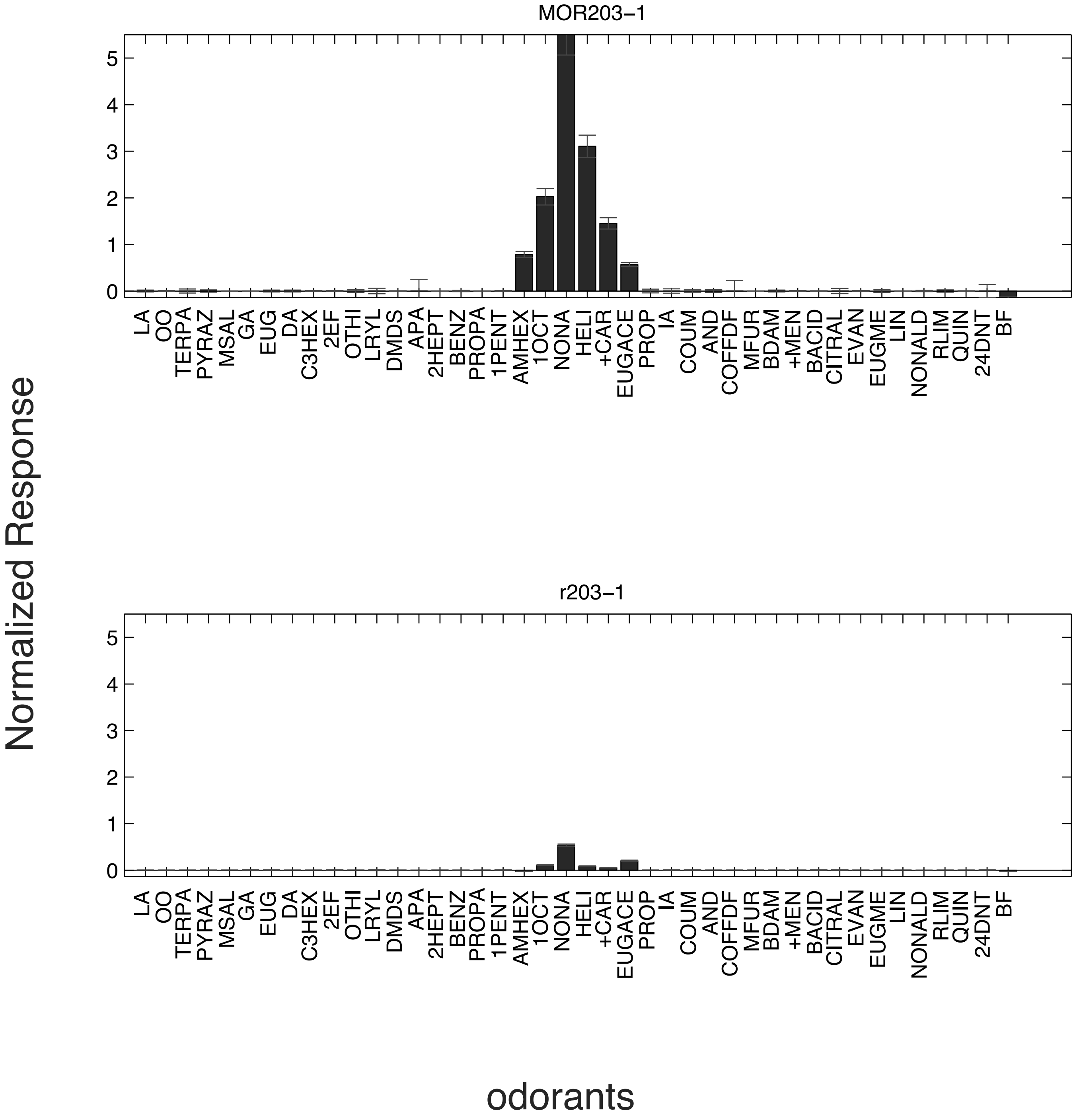
MOR203-1
r203-1
Normalized Response
odorants
0
1
2
3
4
5
LA
OO
TERPA
PYRAZ
MSAL
GA
EUG
DA
C3HEX
2EF
OTHI
LRYL
DMDS
APA
2HEPT
BENZ
PROPA
1PENT
AMHEX
1OCT
NONA
HELI
+CAR
EUGACE
PROP
IA
COUM
AND
COFFDF
MFUR
BDAM
+MEN
BACID
CITRAL
EVAN
EUGME
LIN
NONALD
RLIM
QUIN
24DNT
BF

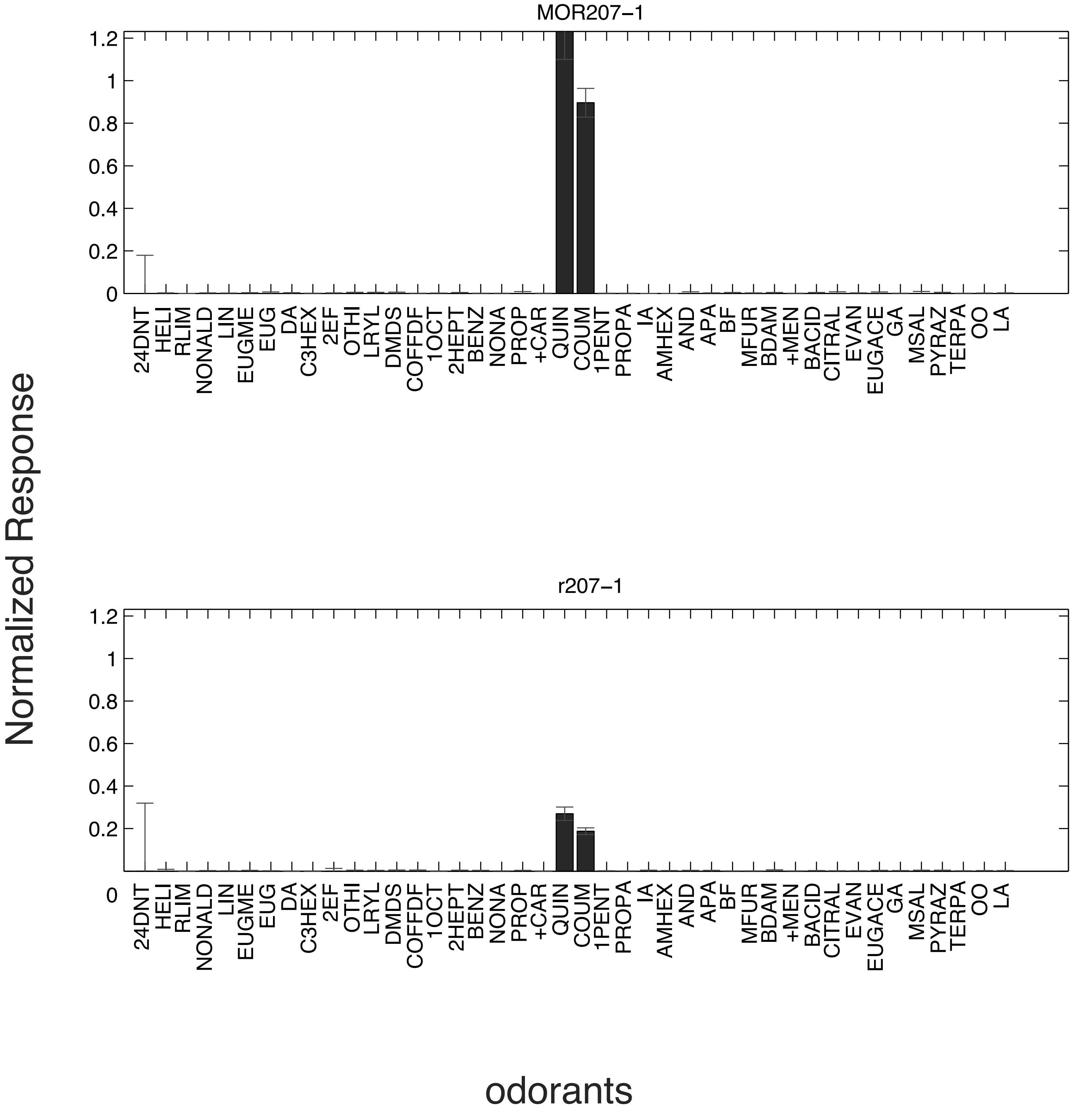

MOR207-1
r207-1
Normalized Response
odorants
24DNT
HELI
RLIM
NONALD
LIN
EUGME
EUG
DA
C3HEX
2EF
OTHI
LRYL
DMDS
COFFDF
1OCT
2HEPT
BENZ
NONA
PROP
+CAR
QUIN
COUM
1PENT
PROPA
IA
AMHEX
AND
APA
BF
MFUR
BDAM
+MEN
BACID
CITRAL
EVAN
EUGACE
GA
MSAL
PYRAZ
TERPA
OO
LA

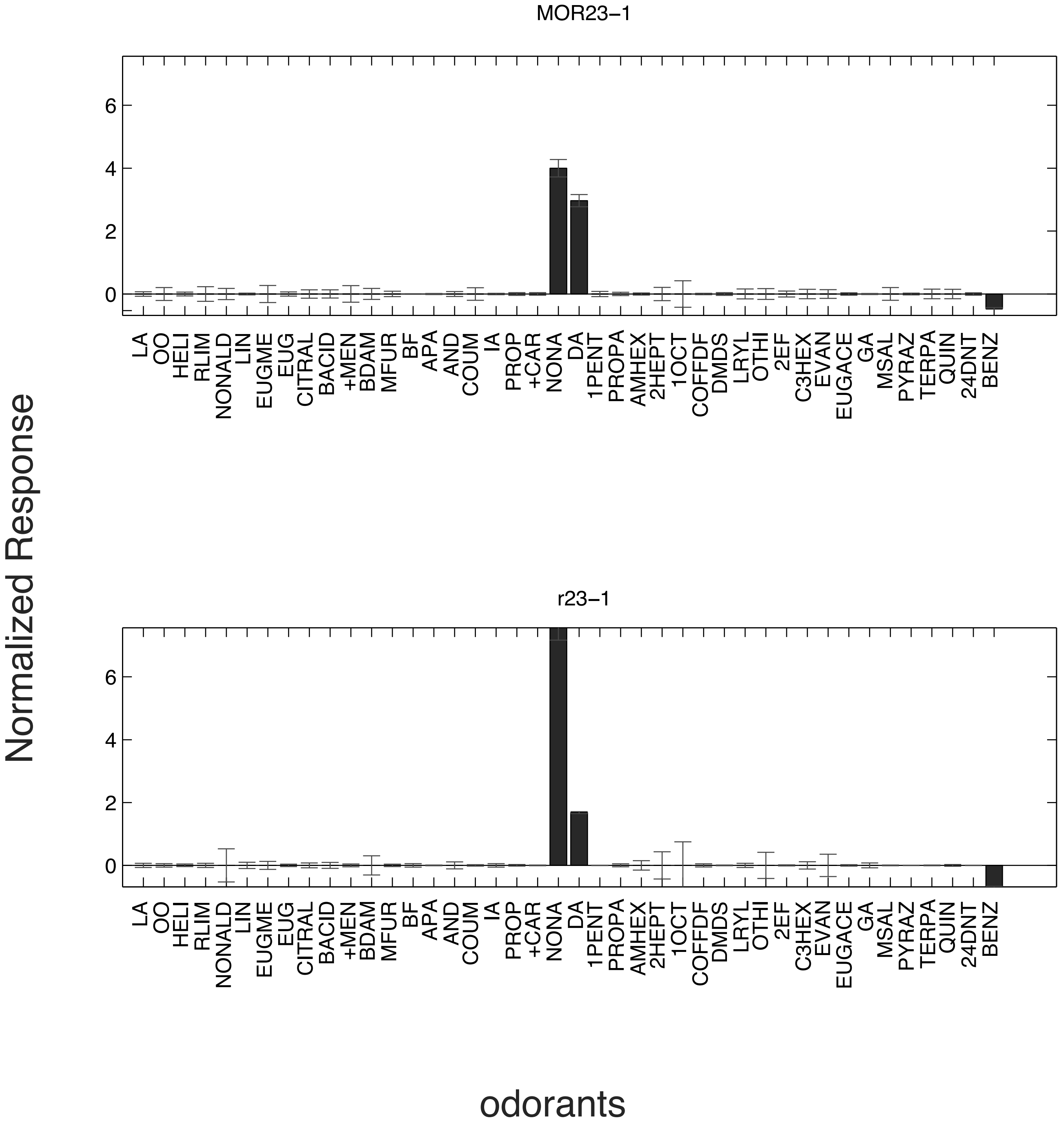
MOR23-1
6
4
2
0
LA OO HELI RLIM NONALD LIN EUGME EUG CITRAL BACID +MEN BDAM MFUR BF APA AND COUM IA PROP +CAR NONA DA 1PENT PROPA AMHEX 2HEPT 1OCT COFFDF DMDS LRYL OTHI 2EF C3HEX EVAN EUGACE GA MSAL PYRAZ TERPA QUIN 24DNT BENZ
r23-1
6
4
2
0
LA OO HELI RLIM NONALD LIN EUGME EUG CITRAL BACID +MEN BDAM MFUR BF APA AND COUM IA PROP +CAR NONA DA 1PENT PROPA AMHEX 2HEPT 1OCT COFFDF DMDS LRYL OTHI 2EF C3HEX EVAN EUGACE GA MSAL PYRAZ TERPA QUIN 24DNT BENZ
Normalized Response
odorants

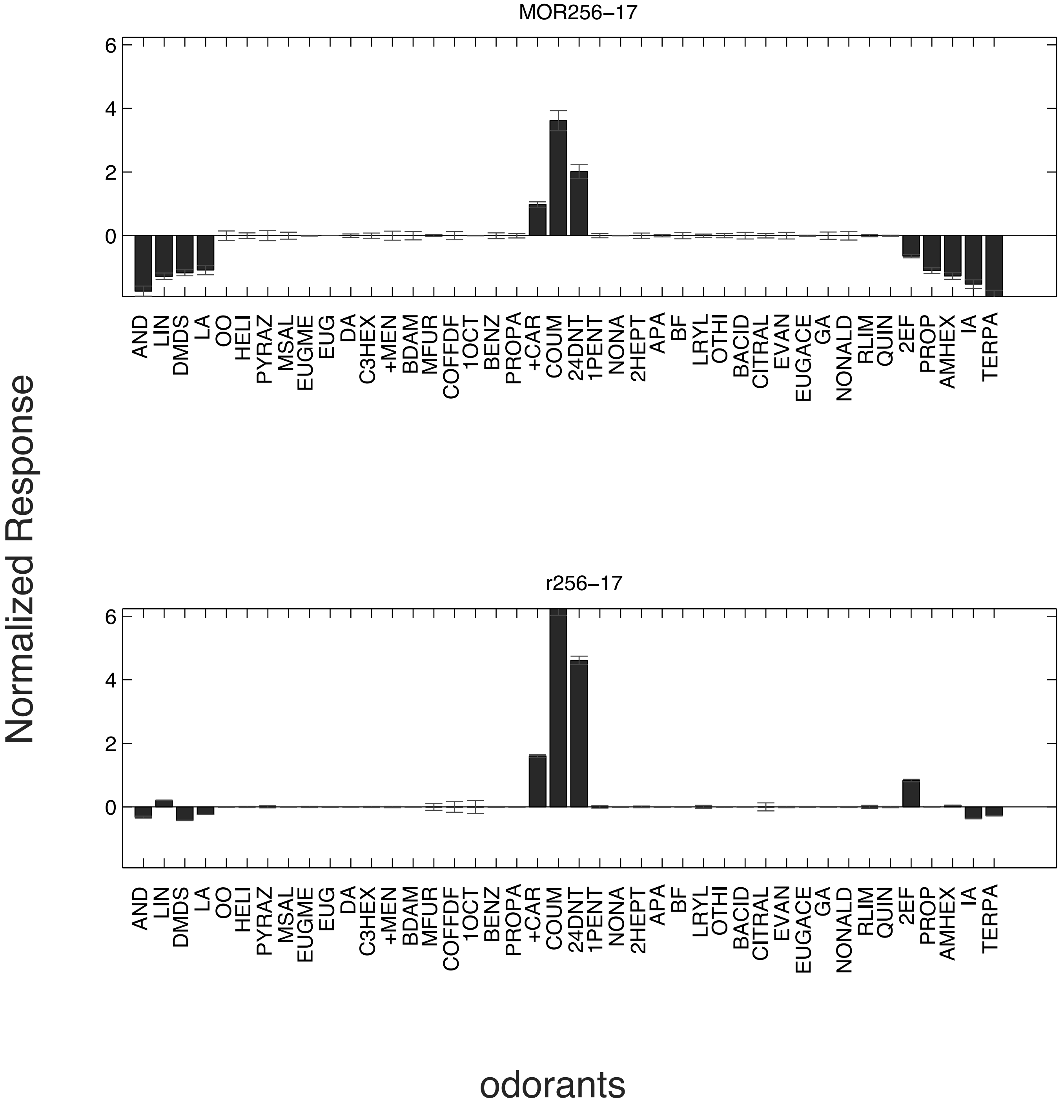

MOR256-17
r256-17
Normalized Response
odorants
6
4
2
0
AND
LIN
DMDS
LA
OO
HELI
PYRAZ
MSAL
EUGME
EUG
DA
C3HEX
+MEN
BDAM
MFUR
COFFDF
1OCT
BENZ
PROPA
+CAR
COUM
24DNT
1PENT
NONA
2HEPT
APA
BF
LRYL
OTHI
BACID
CITRAL
EVAN
EUGACE
GA
NONALD
RLIM
QUIN
2EF
PROP
AMHEX
IA
TERPA

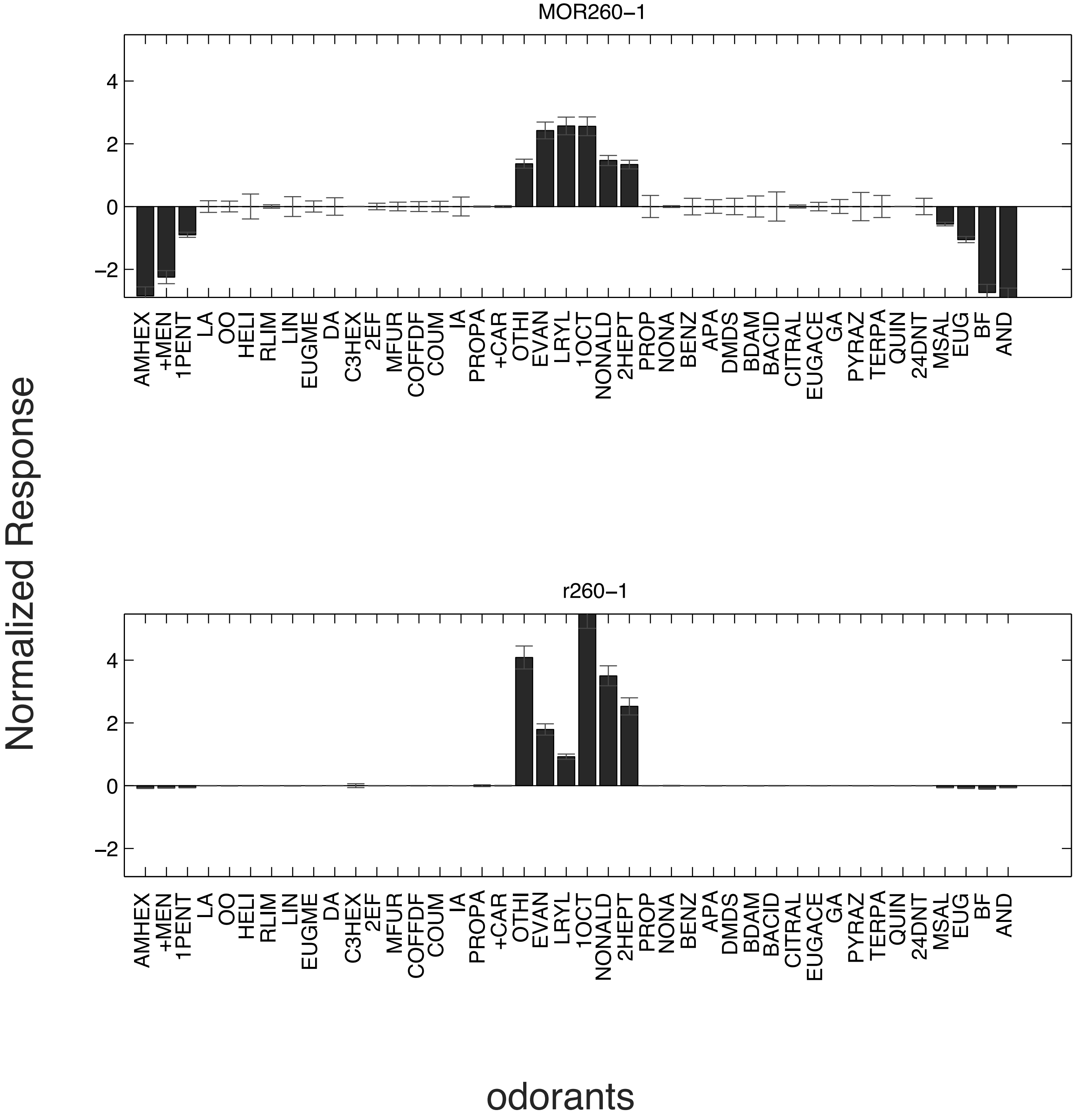
MOR260-1
r260-1
4
2
0
−2
AMHEX
+MEN
1PENT
LA
OO
HELI
RLIM
LIN
EUGME
DA
C3HEX
2EF
MFUR
COFFDF
COUM
IA
PROPA
+CAR
OTHI
EVAN
LRYL
1OCT
NONALD
2HEPT
PROP
NONA
BENZ
APA
DMDS
BDAM
BACID
CITRAL
EUGACE
GA
PYRAZ
TERPA
QUIN
24DNT
MSAL
EUG
BF
AND
Normalized Response
odorants

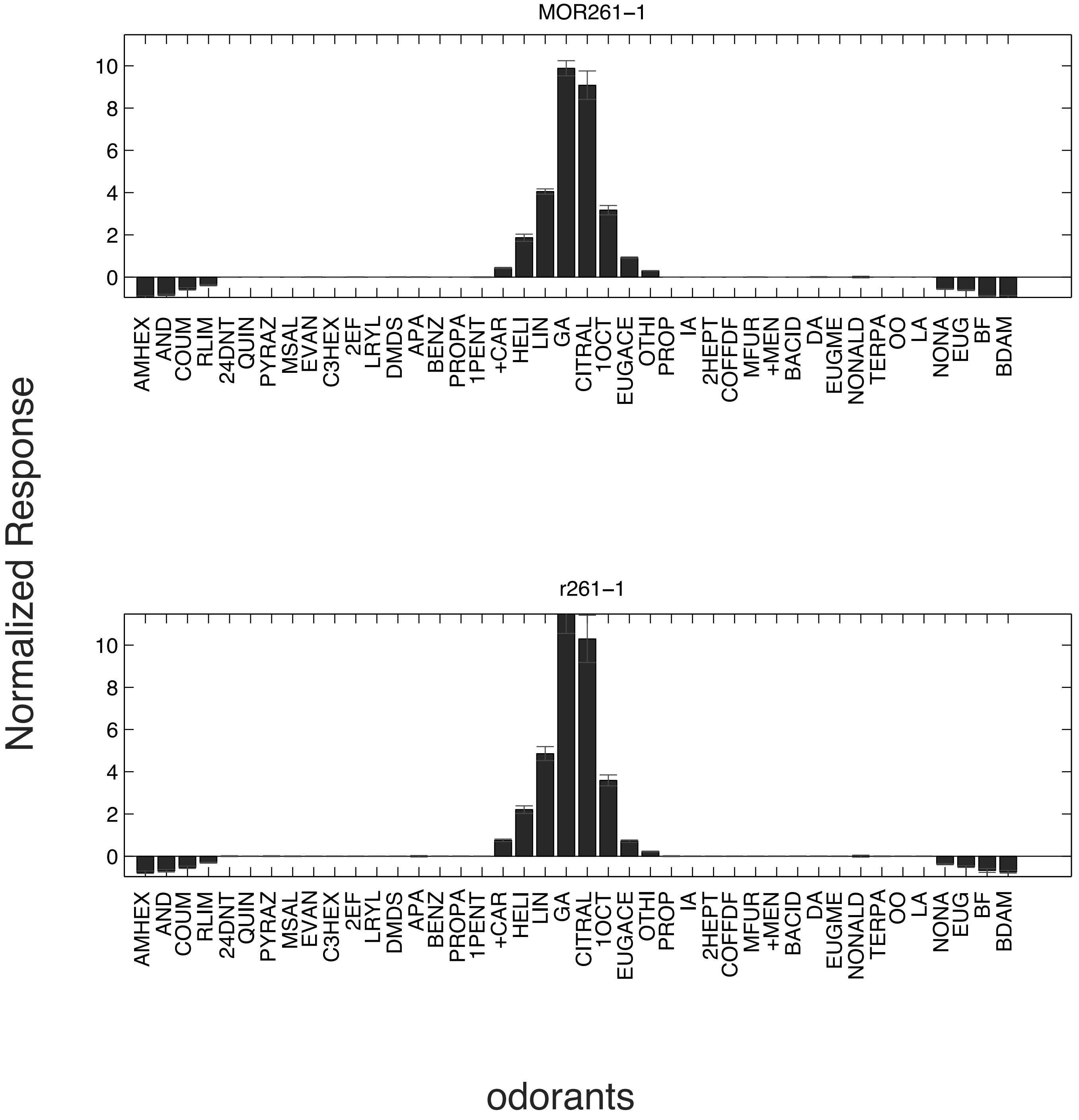
MOR261-1
r261-1
Normalized Response
odorants
0
2
4
6
8
10
AMHEX
AND
COUM
RLIM
24DNT
QUIN
PYRAZ
MSAL
EVAN
C3HEX
2EF
LRYL
DMDS
APA
BENZ
PROPA
1PENT
+CAR
HELI
LIN
GA
CITRAL
1OCT
EUGACE
OTHI
PROP
IA
2HEPT
COFFDF
MFUR
+MEN
BACID
DA
EUGME
NONALD
TERPA
OO
LA
NONA
EUG
BF
BDAM

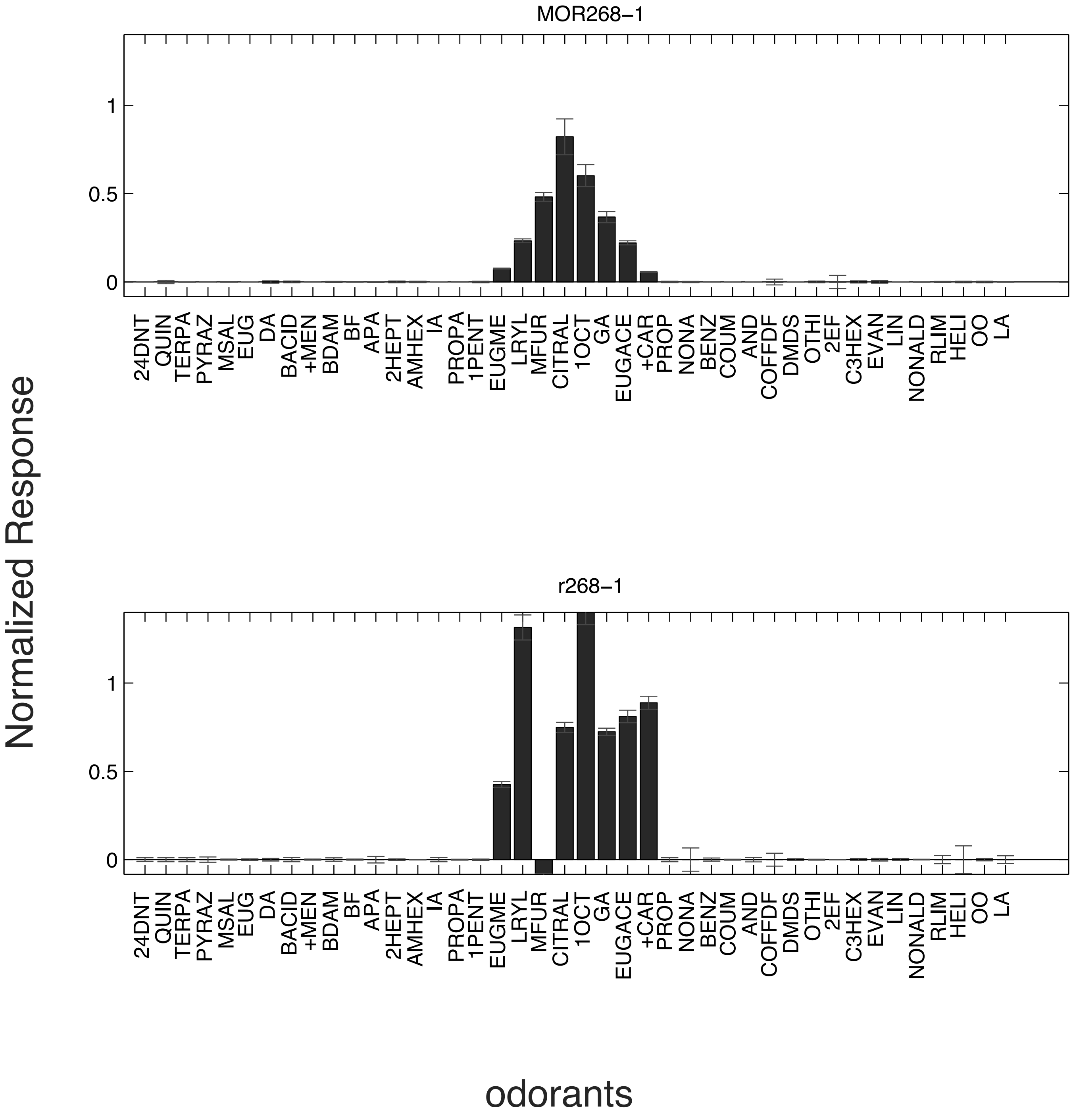
MOR268-1
r268-1
Normalized Response
odorants
0
0.5
1
24DNT
QUIN
TERPA
PYRAZ
MSAL
EUG
DA
BACID
+MEN
BDAM
BF
APA
2HEPT
AMHEX
IA
PROPA
1PENT
EUGME
LRYL
MFUR
CITRAL
1OCT
GA
EUGACE
+CAR
PROP
NONA
BENZ
COUM
AND
COFFDF
DMDS
OTHI
2EF
C3HEX
EVAN
LIN
NONALD
RLIM
HELI
OO
LA

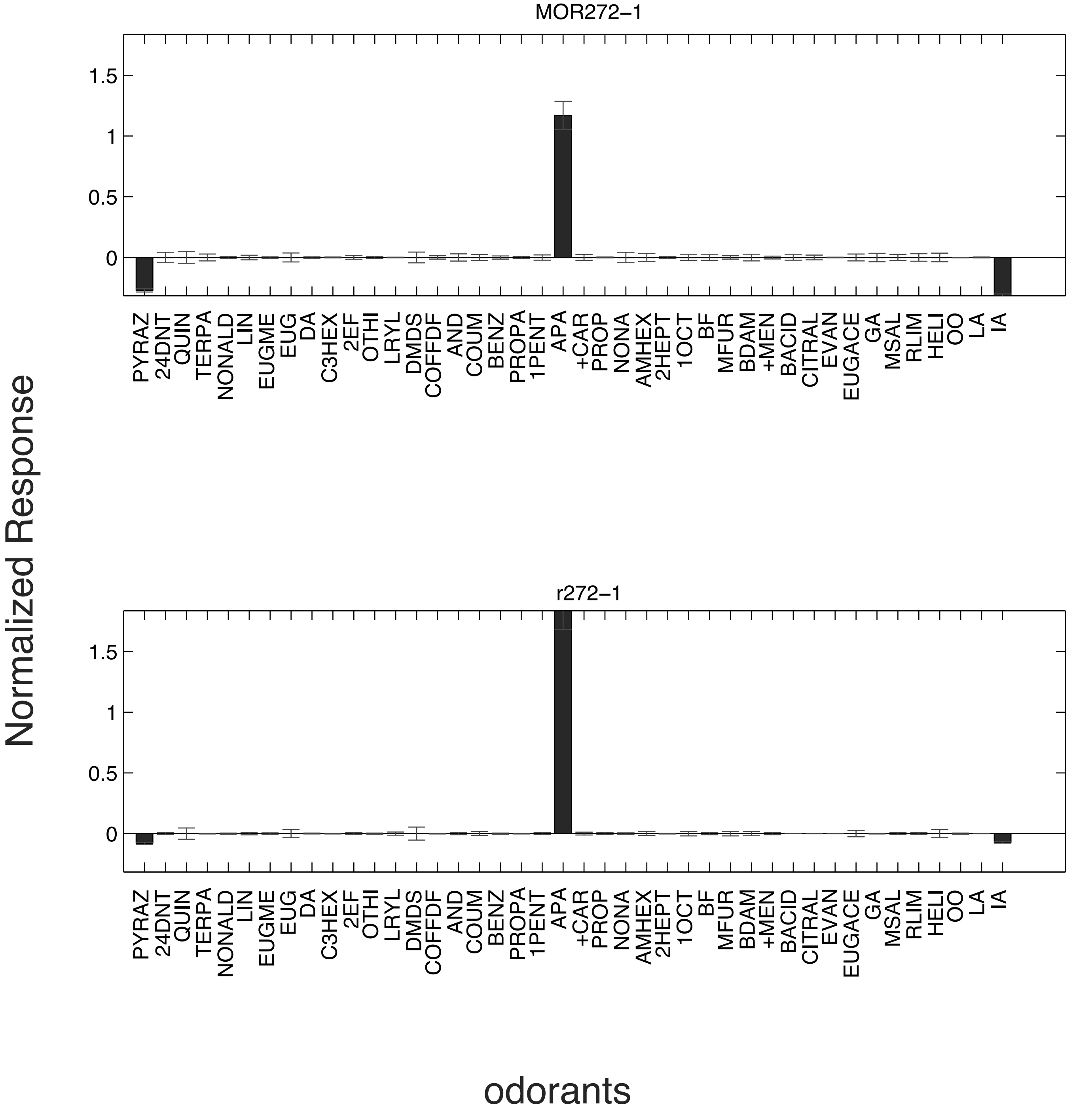
MOR272-1
r272-1
Normalized Response
odorants
1.5
1
0.5
0
PYRAZ
24DNT
QUIN
TERPA
NONALD
LIN
EUGME
EUG
DA
C3HEX
2EF
OTHI
LRYL
DMDS
COFFDF
AND
COUM
BENZ
PROPA
1PENT
APA
+CAR
PROP
NONA
AMHEX
2HEPT
1OCT
BF
MFUR
BDAM
+MEN
BACID
CITRAL
EVAN
EUGACE
GA
MSAL
RLIM
HELI
OO
LA
IA

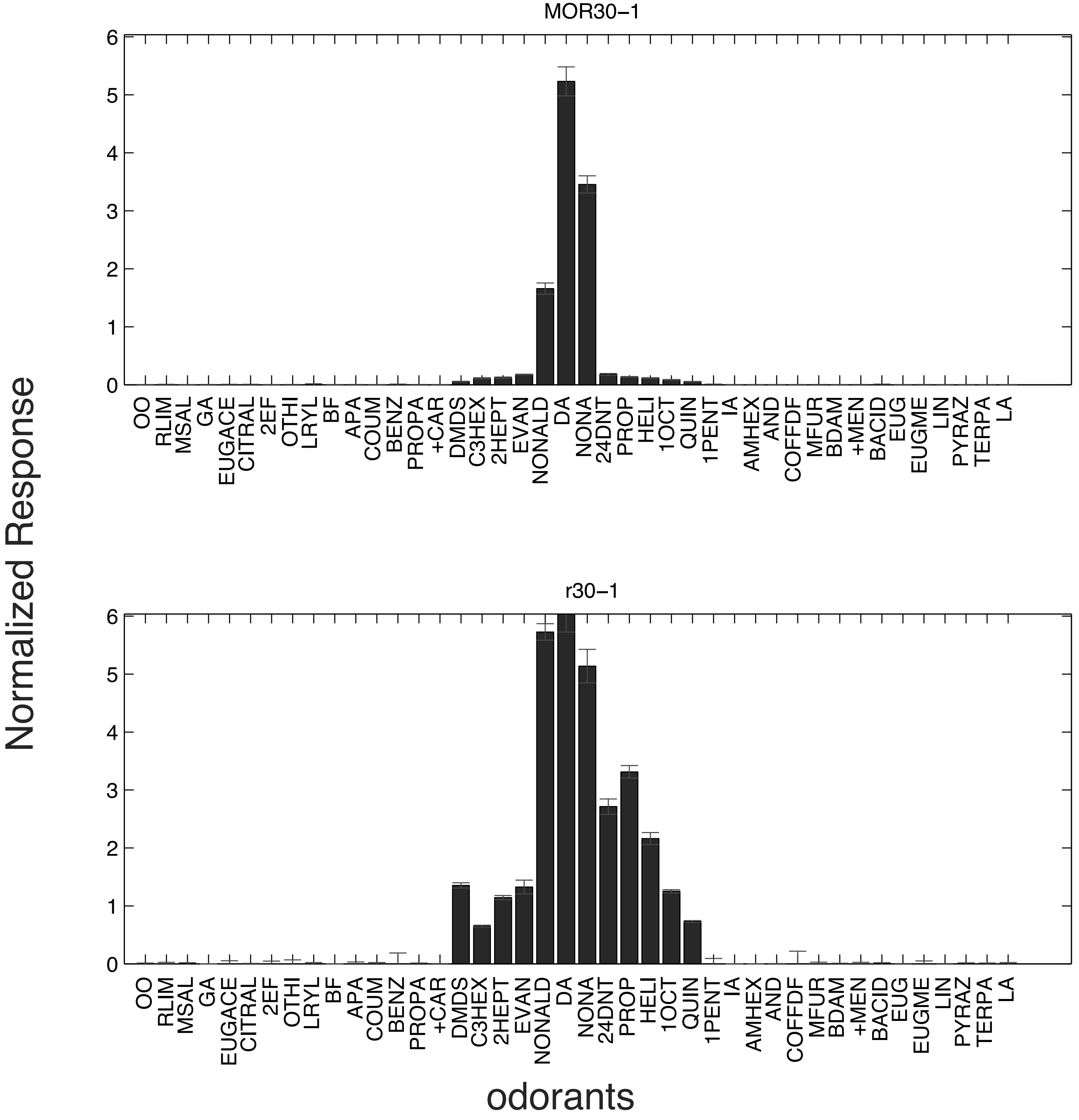
MOR30-1
r30-1
Normalized Response
odorants
0
1
2
3
4
5
6
OO
RLIM
MSAL
GA
EUGACE
CITRAL
2EF
OTHI
LRYL
BF
APA
COUM
BENZ
PROPA
+CAR
DMDS
C3HEX
2HEPT
EVAN
NONALD
DA
NONA
24DNT
PROP
HELI
1OCT
QUIN
1PENT
IA
AMHEX
AND
COFFDF
MFUR
BDAM
+MEN
BACID
EUG
EUGME
LIN
PYRAZ
TERPA
LA

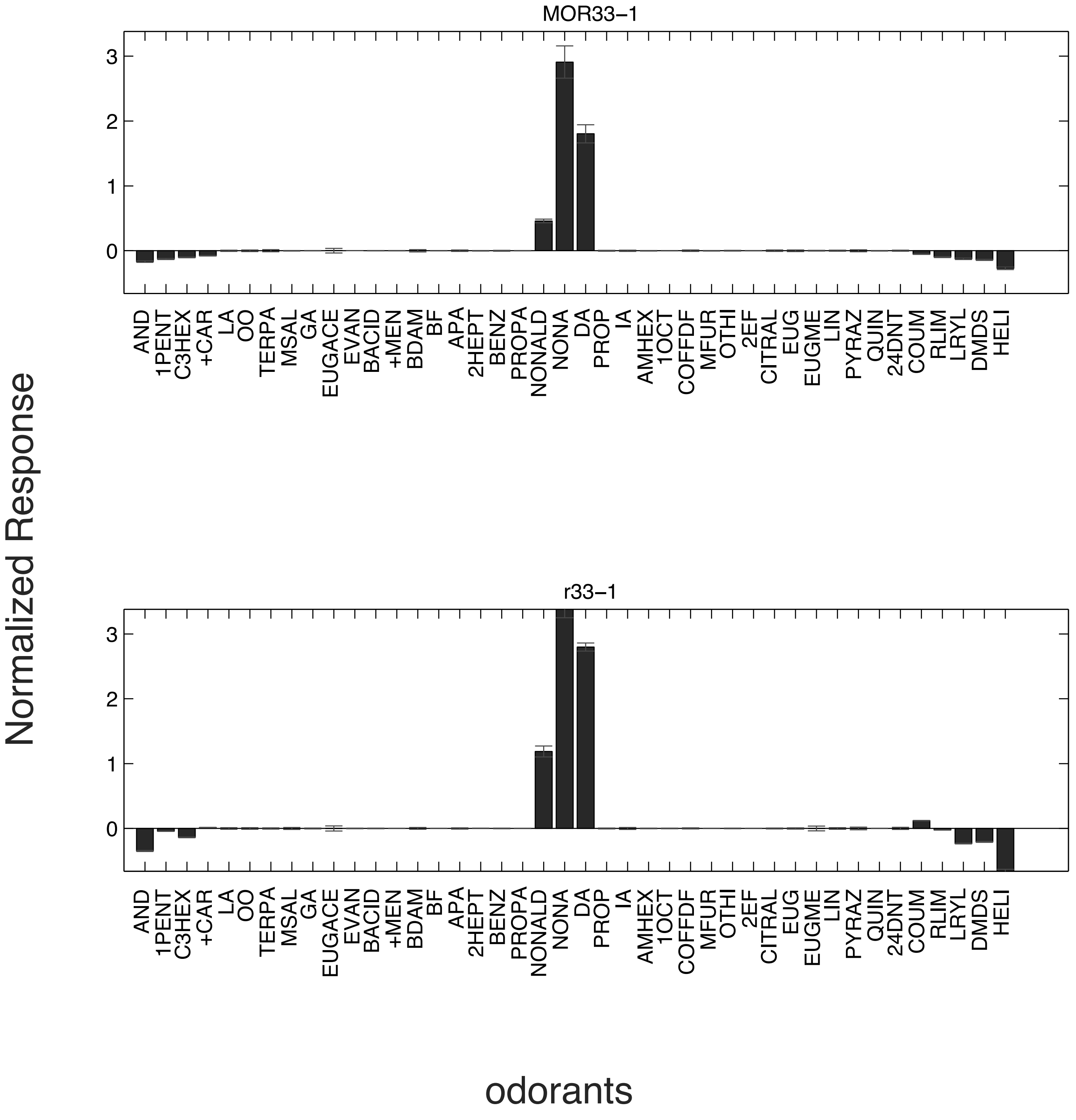

MOR33-1
r33-1
Normalized Response
odorants
AND
1PENT
C3HEX
+CAR
LA
OO
TERPA
MSAL
GA
EUGACE
EVAN
BACID
+MEN
BDAM
BF
APA
2HEPT
BENZ
PROPA
NONALD
NONA
DA
PROP
IA
AMHEX
1OCT
COFFDF
MFUR
OTHI
2EF
CITRAL
EUG
EUGME
LIN
PYRAZ
QUIN
24DNT
COUM
RLIM
LRYL
DMDS
HELI
