# Supplementary material for: Functional Evolution of Mammalian Odorant Receptors
Source: PLoS Genet. 2012 Jul 12;8(7):e1002821. doi: 10.1371/journal.pgen.1002821 (PMC3395614; doi:10.1371/journal.pgen.1002821)
Supplement: Figure S10 — Phylogenetic relationship of OR orthologs and paralogs. A phylogenetic tree for each OR set was constructed using the Neighbor-Joining method in Seaview. Bootstrap values of 100 replicates are included for each branch. For all OR sets, human-chimpanzee-rhesus macaque orthologs are most closely related to each other, while mouse best-hit ORs (presumably human-mouse orthologs) and human paralogs have unique relationships among each OR set. Human reference OR is in black text, ORs that responded to a common a ligand are in green and ORs that did not respond to a common ligand are in red. (PDF) [file pgen.1002821.s010.pdf]

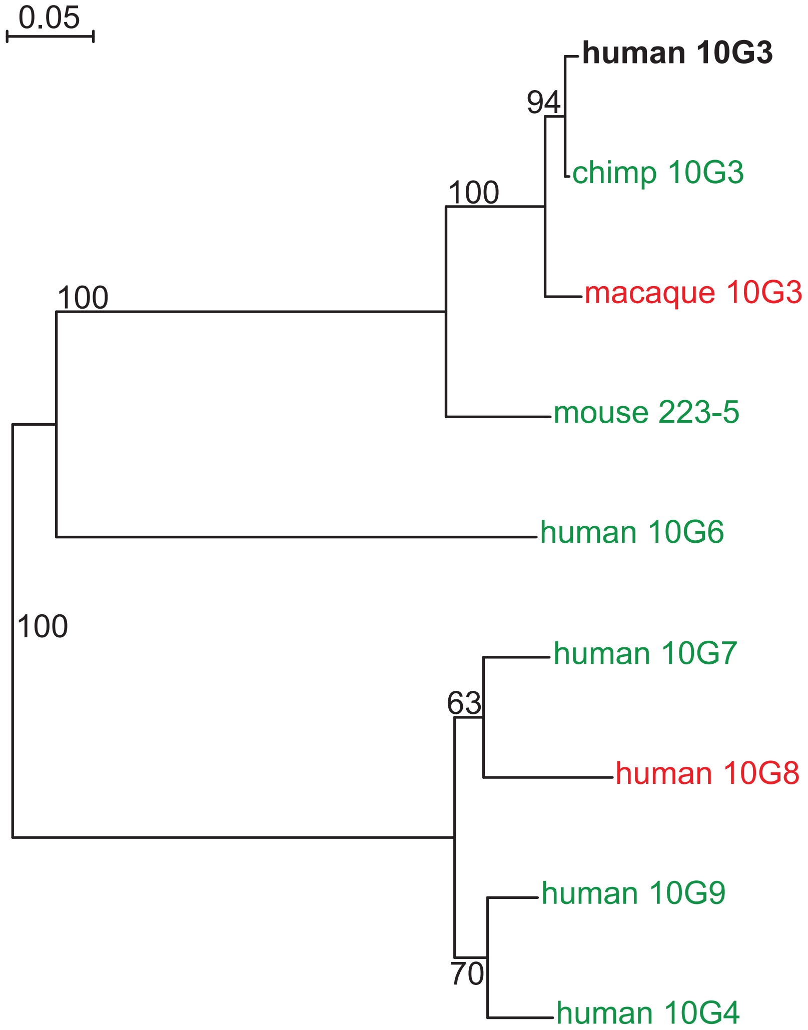
0.05
human 10G3
94
chimp 10G3
100
100
macaque 10G3
mouse 223-5
human 10G6
100
human 10G7
63
human 10G8
human 10G9
70
human 10G4

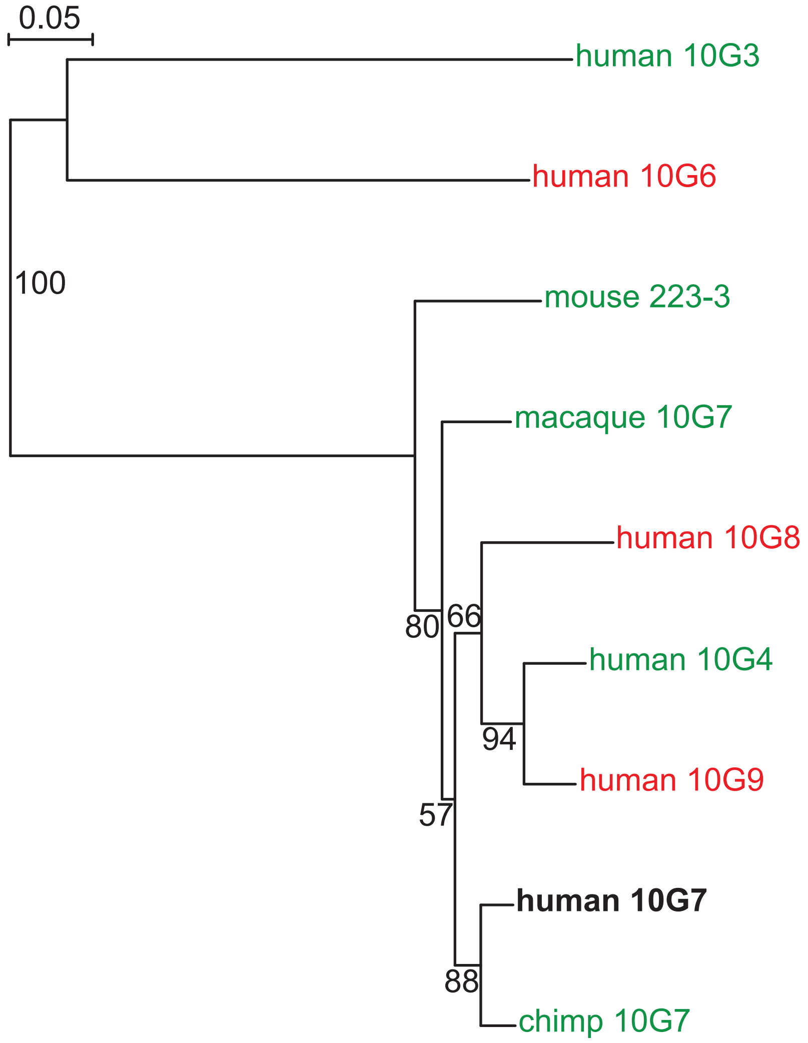
0.05
human 10G3
human 10G6
100
mouse 223-3
macaque 10G7
human 10G8
80
66
human 10G4
94
human 10G9
57
human 10G7
88
chimp 10G7

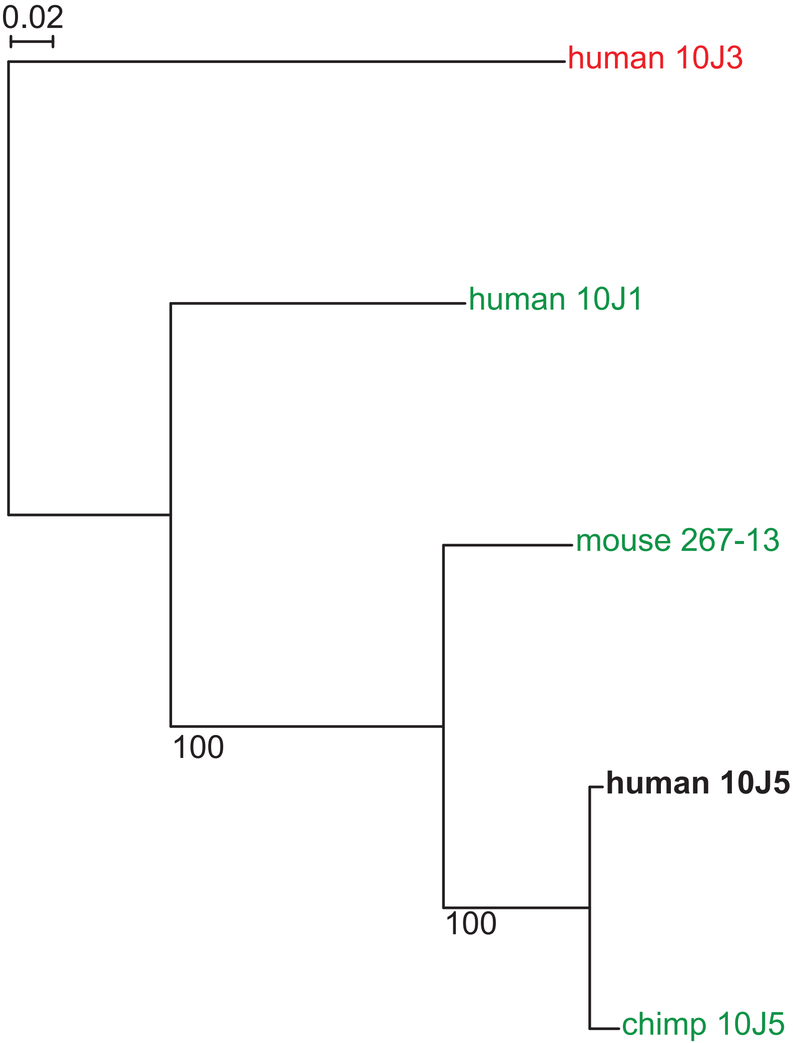
0.02
human 10J3
human 10J1
mouse 267-13
100
human 10J5
100
chimp 10J5

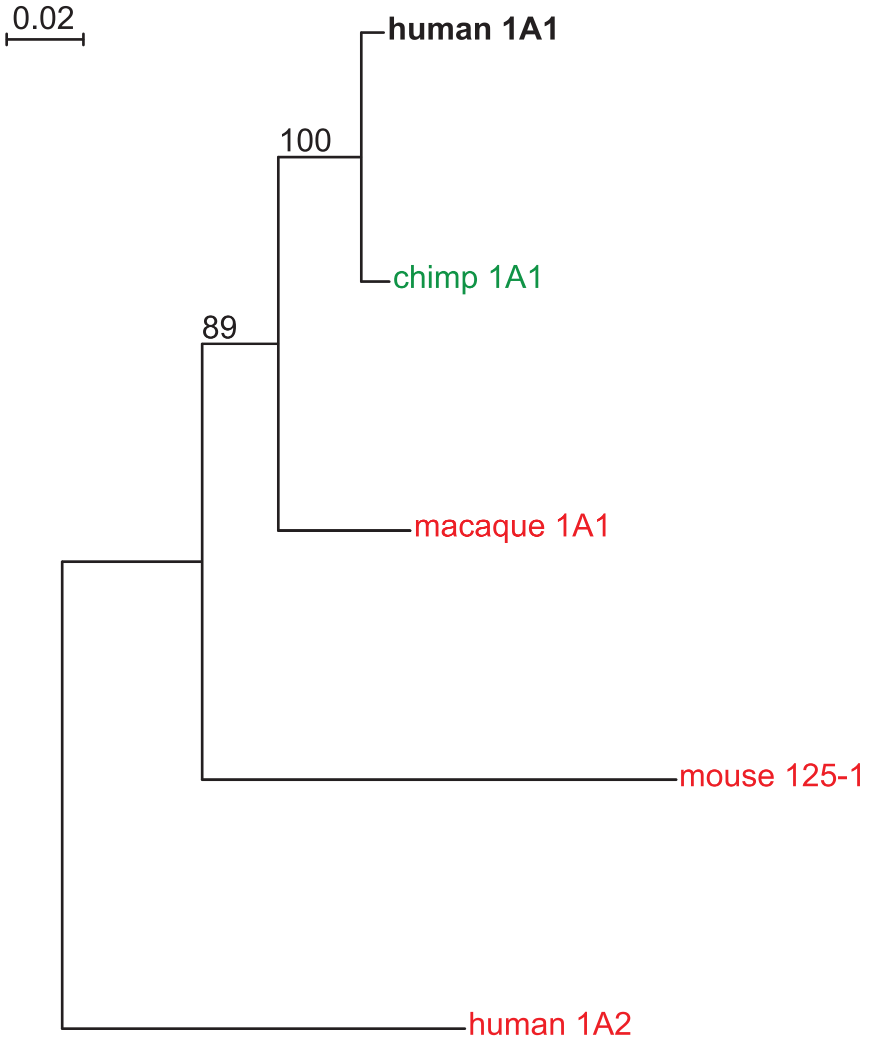
0.02
human 1A1
100
chimp 1A1
89
macaque 1A1
mouse 125-1
human 1A2

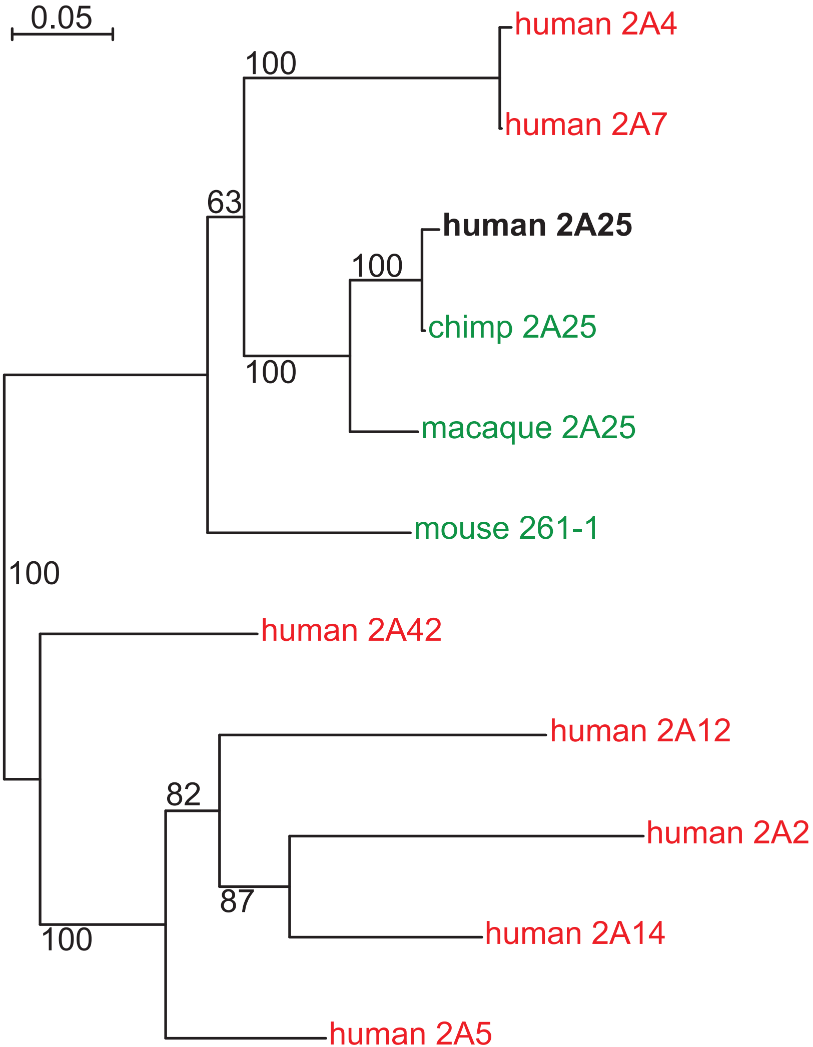
0.05
human 2A4
human 2A7
human 2A25
chimp 2A25
macaque 2A25
mouse 261-1
human 2A42
human 2A12
human 2A2
human 2A14
human 2A5
100
63
100
100
100
100
82
87

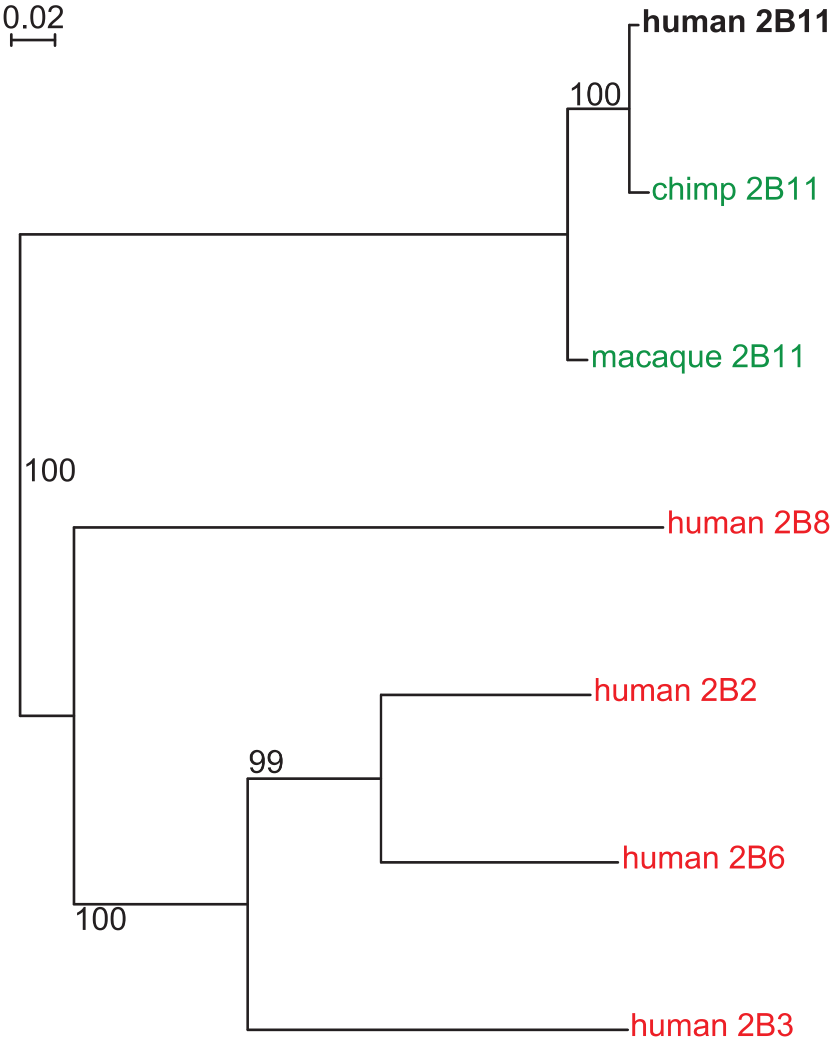
0.02
human 2B11
100
chimp 2B11
macaque 2B11
100
human 2B8
human 2B2
99
human 2B6
100
human 2B3

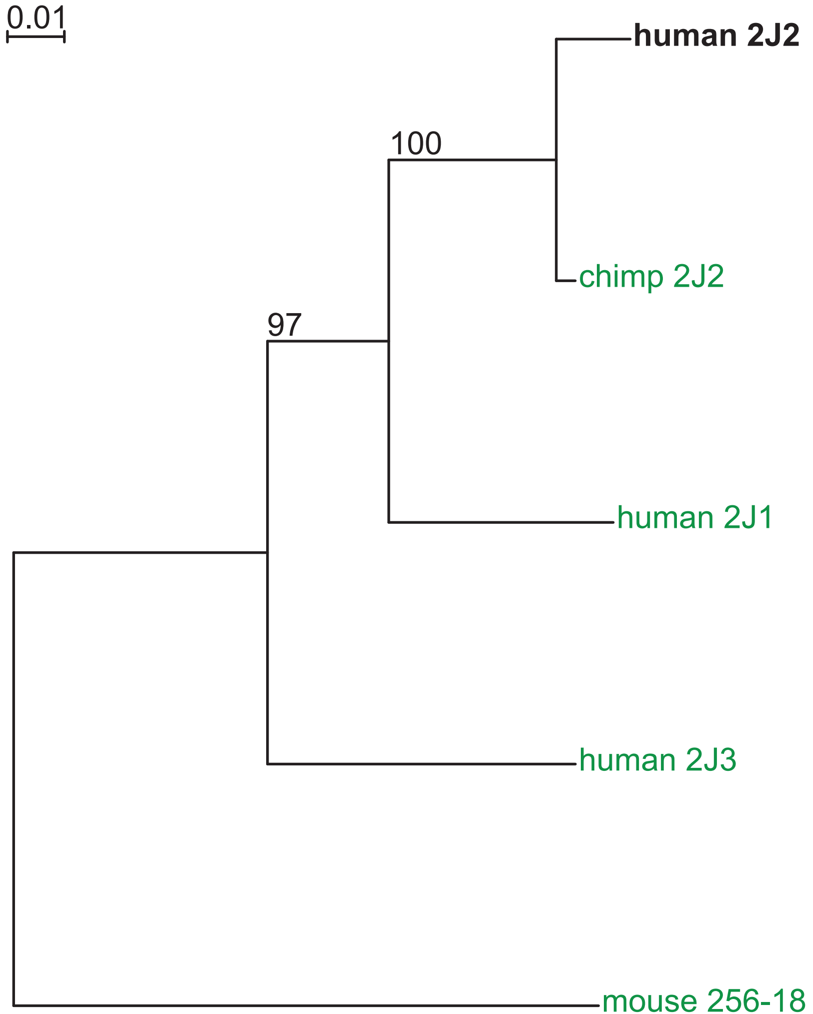
0.01
human 2J2
100
chimp 2J2
97
human 2J1
human 2J3
mouse 256-18

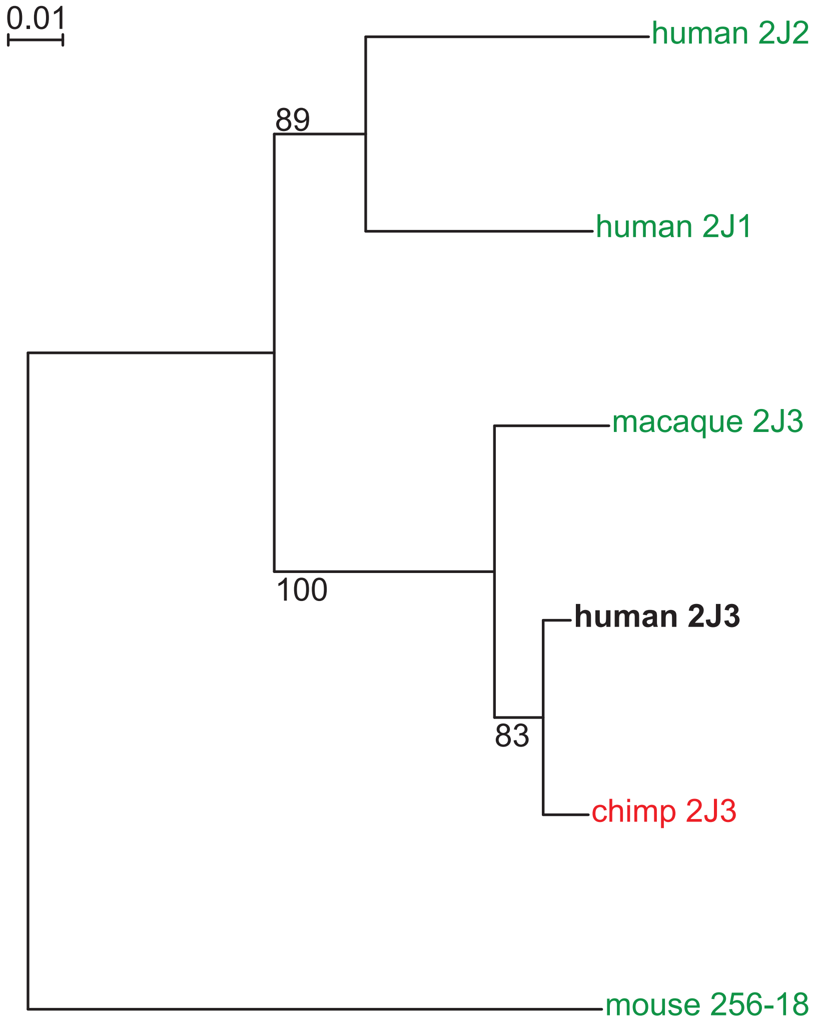
0.01
human 2J2
89
human 2J1
macaque 2J3
100
human 2J3
83
chimp 2J3
mouse 256-18

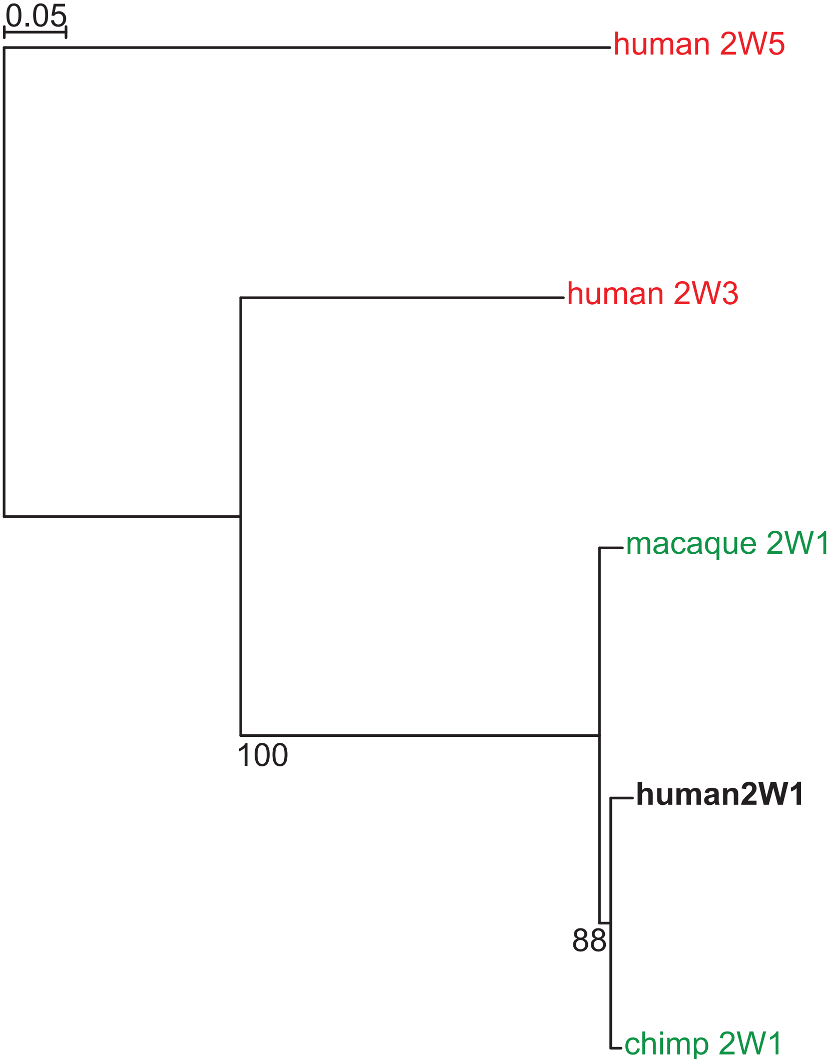
0.05
human 2W5
human 2W3
macaque 2W1
100
human2W1
88
chimp 2W1

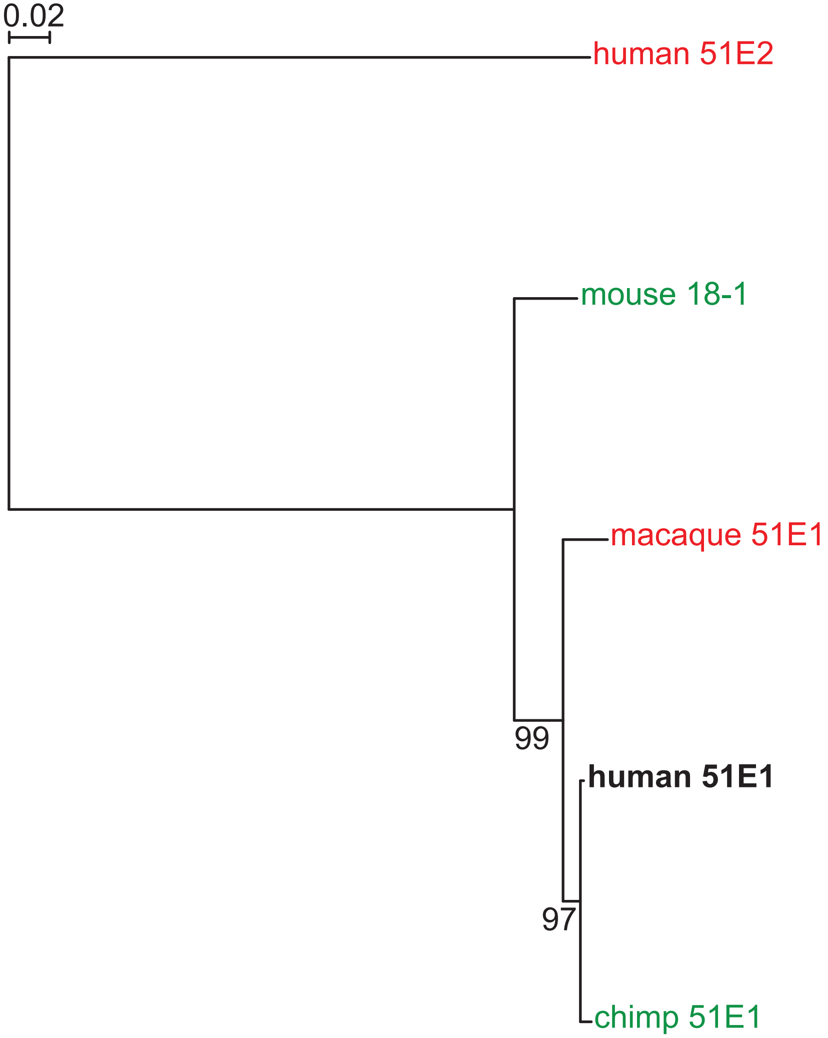
0.02
human 51E2
mouse 18-1
macaque 51E1
99
human 51E1
97
chimp 51E1

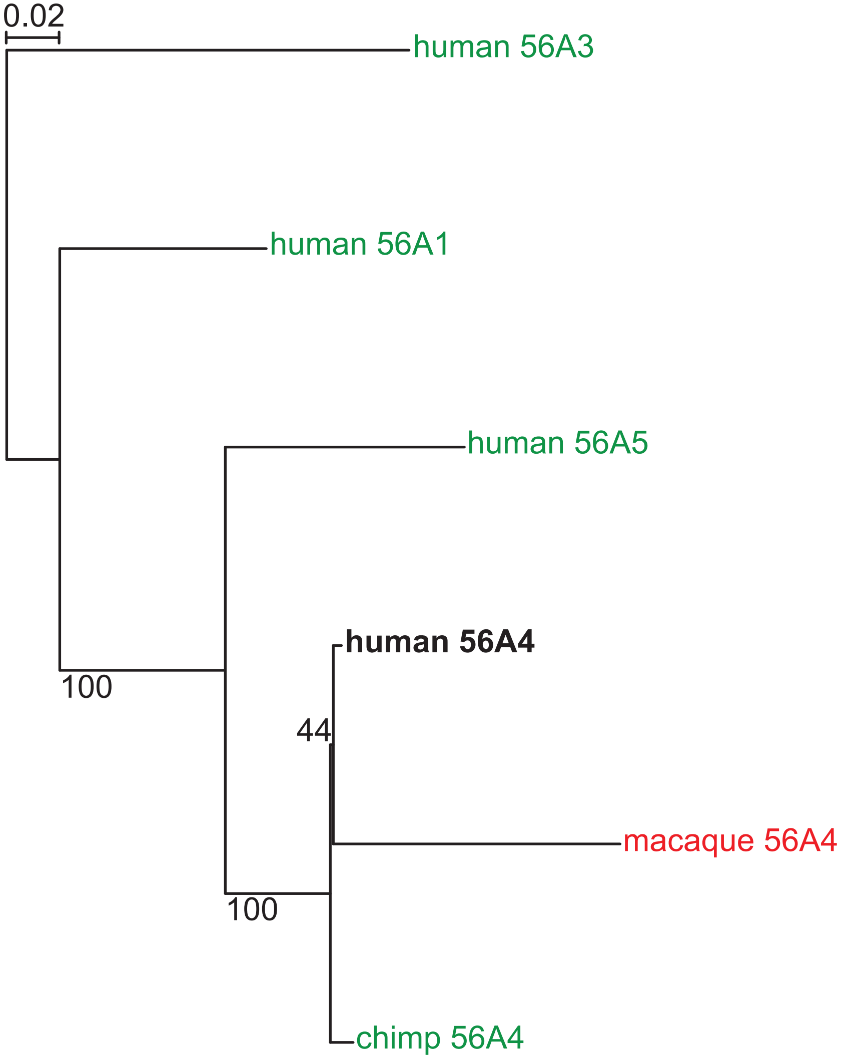
0.02
human 56A3
human 56A1
human 56A5
human 56A4
100
44
macaque 56A4
100
chimp 56A4

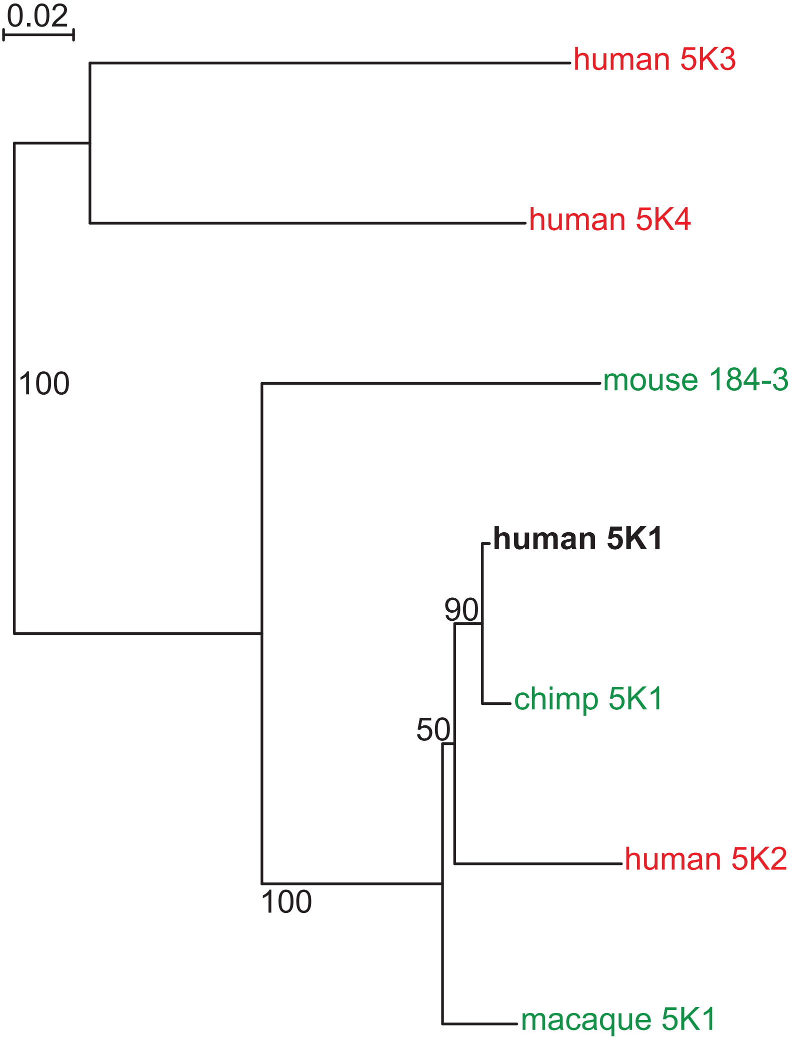
0.02
human 5K3
human 5K4
100
mouse 184-3
human 5K1
90
chimp 5K1
50
human 5K2
100
macaque 5K1

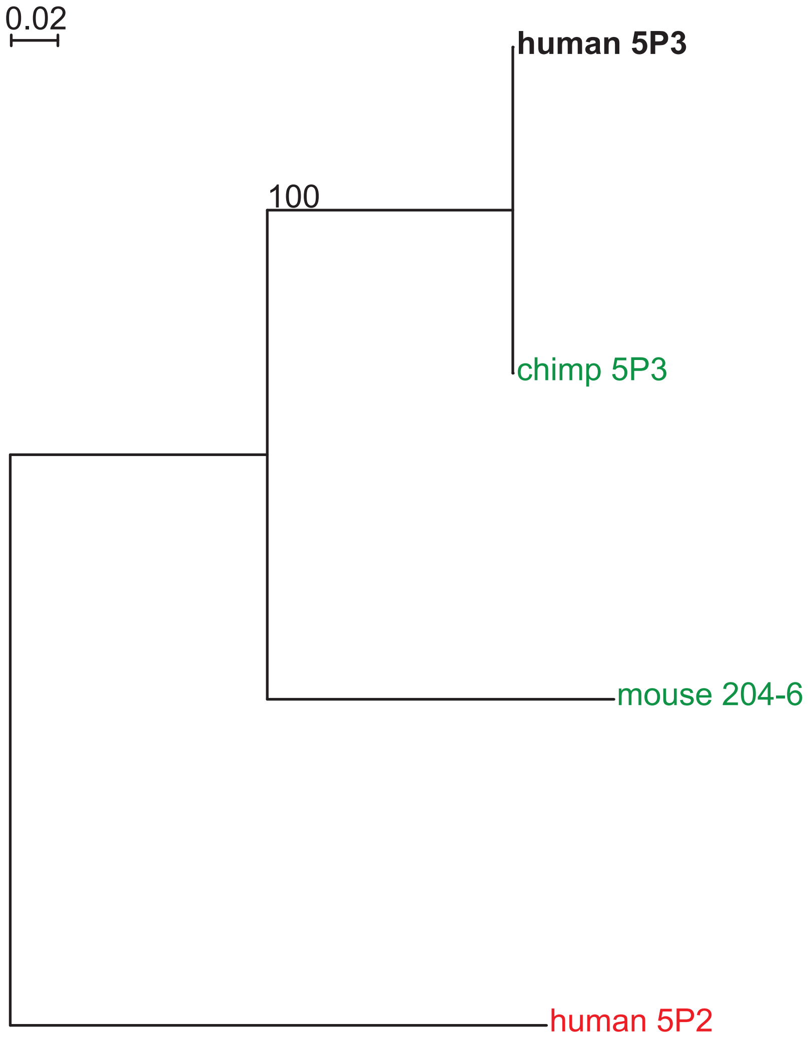
0.02
human 5P3
100
chimp 5P3
mouse 204-6
human 5P2

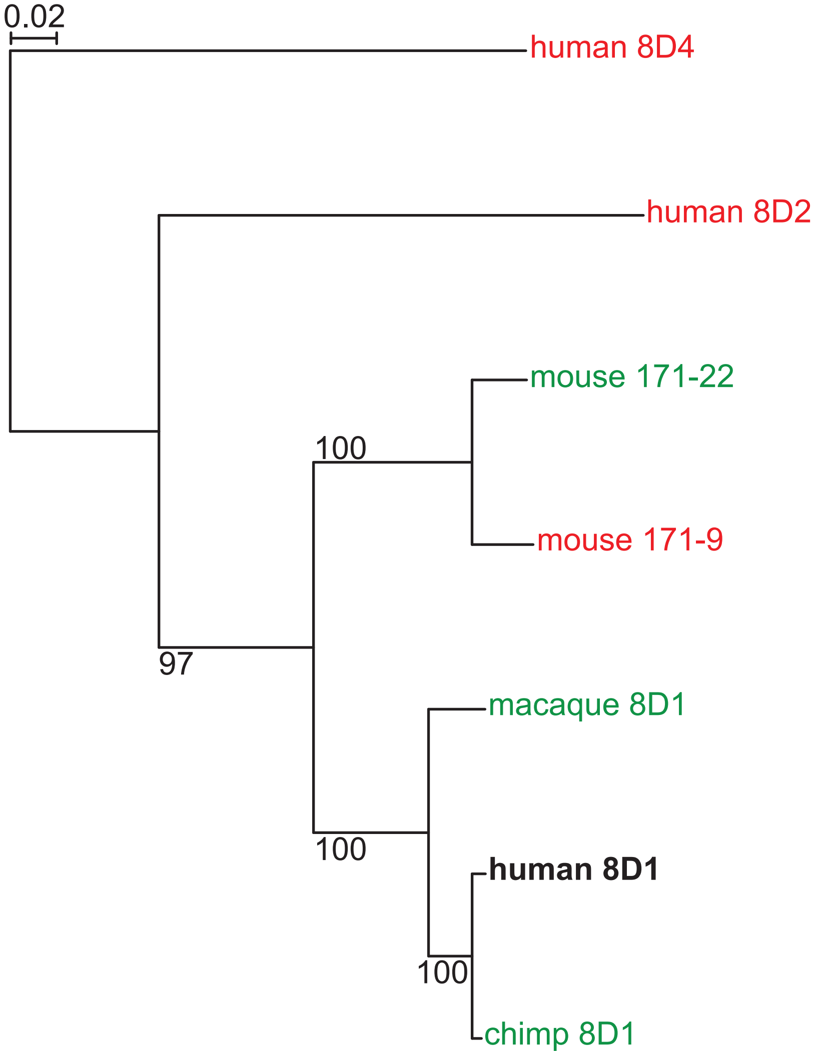

0.02
human 8D4
human 8D2
mouse 171-22
100
mouse 171-9
97
macaque 8D1
100
human 8D1
100
chimp 8D1

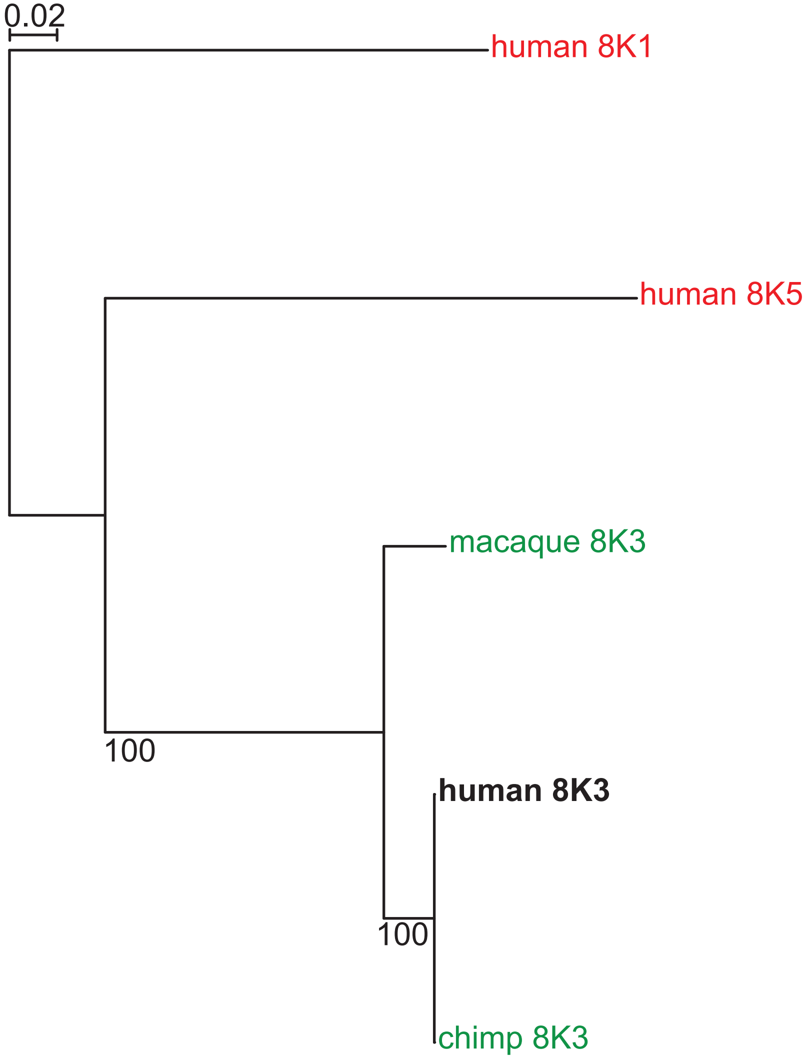
0.02
human 8K1
human 8K5
macaque 8K3
100
human 8K3
100
chimp 8K3
