# Supplementary material for: Functional Evolution of Mammalian Odorant Receptors
Source: PLoS Genet. 2012 Jul 12;8(7):e1002821. doi: 10.1371/journal.pgen.1002821 (PMC3395614; doi:10.1371/journal.pgen.1002821)
Supplement: Figure S12 — Alignment of 22 amino acid positions in orthologs and paralogs predicted to be involved in ligand binding. Alignment of corresponding 22 amino acids [17] from our orthologs and paralogs. Amino acid color categories: KR, red; AFILMVW, blue; NQST, green; HY, teal; C, salmon; DE, purple; P, yellow; G, orange. See Table S6 for corresponding Grantham's distance. (PDF) [file pgen.1002821.s012.pdf]

h1A1	FMMIGNSYGAHYIMGIFYVTVT
c1A1	FMMIGNSYGAHYIMGIFYVTVT
m1A1	FMMIGNSYGGHYIMGVFYVTVM
h1A2	FMMIAKSYGAHYIMGVFYTTVV
m125-1	FMMIGNSYAAHYIMGVFYVTVT
h2W1	FLYMGSCLSSLLLVGIVYITLT
c2W1	FLYMGSCLSSLLLVGIVYITLT
m2W1	FLYMGSCLSSLLLVGIVYITLT
h2W3	FLFLGGCLGSMLMVAVVYIMLN
h2W5	FLYMGSCVGSMLMHGPPRHHHS
h51E1	IMIHSGSTGMLYHGIAIYFANL
c51E1	IMIHSGSTGMLYHGIAIYFANL
m51E1	IMIHSGSTGMLYHGIAIYFANL
h51E2	LMIHSASTGFLYHGIVMYLGDL
m18-1	IMIHSGSTGMLYHGIAIYFANL
h8K3	YLFLIGLFCSVYSIADLYLSIT
c8K3	YLFLIGLFCSVYSIADLYLSIT
m8K3	YLFLIGLFCSVYSIANLYLSIT
h8K1	YLFEIILFSSLYCISNLYLSVT
h8K5	NLFLIIFFSAFYDSSNLYLSVT
h5K1	CFLCETCFGSHYILSVQYLAIT
c5K1	CFLCETCFGSHYILSVQYLAIT
m5K1	CFLCETCFGSHYILSVQYLAIT
h5K2	CFLCETCFGSHYTLSVQYIAIT
h5K3	CFLCETCFGPEFVLAIQCLAIT
h5K4	CFLCETCFGSHFIISIQYCAIA
m184-3	CFLCETCFGSHYILSIQYLAIT
h2A25	CMFLAHCLGAHFIVAVLYALLS
c2A25	CMFLAHCLGAHFIVAVLYALLS
m2A25	YMFLAHCLGAHFIVAVLYALLS
h2A2	YTYLAVCLGSHFIICVLFASLS
h2A4	YTFSAVCLGSHFIVAGLYALLS
h2A5	YTYMAHCLGAHFIISILFASLS
h2A7	YTFSAVCLGSHFIVAGLYALLS
h2A12	YTYLAICLSAHFILSILFASLS
h2A14	YTYLAHCLSAPFIICILFASLS
h2A42	YTCLGHCLGAHFIICFLFAFLS
m261-1	YTFLAHCLGAHFIVAVLYALLS
h10G3	ILYHGSCFGGQFITIVVYCALT
c10G3	ILYHGSCFGGQFITIVVYCALT
m10G3	ILYHGSCFGGQFITIVVYCALT
m223-5	ILYHGSCFGGQFITIVVYCALT
h10G4	FLFHGSCFGSQFAIIVAFCAIT
h10G6	ILFHGCCFGSQFITILAYCAVT
h10G7	FLFHGSCFGSQFAIIVAFGAVT
c10G7	FLFHGSCFGSQFAIIVAFGAVT
m10G7	LLFHGSCFGSQFAIIVAFGAIT
m223-3	FLFHGSCFGSQFAIIVAFGAVT
h10G8	FLFHGGCFGSQLAIVVAFGAVT
h10G9	FLFHGSCFGSQLAIIVAFCAIT

h10J5	YMFVATCFGAHFINSVICASVT
c10J5	YMFVATCFGAHFINSVICASVT
m267-13	YMFVATCFGAHFINSVIYASVT
h10J1	YMFVGICFGAQFITSVLYASVT
h10J3	YLYLGICFGAQFVNSVLYASVT
h8D1	YLFVVVGYGAHFVLANTFISVT
c8D1	YLFVVVGYGAHFVLANTFISVT
m8D1	YLFVVVGYGAHFVLANTFISVT
h8D2	YLFLVIGYGAHFILGNTFISVI
h8D4	YLFCVICYGAHFIIGNMYLSVT
m171-22	YLFVVVGYGAHFVLANTFISVT
m171-9	YLFVVVGYGAHFVLGNTFISVT
h2B11	YYFHGCCIGSQFVLVFVYASLS
c2B11	YYFHGCCIGSQFVLVFVYASLS
m2B11	YYFHGCCIGSQFVLVFVYASLS
h2B2	YLFLGSCLGSQFVLSFLYASLG
h2B3	YLFLGACLGSQFVLSILYASLG
h2B6	YLFLGAYLGSLLVLSFHYASLG
h2B8	YLSLGSCVGSQLVLSILYASLT
h56A4	LMMNLTSCNSVINQGLLSLNIH
c56A4	LMMNLTSCNSVINQGLLSLNIH
m56A4	LMINLTSCNSVINQGLLSLNIH
h56A5	LMMNLTSCNTIINQGLLTLNIH
h56A1	LMMNLPSCNTIINQGLLSLNVH
h56A3	LMMNLASCNTIINQGLLSLNVH
h5P3	YLVVGTCFGAFFYPSIIYISVT
c5P3	YLVVGTCFGAFFYPSIIYISVT
h5P2	YLAAATCVGAYFFLSIIYISVT
m204-6	YLVVGSCFGAFFYPSIIYISVT
h2J2	YLVLGICVGSHFVLSFVFVALT
c2J2	YLVLGICVGSHFVLSFVFVALT
h2J1	YLVLGTCVGSHFVLSFVFVALT
m256-18	YLVLGTCVGSHFVLSFVFIALT
h2J3	YLVLGTCVGSHFVLSFVFAALT
c2J3	YLVLGTCVGSHFVLSFVFAALT
m2J3	YLVLGTCVGSHFVLSFVFAALT
