# Supplementary material for: Functional Evolution of Mammalian Odorant Receptors
Source: PLoS Genet. 2012 Jul 12;8(7):e1002821. doi: 10.1371/journal.pgen.1002821 (PMC3395614; doi:10.1371/journal.pgen.1002821)
Supplement: Table S1 — Receptor Sequences. FASTA file of the amino acid sequences of the ORs used in all functional experiments. (PDF) [file pgen.1002821.s014.pdf]

>h1A1
MRENNQSSTLEFILLGVTGQQEQEDFFYILFLFIYPITLIGNLLIVLAICSDVRLHNPMY
FLLANLSLVDIFFSSVTIPKMLANHLLGSKSISFGGCLTQMYFMIALGNTDSYILAAMAY
DRAVAISRPLHYTTIMSPRSCIWLIAGSWVIGNANALPHTLLTASLSFCGNQEVANFYCD
ITPLLKLSCSDIHFHVKMMYLGVGIFSVPLLCIIVSYIRVFSTVFQVPSTKGMLKAFSTC
GSHLTVVSLYYGTVMGTYFRPLTNYSLKDAVITVMYTAVTPMLNPFIYSLRNRDMKAALR
KLFNKRISS
>c1A1
MRENNQSSTLEFILLGVTGQQEQEDFFYILFLFIYPITLIGNLLIVLAICSDVHLHNPMY
FLLANLSLVDIFFSSVTIPKMLANHLLGSKSISFGGCLTQMYFMIALGNTDSYILAAMAY
DRAVAISRPLHYTTIMSPRSCIWLIAGSWVIGNANALPHTLLTASLSFCGNQEVANFYCD
ITPLLKLSCSDIHFHVKMMYLGVGIFSVPLLCIIVSYIRVFSTVFQVPSTKGVLKAFSTC
GSHLTVVSLYYGTVMGMYFRPLTNYSLKDAVITVMYTAVTPMLNPFIYSLRNRDVKAALR
KLFNKRISS
>m1A1
MRENNQSSTLEFILLGVTGQQEQEDFFYILFLFIYPITLIGNLLIILAIRSDVCLHNPMY
FFLSNLSLVDIFFSSVTIPKMLANHLLGSKSISFEGCLTQMYFMIALGNTDSYILAAMAY
DRAVAISRPLHYTAIMSPRSCVLLVVGSWVIGNANGLPHTLLTASLSFCGNQEVANFYCD
ITPLLKLSCSDIHFNVKMMYVGVGVFSVPLLCIIVSYVRVFSTVFQVPSTKGVLKAFSTC
GSHLTVVSLYYGTVMGMYFRPLTNYSLKDAVITVMYMAVTPMLNPFIYSLRNRDMKAALR
KLFNKRISS
>h1A2
MKKENQSFNLDFILLGVTSQQEQNNVFFVIFLCIYPITLTGNLLIILAICADIRLHNPMY
FLLANLSLVDIIFSSVTIPKVLANHLLGSKFISFGGCLMQMYFMIALAKADSYTLAAMAY
DRAVAISCPLHYTTIMSPRSCILLIAGSWVIGNTSALPHTLLTASLSFCGNQEVANFYCD
IMPLLKLSCSDVHFNVKMMYLGVGVFSLPLLCIIVSYVQVFSTVFQVPSTKSLFKAFCTC
GSHLTVVFLYYGTTMGMYFRPLTSYSPKDAVITVMYVAVTPALNPFIYSLRNWDMKAALQ
KLFSKRISS
>m125-1
MREENESSTIDFTLLGVTRQREQEYFFFILFLFIYPITVFGNMLIILAIHSDTRLHNPMY
FFLANLSLVDIFFSSVTIPKMLANHLLGSKAISFGGCMAQMYFMIGLGNTDSYILAAMAY
DRAVAISRPLHYATIMSPQLCVLLVAGSWVIANANALPHTLLTARLSFCGNKDVANFYCD
ITPLLQLSCSDIRFNVKMMYLGVGVFSVPLLCIIISYVRVFSTVLRVPSTKGFLKALSTC
GSHLTVVSLYYGTVMGMYFRPLTSYSLKHALITVMYTAVTPMLNPFIYSLRNRDMKAALK
KLFHCPSSSSSLM
>h2W1
MDQSNYSSLHGFILLGFSNHPKMEMILSGVVAIFYLITLVGNTAIILASLLDSQLHTPMY
FFLRNLSFLDLCFTTSIIPQMLVNLWGPDKTISYVGCIIQLYVYMWLGSVECLLLAVMSY
DRFTAICKPLHYFVVMNPHLCLKMIIMIWSISLANSVVLCTLTLNLPTCGNNILDHFLCE
LPALVKIACVDTTTVEMSVFALGIIIVLTPLILILISYGYIAKAVLRTKSKASQRKAMNT
CGSHLTVVSMFYGTIIYMYLQPGNRASKDQGKFLTLFYTVITPSLNPLIYTLRNKDMKDA
LKKLMRFHHKSTKIKRNCKS
>c2W1
MDQSNYSSLHGFILLGFSDHPKMEMILSGVVAIFYLITLVGNTVIILASLLDSQLHTPMY
FFLRNLSFLDLCFTTSIIPQMLVNLWGPDKTISYVGCIIQLYVYMWLGSVECLLLAVMSY
DRFTAICKPLHYFVVMNPHLCLKMIIMMWSISLANSVVLCTLTLNLPTCGNKLLDHFLCE
LPALVKIACVDTTTVEMSVFALGIIIVLTPLILILISYGYIAKAVLRMKSKASQRKAMNT
CGSHLTVVSIFYGTIIYMYLQPGNRASKDQGKFLTLFYTIITPSLNPLIYTLRNKDMKDA
LKKLMRFHHKSTKIKRNCKS
>m2W1
MDQRNYSSLHGFILLGFSDHPKMEMILSGVVAVFYLITLVGNTAIILASLLDSQLHTPMY
FFLRNLSFLDLCFTTSIIPQMLVNLWGPDKTISYVGCIIQLYVYMWLGSIECLLLAVMSY
DRFTAICKPLHYFVIMNPHLCLKMIIMVWSISMANSVVLCTLTLNLPTCGNNLLDHFLCE
LPALVKIACVDTTTVEMSVFTLGIIIVLTPLILILISYGYIAKAVLRMKSKAGQRKAMNT
CGSHLTVVSIFYGTIIYMYLQPGNSASKDQGKFLTLFYTIITPSLNPLIYTLRNKDMKDA
LKKLMRFHHKSTKIKRNCKS
>h2W3
MDGTNGSTQTHFILLGFSDRPHLERILFVVILIAYLLTLVGNTTIILVSRLDPHLHTPMY
FFLAHLSFLDLSFTTSSIPQLLYNLNGCDKTISYMGCAIQLFLFLGLGGVECLLLAVMAY
DRCVAICKPLHYMVIMNPRLCRGLVSVTWGCGVANSLAMSPVTLRLPRCGHHEVDHFLRE
MPALIRMACVSTVAIDGTVFVLAVGVVLSPLVFILLSYSYIVRAVLQIRSASGRQKAFGT
CGSHLTVVSLFYGNIIYMYMQPGASSSQDQGMFLMLFYNIVTPLLNPLIYTLRNREVKGG

TGKVASGEERARKGVSGRFEQTV
>h2W5
MGKDNASYLQAFILVGSSDRPGLEKILFAVILIFCILTLVGNTAIILLLVMDVRLHTPMY
FFLGNLSFLDLCFTASIAPQLLWNLGGPEKTITYHGCVAQLYIYMMLGSTECVLLVVMSH
DRYVAVCRSLHYMAVMRPHLCLQLVTVAWCCGFLNSFIMCPQTMQLSRCGRRRVDHFLCE
MPALIAMSCEETMLVEAIHLCPGGGSPPGAALPHPHLLWRDCSRGAEDEVSSRAKESLPH
LLFSPHSGLSLLRNHHLRVPEAGQQLLPRSGEVPDSLLHHRHSQHQPPHLHFEEQGCEGD
HEETSGVGERGWGASTRGTL
>h51E1
MMVDPNGNESSATYFILIGLPGLEEAQFWLAFPLCSLYLIAVLGNLTIIYIVRTEHSLHE
PMYIFLCMLSGIDILISTSSMPKMLAIFWFNSTTIQFDACLLQMFAIHSLSGMESTVLLA
MAFDRYVAICHPLRHATVLTLPRVTKIGVAAVVRGAALMAPLPVFIKQLPFCRSNILSHS
YCLHQDVMKLACDDIRVNVVYGLIVIISAIGLDSLLISFSYLLILKTVLGLTREAQAKAF
GTCVSHVCAVFIFYVPFIGLSMVHRFSKRRDSPLPVILANIYLLVPPVLNPIVYGVKTKE
IRQRILRLFHVATHASEP
>c51E1
MMVDPNGNESSATYFILIGLPGLEEAQFWLAFPLCSLYLIAVLGNLTIIYIVRTEHSLHE
PMYIFLCMLSGIDILISTSSMPKMLAIFWFNSTTIQFDACLLQMFAIHSLSGMESTVLLA
MAFDRYVAICHPLRHATVLTLPRVTKIGVAAVVRGAALMAPLPVFIKQLPFCRSNILSHS
YCLHQDVMKLACDDIRVNVVYGLIVIISAIGLDSLLISFSYLLILKTVLGLTREAQAKAF
GTCVSHVCAVFIFYVPFIGLSMVHRFSKRRDSPLPIILANIYLLVPPVLNPIVYGVKTKE
IRQRILRLFHVATHASET
>m51E1
MMVGPNGNESSATYFILIGLPGLEEAQFWLAFPLCSLYLIAVLGNLTIIYVVRAEHSLHE
PMYIFLCMLSGIDILISTSSMPKMLAIFWFNSTTIQFDACLLQMFAIHSLSGMESTVLLA
MAFDRYVAICHPLRHATVLTLPRVTKIGVAAVVRGAALMAPLPVFIKQLPFCRSNILSHS
YCLHQDVMKLACDDIQVNVIYGLIVIISAIGLDSLLISFSYLLILKTVLGLTREAQAKAF
GTCVSHVCAVFIFYVPFIGLSMVHRFSKRRDSLLPVILANIYLLVPPVRAASS
>h51E2
MSSCNFTHATFVLIGIPGLEKAHFWVGFPLLSMYVVAMFGNCIVVFIVRTERSLHAPMYL
FLCMLAAIDLALSTSTMPKILALFWFDSREISFEACLTQMFFIHALSAIESTILLAMAFD
RYVAICHPLRHAAVLNNTVTAQIGIVAVVRGSLFFFPLPLLIKRLAFCHSNVLSHSYCVH
QDVMKLAYADTLPNVVYGLTAILLVMGVDVMFISLSYFLIIRTVLQLPSKSERAKAFGTC
VSHIGVVLAFYVPLIGLSVVHRFGNSLHPIVRVVMGDIYLLLPPVINPIIYGAKTKQIRT
RVLAMFKISCDKDLQAVGGK
>m18-1
MVGFNSNESSATYFILIGLPGLEEVQFWLAFPLCSLYLIAVLGNLTIIYIVRTEHSLHEP
MYIFLCMLSGLDILISTSSMPKMMAIFWFNSTTIQFDACLVQMFAIHSLSGMESTVLLAM
AFDRYVAICHPLRHATVLTLPRVAKIGMAAVVRGAVLMAPLPVFIKRLPFCRSNILSHSY
CLHQDVMKLACADIRVNIIYGLIVIISAIGLDSLLISFSYLLILKTVLGLTREAQAKAFG
TCVSHVCAVFIFYVPFIGLSMVHRFSKRRDSLLPVIMANIYLLVPPVLNPIVYGVKTKEI
RQRILRLFLVTTHTSDH
>h8K3
MEQHNLTTVNEFILTGITDIAELQAPLFALFLMIYVISVMGNLGMIVLTKLDSRLQTPMY
FFLRHLAFMDLGYSTTVGPKMLVNFVVDKNIISYYFCATQLAFFLVFIGSELFILSAMSY
DRYVAICNPLLYTVIMSRRVCQVLVAIPYLYCTFISLLVTIKIFTLSFCGYNVISHFYCD
SLPLLPLLCSNTHEIELIILIFAAIDLISSLLIVLLSYLLILVAILRMNSAGRQKAFSTC
GAHLTVVIVFYGTLLFMYVQPKSSHSFDTDKVASIFYTLVIPMLNPLIYSLRNKDVKYAL
RRTWNNLCNIFV
>c8K3
MEQHNLTTVNEFILTGITDIAELQAPLFALFLMIYVISVMGNLGMIVLTKLDSRLQTPMY
FFLRHLAFMDLGYSTTVGPKMLVNFVVDKNIISYYFCATQLAFFLVFIGSELFILSAMSY
DRYVAICNPLLYTVIMSRRVCQVLVAIPYLYCTFISLLVTIKIFTLSFCGYNVISHFYCD
SLPLLPLLCSNTHEIELIILIFAAIDLISSLLIVLLSYLLILVAILRMNSAGRQKAFSTC
GAHLTVVIVFYGTLLFMYVQPKSSHSFDTDKVASIFYTLVIPMLNPLIYSLRNKDVKYAL
RRTWNNLCNIFV
>m8K3
MEQHNLTTVNEFILAGITNIAELQAPLFALFLMIYVISVMGNLGMIVLTKLDSRLQTPMY
FFLRHLALMDLGYSTTVGPKMLVNFVVDKNTISYYFCATQLACFLVFIGSELFILSAMSY
DRYVAICNPLLYTVIMSQKVCQVLVAIPYLYCTFISLLVTVKIFTLSFCGCNVIRHFYCD
SLPLLPLLCSNAHEIELIILIFAAINLISSLLIVLVSYLLILVAILRMNSAGRQKAFSTC
GAHLTVVIVFYGTLLFMYVQPKSSHSFDTDKVASIFYTLVIPMLNPLIYSLRNKDVKYAL

WRTWNNLMFFFKVCTI
>h8K1
MNHVVKHNHTAVTKVTEFILMGITDNPGLQAPLFGLFLIIYLVTVIGNLGMVILTYLDSK
LHTPMYFFLRHLSITDLGYSTVIAPKMLVNFIVHKNTISYNWYATQLAFFEIFIISELFI
LSAMAYDRYVAICKPLLYVIIMAEKVLWVLVIVPYLYSTFVSLFLTIKLFKLSFCGSNII
SYFYCDCIPLMSILCSDTNELELIILIFSGCNLLFSLSIVLISYMFILVAILRMNSRKGR
YKAFSTCSSHLTVVIMFYGTLLFIYLQPKSSHTLAIDKMASVFYTLLIPMLNPLIYSLRN
KEVKDALKRTLTNRFKIPI
>h8K5
MGQHNLTVLTEFILMELTRRPELQIPLFGVFLVIYLITVVGNLTMIILAKLDSHLHTPMY
FSIRHLAFVDLGNSTVICPKVLANFVVDRNTISYYACAAQLAFFLMFIISEFFILSAVAY
DRYVAICNPLLYYVIMSQRLCHVLVGIQYLYSTFQALMFTIKIFTLTFCGSNVISHFYCD
DVPLLPMLCSNAQEIELLSILFSVFNLISSFLIVLVSYMLILLAICQMHSAEGRKKAFST
CGSHLTVVVVFYGSLLFMYMQPNSTHFFDTDKMASVFYTLVIPMLNPLIYSLRNEEVKNA
FYKLFEN
>h5K1
MAEENHTMKNEFILTGFTDHPELKTLLFVVFFAIYLITVVGNISLVALIFTHRRLHTPMY
IFLGNLALVDSCCACAITPKMLENFFSENKRISLYECAVQFYFLCTVETADCFLLAAMAY
DRYVAICNPLQYHIMMSKKLCIQMTTGAFIAGNLHSMIHVGLVFRLVFCGSNHINHFYCD
ILPLYRLSCVDPYINELVLFIFSGSVQVFTIGSVLISYLYILLTIFKMKSKEGRAKAFST
CASHFLSVSLFYGSLFFMYVRPNLLEEGDKDIPAAILFTIVVPLLNPFIYSLRNREVISV
LRKILMKK
>c5K1
MTEENHTMKNEFILMGFTDHPELKTLLFVVFFAIYLITVVGNISLVALIFTHRRLHTPMY
IFLGNLALVDSCCACAITPKMLENFFSENKRISLYECAVQFYFLCTVETADCFLLAAMAY
DRYVAICNPLQYHIMMSKKLCIQMTTGAFIAGNLHSMIHVGLVFRLVFCGSNHINHFYCD
ILPLYRLSCVDPCINELVLFIFSGSVQVFTIGSVLISYLYILLTIFKMKSKEGRAKAFST
CASHFLSVSLFYGSLFFMYVRPNLLEEGDKDIPAAILFTIVVPLLNPFIYSLRNREVISV
LRKILMKK
>m5K1
MAEENHTMKNEFILTGFTDHPELKTLLFVVFFAIYLITMVGNIGMMALIFTHRRLHTPMY
IFLGNLALVDSCCACAITPKMLENFFSEDKRISLYECTVQFYFLCTVETADCFLLAAMAY
DRYVAICSPLQYHIMMSKKLCIQMTTGAFIAGNLHSMIHVGLVFRLVFCGSNHINHFYCD
ILPLYRLSRVDPFINELVLFIFSGSVQVFTIGSVLISYLYILLTIFRMKSKEGRAKAFST
CASHFLSVSLFYGSLFFMYVRPNLLEEGDKDIPAAILFTIVVPLLNPFIYSLRNREVISV
LRKILMKK
>h5K2
MVEENHTMKNEFILTGFTDHPELKTLLFVVFFAIYLITVVGNISLVALIFTHCRLHTPMY
IFLGNLALVDSCCACAITPKMLENFFSEGKRISLYECAVQFYFLCTVETADCFLLAAVAY
DRYVAICNPLQYHIMMSKKLCIQMTTGAFIAGNLHSMIHVGLVFRLVFCGLNHINHFYCD
TLPLYRLSCVDPFINELVLFIFSGSVQVFTIGSVLISYLYILLTIFRMKSKEGRAKAFST
CASHFSSVSLFYGSIFFLYIRPNLLEEGGNDIPAAILFTIVVPLLNPFIYSLRNKEVISV
LRKILLKIKSQGSVNK
>h5K3
MNKENHSLIAEFILTGFTYHPKLKTVLFVVFFAIYLITMVGNIGLVALIYIEQRLHTPMY
IFLGNLALMDSCCSSAITPKMLENFFSEDKRITLYECMAQFYFLCLAETTDCFLLAAMAY
DRYVAICNPLQYHTMMSKTLCIQMTAGAYLAGNLHPMIEVEFLLRLTFCGSHQISHLFCD
VLPLYRLSCINPYINELVLFILAGSIQIFTIVLVSYFYILFTIFTMKSKEGRGKALSTCA
SHFLSVSIFCDSLLFMYARPGAVNEADKDIPVAIFYTLVIPLLNPFIYSLRNKEVINIMK
KIMKKRKFCHILKQMSSPLAT
>h5K4
MARENHSLAAEFILIGFTNYPELKTLLFVVFSAIYLVTMVGNLGLVALIYVERRLLTPMY
IFLGNLALMDSCCSCAVTPKMLENFFSEDRIISLYECMAQFYFLCLAETTDCFLLATMAY
DRYVAICHPLQYHTMMSKTLCIRMTTGAFKAGNLHSMIHVGLLLRLTFCRSNKIHHFFCD
ILPLYRLSCTDPSINELMIYIFSIPIQIFTIATVLISYLCILLTVFKMKSKEGRGKAFST
CSSHFLSVSIFYICLLMYIGPSEEGDKDTPVAIFYAIVIPLLNPFIYSLRNKEVINVLKK
IMRNYNILKQTCSIANLFLIY
>m184-3
MVEENHTMKREFVLTGFTDHPEMKGLLFAVFFFIYLITMIGNMGLVILISKERSLHTPMY
IFLGNLAFIDSCCACAITPKMLENFFSEDRIISLYECMAQFYFLCTVETADCFLLSAMAY
DRYVAICNPLQYHTTMSKKLCLQMTTGAFIAGNLHSMVHVGLLFRLAFCGSNQINHFYCD
ILPLYRLSCVDPYINELVLFVFSGSIQVFTIGCVLISYLFIVYTIFQMKSKEGRIKAFST

CASHFLSVSLFYGSLFFMYIRPNLLEEGDKDMPAAILFTIVVPLLNPFIYSLRNKEVKNV
LQKILQKKIISKNFKQASIIV
>h2A25
MGGNQTSITEFLLLGFPIGPRIQMLLFGLFSLFYIFILLGNGTILGLISLDSRLHTPMYF
FLSHLAVVDIACACSTVPQMLVNLLHPAKPISFAGCMTQMFLFLSFAHTECLLLVVMSYD
RYVAICHPLRYSTIMTWKVCITLALTSWILGVLLALVHLVLLLPLSFCGPQKLNHFFCEI
MAVLKLACADTHINEVMVLAGAVSVLVGAFFSTVISYVHILCAILKIQSGEGCQKAFSIC
SSHLCVVGLFYGTAIIMYVEPQYESPKEQKKYLLLFHSLFNPMLNPLIYSLRNKEVQGTL
KRMLEKKRTS
>c2A25
MGGNQTSITEFLLLGFPIGPRIQMLLFGLFSLFYIFILLGNGTILGLISLDSRLHTPMYF
FLSHLAVVDIACACNTVPQMLVNLLHPAKPISFAGCMTQMFLFLNFAHTECLLLVVMSYD
RYVAICHPLRYSTIMTWKVCITLALTSWILGVLLALVHLVLLLPLSFCGPQKLNHFFCEI
MAVLKLACADTHINEVMVLAGAVSVLVGPFFSTVISYVHILCAILKIQSGEGCQKAFSIC
SSHLCVVGLFYGTAIIMYVEPQYESPKEQKKYLLLFHSLFNPMLNPLIYSLRNKEVQGTL
KRMLEKKRTS
>m2A25
MGGNQTSITEFLLLGFPVGPRIQMLLFGLFSLFYVFTLLGNGTILGLISLDSRLHTPMYF
FLSHLAVVDIAYACNTVPQMLVNLLHPAKPISFAGCMTQMFLFLSFAHTECLLLVLMSYD
RYVAICHPLRYSTIMTWRVCITLALTSWTLGVLLALVHLMLLLSLSFCGPQKVNHFFCEI
TAVLKLACVDTHINEVMVLAGALSVLVGPFFSIVISYVHILCAILKIQSGEGRQKAFSIC
SSHLCVAGFFYGTAIIMYVEPQYESPKEQKKYLLLFHSLFNPMLNPLIYSLRNKEVQGVL
KRMLVKERTS
>h2A2
MEGNQTWITDITLLGFQVGPALAILLCGLFSVFYTLTLLGNGVIFGIICLDSKLHTPMYF
FLSHLAIIDMSYASNNVPKMLANLMNQKRTISFVPCIMQTFLYLAFAVTECLILVVMSYD
RYVAICHPFQYTVIMSWRVCTILVLTSWSCGFALSLVHEILLLRLPFCGPRDVNHLFCEI
LSVLKLACADTWVNQVVIFATCVFVLVGPLSLILVSYMHILGAILKIQTKEGRIKAFSTC
SSHLCVVGLFFGIAMVVYMVPDSNQREEQEKMLSLFHSVLNPMLNPLIYSLRNAQLKGAL
HRALQRKRSMRTVYGLCL
>h2A4
MGDNITSIREFLLLGFPVGPRIQMLLFGLFSLFYVFTLLGNGTILGLISLDSRLHAPMYF
FLSHLAVVDIAYACNTVPRMLVNLLHPAKPISFAGRMMQTFLFSTFAVTECLLLVVMSYD
LYVAICHPLRYLAIMTWRVCITLAVTSWTTGVLLSLIHLVLLLPLPFCRPQKIYHFFCEI
LAVLKLACADTHINENMVLAGAISGLVGPLSTIVVSYMCILCAILQIQSREVQRKAFRTC
FSHLCVIGLVYGTAIIMYVGPRYGNPKEQKKYLLLFHSLFNPMLNPLICSLRNSEVKNTL
KRVLGVERAL
>h2A5
MTKNQTWVTEFILLGFPLSLRIQMLLSGLFSLLYVFTLLGNGAILGLIWLDSRLHTPMYF
FLSHLAIIDISYASNNVPKMLTNLGLNKRKTISFVPCTMQTFLYMAFAHTECLILVMMSY
DRYMAICHPLQYSVIMRWGVCTVLAVTSWACGSLLALVHVVLILRLPFCGPHEINHFFCE
ILSVLKLACADTWLNQVVIFAASVFILVGPLCLVLVSYSRILAAILRIQSGEGRRKAFST
CSSHLCMVGLFFGSAIVMYMAPKSRHPEEQQKVLSLFYSLFNPMLNPLIYSLRNAEVKGA
LKRVLWKQRSK
>h2A7
MGDNITSIREFLLLGFPVGPRIQMLLFGLFSLFYVFTLLGNGTILGLISLDSRLHAPMYF
FLSHLAVVDIAYACNTVPRMLVNLLHPAKPISFAGRMMQTFLFSTFAVTECLLLVVMSYD
LYVAICHPLRYLAIMTWRVCITLAVTSWTTGVLLSLIHLVLLLPLPFCRPQKIYHFFCEI
LAVLKLACADTHINENMVLAGAISGLVGPLSTIVVSYMCILCAILQIQSREVQRKAFCTC
FSHLCVIGLFYGTAIIMYVGPRYGNPKEQKKYLLLFHSLFNPMLNPLICSLRNSEVKNTL
KRVLGVERAL
>h2A12
MESNQTWITEVILLGFQVDPALELFLFGFFLLFYSLTLMGNGIILGLIYLDSRLHTPMYV
FLSHLAIVDMSYASSTVPKMLANLVMHKKVISFAPCILQTFLYLAFAITECLILVMMCYD
RYVAICHPLQYTLIMNWRVCTVLASTCWIFSFLLALVHITLILRLPFCGPQKINHFFCQI
MSVFKLACADTRLNQVVLFAGSAFILVGPLCLVLVSYLHILVAILRIQSGEGRRKAFSTC
SSHLCVVGLFFGSAIVMYMAPKSSHSQERRKILSLFYSLFNPILNPLIYSLRNAEVKGAL
KRVLWKQRSM
>h2A14
MEGNKTWITDITLPRFQVGPALEILLCGLFSAFYTLTLLGNGVIFGIICLDCKLHTPMYF
FLSHLAIVDISYASNYVPKMLTNLMNQESTISFFPCIMQTFLYLAFAHVECLILVVMSYD
RYADICHPLRYNSLMSWRVCTVLAVASWVFSFLLALVPLVLILSLPFCGPHEINHFFCEI

LSVLKLACADTWLNQVVIFAACVFILVGPLCLVLVSYLRILAAILRIQSGEGRRKAFSTC
SSHLCVVGLFFGSAIVTYMAPKSRHPEEQQKVLSLFYSLFNPMLNPLIYSLRNAEVKGAL
RRALRKERLT
>h2A42
MVTEFLLLGFLLGPRIQMLLFGLFSLFYIFTLLGNGAILGLISLDSRLHTPMYFFLSHLA
VVDIAYTRNTVPQMLANLLHPAKPISFAGCMTQTFLCLSFGHSECLLLVLMSYDRYVAIC
HPLRYSVIMTWRVCITLAVTSWTCGSLLALAHVVLILRLPFSGPHEINHFFCEILSVLRL
ACADTWLNQVVIFAACVFFLVGPPSLVLVSYSHILAAILRIQSGEGRRKAFSTCSSHLCV
VGLFFGSAIIMYMAPKSRHPEEQQKVFFLFYSSFNPMLNPLIYNLRNVEVKGALRRALCK
ESHS
>m261-1
MGGNQTLITQFILLGFPLSPRMQMLLFALFSLFYAFTLLGNGTILGLICLDSRLHTPMYF
FLSHLAIVDIAYACNTVPQMLVNLMDPAKPISFAGCMTQTFLFLTFAHTECLLLVVMSYD
RYVAICHPLRYTAIMSWRVCVILVLTSWILGVLLALVHLVLLLPLPFCGSQKVNHFFCEI
IAVLKLACSDTRINELMVLAGAVSVLVGPFSSIVVSYAHILCAILKIKSQQGRQKAFSTC
SSHLCVVGLFYGTAIVMYIGPQHGKSNEQKKYLLLFHSLFNPMLNPLIYSLRNKEVKSAL
KRTLLKEDTS
>h10G3
MERINSTLLTAFILTGIPYPLRLRTLFFVFFFLIYILTQLGNLLILITVWADPRLHARPM
YIFLGVLSVIDMGISSIIVPRLMMNFTLGVKPIPFGGCVAQLYFYHFLGSTQCFLYTLMA
YDRYLAICQPLRYPVLMTAKLSALLVAGAWMAGSIHGALQAILTFRLPYCGPNQVDYFFC
DIPAVLRLACADTTVNELVTFVDIGVVVASCFSLILLSYIQIIQAILRIHTADGRRRAFS
TCGAHVTVVTVYYVPCAFIYLRPETNSPLDGAAALVPTAITPFLNPLIYTLRNQEVKLAL
KRMLRSPRTPSEV
>c10G3
MERINSTLLTAFILTGIPYPLRLRTLFFVFFLLIYILTQLGNLLILITVWADPRLHARPM
YIFLGVLSVIDMGISSIIVPRLMMNFTLGVKPIPFGGCVAQLYFYHFLGSTQCFLYTLMA
YDRYLAICQPLRYPVLMTAKLSALLVAGAWMAGSIHGALQAILTFRLPYCGPNQVDYFFC
DIPAVLRLACADTTVNELVTFVDIGVVVASCFSLILLSYIQIIQAILRIHTADGRRRAFS
TCGAHVTVVTVYYVPCAFVYLRPETNSPLDGAAALFPTAITPFLNPLIYTLRNQEVKLAL
KRMLRSPRTPSEV
>m10G3
MERVNNTLLTAFILTGIPYPLRLRTLFFVFFLLIYILTQLGNLLIFITVWADPRLHAHPM
YIFLGVLSVIDMGISSIIVPRLMMNFTLGVKSIPFGGCVAQLYFYHFLGSTQCFLYTLMA
YDRYLAICQPLRYPVLMTAKLSALLVAGAWVAGSIHGALQAILTFRLPYCGPNQVDYFFC
DIPAVLRLACADTTVNEMVTFVDIGVVVASCFFLILLSYIQIIQAILRIHTADGRRRAFS
TCGAHVTVVTVYYVPCAFIYLRPETNSPLDGAAALLPTAITPFLNPLIYTLRNQEVKLAL
KTMLRSPRTMSEV
>m223-5
MERINYTVLTEFILTGVPHPPRLRTFLFVFFLLIYILTQLGNALILITVCADTQLHARPM
YIFLGALSVIDMGISTIIVPRLMMNFTPGIKPIPFGGCVAQLYFYHFLGSSQCFLYTTMA
YDRYLAICQPLRYPVLMSAKLSILLVAGAWVAGSIHGAIQAILTFRLPYCGPNQVDYFFC
DIPAVLKLACADTTVNELVTFVDIGVVVASCFSLILLSYIYIIRAILRIRTADGRRRAFS
TCGAHVTIVTVYYVPCAFIYLRPDSHSILDGAAALFPTAITPFLNPLIYTLRNQEVKLAL
RRMVGSQSTKSEV
>h10G4
MSNASLVTVFILTGLPHAPGLDALLFGIFLVVYVLTVLGNLLILLVIRVDSHLHTPMYYF
LTNLSFIDMWFSTVTVPKMLMTLVSPSGRAISFHSCVAQLYFFHFLGSTECFLYTVMSYD
RYLAISYPLRYTSVMSGSRCALLATGTWLSGSLHSAVQTILTFHLPYCGPNQIQHYFCDA
PPILKLACADTSANEMVIFVDIGIVASGCFVLIVLSYVSIVCSILRIRTSDGRRGAFQTC
ASHCIVVLCFFVPCVVIYLRPGSMDAMDGVVAIFYTVLTPLLNPVVYTLRNKEVQKAVLK
LRDKVAHPQRK
>h10G6
MLEGVEHLLLLLLLLTDVNSKELQSGNQTSVSHFILVGLHHPPQLGAPLFLAFLVIYLLTV
SGNGLIILTVLVDIRLHRPMCLFLCHLSFLDMTISCAIVPKMLAGFLLGSRIISFGGCVI
QLFSFHFLGCTECFLYTLMAYDRFLAICKPLHYATIMTHRVCNSLALGTWLGGTIHSLFQ
TSFVFRLPFCGPNRVDYIFCDIPAMLRLACADTAINELVTFADIGFLALTCFMLILTSYG
YIVAAILRIPSADGRRNAFSTCAAHLTVVIVYYVPCTFIYLRPCSQEPLDGVVAVFYTVI
TPLLNSIIYTLCNKEMKAALQRLGGHKEVQPH
>h10G7
MSNATLLTAFILTGLPHAPGLDAPLFGVFLVVYVLTVLGNLLILLVIRVDSHLHTPMYYF
LTNLSFIDMWFSTVTVPKMLMTLVSPSGRTISFHSCVAQLYFFHFLGSTECFLYTVMSYD

RYLAISYPLRYTNMMTGRSCALLATGTWLSGSLHSAVQTILTFHLPYCGPNQIQHYFCDA
PPILKLACADTSANEMVIFVNIGIVASGCFVLIVLSYVSIVCSILRIRTSEGRHRAFQTC
ASHCIVVLCFFGPGLFIYLRPGSRDALHGVVAVFYTTLTPLFNPVVYTLRNKEVKKALLK
LKNGSVFAQG
>c10G7
MSNASLLTAFILMGLPHVQALDAPLFGVFLVVYVLTVLGNLLILLVIRVDSHLHTPMYYF
LTNLSFIDMWFSTVTVPKMLMTLVSPSGRAISFHSCVAQLYFFHFLGSTECFLYTVMSYD
RYLAISYPLRYTSMMSGSRCALLATGTWLSGSLHSAVQTIMTFHLPYCGPNQIQHYFCDA
PPILKLACADTSANEMVIFVNIGVVASGCFVLIVLSYVSIVCSILRIRTSEGRHRAFQTC
ASHCIVVLCFFGPGLFIYLRPGSRDALHGVVAVFYTTLTPLFNPVVYTLRNKEVKKALLK
LKNGSVFAQGE
>m10G7
MSNSSLVTAFILTGLPHAPTLDTPLFGIFLVIYVLTVLGNLLILLVIRVDSHLHTPMYSF
LTNLSFIDMWLSTVTVPKMLMTLASPSGRAISFHSCVAQLYFFHFLGSTECFLYTVMSYD
RYLAISYPLRYTSMMSGRACALLAIGTWLSGSLHSAVQTILTFHLPYCGPNRIQHYFCDA
PPILKLACADTSANEMVIFVNIGVVASGCFLLIVLSYVSIVCSILRIRTSEGRRRAFQTC
ASHGIVVLCFFGPGLFIYLRPGSRDAVDGVVAIFYTTLTPLFNPVVYTLRNKEVKKALLK
LKNGSVFSQGK
>m223-3
MSNTSIVTTFFLSGLPHPPVLDSMLFGIFLVIYILTVLGNLLILTVIRVDSHLHTPMYYF
LTNLSFIDMWFSTVTVPKMLMTLVSTGGGAISFHSCVAQLYCFHFLGSTECFLYTVMSYD
RYLAISYPLRYSSMMGGRMCALLAAGTWFTGSLHSAVQTTLTFHLPYCGPNQIQHYFCDA
PPILKLACADTSANEMVIFVNIGVVASGCFFLISLSYVSIVCSILRIRTSEGRHRAFQTC
ASHCIVVLCFFGPGLFIYLRPGSRDAVDGIVAVFYTVLTPLLNPVVYTLRNKEVKKALLK
IKYGSVLPQDK
>h10G8
MSNASLLTAFILMGLPHAPALDAPLFGVFLVVYVLTVLGNLLILLVIRVDSHLHTTMYYF
LTNLSFIDMWFSTVTVPKLLMTLVFPSGRAISFHSCMAQLYFFHFLGGTECFLYRVMSCD
RYLAISYPLRYTSMMTGRSCTLLATSTWLSGSLHSAVQAILTFHLPYCGPNWIQHYLCDA
PPILKLACADTSAIETVIFVTVGIVASGCFVLIVLSYVSIVCSILRIRTSEGKHRAFQTC
ASHCIVVLCFFGPGLFIYLRPGSRKAVDGVVAVFYTVLTPLLNPVVYTLRNKEVKKALLK
LKDKVAHSQSK
>h10G9
MSKTSLVTAFILTGLPHAPGLDAPLFGIFLVVYVLTVLGNLLILLVIRVDSHLHTPMYYF
LTNLSFIDMWFSTVTVPKMLMTLVSPSGRTISFHSCVAQLYFFHFLGSTECFLYTVMSYD
RYLAISYPLRYTSVMSGSRCALLATSTWLSGSLHSAVQTILTFHLPYCGPNQIQHYLCDA
PPILKLACADTSANEMVIFVDIGLVASGCFLLIVLSYVSIVCSILRIHTSEGRHRAFQTC
ASHCIVVLCFFVPCVFIYLRPGSRDVVDGVVAIFYTVLTPLLNPVVYTLRNKEVKKAVLK
LRDKVAHSQGE
>h10J5
MKRKNFTEVSEFIFLGFSSFGKHQITLFVVFLTVYILTLVANIIIVTIICIDHHLHTPMY
FFLSMLASSETVYTLVIVPRMLLSLIFHNQPISLAGCATQMFFFVILATNNCFLLTAMGY
DRYVAICRPLRYTVIMSKGLCAQLVCGSFGIGLTMAVLHVTAMFNLPFCGTVVDHFFCDI
YPVMKLSCIDTTINEIINYGVSSFVIFVPIGLIFISYVLVISSILQIASAEGRKKTFATC
VSHLTVVIVHCGCASIAYLKPKSESSIEKDLVLSVTYTIITPLLNPVVYSLRNKEVKDAL
CRVVGRNIS
>c10J5
MQRKNFTEVSEFIFLGFSSFGKHQITLFVVFLTVYILTLVANIIIVTIICIDHHLHTPMY
FFLSMLASSETVYTLVIVPRMLLSPIFHNQPISLAGCATQMFFFVILATNNCFLLTAMGY
DRYVAICRPLRYTVIMSKGLCAQLVCGSFGIGPTMAVLHVTAMFNLPFCGTVVDHFFCDI
YPVMKLSCTDTTINEIINYGVSSFVIFVPIGLIFISYVLVISSILQIASAEGRKKTFATC
ASHLTVVIVHCGCASIAYLKLKSESSIEKDLVLSVTYTIITPLLNPVVYSLRNKEVKDAL
CRVVGRNIS
>m267-13
MQRNNFTEVIEFVFLGFSSFGKHQITLFVVFLTIYILTLAGNIIIVTITHIDHHLHTPMY
FFLSMLASSETVYTLVIVPRMLSSLIFYNLPISLAGCATQMFFFVTLATNNCFLLTAMGY
DRYVAICNPLRYTIIMSKGMCALLVCGSLGTGLVMAVLHVPAMFHLPFCGTVVEHFFCDI
YPVMKLSCVDTTVNEIINYGVSSFVILVPIGLIFISYVLIVSSILKIVSTEGQKKAFATC
ASHLTVVIVHYGCASIAYLKPKSESSVEKDLLLSVTYTIITPLLNPVVYSLRNKEVKDAL
CRAVGRNTS
>h10J1
MKRENFTLITDFVFQGFSSFHEQQITLFGVFLALYILTLAGNIIIVTIIRIDLHLHTPMY

FFLSMLSTSETVYTLVILPRMLSSLVGMSQPMSLAGCATQMFFFVTFGITNCFLLTAMGY
DRYVAICNPLRYMVIMNKRLRIQLVLGACSIGLIVAITQVTSVFRLPFCARKVPHFFCDI
RPVMKLSCIDTTVNEILTLIISVLVLVVPMGLVFISYVLIISTILKIASVEGRKKAFATC
ASHLTVVIVHYSCASIAYLKPKSENTREHDQLISVTYTVITPLLNPVVYTLRNKEVKDAL
CRAVGGKFS
>h10J3
MPKLNSTFVTEFLFEGFSSFRRQHKLVFFVVFLTLYLLTLSGNVIIMTIIRLDHHLHTPM
YFFLCMLSISETCYTVAIIPHMLSGLLNPHQPIATQSCATQLFFYLTFGINNCFLLTVMG
YDRYVAICNPLRYSVIMGKRACIQLASGSLGIGLGMAIVQVTSVFGLPFCDAFVISHFFC
DVRHLLKLACTDTTVNEIINFVVSVCVLVLPMGLVFISYVLIISTILKIASAEGQKKAFA
TCASHLTVVIIHYGCASIIYLKLKSQSSLGQDRLISVTYTHHSPTEPCCVQPEEQGGQRC
SAQSRGAKNSVSLMKRGCEGFSFAFINMY
>h8D1
MTMENYSMAAQFVLDGLTQQAELQLPLFLLFLGIYVVTVVGNLGMILLIAVSPLLHTPMY
YFLSSLSFVDFCYSSVITPKMLVNFLGKKNTILYSECMVQLFFFVVFVVAEGYLLTAMAY
DRYVAICSPLLYNAIMSSWVCSLLVLAAFFLGFLSALTHTSAMMKLSFCKSHIINHYFCD
VLPLLNLSCSNTHLNELLLFIIAGFNTLVPTLAVAVSYAFILYSILHIRSSEGRSKAFGT
CSSHLMAVVIFFGSITFMYFKPPSSNSLDQEKVSSVFYTTVIPMLNPLIYSLRNKDVKKA
LRKVLVGK
>c8D1
MTMENYSTAAQFVFDGLTQQAELQLPLFLLFLGIYVVTVVGNLGMILLIAVSPLLHTPMY
YFLSSLSFVDFCYSSVITPKMLVNFLGKKNTILYSECMVQLFFFVVFVVAEGYLLTAMAY
DRYVAICSPLLYNAIMSSWVCSLLVLAAFFLGFLSALTHTSAMMKLSFCKSHIINHYFCD
VLPLLNLSCSNTHLNELLLFIIAGFNTLVPTLAVAVSYAFILYSILHIRSSEGRSKAFGT
CSSHLMAVGIFFGSITFMYFKPPSSNSLDQEKVSSVFYTTVIPMLNPLIYSLRNKDVKKA
LRKVLVGK
>m8D1
MTVENYSTATQFVLAGLTQQAEIQLPLFLLFLGIYLVTVVGNLGMVLLIAISPLLHTPMY
YFLSSLSFVDFCYSSVITPKMLVNFLGKNTILYSECMVQLFFFVVFVVAEGYLLTAMAYD
RYVAICSPLLYNVIMSSWVCSPLVLAAFFLGFLSALAHTSAMMKLSFCKSHIINHYFCDV
LPLLNLSCSNTYLNELLLFIIAGFNTLVPTLAVAISYAFIFYSILHIRSSEGRSKAFGTC
SSHLMAVGIFFGSITFMYFKPPSSNSLDQEKVSSVFYTTVIPMLNPLIYSLRNKDVKKAL
RKVLVGK
>h8D2
MATSNHSSGAEFILAGLTQRPELQLPLFLLFLGIYVVTVVGNLGMIFLIALSSQLYPPVY
YFLSHLSFIDLCYSSVITPKMLVNFVPEENIISFLECITQLYFFLIFVIAEGYLLTAMEY
DRYVAICRPLLYNIVMSHRVCSIMMAVVYSLGFLWATVHTTRMSVLSFCRSHTVSHYFCD
ILPLLTLSCSSTHINEILLFIIGGVNTLATTLAVLISYAFIFSSILGIHSTEGQSKAFGT
CSSHLLAVGIFFGSITFMYFKPPSSTTMEKEKVSSVFYITIIPMLNPLIYSLRNKDVKNA
LKKMTRGRQSS
>h8D4
MGVKNHSTVTEFLLSGLTEQAELQLPLFCLFLGIYTVTVVGNLSMISIIRLNRQLHTPMY
YFLSSLSFLDFCYSSVITPKMLSGFLCRDRSISYSGCMIQLFFFCVCVISECYMLAAMAY
DRYVAICSPLLYKVIMSPRVCSLLVAAVFSVGFTDAVIHGGCILRLSFCGSNIIKHYFCD
IVPLIKLSCSSTYIDELLILVIGGFNMVATSLTIIISYAFILTSILRIHSKKGRCKAFST
CSSHLTAVLMFYGSLMSMYLKPASSSSLTQEKVSSVFYTTVIPMLNPLIYSLRNNEVKNA
LMKLLRRKISLSPG
>m171-22
MGTGNHSVTVVFVLVGLTQQPELLLPLFILFLGIYVVTAVGNLGMILLITVSPLLHTPMY
YFLSSLSCVDLCYSTVITPKMLVNFLGKKNLIVYSECMAQLFFFVIFVVAEGYLLTAMAY
DRYVAICRPLLYNVIMSSRLCSLLVLVAFILGFVSALAHTSAMMNLSFCKSHVISHYFCD
VLPLLNLSCSDIKLNELLLFIIAGFNTLVPTLAVAISYVFIFCSILHIKSSKGRSKAFGT
CSSHLMAVGIFFGSITFMYFKPPSSNSLEQEKVSSVFYTTVIPMLNPLIYSLRNKDVKKA
LGKCLAGR
>m171-9
MGTGNHSAAVVFVLVELTQQPELLLPLFILFLGIYVVTAVGNLGMILLITVSPLLHTPMY
YFLSSLSCVDLCYSTVITPKMLVNFLGKKNVIVYSECMAQLFFFVIFVVAEGYLLTAMAY
DRYVAICRPLLYNVIMSSRLCSLLVLVAFILGFVSALAHTSAMMNLSFCKSHIISHYFCD
VLPLLNLSCSNTHLNELLLFIIGGFNTLVPTLAVAISYVFIFCSILHIKSSKGRSKSFGT
CSSHLMAVGIFFGSITFMYFKPSSSNSLEQEKVSSVFYTTVIPMLNPLIYSLRNKDVKKA
LGRFFVGR
>h2B11

MKSDNHSFLGDSPKAFILLGVSDRPWLELPLFVVLLLSYVLAMLGNVAIILASRVDPQLH
SPMYIFLSHLSFLDLCYTTTTVPQMLVNMGSSQKTISYGGCTVQYAVFHWLGCTECIVLA
AMALDRYVAICKPLHYAVLMHRALCQQLVALAWLSGFGNSFVQVVLTVQLPFCGRQVLNN
FFCEVPAVIKLSCADTAVNDTILAVLVAFFVLVPLALILLSYGFIARAVLRIQSSKGRHK
AFGTCSSHLMIVSLFYLPAIYMYLQPPSSYSQEQGKFISLFYSIITPTLNPFTYTLRNKD
MKGALRRLLARIWRLCG
>c2B11
MKSDNHSFLGDPPKAFILLGVSDRPWLELPLFVVLLLSYVLAMLGNVAIILASRVDPQLH
SPMYIFLSHLSFLDLCYTTTTVPQMLVNMGSSQKTISYGGCTVQYAVFHWLGCTECIVLA
AMALDRYVAICKPLHYAILMHRALCQQLVALAWLSGFGNSFVQVVLTVQLPFCGRQVLNN
FFCEVPAVIKLSCADTAVNDTILAVLVAFFVLVPLAVILLSYGFIARAVLRIQSSKGRHK
AFGTCSSHLMIVSLFYLPAIYMYLQPPSSYSQEQGKFISLFYSIITPTLNPFIYTLRNKD
MKGALRRLLARIWRLCG
>m2B11
MKSDNQSFSGDPPKAFILLGVSDRPWLELPLFVVLLLSYMLAMLGNVAIILASQLDPQLH
SPMYTFLSHLSFLDLCYTTTTVPQMLVNMGSSQKTISYGGCTVQYAVFHWLGCTECILLA
AMALDRYVAICEPLHYAVLMHRALCQQLVALAWLSGFGNSFVQVVLTVQLPFCGRQVLNN
FFCEVPAMIKLSCADTAVNDATLAVLVAFFVLVPLALILLSYGFIARAVLRIQSSKGRHK
AFGTCSSHLMVVSLFYLPAIYMYLQPPSSYSQEQGKFISLFYSIITPTLNPFIYTLRNKD
VKGALRRLLARIWRLCGR
>h2B2
MNWVNKSVPQEFILLVFSDQPWLEIPPFVMFLFSYILTIFGNLTIILVSHVDFKLHTPMY
FFLSNLSLLDLCYTTSTVPQMLVNICNTRKVISYGGCVAQLFIFLALGSTECLLLAVMCF
DRFVAICRPLHYSIIMHQRLCFQLAAASWISGFSNSVLQSTWTLKMPLCGHKEVDHFFCE
VPALLKLSCVDTTANEAELFFISVLFLLIPVTLILISYAFIVQAVLRIQSAEGQRKAFGT
CGSHLIVVSLFYGTAISMYLQPPSPSSKDRGKMVSLFCGIIAPMLNPLIYTLRNKEVKEA
FKRLVAKSLLNQEIRNMQMISFAKDTVLTYLTNFSASCPIFVITIENYCNLPQRKFP
>h2B3
MNWENESSPKEFILLGFSDRAWLQMPLFVVLLISYTITIFGNVSIMMVCILDPKLHTPMY
FFLTNLSILDLCYTTTTVPHMLVNIGCNKKTISYAGCVAHLIIFLALGATECLLLAVMSF
DRYVAVCRPLHYVVIMNYWFCLRMAAFSWLIGFGNSVLQSSLTLNMPRCGHQEVDHFFCE
VPALLKLSCADTKPIEAELFFFSVLILLIPVTLILISYGFIAQAVLKIRSAEGRQKAFGT
CGSHMIVVSLFYGTAIYMYLQPPSSTSKDWGKMVSLFYGIITSMLNSLIYSLRNKDMKEA
FKRLMPRIFFCKK
>h2B6
MNWVNDSIIQEFILLGFSDRPWLEFPLLVVFLISYTVTIFGNLTIILVSRLDTKLHTPMY
FFLTNLSLLDLCYTTCTVPQMLVNLCSIRKVISYRGCVAQLFIFLALGATEYLLLAVMSF
DRFVAICRPLHYSVIMHQRLCLQLAAASWVTGFSNSVWLSTLTLQLPLCDPYVIDHFLCE
VPALLKLSCVETTANEAELFLVSELFHLIPLTLILISYAFIVRAVLRIQSAEGRQKAFGT
CGSHLIVVSLFYSTAVSVYLQPPSPSSKDQGKMVSLFYGIIAPMLNPLIYTLRNKEVKEG
FKRLVARVFLIKK
>h2B8
MDQKNGSSFTGFILLGFSDRPQLELVLFVVLLIFYIFTLLGNKTIIVLSHLDPHLHNPMY
FFFSNLSFLDLCYTTGIVPQLLVNLRGADKSISYGGCVVQLYISLGLGSTECVLLGVMAF
DRYAAVCRPLHYTVVMHPCLYVLMASTSWVIGFANSLLQTVLILLLTLCGRNKLEHFLCE
VPPLLKLACVDTTMNESELFFVSVIILLVPVALIIFSYSQIVRAVVRIKSATGQRKVFGT
CGSHLTVVSLFYGTAIYAYLQPGNNYSQDQGKFISLFYTIITPMINPLIYTLRNKDVKGA
LKKVLWKNY
>h56A4
MASPSNDSTAPVSEFLLICFPNFQSWQHWLSLPLSLLFLLAMGANTTLLITIQLEASLHQ
PLYYLLSLLSLLDIVLCLTVIPKVLAIFWFDLRSISFPACFLQMFIMNSFLTMESCTFMV
MAYDRYVAICHPLRYPSIITDQFVARAVVFVIARNAFVSLPVPMLSARLRYCAGNIIKNC
ICSNLSVSKLSCDDITFNQLYQFVAGWTLLGSDLILIVISYSFILKVVLRIKAEGAVAKA
LSTCGSHFILILFFSTVLLVLVITNLARKRIPPDVPILLNILHHLIPPALNPIVYGVRTK
EIKQGIQNLLKRL
>c56A4
MASPSNDSTVPVSEFLLICFPNFQSWQHWLSLPLSLLFLLAMGANTTLLITIQLDASLHQ
PLYYLLRLLSLLDIVLCLTVIPKVLAIFWFDLRSISFPACFLQMFIMNSFLTMESCTFMV
MAYDRYVAICHPLRYPSIITDQFVARAVVFVIARNAFVSLPVPMLSARLRYCAGNIIKNC
ICTNLSVSKLSCDDITFNQLYQFVAGWTLLGSDLILIVISYSFILKVVLRIKAEGAVAKA
LSTCGSHFILILFFSTVLLVLVITNLARKRIPPDVPILLNILHHLIPPALNPIVYGVRTK
EIKQGIQNLLKRL

>m56A4
MASPSNDSAAPVSEFLLICFPDFQSWQHWLSLPLSLLFLLAMGANTTLLITIQLESSLHQ
PLYYLLSLFSLLDIVLCLTVIPKVLAIFWFDLRSISFPACFLQMFIINSFLTMESCTFMV
MAYDHYVAICHPLRYPSIITDQFVARAVVFVIARNAFVSLPVPMLSARLRYSVGNIIKNC
ICTNLSVSKLSCDDITFNQLYQFVAGWTLLGSDLILIIISYSFILKVVLRIKAEGAVAKA
LSTCGSHFILIFFFSTVLLVLVITNLARKTIPLDVPILLNILHHLIPPALKPHCLWYENQ
GDQAGNPEPAEEVIRVKRIRSTFRVVN
>h56A5
MTLPSNNSTSPVFEFFLICFPSFQSWQHWLSLPLSLLFLLAMGANATLLITIYLEASLHQ
PLYYLLSLLSLLDIVLCLTVIPKVLAIFWFDLRSISFPACFLQMFIMNSFLTMESCTFMI
MAYDRYVAICKPLQYSSIITDQFVARAAIFVVARNGLLTMPIPILSSRLRYCAGHIIKNC
ICTNVSVSKLSCDDITLNQSYQFVIGWTLLGSDLILIVLSYFFILKTVLRIKGEGDMAKA
LGTCGSHFILILFFTTVLLVLVITNLARKRIPPDVPILLNILHHLIPPALNPIVYGVRTK
EIKQGIQNLLRRL
>h56A1
MIQPMASPSNSSTVPVSEFLLICFPNFQSWQHWLSLPLSLLFLLAMGANTTLLITIQLEA
SLHQPLYYLLSLLSLLDIVLCLTVIPKVLAIFWYDLRSISFPACFLQMFIMNSFLPMESC
TFMVMAYDRYVAICHPLRYPSIITNQFVAKASVFIVVRNALLTAPIPILTSLLHYCGENV
IENCICANLSVSRLSCDNFTLNRIYQFVAGWTLLGSDLFLIFLSYTFILRAVLRFKAEGA
AVKALSTCGSHFILILFFSTILLVVVLTNVARKKVPMDILILLNVLHHLIPPALNPIVYG
VRTKEIKQGIQKLLQRGR
>h56A3
MTTHRNDTLSTEASDFLLNCFVRSPSWQHWLSLPLSLLFLLAVGANTTLLTTIWLEASLH
QPLYYLLSLLSLLDIVLCLTVIPKVLTIFWFDLRPISFPACFLQMYIMNCFLAMESCTFM
VMAYDRYVAICHPLRYPSIITDHFVVKAAMFILTRNVLMTLPIPILSAQLRYCGRNVIEN
CICANMSVSRLSCDDVTINHLYQFAGGWTLLGSDLILIFLSYTFILRAVLRLKAEGAVAK
ALSTCGSHFMLILFFSTILLVFVLTHVAKKKVSPDVPVLLNVLHHVIPAALNPIIYGVRT
QEIKQGMQRLLKKGC
>h5P3
MGTGNDTTVVEFTLLGLSEDTTVCAILFLVFLGIYVVTLMGNISIIVLIRRSHHLHTPMY
IFLCHLAFVDIGYSSSVTPVMLMSFLRKETSLPVAGCVAQLCSVVTFGTAECFLLAAMAY
DRYVAICSPLLYSTCMSPGVCIILVGMSYLGGCVNAWTFIGCLLRLSFCGPNKVNHFFCD
YSPLLKLACSHDFTFEIIPAISSGSIIVATVCVIAISYIYILITILKMHSTKGRHKAFST
CTSHLTAVTLFYGTITFIYVMPKSSYSTDQNKVVSVFYTVVIPMLNPLIYSLRNKEIKGA
LKRELRIKIFS
>c5P3
MGTGNDTTVVEFTLLGLSEDTTVCAILFLVFLGIYVVTLMGNISIIVLIRRSHHLHTPMY
IFLCHLAFVDIGYSSSVTPVMLMSFLRKETSLPVAGCVAQLCSVVTFGTAECFLLAAMAY
DRYVAICSPLLYSTCMSPGVCIILVGMSYLGGCVNAWTFIGCLLRLSFCGPNKVNHFFCD
YSPLLKLACSHDFTFEIIPAISSGSIIVATVCVIAISYIYILITILKMHSTKGRHKAFST
CTSHLTAVTLFYGTITFIYVMPKSSYSTDQNKVVSVFYTVVIPMLNPLIYSLRNKEIKGA
LKRELRIKIFS
>h5P2
MNSLKDGNHTALTGFILLGLTDDPILRVILFMIILSGNLSIIILIRISSQLHHPMYFFLS
HLAFADMAYSSSVTPNMLVNFLVERNTVSYLGCAIQLGSAAFFATVECVLLAAMAYDRFV
AICSPLLYSTKMSTQVSVQLLLVVYIAGFLIAVSYTTSFYFLLFCGPNQVNHFFCDFAPL
LELSCSDISVSTVVLSFSSGSIIVVTVCVIAVCYIYILITILKMRSTEGHHKAFSTCTSH
LTVVTLFYGTITFIYVMPNFSYSTDQNKVVSVLYTVVIPMLNPLIYSLRNKEIKGALKRE
LVRKILSHDACYFSRTSNNDIT
>m204-6
MEPGNYTVVTEVILLGFTEDAIIRAILFIVFLIIYSVTLMGNASIIMLIRRSPQLHTPMY
LLLSHLAFVDIGYSSSVTPIMLKGFLRKETFILVSGCVAQLCSVVTFGSTECFLLAAMAY
DRYVAICSPLLYATQMSSTVCILLVGASYLGGCVNAWTFTGCLLNLSFCRPNKVNHFFCD
YSPLLKISCSHDFSSEVIPAISSGSIIVVTVFIIALSYVYILVSILKMRSTEGRQKAFST
CTSHLTAVTLFYGTITFIYVMPKSSYSTDQNKVVSVFYTVVIPMLNPIIYSLRNKDVKEA
MKKLMANTHH
>h2J2
MMIKKNASSEDFFILLGFSNWPQLEVVLFVVILIFYLMTLTGNLFIIILSYVDSHLHTPM
YFFLSNLSFLDLCYTTSSIPQLLVNLRGPEKTISYAGCMVQLYFVLALGITECVLLVVMS
YDRYVAVCRPLHYTVLMHPRFCHLLVAASWVIGFTISALHSSFTFWVPLCGHRLVDHFFC
EVPALLRLSCVDTHANELTLMVMSSIFVLIPLILILTTYGAIARAVLSMQSTTGLQKVFR
TCGAHLMVVSLFFIPVMCMYLQPPSENSPDQGKFIALFYTVVTPSLNPLIYTLRNKHVKG

AAKRLLGWEWGK
>c2J2
MIKKNASSEDFFILLGFSNWPQLEVVLFVVILIFYLMTLTGNLFIIILSYVDSHLHTPMY
FFLSNLSFLDLCYTTSSIPQLLVNLRGPEKTISYAGCMVQLYFVLALGIAECVLLVVMSY
DRYAAVCRPLHYTVLMHPRFCHLLAAASWVIGFTISALHSSFTFWVPLCGHRLVDHFFCE
VPALLRLSCVDTHANELTLMVMSSIFVLIPLILILTSYGAIARAVLSMQSTTGLQKVFRT
CGAHLMVVSLFFIPVMCMYLQPPSENSDQGKFIALFYTVVTPSLNPLIYTLRNKDVKGAA
KRLLGWEWGM
>h2J1
MKKNASFEDFFLLLGFSNWPHLEVVLFVVILIFYLITLIGNLFIIILSYLDSHLHTPMYF
FLSNLSFLDLCYTTSSIPQLLVNLWGPEKTISYAGCTVQLYFVLALGTAECVLLVVMSYD
RYAAVCRPLHYTVLMHPRFCRLLAAASWVSGFTTSALHSSFTFWIPLCRHRLVDHFFCEV
PALLRLSCVDTQANELTLMVMSSIFVLIPLILILTSYGAIARAVLSMQSTTGLQKVLRTC
GAHLMVVSLFFIPVMCMYLQPPSENSQDQGKFIALFYTVVTPSLNPLIYTFRNKDVRGAV
KRLMGWEWGM
>m256-18
MVENFNASWEGYFIFLGFSKWPHLEVVLFVVILIFYMMTLMGNLFIIILSHLDSHLHTPM
YFFLSNLSALDLCYTTSSVPQLLFNLWGPKKTISYAGCMLQLYFVLALGTTECVLLVVMS
YDRYVAVCKPLHYSVLMNPRFCQLLAAASWVCGFTTSALHSSFTFWVPLCGHRKVDHFFC
EVPALLQLSCVDIHANEMTLMVMSAIFVVIPLILILSSYAAIAWTVLEMQSTTRLQKVFG
TCGAHLTVVSLFFIPIMCIYLQPSTKSSQDHAKFIALFYTVVTPSLNPLIYTLRNKDVRG
AIRRLSRYEREK
>h2J3
MNDDGKVNASSEGYFILVGFSNWPHLEVVIFVVVLIFYLMTLIGNLFIIILSYLDSHLHT
PMYFFLSNLSFLDLCYTTSSIPQLLVNLWGPEKTISYAGCMIQLYFVLALGTTECVLLVV
MSYDRYAAVCRPLHYTVLMHPRFCHLLAVASWVSGFTNSALHSSFTFWVPLCGHRQVDHF
FCEVPALLRLSCVDTHVNELTLMITSSIFVLIPLILILTSYGAIVRAVLRMQSTTGLQKV
FGTCGAHLMAVSLFFIPAMCIYLQPPSGNSQDQGKFIALFYTVVTPSLNPLIYTLRNKVV
RGAVKRLMGWE
>c2J3
MNDDGKVNASSEGYFVLVGFSNWPHLEVVIFVVVLIFYLMTLIGNLFIIILSYLDSHLHT
PMYFFLSNLSFLDLCYTTSSIPQLLVNLWGPEKTISYAGCMIQLYFVLALGTTECVLLVV
MSYDRYAAVCRPLHYTVLMHPRFCHLLAVASWVSGFTNSALHSSFTFWVPLCGHRQVDHF
FCEVPALLRLSCVDTHANELTLMITSSIFVLIPLILILTSYGAIVRAVLRMQSTTGLQKV
FGTCGAHLMAVSLFFIPAMCIYLQPPSGNSQDQGKFIALFYTVVTPSLNPLIYTLRNKDV
RGEVKRLMGWE
>m2J3
MNDDGKVNASSEGYFILVGFSNWPHLEVVLFVVVLIFYLMTLIGNLFIIILSYLDSHLHT
PMYFFLLNLSFLDLCYTTSSIPQLLVNLWGPEKTISYAGCMIQLYFVLALGTTECVLLVV
MSYDRYAAVCRPLHYTVLMHPRFCHLLAVASWVSGFTNSALHSSFTFWIPLCGHHQVDHF
FCEVPALLRLSCVDTHVNELTLMITSSVFVLIPLILILTSYGAIVQAVLRMQSTTGLQRV
FGTCGAHLMVVSLFFIPAMCIYLQPPSENSQDQGKFIALFYTVVTPSLNPLIYTLRNKDV
RGAVKRLMGWE
>h1C1
MEKRNLTVVREFVLLGLPSSAEQQHLLSVLFLCMYLATTLGNMLIIATIGFDSHLHSPMY
FFLSNLAFVDICFTSTTVPQMVVNILTGTKTISFAGCLTQLFFFVSFVNMDSLLLCVMAY
DRYVAICHPLHYTARMNLCLCVQLVAGLWLVTYLHALLHTVLIAQLSFCASNIIHHFFCD
LNPLLQLSCSDVSFNVMIIFAVGGLLALTPLVCILVSYGLIFSTVLKITSTQGKQRAVST
CSCHLSVVVLFYGTAIAVYFSPSSPHMPESDTLSTIMYSMVAPMLNPFIYTLRNRDMKRG
LQKMLLKCTVFQQQ
>m1C1
MEKRNLTVVREFVLLGLPSSAEQQHLLSVLFLCMYLATTLGNMLIIVTTGFDSRLHSPMY
FFLSNLAFVDICFTSTTVPQMVVNILTDTKTISFAGCLSQLFFFVTFVNMDSLLLCVMAY
DRYVAICHPLHYTATMNLCLCVQLMAGLWLVTYLHALLHTVLTARLSFCASNIIHHFFCD
LNPLLQLSCSDVSFNVMIIFAVGGLLAITPLVCILISYGIIFSTVLKITSTQGKQRAVST
CSCHLSVVVVFYGTAIAVYFSPSSSHTPESDTVSTVMYSVVAPMLNPFIYTIRNRDMKRG
LQKMLFKCTVFQRNNDLSD
>h11A1
MEIVSTGNETITEFVLLGFYDIPELHFLFFIVFTAVYVFIIIGNMLIIVAVVSSQRLHKP
MYIFLANLSFLDILYTSAVMPKMLEGFLQEATISVAGCLLQFFIFGSLATAECLLLAVMA
YDRYLAICYPLHYPLLMGPRRYMGLVVTTWLSGFVVDGLVVALVAQLRFCGPNHIDQFYC
DFMLFVGLACSDPRVAQVTTLILSVFCLTIPFGLILTSYARIVVAVLRVPAGASRRRAFS

```
TCSSHLAVVTTFYGTLMIFYVAPSAVHSQLLSKVFSLLYTVVTPLFNPVIYTMRNKEVHQ
ALRKILCIKQTETLD
>c11A1
MEIVSIGNKTITEFVLLGFYDIPELHFLFFIVFTAVYVFIIIGNMLIIVAVVSSQRLHKP
MYIFLANLSFLDILYTSAVMPKMLEGFLQEATISVACCLLQFFIFGSLATAECLLLAVMA
YDRYLAICYPLYYPLLMGPSRCMGLVVTTWLSGFMVDGLVVALVAQLRFCGPNHIDQFYC
DFMLFVGLACSDPRVAQVTTLVLSVFCLTIPFGLILTSYARIVVAVLRVPAGASRRRAFS
TCSSHLAVVTTFYGTLMILYVAPSAVHSQLLSKVFSLLYTVVTPLFNPVIYTLRNKEVHQ
VLRKILYIKQTETLD
>h51L1
MGDWNNSDAVEPIFILRGFPGLEYVHSWLSILFCLAYLVAFMGNVTILSVIWIESSLHQP
MYYFISILAVNDLGMSLSTLPTMLAVLWLDAPEIQASACYAQLFFIHTFTFLESSVLLAM
AFDRFVAICHPLHYPTILTNSVIGKIGLACLLRSLGVVLPTPLLLRHYHYCHGNALSHAF
CLHQDVLRLSCTDARTNSIYGLCVVIATLGVDSIFILLSYVLILNTVLDIASREEQLKAL
NTCVSHICVVLIFFVPVIGVSMVHRFGKHLSPIVHILMADIYLLLPPVLNPIVYSVRTKQ
IRLGILHKFVLRRRF
>c51L1
MGDWNNSDAVEPIFILRGFPGLEYVHSWLSILFCLVYLVAFMGNVTILSVIWIESSLHQP
MYYFISILAVNDLGMSLSTLPTMLAVLWLDAPEIQASACYAQLFFIHTFTFLESSVLLAM
AFDRFVAICHPLHYPTILTNSVIGKIGLACLLRSLGVVLPTPLLLRHYHYCHGNALSHAF
CLHQDVLRLSCTDARINSIYGLCVVIATLGVDSIFILLSYVLILNTVLDIASCEEQLKAL
NTCVSHICVVLIFFVPVIGVSMVHRFGKHLSPIVHILMADIYLLLPPVLNPIVYSVRTKQ
IRLGILRKFVLRRRF
>m129-1
MDGDNETMVAEFLLLGLSGKSEQEEVVFGMFLGMYLVTISGNLLIILAISCDPHLHTPMY
FFLANLSSVDICFSSVTVPKALVNHVLGSKSISYTECMIQIYFFITFINMDGFLLSVMAY
DRYVAICHPLHYTMMMRSRLCVLLVAISWVITNLHALLHTLLMVRLTFCSHNAVHHFFCD
PYPILKLSCSDTFINDLMVFTVGGVIFLTPFSCIVVSYVYIFSKVLKIPSARGIRKALST
CGSHLTVVSLFYGAILGVYMRPSSSYSLQDTVATVIFTVVTPLVNPFIYSLRNQDMKGAL
RKIMLRS
>r129-1
MDRDNETMVAGFLLLGLSGKSEQEEVVFGMFLGMYMVTISGNLLIILAISCDPRLHTPMY
FFLANLSSVDICFSSVTIPKALMNHVLGSKSISYTECMIQIYFFITFINMDGFLLSVMAY
DRYVAICHPLHYTMMMRPRLCVLLVAISWVITNQHALLHTLLMVRLTLCSHNAVHHFFCD
PYPILKLSCSDTFINDLMVFTVGGVIFLTPFSCIVVSYVYIFSKVLKIPSARGIRKALST
CGSHLTVVSLFYGAILGVYMRPSSTYSLQDTVATVIFTVVTPLVNPFIYSLRNQDMKGAL
RKIILRS
>m161-1
MTAKNSSVTEFILAGLTDQPGLRMPLFFLFLGFYMVTVVGNLGLISLIGLNSHLHTPMYF
FLFNLSLIDFCYSSTISPKMLMSFISKKNIISHPGCMAQLFFFCFFVISESFILSAMAYD
RYVAICNPLMYMVTMSPQVCLLLLFGVYLMGFVGAMAHTISMARLTFCADNIVNHYMCDI
LPLLEHSCTSTYVNELVVFIFVSFDIGVPIVTIFISYALILSSILHMHSTEGRSKAFSTC
SSHMIVVCLFFGSGAFMYLQPPSVLSLDQGKVSSLFYTIVVPMLNPLIYSLRNKDVKVAV
RKTLDRRIFS
>r161-1
MTAKNSSVTEFILAGLTNQPGLCMPLFFLFLGFYMVTVVGNLGLISLIGLNSHLHTPMYF
FLFNLSLIDFCYSSTISPKMLMSFILKQNIISHSGCMTQLFFFCFFVISESFILSAMAYD
RYVAICNPLMYMVTMSPQVCLFLLFGVYLMGFVGAMAHTISMARLTFCADNLVNHYMCDI
LPLLEHSCTSTYVNELVVFIFVSFDIGVPIVTIFISYALILSSILRMHSTEGMSKAFSTC
SSHMIAVCLFFGSGAFMYLQPPSVLSLDQGKVSSLFYTIVVPMLNPLIYSLRNKDVKVAL
RKTTLGRRIFS
>m189-1
MQMESQNLTVVTEFILRGITDRPELQVPLFGLFFMIYLISLFGNLGMIILTIVESRLQTP
MYFFLRHLAITDLGYSTAIGPKMLANFVVSKNTISFHLCATQLAFFLLFIACELFILSVM
SYDRYVAICNPLLYNVIMSQTVCWVLVAIPYLYSVFISLIVTINIFSSSFCGYNVIPHFY
CDGLPLISLLCTNTDKIGLIILILSAINLISSLLIILGSYLLIFRAILRMNSAEGRRKAF
STCGSHLTVVSVFYGTLIFMYVQPKTSHSFDTDKVASIFYTLVIPMLNPLIYSLRNKDVK
YALRKTGKIIQNNFS
>r189-1
MESQNLTPVTEFILRGITDRPELQAPLFGLFLIIYLISLVGNLGMIILTIAESRLQTPMY
FFLRHLAITDLGYSTAIGPKMLANFVVSKNTISFHLCATQLAFFLLFIACELFILSVMSY
DRYVAICNPLLYNVIMSQIMCWVLVAIPYLYSVFISLIVTINIFSSSFCGYNVIPHFYCD
```

GLPLISLLCTNTDKIELIILILSAINLISSLLVILGSYLLIFRAILRMNSAEGRRKAFST
CGSHLTVVIVFYGTLIFMYVQPKTSHSFDNDKVASIFYTLIIPMLNPLIYSLRNKDVKYA
IRKTGKIICSNFS
>m207-1
MTENNFTKVTVFMFSGFSDHPELQVSLFLIFLFIYLFTVWGNIGLILLIRIDSQLHTPMY
FFLSNLAFIDIFYSSTVTPKALVDFQSTQKSISFVGCFVQMYFFVGLVCSECFLLGSMAY
DRYVAICNPLLYSVIMSQKVCNWLAVIPYMIGFTNSLISICVISSLPLCDPYINHFFCDT
TALLALSCVDAFSTELVIFVLAGFTLLSSLLIITFTYVTIISAILRIQSAAGRWKAFSTC
ASHLTGVTVFYGSLIFTYLQPDNTSSLTQAQVASVFYTIVIPMLNPLIYSLRNKDVKNAL
LRVIHRKHLL
>r207-1
MTENNFTKVTVFMLSGFSDHPELQVSLFLIFLFIYLFTVWGNIGLIMLIRIDSQLHTPMY
FFLSNLAFIDIFYSSTVTPKALVDFQSTQKSISFVGCFVQMYFFVGLVCSECFLLGSMAY
DRYVAICNPLLYSVIMSQKVCNWLAVIPYMIGFTNSLISICVISSLPLCDPYINHFFCDT
TALLALSCVDAFNTELVIFVLAGFTLLSSLLIITFTYLTIISAILKIQSAAGRWKAFSTC
ASHLTGVTVFYGSLIFTYLQPDNTSSLTQAQVASVFYTIVIPMLNPLIYSLRNKDVKNAL
LRVIDRKLFL
>m180-1
MDKENHSVVTEFVFMGITQDPQLQIIFFVVFLLVYLVNVIGNVGMIILIITDSQLHTPMY
FFLCNLSFVDLGYSSAIAPRMLADFLTKHKVISFSSCATQFAFFVGFVDAECYVLAAMAY
DRFVAICRPLHYSTLMSKKVCLVLMLGSYFAGLVSLVAHTSLTFSLSYCGSNIINHFFCE
IPPLLALSCSDTYISEILLFSLCGFIEFSTILIIFISYAFILIAIIRIRSAEGRLKAFST
CGSHLTGVTLFYGTVMFMYLRPTSSYSLDQDKWASVFYTIIIPMLNPLIYSLRNKDVKAA
FKKLIGKKPQ
>r180-1
MHKENHSVVTEFIFMGITQDPQLQIIFFVVFLIVYLVNVIGNVGMIILIITDSQLHTPMY
FFLCNLSFVDLGYSSAIAPRMLADCLTKHKVISFSSCATQFAFFVGFVDAECYVLAAMAY
DRFVAICRPLHYSTLMSKKVCLVLMLGSYLAGLVSLVAHTSLTFSLSYCASNVINHFFCE
IPPLLALSCSDTYISEILLFSLCGFIEFSTILIIFISYAFILIAIIRMRSAEGRLKAFST
CGSHLTGVTLFYGTVMFMYLRPTSSYSLDQDKWASVFYTIIIPMLNPLIYSLRNKDVKAA
FKKLIGKKPQ
>m260-1
MMQANQTQVTEFILLGLSDDPHTQKLLFILFLGIYMVTVLGNLLLMFLVRADSRLHTPMY
FFLCNLSLADLCFSTNIVPQALIHLLSRKKTISFRRCAAQLLLFLIFGCTQCALLAVMSY
DRYVAICNPLHYSSIMTWRVCIQLATVSWTSGILVSVVDTTFTLRLPYRGSNSIAHFFCE
APALLALASTDTQTSEMAIFLMGVVILLIPVSLILVSYGHIIVTVVKMKSAAGRFKAFST
CGSHLMVVILFYGSAIITYMTPKSSKEQEKLVSVFYAMVTPMLNPLIYSLRNKDVKGALW
KVAMKNFSSRLRITH
>r260-1
MRQANQTQVTEFLLLGLSDDPHTQTLLFILFLGIYLVTVLGNLLLMFLVWADSRLHTPMY
FFLCNLSLADLCFSTNIVPQALTHLLSRKKSISFRRCAAQLLLFLIFGCTQCALLAVMSY
DRYVAICNPLHYSSIMTWRLCIQLATVSWTSGILVSVVDTTFTLRLPYRGSNSIPHFFCE
APALLTLASTDTQTSEMVIFLMGVVILLIPVSLILVSYGHIIVTVVKMKSAAGRFKAFST
CGSHLIVVILFYGSGIITYMTPKSSKEQEKLVSVFYAMVTPMLNPLIYSLRNKDVKGALW
KVALKNFSSRPIIIQ
>m256-17
MEVDSNSSSGSFILMGVSDHPHLEIIFFAVILASYLLTLVGNLTIILLSRLDARLHTPMY
FFLSNLSSLDLAFTTSSVPQMLKNLWGPDKTISYGGCVTQLYVFLWLGATECILLVVMAF
DRYVAVCRPLHYMTVMNPRLCWGLAAISWLGGLGNSVIQSTFTLQLPFCGHRKVDNFLCE
VPAMIKLACGDTSLNEAVLNGVCTFFTVVPVSVILVSYCFIAQAVMKIRSVEGRRKAFNT
CVSHLVVVFLFYGSAIYGYLLPAKSSNQSQGKFISLFYSVVTPMVNPLIYTLRNKEVKGA
LGRLLGKGRGAS
>r256-17
MEVGSNISSGSFILMGISNHPQLEIIFFVVILSSYLLTLVGNLTIILLSRLDARLHTPMY
FFLSNLSSLDLAFTTSSVPQMLKNLWGPDKTISYGGCVTQLYVFLWLGATECILLVVMAF
DRYVAVCRPLHYMTVMNPRLCWVLAAISWLGGLGNSVIQSTFTLQLPFCGHRKVDNFLCE
VPAMIKLACGDTSLNEAVLNGVCTFFTAVPLSIILVSYCFIAQAVMKIRSVEGRRKAFNT
CVSHLVVVFLFYGSAIYGYLLPAKSSNQDQGKFISLFYSVVTPMVNPLIYTLRNKEVKGA
LGRLLGKGRGAS
>m23-1
MGYTNLSYLNPGTVILIGIPGLEHVQFWIGFPFFVVCLVALLGNLFLLIIIPTERSLHQP
MYIFLAVLAATDLGLCLAIAPKMLAIFWFGSCSMAFDACLTQLFFIHALQGMESGVLLAM

AFDRYVAICDPLRHTAVLTPLFLLRVVLVVAIRATVLVGVLPILLKRLQWFHSVVIVHSY
CEHMAVVKLAAEDVRINKSYGLFVAFAILGFDMIFVFISYILIFRAVFRLPQKEARSKAF
NTCTAHIVVFLEFYILAFFSFFSHRFGHVSPYVHILLSTIYLLLPPALNPIVYGVKTKEI
RKWVVQIFVLKSNTQ
>r23-1
MGYTNLSYLNPRTVILVGIPGLEHVQFWIGFPFFVVCLVALLGNLFLLIIVPRERSLHQP
MYIFLAVLAATDLGLCLAIAPKMLAIFWFGSCSMAFDTCLTQLFFIHALQGMESGILLAM
AFDRYVAICDPLRHPSILTPLFLLRVILMVAVRATVLVGILPVLLKRLQWFHSVVIVHSY
GEHMAVVKLAAEDVTINKSYGLFVAFAILGFDMIFVFISYFLIFQAVLHLPQKEARSKAF
NTCTAHIVVFLEFYILAFFSFFSHRFGHVSPYIHILLSTIYLLLPPALNPIVYGVKTKEI
RKWVVHIFVLKSNTQ
>m268-1
MGQNDSSVVEFILLGFSHFPELQVHMFGAFLVIYLVTLTGNATIVTVIFLDHSLHIPMYL
FLQNLSVVEASFSTTVMPEMLVVLTSEKATISFGGCFAQTYFILLFGGTECFLLGAMAYD
RFAAICYPLTYPMIMSKRIFVKLVVCSWVLGIMTATVSVTWVFSFPFCGPSKINHISCEV
PAVLELACADTFLFEVYSFTGTILLVLVPFLLILLSYTQILFTVLRMPSTTGRQKAFSTC
ASHLTSVTLFYSTACMTYLQPKSKYSPDTKKLMSLAYSLLTPLLNPLIYSLRNKEMKRAV
VKLCQIKVVF
>r268-1
MTGQNDSSVVEFILLGFSHLPELQVHMFGAFLVIYLVTLAGNATIVTVILLDHSLHIPMY
LFLQNLSVVEASFSTAIMPEMLVVLTTEKATISFGGCFAQMYFILLFGGTECFLLGAMAY
DRFAAICHPLTYPVIMSKRIFVKLAMSSWVLGIMIATMQATWVFSFPFCGPSNINHISCE
TPAVLELACADTFLFEVYAFTGTILIVMVPFLLILLSYTRILFTILRMPSTTGRQKAFST
CASHLTSVTLFYGTACMTYLQPKSRYSPDTKKLMSLAYSLLTPLLNPLIYSLRNKEMKRA
VVKLWQRKVVC
>m170-1
MPLNAQKTMENDSSVSEFILMGLTDQPELQLPLFVLFLVNYTVTVMGNLSLMNLICLNSN
LHTPMYFFIFNLSFIDFCYSMVFTPKMLMGFVVEKNIISFRGCMTQLFFFLFFVNSESYV
LTAMAYDRYVAICQPLLYKAVMSPGICFLLIFCTYLMGLVSALFHTGFMIRLNFCDSNVI
NHYMCDIFPLFRLSCSSTYLTELVSSAVVGTAIILCCLIILISYGMILYNIIHMSSGKGW
SKALGTCGSHIITVSLFYVTGMLAYVKPSSAETVGQGKIFSVFYTFLVPMLNPLIYSLRN
KDVKLAVKKTWKRLTC
>r170-1
MHMTMENGSSVSEFILMGLTDQPELQLPLFVLFLVNYTATVMGNLCLMNLICLNSNLHTP
MYFFIFNLSFIDLCYSMVFTPKMLMSFVLEKNTISFRGCMTQLFFFLFFVNSESYVLTAM
AYDRYVAICKPLLYKVVMSPGICCLLIFGTYLMGFVSALFHTGFMIRLNFCDSNIINHYM
CDIFPLLRLSCSSTYVNELVSSAVVGTAIILCCLIILISYAMILFNIIHMSSGKGWSKAL
GTCGSHIITVSLFYVTGLLAYVKPSSAETVGQGKFFSVFYTFLVPMLNPLIYSLRNKDVK
LAVKKNLEETHILVYPLISFLASSNPV
>m162-1
MTPGMVSENNSSVKEFILLGLTQLPELQLPLFFLFLGIYVFSMVGNLGLIVLIVLNPPLH
TPMYYFLFNLSFTDLCYSSVITPKMLVGFVKQNIISHAECMTQLFFFAFFVIDECCILTA
MAYDRYAAICKPLLYKVIMSHQVCFVLMVGGYTVGFVGATAHTVCMLRLTFCDGNIINHY
MCDIPPLLKLSCTSTSINELVVFIVVGVSIIVPSLTVFISYTLILSNILRIHSAKGRSKA
LSTCSSHMIAVSLFFGSSSFIYFKSSPVGSVDKDKISTVFYTVVVPMMNPFIYSLRNKDV
QIALRKTLKKNCSLK
>r162-1
MTQGVVSENNSSVKEFILLGLTQQPELQLPLFFLFLGIYVVSMVGNLGLIVLIVLNPHLH
TPMYYFLFNLSFTDLCYSSVITPKMLVGFVKQNVISHAECMTQLFFFAFFVIDECCILTA
MAYDRYAAICKPLLYKVIMSHQVCFMLMVGGYTVGFVGAIAHIVSMLRLTFCDGNIINHY
MCDIPPLLKLSCTSTSINELVVFIVVGVSIIVPSVTVSVSYTLILSNILHIHSTKGRSKA
LSTCSSHMIAVSLFFGSSSFIYFKSSPVGSVDQDKISTVFYTVVVPMMNPFIYSLRNKDV
QIALSKTLKKKSSLE
>m184-1
MTEDNYSLTTEFILIGFSDHPDLKILLFLVFSTIYLVTMVGNLGLVALIYMEPRLHTPMY
IFLGNLALMDSCCSCAITPKMLENFFSVNKRISLYECMAQFYFLCLAETADCFLLAAMAY
DRYVAICNPLQYHTMMSKKLCLQMTTGAYIAGNLHSMIHIGFLFRLTFCRSHVIKHFFCD
VLPLYRLSCVDPYINELMILIFSGSVQTFSIIIVLISYFCIIFTIFTMKSREGRSKALST
CASHFLSVSIFYGSLLYTYIRPSSINEGNEDIPVAIFYTLVIPLLNPFIYSLRNKEVINA
IKRTMNKG
>r184-1
MTEDNYSLTTEFILVGFSDHPDLKTLLFLVFSTIYLVTMVGNLGLVALIYMEPRLHTPMY

IFLGNLALMDSCCSCAITPKMLENFFSVDRRISLYECMAQFYFLCLAETADCFLLAAMAY
DRYVAICNPLQYHTMMSKKLCVQMTTGAYIAGNLHSIIHIGFLFRLTFCRSNVIKHFFCD
VIPLYRLSCADPYINELMILIFSGSVQTFSIIIVLISYFCILYTIFTMKSTEGRSKALST
CASHFLSVSIFYGSLLYTYIRPNSLNEGEEDIPVAIFYTLVIPLLNPFIYSLRNKEVINV
IKKTVKKG
>m203-1
MKHSNDSKVTEFILLGFAGQNESWHILFVVFLVIYIATLVGNIGMILLIKLHSSLHTPMY
FFLQHLAFVDLCYSSAITPRTLQNFVSTKPSISFTGCLAQLLVYGIFVTSDCFILAAMAV
DRYVAICNPLRYPIIMSQRLCILLLLGSYTMGFLNATVNTGFTFLSNFCKSNVINHFFCD
VPPILALSCSSIDLNIMVLTIFVGFNLTFTVSVVILSYTFILAAILRMSSASGRRKAFST
CASHMTAVTIFYGTLSYMYVLHGTNRSQEQEKVASVFYGIMIPMLNPLIYSLRNQDVIEA
LRHIGNKCF
>r203-1
MGHSNDTKVTEFILLGFAGQHESWHILFVVFLIIYIATLVGNIGMILLIKLHSSLHTPMY
FFLQHLAFVDLCYSSAITPKTLQNFVSSKPSISFTGCIVQLLVYGIFVTSDCFILAAMAV
DRYVAICNPLRYPIIMSQTLCILLLLGSYTMGFLNATVNTGFTFLSNFCKSNTINHFFCD
VPPILALSCSSIDLNIMVLTIFVGFNLTFTVSVVILSYTFILAAILRMSSAAGRRKAFST
CASHMTAVTIFYGTLSYMYVLHGTNKSQEQEKVASVFYGIMIPMLNPLIYSLRNQDVIGA
LRNIGNKCF
>m272-1
MDSYNQTFTGFILLGLFPPSKIGLFLFILIVLIFLTAWIGNLSMILLILLDSHLHTPMYF
LLSQLSLIDLNYISTIVPKMVSDFMLGNKYISFIGCGFQIFLFLTFGGAETLLLASMAYD
RYVAICFPLHYATHMNKRVCVMMITGAWILGSINSCAHTGYALQIPYCRSRAINHFFCDV
PAMLTLACTDTWVYEYTVFVSTILFLVFPFIGIVCSYGRVFLAIYRMHSRAGKKKAYSTC
STHLTVVTFYYAPFAYTYLRPRSLRSPEEDKILAVFYTVLTPMLNPIIYSLRNKEVIGAL
RRMTHRICFAKI
>r272-1
MDSYNQTFTGFILMGLFPPSKIGLFLFILIVVVFLTAWIGNLSMILLIFLDSHLHTPMYF
LLSQLSLIDLNYISTIVPKMVSDFMLGNKYISFIGCGFQIFLFLTFGGAETLLLASMAYD
RYVAICFPLHYATHMNKRVCVMMITGAWVLGSINSCAHTGYALQIPYCRSRAINHFFCDV
PAMLTLACMDTWIYEYTVFVSTILFLVFPFIGIVCSYGRVFLAVYRMHSGAGKKKAYSTC
STHLTVVTFYYAPFAYTYLRPRSLRSPTEDKILAVFYTVLTPMLNPIIYSLRNKEVMGAM
KRMTHRICFAKI
>r261-1
MGGNQTLITQFILLGFPLSPRMQMLLFVLFSLFYAFTLLGNGTILGLICLDSRLHTPMYF
FLSHLAIVDIAYACNTVPQMLVNLMDPAKPISFAGCMTQTFLFLTFAHTECLLLVVMSYD
RYVAICHPLRYTVIMSWRVCVILVLTSWILGVLLALVHLVLLLPLPFCGSQKVNHFFCEI
IAVLKLACSDPRINEVMVLAGAVSVLVGPFSSIVVSYTHILCAILKIKSQQGRQKAFSTC
SSHLCVVGLFYGTAIVMYIGPQHGKSNEQKKYLLLFHSLFNPMLNPLIYSLRNKDVKSAL
KRMLIKEDTSSGC
>m30-1
MVASSNSSSHPLFFMLLGIPGLENYQFWIAFPFCVMYIVALTGNITILYIIRIDHTLHEP
MYLFLALLAITDLVLSSSTQPKMLAILWFHSHEIEYNACLIQVFFIHAFSSVESGVLMTM
ALDRYVAICFPLRHSSILTTSVVIKLGAAVMVRGLLWVSPFCFMVSRMPFCPNKVIPQSY
CEHMAVLKLVCADTRVNRGYGLFVAFSVVGFDIIVISVSYVMILRAVLRLPSGEARLKAF
GTCASHVCVILAFYIPALFTFLTHRFGHHVPRVVHIMFANFYLLVPPMLNPIIYGVRTKQ
IRDRVIRGFRRKDP
>r30-1
MVASSNSSSHPLFFILLGIPGLENYQFWIAFPFCVMYIVALTGNITILYIIQTDHTLHEP
MYLFLALLGITDLVLSSSTQPKMLAILWFHDHEIEYNACLIQVFFIHAFSSVESGVLMAM
ALDRYVAICFPLRHSSILTTSVVIKLGAAVMVRGLLWVSPFCFMISRMPFCPNNVIPQSY
CEHMAVLKLVCADTIVNRGYGLFVAFSVVGFDIIVISVSYVMILRAVLRLPSGEARLKAF
GTCASHVCVILAFYIPALFTFLTHRFGHHVPRVVHIIFANFYLLVPPMLNPIVYGVRTKQ
IRDRVIRGFRRKKLEPGITVAPGQ
>m33-1
MMLSAAIPNGTAFHPPTFVLLGIPGMQDQHVWIAIPFCSMYILALVGNGTILYIIITDRA
LHEPMYLFLCLLSITDLVLCSTTLPKMLAIFWLRSHVISYHGCLTQMFFVHAVFATESAV
LLAMAFDRYVAICRPLHYTSILNAVVIGKIGLACVTRGLLFVFPFVILIERLPFCGHHII
PHTYCEHMGIAKLACASIKPNTIYGLTVALSVTGMDVVLIATSYILILQAVLRLPSKDAQ
FRAFSTCGAHICVILVFYIPAFFSFFTHRFGHHVPPQVHIILANLYLLVPPVLNPLVYGI
NTKQIRLRILDFFVKRR
>r33-1

MMLSASIPNGTAFHPSTFVLLGIPGMQDQHVWIAIPFCSMYILALVGNGTILYIIITDRA
LHEPMYLFLCLLSITDLVLCSTTLPKMLAIFWLRSHIISYHGCLTQMFFVHAVFATESAV
LLAMAFDRYVAICRPLHYTSILNAVVIGKIGLACVTRGLLFVFPFVILIERLPFCGHHII
PHTYCEHMGIAKLACASIKPNTIYGLTVALSVTGMDVVLIATSYILILQAVLRLPSKDAQ
FRAFSTCGAHICVILVFYIPAFFSFFTHRFGHHVPPQVHIILANLYLLVPPVLNPLVYGI
NTKQIRLRILDFFVKRR
