# Supplementary material for: Functional Evolution of Mammalian Odorant Receptors
Source: PLoS Genet. 2012 Jul 12;8(7):e1002821. doi: 10.1371/journal.pgen.1002821 (PMC3395614; doi:10.1371/journal.pgen.1002821)
Supplement: Table S2 — Comparison of OR orthologs. For each pair of orthologs, the Jukes-Cantor distance (38), Grantham distance (37) of open reading frame (ORF) and 22 amino acids from Man et al. (2004) [17] (22AA), ω (dN/dS) and correlation values (R and p) for tuning curve responses are listed. (PDF) [file pgen.1002821.s015.pdf]

	OR	Evolutionary Distance Jukes-Cantor	Grantham Distance Grantham full length OR	Grantham 22 AA	ω =dN/dS	42 Odor Correlation R	p-value
Human-Chimp	OR10G3	0.007	2.00	0	0.261	0.971	<0.001
	OR10G7	0.022	15.03	0	0.546	0.896	<0.001
	OR10J5	0.008	9.86	0	3.003		
	OR11A1	0.023	20.42	0	0.545	0.986	<0.001
	OR1A1	0.009	3.01	0	0.401	0.979	<0.001
	OR2A25	0.008	2.35	0	0.324	0.840	0.366
	OR2B11	0.011	4.42	0	0.207	0.959	<0.001
	OR2J2	0.010	8.29	0	0.656	0.934	<0.001
	OR2J3	0.008	6.95	0	0.549	0.919	0.009
	OR2W1	0.006	6.25	0	Inf	0.973	<0.001
	OR51E1	0.008	1.32	0	0.141	0.962	<0.001
	OR51L1	0.005	7.14	0	2.430		
	OR56A4	0.005	5.44	0	0.937	0.311	0.689
	OR5K1	0.009	6.56	0	0.318	0.847	0.001
	OR5P3	0.001	0	0	0	0.997	<0.001
	OR8D1	0.008	4.17	0	0.301	0.388	0.124
	OR8K3	0.004	0	0	0	1.000	<0.001
Human-Macaque	OR10G3	0.042	11.86	0	0.187	0.438	0.178
	OR10G7	0.053	29.13	1.01	0.363	0.952	<0.001
	OR1A1	0.044	24.70	3.36	0.349	0.316	0.069
	OR1C1	0.053	20.50	0.58	0.339	0.851	0.352
	OR2A25	0.054	28.96	3.82	0.419	0.518	0.653
	OR2B11	0.041	16.26	0	0.209	0.959	<0.001
	OR2J3	0.036	12.22	0	0.200	0.954	0.003
	OR2W1	0.027	12.09	0	0.537	0.800	<0.001
	OR51E1	0.024	14.89	0	0.300	0.573	0.065
	OR56A4	0.038	48.01	0.20	0.450	−0.443	0.557
	OR5K1	0.028	9.21	0	0.267	0.715	0.013
	OR8D1	0.038	18.53	0	0.368	0.181	0.488
	OR8K3	0.048	29.25	0.45	0.346	1.000	<0.001
Chimp-Macaque	OR10G3	0.038	11.82	0	0.232	0.504	0.114
	OR10G7	0.058	32.34	1.01	0.331	0.943	<0.001
	OR1A1	0.047	22.99	3.36	0.308	0.355	0.040
	OR2A25	0.053	28.43	3.82	0.413	0.900	0.287
	OR2B11	0.040	14.26	0	0.222	0.929	<0.001
	OR2J3	0.037	13.19	0	0.260	0.982	<0.001
	OR2W1	0.025	12.12	0	0.389	0.881	<0.001
	OR51E1	0.026	15.48	0	0.350	0.496	0.121
	OR56A4	0.040	51.18	0.20	0.483	0.714	0.286
	OR5K1	0.034	15.55	0	0.269	0.933	<0.001
	OR8D1	0.036	15.22	0	0.328	0.230	0.375
	OR8K3	0.050	29.25	0.45	0.322	1.000	<0.001
Mouse-Rat	MOR129-1	0.056	11.68	0	0.118	0.996	<0.001
	MOR161-1	0.082	28.34	0	0.093		
	MOR162-1	0.062	27.69	0	0.278	0.992	<0.001
	MOR170-1	0.092	39.65	0	0.128	0.173	0.571
	MOR180-1	0.059	8.70	0	0.082		
	MOR184-1	0.064	14.64	0	0.052	0.911	<0.001
	MOR189-1	0.098	18.89	0	0.026	0.956	<0.001
	MOR203-1	0.070	17.09	0	0.120	0.826	0.022
	MOR207-1	0.041	6.74	0	0.155		
	MOR23-1	0.078	20.64	0	0.108	0.856	0.346
	MOR256-17	0.057	15.30	0	0.193	0.974	<0.001
	MOR260-1	0.069	19.93	1.18	0.189	0.755	0.003
	MOR261-1	0.077	7.62	0	0.271	0.999	<0.001
	MOR268-1	0.086	37.58	6.36	0.182	0.030	0.944
	MOR272-1	0.046	10.02	0	0.121	1.000	0.019
	MOR30-1	0.062	15.49	0	0.092	0.794	<0.001
	MOR33-1	0.048	3.98	0	0.185	0.976	<0.001
