# Supplementary material for: Functional Evolution of Mammalian Odorant Receptors
Source: PLoS Genet. 2012 Jul 12;8(7):e1002821. doi: 10.1371/journal.pgen.1002821 (PMC3395614; doi:10.1371/journal.pgen.1002821)
Supplement: Table S3 — Odors used in the study. Odors are listed by their common name, Chemical Abstract Service registry number (CAS#), and corresponding abbreviation used in the tuning curve data (Figure 2, Figure S6). (PDF) [file pgen.1002821.s016.pdf]

Common Odor Name	CAS #	Odor Abbreviation
methyl salicylate	119-36-8	MSAL
2-ethyl fenchol	18368-91-7	2EF
amyl alcohol (1-pentanol)	71-41-0	1PENT
coffee difuran (2,2 dithiomethelyne)	4437-20-1	COFFDF
Ethylenedichloride (benzene)	71-43-2	BENZ
2,4-DNT	121-14-2	24DNT
decyl aldehyde	112-31-2	DA
propionaldehyde (propanal)	123-38-6	PROP
ocean propanal (helional)	1205-17-0	HELI
dimethyl disulfide	624-92-0	DMDS
allyl phenyl acetate	1797-74-6	APA
eugenyl acetate	93-28-7	EUGACE
methyl furfuryl disulfide	57500-00-2	MFUR
laevo-arabinose	5328-37-0	LA
octyl octanoate	2306-88-9	OO
quinoline	91-22-5	QUIN
isobutyl amine	78-81-9	IA
pyrazine	290-37-9	PYRAZ
alpha-terpinyl acetate	80-26-2	TERPA
butyric acid	107-92-6	BACID
(+)-menthol	15356-60-2	+MEN
eugenol methyl ether	93-15-2	EUGME
dextro(r/(+))-limonene	5989-27-5	RLIM
citral	5392-40-5	CITRAL
amyl hexanoate	540-07-8	AMHEX
beta-damascone	23726-91-2	BDAM
butyl formate	592-84-7	BF
geranyl acetate	105-87-3	GA
androstenone	18339-16-7	AND
nonanoic acid	112-05-0	NONA
linalool	78-70-6	LIN
coumarin	91-64-5	COUM
eugenol	97-53-0	EUG
1-octanol	111-87-5	1OCT
octanethiol	111-88-6	OTHI
nonyl aldehyde	124-19-6	NONALD
2-heptanone	110-43-0	2HEPT
ethyl vanillin	121-32-4	EVAN
lyral	130066-44-3	LYRL
cis-3-hexen-1-ol	928-96-1	C3HEX
propionic acid	79-09-4	PROPA
(+)-carvone	2244-16-8	+CAR
4,5-Dimethyl-3-hydroxy-2,5-dihydrofuran-2-one	28664-35-9	45DIM
