# Supplementary material for: Functional Evolution of Mammalian Odorant Receptors
Source: PLoS Genet. 2012 Jul 12;8(7):e1002821. doi: 10.1371/journal.pgen.1002821 (PMC3395614; doi:10.1371/journal.pgen.1002821)
Supplement: Table S4 — Comparison of dose-response curves from orthologous sets. LogEC50 (M), Span (dynamic-range) for each OR is given. DNR, does not respond. F-ratio and p-values from extra sum-of-squares test. (PDF) [file pgen.1002821.s017.pdf]

PRIMATE	Log EC50 (M)			Span			F-test F (DFn,DFd)			P-value			Odor
	H	C	M	H	C	M	H to C	H to M	C to M	H to C	H to M	C to M	
OR1A1	-7.54	-7.16	DNR	5.98	6.61	DNR	3.70 (3,24)	806.59 (3,24)	1055.70 (3,24)	0.025*	<0.001**	<0.001**	(+)-carvone
OR10G3	-4.01	-5.25	DNR	2.48	4.00	DNR	783.36 (3,42)	66895.62 (3,42)	2016.07 (3,42)	<0.001**	<0.001**	<0.001**	ethyl vanillin
OR2A25	-5.09	-3.24	-5.47	1.89	1.37	2.71	250.67 (3,39)	10.35 (3,36)	227.53 (3,39)	<0.001**	<0.001**	<0.001**	geranyl acetate
OR2B11	-4.10	-3.07	-2.65	4.63	3.33	4.97	102.34 (3,36)	141.04 (3,36)	-2.22 (3,36)	<0.001**	<0.001**	1.0	coumarin
OR2W1	-7.35	-6.99	-5.79	3.91	3.73	1.98	56.74 (3,36)	775.93 (3,36)	375.88 (3,36)	<0.001**	<0.001**	<0.001**	allyl phenyl acetate
OR8K3	-4.32	-4.48	-4.48	4.20	4.13	5.69	0.13 (3,36)	15.16 (3,36)	17.40 (3,36)	0.944	<0.001**	<0.001**	(+)-menthol
OR2J3	-3.71	-4.44	-4.21	3.33	0.79	1.66	203.67 (3,36)	38.56 (3,36)	-8.28 (3,36)	<0.001**	<0.001**	1.0	geranyl acetate
OR8D1	-4.99	-4.43	-4.31	7.20	2.11	0.62	1152.55 (3,42)	2148.79 (3,42)	460.25 (3,42)	<0.001**	<0.001**	<0.001**	4,5-dimethyl-3-hydroxy-2,5-dihydrofuran-2-one
OR10G7	-7.99	-6.19	-7.60	1.49	1.19	1.12	54.54 (3,36)	0.97 (3,36)	84.82 (3,36)	<0.001**	0.418	<0.001**	eugenol
OR51E1	-4.47	-5.00	DNR	5.04	3.77	DNR	12.55 (3,42)	608.78 (3,42)	564.64 (3,42)	<0.001**	<0.001**	<0.001**	butyric acid
OR56A4	-3.41	-2.71	DNR	5.24	1.40	DNR	605.01 (3,39)	173.41 (3,39)	4.49 (3,42)	<0.001**	<0.001**	0.008*	decyl aldehyde
OR5K1	-7.26	-5.85	-5.05	4.48	2.65	2.72	194.74 (3,36)	243.17 (3,36)	47.74 (3,36)	<0.001**	<0.001**	<0.001**	eugenol methyl ether
OR10J5	-4.14	-3.96		11.48	0.13		1214.71 (3,36)			<0.001**			lyral
OR11A1	-8.33	-6.34		3.10	1.45		556.99 (3,42)			<0.001**			2-ethyl fenchol
OR1C1	-5.09		-5.19	0.55		0.03		296.62 (3,42)			<0.001**		linalool
OR51L1	-5.04	-4.79		7.42	6.54		107.23 (3,42)			<0.001**			allyl phenyl acetate
OR5P3	-5.72	-5.35		9.99	11.66		2.08 (3,36)			0.120			coumarin
OR2J2	-5.89	-6.94		2.57	3.07		63.27 (3,42)			<0.001**			1-octanol
**RODENT**	**Log EC50 (M)**			**Span**			**F-test F (DFn,DFd)**			**P-value**			**Odor**
	Mouse	Rat		Mouse	Rat		Mouse to Rat			Mouse to Rat			
MOR129-1	-4.21	-4.14		8.11	12.20		132.79 (3,30)			<0.001**			coumarin
MOR180-1	-3.23	-3.15		2.71	1.93		29.86 (3,36)			<0.001**			r-limonene
MOR23-1	-5.19	-5.30		2.47	2.02		169.15 (3,32)			<0.001**			nonanoic acid
MOR256-17	-4.35	-4.30		3.17	2.99		-0.57 (3,42)			1.0			coumarin
MOR260-1	-9.71	-6.04		1.77	2.37		482.92 (3,42)			<0.001**			1-octanol
MOR268-1	-5.64	-7.89		0.94	3.16		553.27 (3,42)			<0.001**			1-octanol
MOR161-1	-3.66	-3.57		4.33	3.67		8.92 (3,36)			<0.001**			coumarin
MOR184-1	-4.84	-4.28		10.27	8.15		67.70 (3,42)			<0.001**			(+)-carvone
MOR189-1	-3.65	-3.82		2.24	3.38		18.57 (3,36)			<0.001**			coumarin
MOR162-1	-4.51	-4.41		8.52	7.58		9.33 (3,42)			<0.001**			coumarin
MOR170-1	-3.76	DNR		6.35	DNR		10912.92 (3,42)			<0.001**			coumarin
MOR203-1	-5.05	-3.89		3.98	0.15		861.33 (3,42)			<0.001**			(+)-carvone
MOR207-1	-4.16	-3.39		5.42	2.97		459.19 (3,33)			<0.001**			coumarin
MOR30-1	-4.52	-6.47		17.08	6.76		167.38 (3,42)			<0.001**			nonanoic acid
MOR261-1	-6.86	-6.70		18.18	21.80		25.93 (3,42)			<0.001**			1-octanol
MOR33-1	-6.45	-6.46		8.45	10.30		59.00 (3,42)			<0.001**			octanoic acid
MOR272-1	-7.72	-7.01		8.65	3.76		462.76 (3,36)			<0.001**			(+)-carvone
