# Supplementary material for: Functional Evolution of Mammalian Odorant Receptors
Source: PLoS Genet. 2012 Jul 12;8(7):e1002821. doi: 10.1371/journal.pgen.1002821 (PMC3395614; doi:10.1371/journal.pgen.1002821)
Supplement: Table S5 — Comparison of dose-response curves from orthologs and paralogs. LogEC50 (M), Span (dynamic-range) for each OR is given. DNR, does not respond. F-ratio and p-values from extra sum-of-squares test. (PDF) [file pgen.1002821.s018.pdf]

OR	Log EC50 (M)	Span	F-test F (DFn,DFd) to hOR	P-value	Odor
h1A1	-5.92	11.40			(+)-carvone
c1A1	-5.59	13.05	29.03 (3,30)	<0.001**	
m1A1	DNR	DNR	639.49 (3,30)	<0.001**	
h1A2	DNR	DNR	657.14 (3,30)	<0.001**	
MOR125-1	DNR	DNR	640.39 (3,30)	<0.001**	
pCI	DNR	DNR	637.64 (3,30)	<0.001**	
h2W1	-5.22	13.14			allyl phenyl acetate
c2W1	-5.11	9.40	407.63 (3,42)	<0.001**	
m2W1	-4.28	6.49	2188.15 (3,42)	<0.001**	
h2W3	DNR	DNR	4456.41 (3,42)	<0.001**	
h2W5	DNR	DNR	4498.57 (3,42)	<0.001**	
pCI	DNR	DNR	4337.53 (3,42)	<0.001**	
h51E1	-4.24	11.54			butyric acid
c51E1	-4.15	15.44	4.66 (3,42)	0.007*	
m51E1	DNR	DNR	3453.62 (3,42)	<0.001**	
h51E2	DNR	DNR	3379.58 (3,42)	<0.001**	
MOR18-1	-3.92	15.41	35.93 (3,42)	<0.001**	
pCI	DNR	DNR	3526.53 (3,42)	<0.001**	
h8K3	-4.96	3.47			(+)-menthol
c8K3	-5.04	2.57	6.91 (3,42)	<0.001**	
m8K3	-4.75	2.31	48.25 (3,42)	<0.001**	
h8K1	DNR	DNR	524.45 (3,42)	<0.001**	
h8K5	DNR	DNR	711.87 (3,42)	<0.001**	
pCI	-4.68	-0.15	660.95 (3,42)	<0.001**	
h10G3	-4.38	16.96			ethyl vanillin
c10G3	-5.50	17.88	425.80 (3,42)	<0.001**	
m10G3	DNR	DNR	6610.46 (3,42)	<0.001**	
h10G4	-4.39	2.55	2608.06 (3,42)	<0.001**	
h10G6	-3.23	0.52	7442.57 (3,42)	<0.001**	
h10G7	-4.62	13.70	13.89 (3,42)	<0.001**	
h10G8	DNR	DNR	7502.57 (3,42)	<0.001**	
h10G9	-3.27	6.38	5017.64 (3,42)	<0.001**	
MOR223-5	-6.49	9.56	43.26 (3,42)	<0.001**	
pCI	DNR	DNR	7237.20 (3,42)	<0.001**	
h10G7	-7.17	8.17			eugenol
c10G7	-6.34	9.14	155.22 (3,42)	<0.001**	
m10G7	-6.87	8.26	12.16 (3,42)	<0.001**	
h10G3	-3.88	4.19	976.95 (3,42)	<0.001**	
h10G4	-4.49	0.25	1065.52(3,42)	<0.001**	
h10G6	DNR	DNR	1066.38 (3,42)	<0.001**	
h10G8	DNR	DNR	1080.47 (3,42)	<0.001**	
h10G9	DNR	DNR	1067.42 (3,42)	<0.001**	
MOR223-3	-6.43	5.36	259.40 (3,42)	<0.001**	
pCI	DNR	DNR	1018.53 (3,42)	<0.001**	
h5K1	-4.62	19.24			eugenol methyl ether
c5K1	-3.97	8.79	1647.40 (3,42)	<0.001**	
m5K1	-3.51	6.52	2179.01 (3,42)	<0.001**	
h5K2	DNR	DNR	2535.15 (3,42)	<0.001**	
h5K3	DNR	DNR	2610.65 (3,42)	<0.001**	
h5K4	DNR	DNR	2642.73 (3,42)	<0.001**	
MOR184-3	-5.23	19.09	21.59 (3,39)	<0.001**	
pCI	DNR	DNR	2664.95 (3,42)	<0.001**	

OR	Log EC50 (M)	Span	F-test F (DFn,DFd) to hOR	P-value	Odor
h2A25	-5.02	12.13			geranyl acetate
c2A25	-3.38	9.89	1157.56 (3,36)	<0.001**	
m2A25	-4.74	11.93	56.88 (3,36)	<0.001**	
h2A2	DNR	DNR	1650.87 (3,36)	<0.001**	
h2A4	DNR	DNR	1463.08 (3,36)	<0.001**	
h2A5	DNR	DNR	1326.92 (3,36)	<0.001**	
h2A7	DNR	DNR	1770.44 (3,36)	<0.001**	
h2A12	DNR	DNR	1644.75 (3,36)	<0.001**	
h2A14	DNR	DNR	1770.09 (3,36)	<0.001**	
h2A42	DNR	DNR	1362.92 (3,36)	<0.001**	
MOR261-1	-6.73	4.61	152.77 (3,34)	<0.001**	
pCI	-3.53	4.41	1038.94 (3,36)	<0.001**	
h10J5	-4.32	29.25			lyral
c10J5	-2.95	5.82	3812.91 (3,39)	<0.001**	
h10J1	-3.14	4.39	3810.04 (3,39)	<0.001**	
h10J3	DNR	DNR	2483.29 (3,36)	<0.001**	
MOR267-13	-4.68	12.98	143.02 (3,39)	<0.001**	
pCI	DNR	DNR	4003.96 (3,39)	<0.001**	
h8D1	-5.38	16.73			4,5-dimethyl-3-hydroxy-2,5-dihydrofuran-2-one
c8D1	-4.73	3.85	1167.60 (3,42)	<0.001**	
m8D1	-4.80	5.52	916.29 (3,42)	<0.001**	
h8D2	DNR	DNR	1952.08 (3,42)	<0.001**	
h8D4	DNR	DNR	1868.58 (3,42)	<0.001**	
MOR171-22	-4.48	6.49	873.69 (3,42)	<0.001**	
MOR171-9	DNR	DNR	1959.16 (3,42)	<0.001**	
pCI	DNR	DNR	1828.41 (3,42)	<0.001**	
h2B11	-4.14	8.23			coumarin
c2B11	-3.55	14.08	33.69 (3,42)	<0.001**	
m2B11	-3.48	13.38	19.17 (3,42)	<0.001**	
h2B2	DNR	DNR	827.54 (3,39)	<0.001**	
h2B3	DNR	DNR	915.43 (3,39)	<0.001**	
h2B6	DNR	DNR	847.34 (3,39)	<0.001**	
h2B8	DNR	DNR	959.72 (3,39)	<0.001**	
pCI	DNR	DNR	770.34 (3,39)	<0.001**	
h56A4	-3.72	14.68			decyl aldehyde
c56A4	-3.16	6.09	8609.47 (3,42)	<0.001**	
m56A4	DNR	DNR	15415.03 (3,42)	<0.001**	
h56A5	-3.79	18.41	112.30 (3,42)	<0.001**	
h56A1	-3.24	10.33	10679.58 (3,42)	<0.001**	
h56A3	-3.90	0.79	1949.15 (3,42)	<0.001**	
pCI	-3.01	1.25	10551.55 (3,42)	<0.001**	
h5P3	-5.53	14.49			coumarin
c5P3	-5.42	15.09	0.94 (3,36)	0.43	
h5P2	DNR	DNR	556.80 (3,36)	<0.001**	
MOR204-6	-4.70	13.47	51.89(3,36)	<0.001**	
pCI	DNR	DNR	568.41 (3,36)	<0.001**	
h2J2	-5.13	3.61			1-ocatnol
c2J2	-5.36	1.71	81.75 (3,42)	<0.001**	
h2J1	-3.95	2.33	901.15 (3,42)	<0.001**	
h2J3	-5.73	3.38	198.08 (3,42)	<0.001**	
MOR256-18	-5.56	8.55	443.59 (3,42)	<0.001**	
pCI	-3.57	1.11	1122.35 (3,42)	<0.001**	
h2J3	-3.79	9.42			geranyl acetate
c2J3	DNR	DNR	268.99 (3,36)	<0.001**	
m2J3	-3.28	12.58	149.22 (3,36)	<0.001**	
h2J1	-3.32	8.08	264.66 (3,36)	<0.001**	
h2J2	-4.37	3.60	80.16 (3,36)	<0.001**	
MOR256-18	-4.44	6.38	17.58 (3,36)	<0.001**	
pCI	-2.59	27.52	381.71 (3,36)	<0.001**	
