# Supplementary material for: Functional Evolution of Mammalian Odorant Receptors
Source: PLoS Genet. 2012 Jul 12;8(7):e1002821. doi: 10.1371/journal.pgen.1002821 (PMC3395614; doi:10.1371/journal.pgen.1002821)
Supplement: Table S7 — Live cell-surface expression of individual receptors. For each receptor in a group, the average Cy3 intensity in arbitrary units (a.u.) (n = 3, ± S.E.M.) and p-value to the main human OR in the group is given. S6 is positive control and Rho-pCI is negative control. (PDF) [file pgen.1002821.s020.pdf]

OR	Average Cy3 Intensity (arbitrary units)	S.E.M.	P-value to hOR
h1A1	38796.33	470.10	
c1A1	39870.67	5819.16	0.86
m1A1	6979.67	1558.60	<0.001***
h1A2	3485.67	1205.40	<0.001***
MOR125-1	9900.00	6575.93	<0.05*
S6	60269.33	11116.38	0.13
pCI	3637.33	898.23	<0.001***
h2W1	55136.67	10149.87	
c2W1	44339.33	2605.18	0.36
m2W1	66935.67	6579.17	0.38
h2W3	77207.67	18000.20	0.35
h2W5	56392.67	4779.72	0.92
S6	60269.33	11116.38	0.75
pCI	3637.33	898.23	<0.01**
h51E1	147512.33	23875.55	
c51E1	49652.00	11425.13	<0.05*
m51E1	41031.67	4498.55	<0.05*
h51E2	14710.67	1984.41	<0.01**
MOR18-1	154629.33	37431.41	0.88
S6	59396.00	13552.79	<0.05*
pCI	21339.67	1155.63	<0.01**
h8K3	28239.67	1391.77	
c8K3	99231.67	26490.79	0.06
m8K3	86757.33	5626.56	<0.001***
h8K1	41101.33	7216.89	0.16
h8K5	135019.67	72469.58	0.21
S6	87659.67	1055.77	<0.001***
pCI	18970.67	3226.28	0.06
h10G3	64375.33	18075.76	
c10G3	63061.33	29658.53	0.97
m10G3	48767.33	5678.05	0.46
h10G4	36686.67	15334.99	0.31
h10G6	200780.00	3917.69	<0.01**
h10G7	30347.33	2684.86	0.14
h10G8	27246.33	2366.13	0.11
h10G9	45641.33	13863.31	0.46
MOR223-5	226760.67	18036.37	<0.01**
S6	111108.33	20213.17	0.07
pCI	19514.00	1103.83	0.16
h10G7	30347.33	2684.86	
c10G7	114261.00	9910.63	<0.01**
m10G7	67425.67	14382.84	0.06
h10G3	64375.33	18075.76	0.14
h10G4	36686.67	15334.99	0.70
h10G6	200780.00	3917.69	<0.001***
h10G8	27246.33	2366.13	0.44
h10G9	45641.33	13863.31	0.34
MOR223-3	70963.33	14050.01	<0.05*
S6	111108.33	20213.17	<0.05*
pCI	19514.00	1103.83	<0.05*
h5K1	213646.00	32401.61	
c5K1	56569.00	7447.62	<0.01**
m5K1	58177.67	13535.79	<0.05*
h5K2	21903.00	4403.95	<0.01**
h5K3	16691.67	6639.33	<0.01**
h5K4	20481.33	1328.90	<0.01**
MOR184-3	223080.67	42627.65	0.87
S6	127888.00	17764.53	0.08
pCI	8428.00	539.40	<0.01**
h5P3	139664.33	9824.49	
c5P3	180789.67	32566.39	0.29
h5P2	43046.33	6565.51	<0.01**
MOR204-6	53418.33	14704.63	<0.01**
S6	127888.00	17764.53	<0.01**
pCI	87659.67	1055.77	<0.001***

OR	Average Cy3 Intensity (arbitrary units)	S.E.M.	P-value to hOR
h2A25	102380.33	29015.45	
c2A25	10303.33	1299.96	<0.05*
m2A25	26672.00	1112.92	0.06
h2A2	42464.33	9727.95	0.12
h2A4	34691.33	3210.00	0.08
h2A5	90090.33	33740.56	0.80
h2A7	26280.67	4330.63	0.06
h2A12	19814.67	6726.69	0.05
h2A14	140245.33	35035.17	0.45
h2A42	15264.33	3448.74	<0.05*
MOR261-1	92716.67	19444.32	0.80
S6	67217.67	7288.73	0.31
pCI	8434.67	4529.58	<0.05*
h10J5	26989.00	6700.39	
c10J5	17079.67	1885.40	0.23
h10J1	18271.33	1007.72	0.27
h10J3	24796.67	2843.45	0.78
MOR267-13	32429.33	4157.43	0.53
S6	72563.00	9033.07	<0.05*
pCI	14888.33	1657.51	0.15
h8D1	98875.33	17801.75	
c8D1	35679.33	8456.27	<0.05*
m8D1	24419.33	2473.52	<0.05*
h8D2	69462.33	6880.05	0.20
h8D4	36008.00	8284.55	<0.05*
MOR171-22	18129.33	1282.00	<0.01**
MOR171-9	15465.00	1968.57	<0.05*
S6	72563.00	9033.07	0.26
pCI	14888.33	1657.51	<0.01**
h2B11	101000.67	11900.30	
c2B11	183058.33	17549.90	<0.05*
m2B11	102338.67	5248.42	0.92
h2B2	21537.67	737.70	<0.01**
h2B3	20781.00	1713.62	<0.01**
h2B6	33121.00	6995.35	<0.01**
h2B8	24680.67	5922.72	<0.01**
S6	59396.00	13552.79	0.08
pCI	21339.67	1155.63	<0.01**
h56A4	45746.33	12989.70	
c56A4	34285.00	5474.29	0.46
m56A4	24827.00	4904.81	0.21
h56A5	57058.67	1246.14	0.43
h56A1	63605.00	19531.93	0.49
h56A3	12990.00	8050.82	0.10
S6	127888.00	17764.53	<0.05*
pCI	8428.00	539.40	<0.05*
h2J2	610993.00	3226.28	
c2J2	389365.67	82769.59	0.08
h2J1	186332.00	1357.49	<0.01**
h2J3	43645.00	45526.27	<0.001***
MOR256-18	263093.33	36086.15	<0.01**
S6	230776.67	39370.33	<0.01**
pCI	40520.67	2977.18	<0.001***
h2J3	43645.00	1357.49	
c2J3	65184.00	3069.23	<0.01**
m2J3	83413.33	23788.08	0.17
h2J2	610993.00	46072.06	<0.001***
h2J1	186332.00	45526.27	<0.05*
MOR256-18	263093.33	36086.15	<0.01**
S6	230776.67	39370.33	<0.01**
pCI	40520.67	2977.18	0.39
