# Supplementary material for: Functional Evolution of Mammalian Odorant Receptors
Source: PLoS Genet. 2012 Jul 12;8(7):e1002821. doi: 10.1371/journal.pgen.1002821 (PMC3395614; doi:10.1371/journal.pgen.1002821)
Supplement: Table S6 — Grantham's distance for orthologs and paralogs. The Grantham's distance was calculated for OR open reading frame (ORF) and for 22 amino acids (22AA) used in Man et al. (2004) [17]. Each ortholog and paralog is compared to the reference human OR in that group (listed first). The distribution of orthologs to paralogs using ORF and 22A was significantly different (z = −6.61, p<0.0001 ORF; z = −7.35, p<0.0001 22AA, Wilcoxon Rank Sum), as was the distribution of paralogs that did or did not respond using 22AA (z = −3.54, p<0.0004, Wilcoxon Rank Sum) (Figure 7). (PDF) [file pgen.1002821.s019.pdf]

OR	Grantham's Distance 22 AA	ORF
h1A1		
c1A1	0.00	3.01
m1A1	3.36	24.70
h1A2	6.36	65.03
MOR125-1	1.77	57.54
h2W1		
c2W1	0.00	6.25
m2W1	0.00	12.09
h2W3	8.09	191.25
h2W5	14.54	301.82
h51E1		
c51E1	0.00	1.32
m51E1	0.00	16.87
h51E2	5.38	156.77
MOR18-1	0.00	23.61
h8K3		
c8K3	0.00	0.00
m8K3	0.45	29.25
h8K1	13.42	131.18
h8K5	18.12	110.02
h10G3		
c10G3	0.00	2.00
m10G3	0.00	11.86
h10G4	7.33	182.58
h10G6	6.24	182.30
h10G7	10.98	182.62
h10G8	13.09	206.50
h10G9	7.76	185.68
MOR223-5	0.00	50.04
h10G7		
c10G7	0.00	15.03
m10G7	1.01	29.13
h10G3	10.98	182.62
h10G4	3.70	44.58
h10G6	10.40	211.17
h10G8	2.10	47.74
h10G9	4.13	38.86
MOR223-3	0.00	49.74
h5K1		
c5K1	0.00	6.56
m5K1	0.00	9.21
h5K2	1.86	22.63
h5K3	9.61	82.43
h5K4	6.14	96.32
MOR184-3	0.58	50.42
h2A25		
c2A25	0.00	2.35
m2A25	3.82	28.96
h2A2	17.14	162.66
h2A4	14.01	106.30
h2A5	12.51	142.78
h2A7	14.01	105.36
h2A12	15.22	145.50
h2A14	16.71	159.47
h2A42	16.89	121.81
MOR261-1	5.42	75.03

OR	Grantham's Distance 22 AA	ORF
h10J5		
c10J5	0.00	9.86
h10J1	8.61	121.54
h10J3	9.25	180.81
MOR267-13	3.82	51.79
h8D1		
c8D1	0.00	4.17
m8D1	0.00	21.88
h8D2	4.74	111.69
h8D4	11.45	144.01
MOR171-22	0.00	63.70
MOR171-9	1.18	63.31
h2B11		
c2B11	0.00	4.42
m2B11	0.00	16.26
h2B2	9.09	165.99
h2B3	11.15	161.88
h2B6	18.22	166.54
h2B8	13.52	168.57
h56A4		
c56A4	0.00	5.44
m56A4	0.20	50.18
h56A5	2.86	49.87
h56A1	3.05	50.45
h56A3	3.45	79.67
h5P3		
c5P3	0.00	0.00
h5P2	7.49	155.53
MOR204-6	1.14	95.24
h2J2		
c2J2	0.00	28.58
h2J1	1.76	46.82
h2J3	3.03	60.48
MOR256-18	2.34	84.52
h2J3		
c2J3	0.00	6.95
m2J3	0.00	12.22
h2J1	1.27	54.07
h2J2	3.03	60.48
MOR256-18	1.85	79.32

**Wilcoxon Rank Sum**		**z**	**p**
**Orthologs vs Paralogs**		**22AA**	
		-6.61	<0.0001**
		**ORF**	
		-7.35	<0.0001**
**Response vs No Response**			
	**Orthologs**	**22AA**	
		1.22	0.22
		**ORF**	
		0.89	0.37
	**Paralogs**	**22AA**	
		-3.54	<0.0004**
		**ORF**	
		-1.47	0.14
